# Molecular Typing of MRSA and of Clinical *Staphylococcus aureus* Isolates from Iaşi, Romania

**DOI:** 10.1371/journal.pone.0097833

**Published:** 2014-05-20

**Authors:** Stefan Monecke, Elke Müller, Olivia Simona Dorneanu, Teodora Vremeră, Ralf Ehricht

**Affiliations:** 1 Institute for Medical Microbiology and Hygiene, Medizinische Fakultät “Carl Gustav Carus”, Technische Universität Dresden, Dresden, Germany; 2 Alere Technologies GmbH, Jena, Germany; 3 Microbiology Department, University of Medicine and Pharmacy “Grigore T. Popa” Iaşi, Iaşi, Romania; Rockefeller University, United States of America

## Abstract

Romania is one of the countries with the highest prevalence of methicillin-resistant *Staphylococcus aureus* (MRSA) in the world. To obtain data on affiliation of MRSA to strains and clonal complexes and on the population of methicillin susceptible *S. aureus* (MSSA), clinical isolates from bloodstream infections, skin and soft tissue infections as well as from screening swabs were collected at hospitals in Ia?i, a city in the North-Eastern part of Romania. Isolates were characterised by microarray hybridisation. Nearly half of all isolates (47%), and about one third (34%) of bloodstream isolates were MRSA. The prevalence of the Panton-Valentine leukocidin (PVL) was also high (31% among MRSA, 14% among MSSA). The most common MRSA strain was a PVL-negative CC1-MRSA-IV that might have emerged locally, as a related MSSA was also common. PVL-positive CC8-MRSA-IV (“USA300”) and PVL-negative ST239-like MRSA-III were also frequently found while other MRSA strains were only sporadically detected. Among MSSA, PVL-positive CC121 as well as PVL-negative CC1, CC22 and CC45 predominated. Although this study provides only a snapshot of *S. aureus*/MRSA epidemiology in Romania, it confirms the high burden of MRSA and PVL on Romanian healthcare settings.

## Introduction

The healthcare systems in Eastern European countries in general, and Romania in particular, have been significantly affected by the political changes in the 1990s, de-centralisation, transition to market-orientated economies, as well as various political and economic crises [1], [2], [3], [4].

With the public attention being focused mainly on HIV and tuberculosis, there are only few data available on other issues such as, *e.g.,* antibiotic resistance in various pathogens such as *Staphylococcus aureus*. Since its first description roughly 50 years ago, methicillin-resistant *S. aureus* (MRSA) has globally become a public health threat. While there is an abundance of epidemiological and typing data from Western Europe, the United States and Australia, relatively few data are available for other parts of the world. Unfortunately, this is also true for Eastern Europe including Romania. It is known that the MRSA rates are extremely high, ranging from approximately 30% up to 70% in recent Romanian studies [5], [6], [7], [8], [9], [10], [11], [12], thus reaching the highest prevalence levels reported anywhere in the world. According to the ECDC antimicrobial resistance surveillance (formerly EARSS), the MRSA rate among invasive infections was “equal to or above 25%” in 2008 and slightly above 50% in 2011/2012 [9]; [http://www.ecdc.europa.eu/en/publications/publications/antimicrobial-resistance-surveillance-europe-2011.pdf, http://www.ecdc.europa.eu/en/publications/publications/antimicrobial-resistance-surveillance-europe-2012.pdf]. PVL-positive *S. aureus/*MRSA have also occasionally been described from Romania [8], [10], [12].

The purpose of the study was to characterise in detail a collection of *S. aureus* recovered between 2008–2012 in a large tertiary care hospital serving the North-Eastern part of Romania in order to obtain a detailed overview of the range of *S. aureus* clones and strains prevalent in this part of Romania.

## Materials and Methods

### Setting

The *S. aureus* isolates were collected at “Sf. Parascheva” Hospital for Infectious Diseases in Ia?i, Romania. Ia?i is a city of about 260,000 inhabitants in the North-Eastern part of Romania (47°09′25″N 27°35′25″E). The 300-bed university hospital mainly serves the Ia?i district (∼800,000 people). Moreover, patients with severe infections may be transferred to this hospital from the entire North East of Romania. It has an Intensive Care Unit (ICU) and a further ward for HIV-infected patients and is also specialized in treating patients with infections acquired in other medical settings.

### Isolates

Isolates from skin and soft tissue infections (SSTI) and blood cultures as well as MRSA from miscellaneous samples were included. These isolates originated from routine diagnostic tasks and were routinely preserved during the years 2008–2012. Carriage isolates were collected in 2012; and they originated from diagnostic tasks or from admission screening.

Isolates were retrospectively selected from frozen stocks (using cryobeads by AES Chemunex, Combourg, France) in order to test all available blood culture, SSTI and carriage/screening isolates of both, methicillin-susceptible *S. aureus* (MSSA) and MRSA, as well as additional, MRSA from other diagnoses. Only one isolate per patient was considered. This resulted in a total of 97 MRSA and 111 MSSA isolates. The carriage/screening group consisted of 50 isolates (17 MRSA and 33 MSSA). The blood culture group comprised 76 isolates (26 MRSA and 50 MSSA). The SSTI group included 61 isolates (33 MRSA and 28 MSSA), Additional MRSA isolates originated from pulmonary specimens (n = 14), cerebrospinal fluid (n = 4) or unspecified swabs (n = 3). Basic demographic and clinical information for the individual isolates is provided in the File S1.

Ethic committees consent was not requested since isolates were not purposefully obtained for this study.

### Microarray Procedures

Microarray procedures as well as lists of probes and primers were published previously [21], [22]. In short, isolates are grown overnight, harvested and enzymatically digested prior to DNA purification. Then, a linear primer elongation reaction is used to simultaneously label and amplify 333 target sequences corresponding to about 170 genes and their alleles. These include resistance markers, SCC*mec*-associated genes as well as genes encoding virulence and adhesion factors. Resulting single-stranded, biotin-labelled DNA amplicons are hybridised to arrays with spotted specific oligonucleotides. Hybridisations are visualised by adding a streptavidin-horseradish-peroxidase conjugate that triggers in a later step a local precipitation of a dye.

Arrays are scanned and normalised intensities of the spots were determined based on their average intensities and on the local background [21], [22]. Results were regarded as negative if the normalised intensity for a given probe was below 25% of the median of predefined species markers and a staining control [21]. If the normalised intensity for a given probe was higher than 50% of this median, it was interpreted positive. Values between 25% and 50% were regarded as “ambiguous” indicating technical issues, or, for some genes a presence on low copy number plasmids (*e.g*., *aacA-aphD*) as well as cross-reactions with other alleles of the same gene or with closely related genes. For some markers, for which allelic variants were to be discriminated that differ only in single nucleotides (*bbp, clfA, clfB, fnbB,* some *set/ssl* genes, *isaB, mprF* and *isdA*), a different approach was used. For these genes only the probe with the strongest signal value was considered positive (provided that it exceeded the 50% breakpoint). All others were regarded as “ambiguous” or, if below the 25% breakpoint, as negative.

The affiliations to clonal complexes (CC), SCC*mec* types and epidemic strains (Table 1) were determined automatically. For CC recognition, this approach relies on a comparison to reference profiles of independently identified strains in a database as well as on the absence/presence of certain marker genes (*e.g*., *fosB*, collagen adhaesin gene *cna*, *sasG*, enterotoxin gene cluster *egc*, enterotoxin H gene *seh,* ORF CM14) or to distinct alleles (*e.g*., *agr*-genes, capsule genes, genes encoding adhesion factors and proteases, *ssl/set*-, *hysA*- and *hsdS*-genes) that mirror the phylogenetic background of a given strain or isolate. Analysis of hybridisation patterns cannot discriminate sequence types which differ only in single point mutations affecting MLST genes (*e.g*., ST5, ST73, ST125, ST149, ST225 and ST228); and for that reason here will usually be referred to CC affiliations rather than to sequence types (ST). However, there are also STs that differ with regard to hybridisation patterns from their parental CCs due to chromosomal replacements or due to other, not yet elucidated reasons. In this study, these include ST239-like isolates (belonging to CC8 but differing in presence of *cna*, capsule type 8 and the alleles of *aur, clfB and isaB*; including ST239, ST240, ST241) and ST291/813-like isolates (belonging to CC398 but lacking *coa* and *cna*; harbouring *etD+edinB, lukE* and *spl* genes and differing in alleles of *ssl01, ebpS, sdrD, and hsdS*). Other examples for such divergent sequence types (that were not found in this study) are ST34/42-, ST72-, and ST573/772-like strains that belong to CC30, CC8 and CC1, respectively.

**Table 1:** Population structures for the total sample and for the isolates from different diagnoses.

Clonal complex	Strain	Total		Carriage isolates		Blood culture isolates		SSTI isolates		Other MRSA
		n	%	n	%	n	%	n	%	n
**CC1**	**CC1-MSSA**	17	8.2	4	8.0	6	7.9	7	11.5	
	**CC1-MRSA-IV**	40	19.2	12	24.0	9	11.8	12	19.7	7
**CC5**	**CC5-MSSA**	6	2.9	1	2.0	2	2.6	3	4.9	
	**CC5-MSSA [PVL+]**	1	0.5			1	1.3			
	**CC5-MRSA-II**	2	1.0							2
	**CC5-MRSA-IV**	1	0.5							1
**CC6**	**CC6-MSSA**	1	0.5			1	1.3			
**CC7**	**CC7-MSSA**	3	1.4	2	4.0	1	1.3			
**CC8**	**CC8-MSSA**	2	1.0			1	1.3	1	1.6	
	**CC8-MSSA [PVL+]**	3	1.4			1	1.3	2	3.3	
	**ST239-like MRSA-III**	22	10.6			7	9.2	4	6.6	11
	**CC8-MRSA-IV [PVL+/ACME+]**	24	11.5	5	10.0	4	5.3	15	24.6	
	**CC8-MRSA-IV [PVL+/ACME-]**	1	0.5					1	1.6	
**CC10**	**CC10-MSSA**	2	1.0	1	2.0			1	1.6	
**CC15**	**CC15-MSSA**	4	1.9	2	4.0	1	1.3	1	1.6	
**CC22**	**CC22-MSSA**	26	12.5	10	20.0	11	14.5	5	8.2	
	**CC22-MRSA-IV**	1	0.5			1	1.3			
**CC25**	**CC25-MSSA**	1	0.5			1	1.3			
**CC30**	**CC30-MSSA**	4	1.9	2	4.0	2	2.6			
**CC45**	**CC45-MSSA**	17	8.2	7	14.0	8	10.5	2	3.3	
	**CC45-MSSA [PVL+]**	1	0.5			1	1.3			
**CC80**	**CC80-MRSA-IV [PVL+]**	5	2.4			4	5.3	1	1.6	
**CC88**	**CC88-MSSA**	1	0.5			1	1.3			
**CC97**	**CC97-MSSA**	5	2.4	3	6.0	1	1.3	1	1.6	
**CC121**	**CC121-MSSA**	3	1.4			3	4.0			
	**CC121-MSSA [PVL+]**	10	4.8			6	7.9	4	6.6	
**CC133**	**CC133-MSSA**	1	0.5					1	1.6	
**CC188**	**CC188-MSSA**	1	0.5	1	2.0					
**CC398**	**ST291/813-like MSSA**	1	0.5			1	1.3			
	**CC398-MRSA-V**	1	0.5			1	1.3			
**CC1021**	**CC1021-MSSA**	1	0.5			1	1.3			

Strains were then defined based on their assignment to lineages as discussed above as well as on the presence or absence of pre-defined marker genes (*mecA, ccr* genes and other SCC*mec*-specific markers, *fusC*, ACME genes, *lukF/S*-PV).

### *sasX* PCR

The *sasX* gene was recently described as a factor that could be related to the spread of the MRSA strain ST239-like MRSA-III [23]. As these MRSA were commonly found in Ia?i, a PCR for *sasX* detection was established.

Primers were 01_sasX_fwd (ATTGAAGCTCAGACTCCTAG) and 02_sasX_Saur-rev (GTTATCAGTTGTAGCAGTAGT), with 0.15 µl each being used to achieve a final concentration of 1 µM each. Furthermore, the PCR master mix (14.5 µl per reaction) included 1.5 µl Genaxxon buffer (10x), 0.9 µl MgCl_2_ (25 mM, resulting in a final concentration of 1.5 mM), 0.15 µl Taq Polymerase (all Genaxxon M3001.0500), 0.15 µl dNTPs (10 mM, resulting in a final concentration of 0.1 mM) as well as 11.5 µl PCR-grade water. Finally, 0.5 µl DNA were added that was prepared as previously described for the microarray studies [21], [22]. The PCR protocol comprised of 35 cycles with 30 sec at 94°C, 30 sec at 50°C and 30 sec at 72°C, followed by detection in a 3% agarose gel; and it was supposed to yield products of ca. 120 bp. *Staphylococcus epidermidis* ATCC 35984 (RP62A), which carries *sasX* (albeit designated as *sesI*: CP000029.1; positions 1693235 to1693840; locus tag SERP1654), was used as positive control.

### Statistical Analysis

For relevant (resistance or virulence-associated) target genes, contingency tables were created, indicating whether or not (Yes/No) the gene was identified within the different types of isolates comparing BSI *vs*. carriage isolates as well as SSTI *vs*. carriage isolates. Fisher exact test for count data was then used [24] for testing the hypothesis of independence of rows and columns in the contingency table. If the Fisher test yielded a p-value below 0.05, this was regarded a significant difference in the Yes/No ratio between the diagnosis related groups. In Tables 2 and 3, significant values are shown in bold font.

**Table 2:** Resistance genes in MRSA, MSSA and in isolates from different diagnoses.

	MRSA isolates		MSSA isolates		Carriage isolates		Blood culture isolates			SSTI isolates		
	N =	% =	N =	% =	N =	% =	N =	% =	P (BSI vs. carriage)	N =	% =	P (SSTI vs. carriage)
***mecA***	97	100.0	0	0.0	17	34.0	26	34.2	1.00	33	54.1	**0.04**
**SCC** ***mec*** ** I**	0	0.0	0	0.0	0	0.0	0	0.0	1.00	0	0.0	1.00
**SCC** ***mec*** ** II**	2	2.1	0	0.0	0	0.0	0	0.0	1.00	0	0.0	1.00
**SCC** ***mec*** ** III**	22	22.7	0	0.0	0	0.0	7	9.2	**0.04**	4	6.6	0.13
**SCC** ***mec*** ** IV**	72	74.2	0	0.0	17	34.0	18	23.7	0.23	29	47.5	0.18
**SCC** ***mec*** ** V**	1	1.0	0	0.0	0	0.0	1	1.3	1.00	0	0.0	1.00
***merA/B***	0	0.0	0	0.0	0	0.0	0	0.0	1.00	0	0.0	1.00
***blaZ/I/R***	97	100.0	98	88.3	45	90.0	72	94.7	0.48	57	93.4	0.73
***erm*** **(A)**	24	24.7	4	3.6	1	2.0	10	13.2	**0.05**	4	6.6	0.38
***erm*** **(B)**	1	1.0	0	0.0	0	0.0	0	0.0	1.00	0	0.0	1.00
***erm*** **(C)**	41	42.3	18	16.2	17	34.0	16	21.1	0.15	19	31.2	0.84
***msr*** **(A)**	24	24.7	3	2.7	6	12.0	5	6.6	0.34	16	26.2	0.09
***mpbBM***	24	24.7	3	2.7	6	12.0	5	6.6	0.34	16	26.2	0.09
***aacA-aphD***	25	25.8	3	2.7	4	8.0	7	9.2	1.00	7	11.5	0.75
***aadD***	3	3.1	1	0.9	1	2.0	1	1.3	1.00	0	0.0	0.45
***aphA3***	69	71.1	15	13.5	20	40.0	21	27.6	0.18	35	57.4	0.09
***sat***	69	71.1	15	13.5	20	40.0	21	27.6	0.18	35	57.4	0.09
***dfrA***	1	1.0	1	0.9	0	0.0	1	1.3	1.00	1	1.6	1.00
***far1***	4	4.1	0	0.0	0	0.0	3	4.0	0.28	1	1.6	1.00
***fusC***	0	0.0	2	1.8	0	0.0	0	0.0	1.00	2	3.3	0.50
***mupR***	0	0.0	0	0.0	0	0.0	0	0.0	1.00	0	0.0	1.00
***tet*** **(K)**	44	45.4	25	22.5	16	32.0	21	27.6	0.69	24	39.3	0.44
***tet*** **(M)**	23	23.7	2	1.8	0	0.0	8	10.5	**0.02**	6	9.8	**0.03**
***cat***	1	1.0	1	0.9	0	0.0	1	1.3	1.00	1	1.6	1.00
***cfr***	0	0.0	0	0.0	0	0.0	0	0.0	1.00	0	0.0	1.00
***qacA***	5	5.2	0	0.0	3	6.0	0	0.0	0.06	2	3.3	0.66
***qacC***	1	1.0	1	0.9	0	0.0	0	0.0	1.00	2	3.3	0.50
***vanA***	0	0.0	0	0.0	0	0.0	0	0.0	1.00	0	0.0	1.00

**Table 3:** Virulence-associated genes in MRSA, MSSA and in isolates from different diagnoses.

	MRSA isolates		MSSA isolates		Carriage isolates		Blood culture isolates			SSTI isolates		
	N =	% =	N =	% =	N =	% =	N =	% =	P (BSI vs. carriage)	N =	% =	P (SSTI vs. carriage)
***tst1***	0	0.0	9	8.1	3	6.0	6	7.9	1.00	0	0.0	0.09
***sea***	22	22.7	6	5.4	2	4.0	11	14.5	0.08	4	6.6	0.69
***sea (N315)***	0	0.0	6	5.4	2	4.0	3	4.0	1.00	1	1.6	0.59
***seb***	0	0.0	3	2.7	0	0.0	3	4.0	0.28	0	0.0	1.00
***sec+sel***	0	0.0	17	15.3	6	12.0	10	13.2	1.00	1	1.6	**0.04**
***sed+sej+ser***	3	3.1	0	0.0	0	0.0	0	0.0	1.00	0	0.0	1.00
***see***	0	0.0	0	0.0	0	0.0	0	0.0	1.00	0	0.0	1.00
***seh***	40	41.2	17	15.3	16	32.0	15	19.7	0.14	19	31.2	1.00
***sek***	47	48.5	3	2.7	5	10.0	12	15.8	0.43	22	36.1	**0.00**
***egc***	4	4.1	72	64.9	21	42.0	37	48.7	0.47	15	24.6	0.07
***seq***	44	45.4	3	2.7	5	10.0	11	14.5	0.59	20	32.8	**0.01**
***ORF CM14***	0	0.0	13	11.7	0	0.0	9	11.8	**0.01**	4	6.6	0.13
***lukF/S-PV***	30	30.9	15	13.5	5	10.0	17	22.4	0.09	23	37.7	**0.00**
***lukD***	95	97.9	60	54.1	30	60.0	51	67.1	0.45	53	86.9	**0.00**
***lukE***	95	97.9	63	56.8	31	62.0	52	68.4	0.57	54	88.5	**0.00**
***sak***	85	87.6	98	88.3	40	80.0	72	94.7	**0.02**	52	85.3	0.61
***chp***	27	27.8	61	55.0	25	50.0	33	43.4	0.58	28	45.9	0.71
***scn***	85	87.6	102	91.9	42	84.0	73	96.1	**0.03**	53	86.9	0.79
***etA***	0	0.0	1	0.9	0	0.0	1	1.3	1.00	0	0.0	1.00
***etB***	0	0.0	2	1.8	0	0.0	2	2.6	0.52	0	0.0	1.00
***etD***	5	5.2	2	1.8	0	0.0	6	7.9	0.08	1	1.6	1.00
***edinA***	0	0.0	2	1.8	0	0.0	1	1.3	1.00	1	1.6	1.00
***edinB***	5	5.2	2	1.8	0	0.0	6	7.9	0.08	1	1.6	1.00
***edinC***	0	0.0	2	1.8	0	0.0	2	2.6	0.52	0	0.0	1.00
***ACME***	24	24.7	0	0.0	5	10.0	4	5.3	0.48	15	24.6	0.05

## Results

CC and strain affiliations are shown in Table 1. Absolute numbers and percentages of resistance markers and of virulence-associated genes in MRSA and in MSSA isolates are provided in Table 2 and 3, respectively. Full hybridisation results are provided as File S1.

### MRSA in Iaşi

The most common SCC*mec* element was SCC*mec* IV, followed by SCC*mec* III, while SCC*mec* II and SCC*mec* V were found only sporadically (Table 2). None of the SCC*mec* III elements was associated with the mercury resistance operon, contrarily to findings from other geographic settings [17], [21], [25]. All MRSA isolates also harboured the beta-lactamase operon. Other common resistance markers included *erm*(A), *erm*(C), *msr*(A)+*mph*(C), *aacA-aphD*, *aphA3+sat*, *tet*(K) and *tet*(M). The *mupR* mupirocin resistance gene, the *cfr* linezolid resistance gene and genes associated with glycopeptide resistance were not found (Table 2). Thirty MRSA isolates (31%) were positive for PVL genes. Other common virulence genes were *tst1,* enterotoxin genes *sea, seh* (associated with the CC1-MRSA-IV strain), *sek/q*, the immunevasion cluster genes (*sak, chp, scn*) and ACME which was associated with CC8-MRSA-IV (“USA300”). Exfoliative toxin genes A and B were not found; *edinB* and *etD* were restricted to CC80 isolates.

The most common strain found in Ia?i was a PVL-negative CC1-MRSA-IV ([21], [26], [27]. Apparently its prevalence increased during the collection period, with 29% of MRSA (2/7) belonging to that strain in 2008; 18% (3/17) in 2009; 29% (4/14) in 2010; 41% (9/22) in 2011 and 63% (19/30) in 2012. All carried the enterotoxin H gene *seh*, lacked other enterotoxin genes, PVL genes and a protease gene (*splE*) that is usually present in CC1. These isolates were positive for beta-lactamase genes, *erm*(C), *aphA3* and *sat*. The *fusC* gene (Q6GD50), as well as *ccrA/B-1,* which can commonly be found in CC1 as part of an additional SCC element, were absent from all isolates. The vast majority of isolates was positive for *tet*(K). There was minor variability affecting additional resistance markers (*aacA-aphD, erm*(B), *msr*(A), *mph*(C), *cat, qacA*) and carriage of beta-haemolysin-integrating phages (*sak* and *scn,* or neither).

Another common strain was PVL/ACME-positive CC8-MRSA-IV (“USA300”, 24 isolates). In addition, there was one closely related isolate that lacked ACME.

The third most common strain was ST239-like MRSA-III (“Vienna/Hungarian/Brazilian Clone”, 22 isolates). As previously described [21], these MRSA clearly can be differentiated by array hybridisation from the parental clonal complex CC8; but single locus variants ST239, ST240 and ST241 cannot be differentiated from each other. Hence, these isolates will be named “ST239-like MRSA-III” herein. These isolates harboured, beside SCC*mec* III markers such as *ccrA/B3*, also *ccrC* but lacked a mercury resistance element that is common in ST239-like MRSA-III [21], [28]. Isolates were also screened by PCR for *sasX*, a virulence/adhesion factor recently discovered in that strain [23]. All 22 isolates were negative.

Five isolates belonged to PVL-positive CC80-MRSA-IV (“European CA-MRSA Clone”), all isolated in 2011. Two isolates were identified as CC5-MRSA-II (“Rhine-Hesse EMRSA/New York Japan”). Other sporadic strains included CC22-MRSA-IV (“UK-EMRSA-15/Barnim EMRSA”), CC398-MRSA-V and CC5-MRSA-IV (“Paediatric clone”).

### Carriage Isolates

Seventeen, *i.e*., about one-third of screening isolates, were MRSA. Twelve of these 17 isolates were assigned to the endemic strain CC1-MRSA-IV while five belonged to PVL/ACME-positive CC8-MRSA-IV (“USA300”). Apart from the “USA300” isolates, there were no other PVL-positives in the carriage group. The remaining, PVL-negative and methicillin-susceptible isolates belonged to different clonal complexes (Table 1) with CC22 and CC45 being the most frequent lineages.

With regard to toxin carriage (Table 3), *tst1* (6%) was less common in this group than in the bacteraemia isolates while PVL (10%), *sak* (80%), the *egc* cluster (42%) and *sek/q* were less common (10%) than in both clinical groups. The *seh* gene was more common (32%) in carriage isolates than in blood culture isolates.

### Blood Culture Isolates

Among blood culture isolates, the MRSA rate was 34% with locally epidemic CC1-MRSA-IV, “USA300” and ST239-like MRSA-III (“Vienna/Hungarian/Brazilian Clone”) being the dominant strains. Four isolates of CC80-MRSA-IV [PVL+] (“European CA-MRSA Clone”) were observed, all isolated in 2011, but there was no obvious epidemiological connection. Single isolates of the “livestock-associated” MRSA strain CC398-MRSA-V and of CC22-MRSA-IV (“UK-EMRSA-15/Barnim EMRSA”) were both found in 2010. MSSA isolates belonged to various lineages with CC22 and CC45-MSSA being the most common ones.

PVL genes were commonly detected among blood culture group isolates (22%) with CC121-MSSA [PVL+] being the single most common lineage. With regard to other virulence factors, the *egc* cluster genes (enterotoxin genes/enterotoxin like genes *seg, sei, selm, seln, selo, selu*) were common being associated with CC22, CC45 and CC121. The enterotoxin H gene *seh* was strictly associated with CC1 affiliation. Enterotoxin genes *sec* plus *sel* were found in 10 isolates (13%); *tst1* (encoding toxic shock syndrome toxin) in 6 isolates (8%). The staphylokinase gene *sak* was present in 72 blood culture isolates (95%).

### Skin and Soft Tissue Infections

Sixty-one SSTI isolates were included. More than half of them were MRSA (54%). The most common strain among SSTI isolates was the PVL/ACME-positive CC8-MRSA-IV (“USA300”) followed by the PVL-negative CC1-MRSA-IV strain. PVL-positive, methicillin-susceptible lineages included CC8- and CC121-MSSA. PVL-negative MSSA belonged to several different lineages (Table 1). PVL was detected in more than one-third (37%) of the sixty-one SSTI isolates. Other common genes for virulence factors were genes were *sek/q*, *seh* (strictly associated with CC1), *egc*-genes, *lukD/E* and *sak* (Table 3).

## Discussion

A high prevalence of MRSA was noted with roughly one-third of bloodstream isolates being MRSA. This is in accordance with previous studies from Romania (see Introduction). Three strains appeared to be particularly abundant among tested isolates.

The most common MRSA strain (with a total of 40 out of 97 MRSA isolates) was CC1-MRSA-IV. CC1-MRSA-IV was described previously in Australia (“West Australian MRSA 1, 45 and 57”, [27], [29], [30]) and the Middle East [21], [31], but these isolates [21] usually differed from the Ia?i isolates in the absence of the *aphA3/sat* genes as well as in the presence of additional toxin genes (*sea, sek, seq*) and *splE*. Interestingly, a number of methicillin-susceptible, PVL-negative CC1 isolates (12 out of 17 CC1-MSSA) were identical to this MRSA strain with regard to several features including the absence of *splE*, the absence of other enterotoxin genes besides *seh,* as well as to the simultaneous presence of *aphA3, sat, erm*(C) and *tet*(K). While it cannot be ruled out that these isolates are spontaneous *mecA* deletion mutants of CC1-MRSA-IV, this strain might indeed have emerged locally. CC1 MRSA have already been found in Romania before [8], although in a different part of the country (Braşov; Transylvania region).

PVL/ACME-positive CC8-MRSA-IV (“USA300”) was also common among Romanian isolates. This strain is known to be abundant in the United States and it has also been detected in Canada, Australia, and, to a lesser extent, in Western Europe. It possibly emerged in the Caribbean [32] where PVL-positive CC8-MSSA are common [33], [34]. CC8-MRSA-IV (“USA300”) was already found in neighbouring Bulgaria [20] and there was also previous evidence for its presence in Romania [http://registration.akm.ch/ einsicht.php?XNABSTRACT_ID = 61874&XNSPRACHE_ID = 2&XNKONGRESS_ID = 73&XNMASKEN_ID = 900].

An importation from the United States or Western Europe appears to be likely, given that considerable numbers of Romanian citizen have travelled to and worked in these countries in recent times (for 2007, it was estimated that 3.4 million Romanians, out of a total population of about 22 million, worked abroad; http://focus-migration.hwwi.de/ Romania.2515.0.html?&L = 1; retrieved January 2014). It is noteworthy that the ACME-positive variant of this strain predominated, which is also the predominant variant in the United States and other English speaking countries. The ACME-negative, PVL-positive CC8-MRSA-IV strain that is associated with Spain and Latin American countries was found once only.

A third common MRSA strain was ST239-like MRSA-III, a rather ancient, hospital-associated clone that has essentially been found worldwide [21], [35] including Romania [8], [13], [14], [15] and other South-Eastern European countries, such as neighbouring Hungary (although there it has been replaced by CC5 MRSA strains [16]). There are different variants of this strain [21], [28], [36], differing in the carriage of a mercury resistance operon, of *ccrC, sasX*, enterotoxin genes, tetracycline and aminoglycoside resistance genes. Isolates from Ia?i proved to be quite homogenous suggesting a clonal epidemic spread rather than, *e.g*., multiple importations.

Other MRSA strains were PVL-positive CC80-MRSA-IV. This is a community-associated (CA-) MRSA known to occur commonly in Greece [37], [38], in Maghreb (North African; [39], [40]) and Arab countries [17], [31] as well as sporadically in Western Europe [18]. It also been observed in Romania in recent years [8], [13], [15] as well as in neighbouring Serbia [19] and Bulgaria [20]. All isolates from the present study were found in 2011. Four out of five of them were related to diagnoses suggesting a hospital-acquired infection (blood cultures of patients undergoing medical procedures or care/treatment for chronic diseases such as chronic hepatitis, cirrhosis or cancer). This could indicate a largely hospital-based transmission upon accidental importation. CC5-MRSA-II (“Rhine-Hesse EMRSA/New York Japan”), CC5-MRSA-IV (“Paediatric clone”) and CC22-MRSA IV (“UK-EMRSA-15/Barnim EMRSA”) were found sporadically only, although these strains are common in Central and Western Europe where a large number of Romanian nationals works. MRSA with *spa* type t015 and SCC*mec* IV elements, thus likely to correspond to “UK-EMRSA-15/Barnim EMRSA”, have been observed in Romania before [15]. The livestock-associated CC398-MRSA-V strain was found once in a blood culture from a patient from an urban area who had chronic obstructive lung disease. There are no systematic studies available on CC398-MRSA-V in Romania, neither on humans nor on livestock, although a MRSA with a *spa* type associated with the CC398 lineage (t034) has been found there before [15]. CC30- and CC45-MRSA that have been observed in other Romanian studies [8], [15] were not identified among the isolate collection described herein.

PVL-positive MSSA belonged to CC5, CC8, CC45 and CC121. In previous studies from different geographic regions, PVL-positive CC5 isolates were not uncommon while CC45 harbouring PVL genes were distinctly rare [34]. PVL-positive CC8 isolates were commonly found in the Caribbean, whereas they appear to be rather rare in Western Central Europe [33], [34]. CC121 is a pandemic lineage, and PVL-positives have been commonly described from very diverse settings [21], [41], [42], [43], [44], [45], [46]. Finally, PVL-negative MSSA were assigned to a wide variety of clonal complexes. The most common lineages were CC1 (17 isolates in total, including a possible precursor to a locally common MRSA, see above), CC22 and CC45. These lineages were also found in studies from other European countries [47], [48], [49], [50], [51] although their abundances might differ locally.

Virulence-associated genes that were more common in bloodstream isolates than in other groups were *tst1*, the *egc* locus genes, possibly *sec+sel, sak* and *scn*. The enterotoxin H gene was less common in the bloodstream group than in others. This might be attributed to different population structures within the different groups since (*seh*-positive) CC1 isolates were more common among carriage and SSTI isolates. Higher rates of *sak* in invasive infections were already reported previously [48]. A similar high rate of *tst1* positives like in this study has been described for bloodstream isolates from Norway (16%, [47]), while the prevalence of *tst1* genes in healthy carriers from Germany was much higher (15%) than observed in carriage isolates from this study. For *egc,* the Norwegian study [47] also reported a high prevalence in bacteraemia isolates (58%) while the rate in German carriers [49] was reported to be similar (47%) to the one reported in the present study. Contrarily, a Dutch study reported *egc* to be more common in carriers than in sepsis patients [52]. The contradicting observations for *tst1* and *egc* could indicate that the presence or absence of individual exotoxin genes is only secondary to other factors, and that the different prevalences do not reflect a role in pathogenesis, but just regional differences in population structure and the presence of outbreak strains on certain wards. This issue warrants further, more systematic and larger multicentre studies. Leukocidin genes were more common in the SSTI group than in other groups. This included *lukD/E* as well as PVL genes. The latter were present in more than one third of SSTI isolates. Surprisingly, they also were detected in about one-fifth of blood culture isolates although PVL apparently plays no role in the pathogenesis of bloodstream infections [53]. Most PVL-positive bloodstream isolates were *mecA*-negative *S. aureus*, with CC121-MSSA being the most common lineage. However, eight PVL-positive MRSA isolates originated from blood cultures. They belonged to CC80-MRSA-IV and “USA300” possibly indicating an invasion of these normally “community-associated” strains into hospital settings.

Our study provides a snapshot of *S. aureus*/MRSA epidemiology in Romania. Although it focuses on one city only, it confirms a high burden of MRSA and PVL on Romanian healthcare settings that might also become relevant for Western Europe with increasing travel activities.

## Supporting Information

File S1 **Clinical data and full hybridisation profiles.**
(PDF)
