# Supplementary material for: Molecular Typing of MRSA and of Clinical Staphylococcus aureus Isolates from Iaşi, Romania
Source: PLoS One. 2014 May 20;9(5):e97833. doi: 10.1371/journal.pone.0097833 (PMC4028265; doi:10.1371/journal.pone.0097833)
Supplement: File S1 — Clinical data and full hybridisation profiles. (PDF) [file pone.0097833.s001.pdf]

Isolate	Group	Sample type / Diagnosis	Patient Characteristics	Year	CC	Strain	SPECIES MARKER							REGULATORY GENES															REGULATORY GENES			
							Domain 1 of 23S-rRNA	glyceraldehyde 3-phosphate dehydrogenase, locus 1	katalase A	coagulase	thermostable extracellular nuclease	staphylococcal protein A	IgG-binding protein	staphylococcal accessory regulator A	histidine protein kinase, sae locus	sensor protein	accessory gene regulator allele I				accessory gene regulator allele II				accessory gene regulator allele III				accessory gene regulator allele IV			haemolysin delta
							rrnD1 (S. aureus)	gapA	katA	coA	nuc1	spa	sbi	sarA	saeS	vraS	agrI (total)	agrB-I	agrC-I	agrD-I	agrII (total)	agrB-II	agrC-II	agrD-II	agrIII (total)	agrB-III	agrC-III	agrD-III	agrIV (total)	agrB-IV	agrC-IV	hld
Iasi-01-4	BSI	blood culture, Sepsis	35 years old female patient from urban area	III 2010	CC1	CC1-MSSA	POS	POS	POS	POS	POS	POS	POS	POS	POS	POS	NEG	NEG	NEG	NEG	NEG	NEG	NEG	NEG	POS	POS	POS	POS	NEG	NEG	NEG	POS
Iasi-05-32	BSI	blood culture, Sepsis	14 years old female patient from urban area	I 2010	CC1	CC1-MSSA	POS	POS	POS	POS	POS	POS	POS	POS	POS	POS	NEG	NEG	NEG	NEG	NEG	NEG	NEG	NEG	POS	POS	POS	POS	NEG	NEG	NEG	POS
Iasi-16-88	BSI	blood culture, Sepsis	75 years old male patient from urban area with diabetes, renal failure	II 2009	CC1	CC1-MSSA	POS	POS	POS	POS	POS	POS	POS	POS	POS	POS	NEG	NEG	NEG	NEG	NEG	NEG	NEG	NEG	POS	POS	POS	POS	NEG	NEG	NEG	POS
Iasi-25-141	BSI	3.blood culture (+CSF), Sepsis	67 years old female patient from rural area with diabetes, chronic C hepatitis	VI 2010	CC1	CC1-MSSA	POS	POS	POS	POS	POS	POS	POS	POS	POS	POS	NEG	NEG	NEG	NEG	NEG	NEG	NEG	NEG	POS	POS	POS	POS	NEG	NEG	NEG	POS
Iasi-32-215	BSI	2.blood culture, Sepsis	75 years old male patient from rural area with chronic hepatitis, tbc	XI 2010	CC1	CC1-MSSA	POS	POS	POS	POS	POS	POS	POS	POS	POS	POS	NEG	NEG	NEG	NEG	NEG	NEG	NEG	NEG	POS	POS	POS	POS	NEG	NEG	NEG	POS
Iasi-43-359	BSI	blood culture, Sepsis	41 years old male patient from rural area with chron. pancreatitis, hypoproteinaemia	VII 2011	CC1	CC1-MSSA	POS	POS	POS	POS	POS	POS	POS	POS	POS	POS	NEG	NEG	NEG	NEG	NEG	NEG	NEG	NEG	POS	POS	POS	POS	NEG	NEG	NEG	POS
Iasi-03-27	SSTI	pus, panaritium	13 years old female from urban area	2010	CC1	CC1-MSSA	POS	POS	POS	POS	POS	POS	POS	POS	POS	POS	NEG	NEG	NEG	NEG	NEG	NEG	NEG	NEG	POS	POS	POS	POS	NEG	NEG	NEG	POS
Iasi-13-149	SSTI	pus, Calf cellulitis	50 years old male from urban area	2010	CC1	CC1-MSSA	POS	POS	POS	POS	POS	POS	POS	POS	POS	POS	NEG	NEG	NEG	NEG	NEG	NEG	NEG	NEG	POS	POS	POS	POS	NEG	NEG	NEG	POS
Iasi-29-337	SSTI	pus, Calf cellulitis/ sepsis	82 years old female from rural area	2011	CC1	CC1-MSSA	NEG	POS	POS	POS	POS	POS	POS	POS	POS	POS	NEG	NEG	NEG	NEG	NEG	NEG	NEG	NEG	POS	POS	POS	POS	NEG	NEG	NEG	POS
Iasi-31-355	SSTI	pus, cellulitis/sepsis	61 years old male from rural area with cirrhosis, chronic alcoholism	2011	CC1	CC1-MSSA	POS	POS	POS	POS	POS	POS	POS	POS	POS	POS	NEG	NEG	NEG	NEG	NEG	NEG	NEG	NEG	POS	POS	POS	POS	NEG	NEG	NEG	POS
Iasi-38-377	SSTI	pus, abdominal wound	75 years old male	2011	CC1	CC1-MSSA	POS	POS	POS	POS	POS	POS	POS	POS	POS	POS	NEG	NEG	NEG	NEG	NEG	NEG	NEG	NEG	POS	POS	POS	POS	NEG	NEG	NEG	POS
Iasi-41-388	SSTI	pus, inguinal pus	female	2012	CC1	CC1-MSSA	POS	POS	POS	POS	POS	POS	POS	POS	POS	POS	NEG	NEG	NEG	NEG	NEG	NEG	NEG	NEG	POS	POS	POS	POS	NEG	NEG	NEG	POS
Iasi-46-417	SSTI	pus, leg wound	84 years old female	2012	CC1	CC1-MSSA	POS	POS	POS	POS	POS	POS	POS	POS	POS	POS	NEG	NEG	NEG	NEG	NEG	NEG	NEG	NEG	POS	POS	POS	POS	NEG	NEG	NEG	POS
Iasi-02-S9	Carrier	Carriage	35 years old male	2012	CC1	CC1-MSSA	POS	POS	POS	POS	POS	POS	POS	POS	POS	POS	NEG	NEG	NEG	NEG	NEG	NEG	NEG	NEG	POS	POS	POS	POS	NEG	NEG	NEG	POS
Iasi-17-S33	Carrier	Carriage	40 years old male	2012	CC1	CC1-MSSA	POS	POS	POS	POS	POS	POS	POS	POS	POS	POS	NEG	NEG	NEG	NEG	NEG	NEG	NEG	NEG	POS	POS	POS	POS	NEG	NEG	NEG	POS
Iasi-30-P17	Carrier	Carriage	43 years old female from rural area with pultaceous angina, Candida stomatitis	2012	CC1	CC1-MSSA	POS	POS	POS	POS	POS	POS	POS	POS	POS	POS	NEG	NEG	NEG	NEG	NEG	NEG	NEG	NEG	POS	POS	POS	POS	NEG	NEG	NEG	POS
Iasi-39-P28	Carrier	Carriage			CC1	CC1-MSSA	POS	POS	POS	POS	POS	POS	POS	POS	POS	POS	NEG	NEG	NEG	NEG	NEG	NEG	NEG	NEG	POS	POS	POS	POS	NEG	NEG	NEG	POS
Iasi-04-26	BSI	2.blood culture, infectious endocarditis	29 years old female patient from rural area	II 2010	CC1	CC1-MRSA-IV, WA MRSA-1/57	POS	POS	POS	POS	POS	POS	POS	POS	POS	POS	NEG	NEG	NEG	NEG	NEG	NEG	NEG	NEG	POS	POS	POS	POS	NEG	NEG	NEG	POS
Iasi-09-72	BSI	blood culture, Right lung tumor	77 years old male patient from urban area with tumor, diabetes, chronic hepatitis	VIII 2009	CC1	CC1-MRSA-IV, WA MRSA-1/57	POS	POS	POS	POS	POS	POS	POS	POS	POS	POS	NEG	NEG	NEG	NEG	NEG	NEG	NEG	NEG	POS	POS	POS	POS	NEG	NEG	NEG	POS
Iasi-11-131	BSI	Blood culture, Sepsis	53 years old Female from urban area	2008	CC1	CC1-MRSA-IV, WA MRSA-1/57	POS	POS	POS	POS	POS	POS	POS	POS	POS	POS	NEG	NEG	NEG	NEG	NEG	NEG	NEG	NEG	POS	POS	POS	POS	NEG	NEG	NEG	POS
Iasi-14-143	BSI	Blood culture, CSF, Sepsis	54 years old Female from rural area with chronic hepatitis	2010	CC1	CC1-MRSA-IV, WA MRSA-1/57	POS	POS	POS	POS	POS	POS	POS	POS	POS	POS	NEG	NEG	NEG	NEG	NEG	NEG	NEG	NEG	POS	POS	POS	POS	NEG	NEG	NEG	POS
Iasi-18-192	BSI	2. Blood culture, Sepsis	36 years old Female from urban area	2010	CC1	CC1-MRSA-IV, WA MRSA-1/57	POS	POS	POS	POS	POS	POS	POS	POS	POS	POS	NEG	NEG	NEG	NEG	NEG	NEG	NEG	NEG	POS	POS	POS	POS	NEG	NEG	NEG	POS
Iasi-23-253	BSI	Blood culture, Sepsis	85 years old Male from urban area	2011	CC1	CC1-MRSA-IV, WA MRSA-1/57	POS	POS	POS	POS	POS	POS	POS	POS	POS	POS	NEG	NEG	NEG	NEG	NEG	NEG	NEG	NEG	POS	POS	POS	POS	NEG	NEG	NEG	POS
Iasi-35-322	BSI	Blood culture,	3,5 years old Male	2011	CC1	CC1-MRSA-IV, WA MRSA-1/57	POS	POS	POS	POS	POS	POS	POS	POS	POS	POS	NEG	NEG	NEG	NEG	NEG	NEG	NEG	NEG	POS	POS	POS	POS	NEG	NEG	NEG	POS
Iasi-40-380	BSI	Blood culture,	48 years old Female	2011	CC1	CC1-MRSA-IV, WA MRSA-1/57	POS	POS	POS	POS	POS	POS	POS	POS	POS	POS	NEG	NEG	NEG	NEG	NEG	NEG	NEG	NEG	POS	POS	POS	POS	NEG	NEG	NEG	POS
Iasi-46-406	BSI	2. Blood culture, meningitis	63 years old Male from urban area	2012	CC1	CC1-MRSA-IV, WA MRSA-1/57	POS	POS	POS	POS	POS	POS	POS	POS	POS	POS	NEG	NEG	NEG	NEG	NEG	NEG	NEG	NEG	POS	POS	POS	POS	NEG	NEG	NEG	POS
Iasi-02-6	SSTI	pus, Staphylococcal skin infection	31 years old male from urban area with chronic hepatitis	2010	CC1	CC1-MRSA-IV, WA MRSA-1/57	POS	POS	POS	POS	POS	POS	POS	POS	POS	POS	NEG	NEG	NEG	NEG	NEG	NEG	NEG	NEG	POS	POS	POS	POS	NEG	NEG	NEG	POS
Iasi-09-97	SSTI	pus, infected wound	67 years old female from urban area with AIDS C3	2009	CC1	CC1-MRSA-IV, WA MRSA-1/57	POS	POS	POS	POS	POS	POS	POS	POS	POS	POS	NEG	NEG	NEG	NEG	NEG	NEG	NEG	NEG	POS	POS	POS	POS	NEG	NEG	NEG	POS
Iasi-19-182	SSTI	pus, Foot cellulitis	51 years old male from urban area	2008	CC1	CC1-MRSA-IV, WA MRSA-1/57	POS	POS	POS	POS	POS	POS	POS	POS	POS	POS	NEG	NEG	NEG	NEG	NEG	NEG	NEG	NEG	POS	POS	POS	POS	NEG	NEG	NEG	POS
Iasi-31-314	SSTI	pus (conjunctival exudate),		2011	CC1	CC1-MRSA-IV, WA MRSA-1/57	POS	POS	POS	POS	POS	POS	POS	POS	POS	POS	NEG	NEG	NEG	NEG	NEG	NEG	NEG	NEG	POS	POS	POS	POS	NEG	NEG	NEG	POS
Iasi-33-367	SSTI	pus, Staphylococcal skin infection	34 years old female from urban area	2011	CC1	CC1-MRSA-IV, WA MRSA-1/57	POS	POS	POS	POS	POS	POS	POS	POS	POS	POS	NEG	NEG	NEG	NEG	NEG	NEG	NEG	NEG	POS	POS	POS	POS	NEG	NEG	NEG	POS
Iasi-39-378	SSTI	pus, sacral wound	50 years old male from urban area	2011	CC1	CC1-MRSA-IV, WA MRSA-1/57	POS	POS	POS	POS	POS	POS	POS	POS	POS	POS	NEG	NEG	NEG	NEG	NEG	NEG	NEG	NEG	POS	POS	POS	POS	NEG	NEG	NEG	POS
Iasi-42-394	SSTI	pus, calf pus	74 years old female from rural area	2012	CC1	CC1-MRSA-IV, WA MRSA-1/57	POS	POS	POS	POS	POS	POS	POS	POS	POS	POS	NEG	NEG	NEG	NEG	NEG	NEG	NEG	NEG	POS	POS	POS	POS	NEG	NEG	NEG	POS
Iasi-44-409	SSTI	pus, suppuration of index finger	9 days years old male	2012	CC1	CC1-MRSA-IV, WA MRSA-1/57	POS	POS	POS	POS	POS	POS	POS	POS	POS	POS	NEG	NEG	NEG	NEG	NEG	NEG	NEG	NEG	POS	POS	POS	POS	NEG	NEG	NEG	POS
Iasi-45-405	SSTI	pus, Sepsis	69 years old Male	2012	CC1	CC1-MRSA-IV, WA MRSA-1/57	POS	POS	POS	POS	POS	POS	POS	POS	POS	POS	NEG	NEG	NEG	NEG	NEG	NEG	NEG	NEG	POS	POS	POS	POS	NEG	NEG	NEG	POS
Iasi-50-425	SSTI	pus, Sepsis	63 years old Male	2012	CC1	CC1-MRSA-IV, WA MRSA-1/57	POS	POS	POS	POS	POS	POS	POS	POS	POS	POS	NEG	NEG	NEG	NEG	NEG	NEG	NEG	NEG	POS	POS	POS	POS	NEG	NEG	NEG	POS
Iasi-50-428	SSTI	pus, buttock eschar	28 years old male	2012	CC1	CC1-MRSA-IV, WA MRSA-1/57	POS	POS	POS	POS	POS	POS	POS	POS	POS	POS	NEG	NEG	NEG	NEG	NEG	NEG	NEG	NEG	POS	POS	POS	POS	NEG	NEG	NEG	POS
Iasi-51-37	SSTI	Respiratory failure. Sacral eschar			CC1	CC1-MRSA-IV, WA MRSA-1/57	POS	POS	POS	POS	POS	POS	POS	POS	POS	POS	NEG	NEG	NEG	NEG	NEG	NEG	NEG	NEG	POS	POS	POS	POS	NEG	NEG	NEG	POS
Iasi-01-S8	Carrier	Carriage	1 year old female	2012	CC1	CC1-MRSA-IV, WA MRSA-1/57	POS	POS	POS	POS	POS	POS	POS	POS	POS	POS	NEG	NEG	NEG	NEG	NEG	NEG	NEG	NEG	POS	POS	POS	POS	NEG	NEG	NEG	POS
Iasi-08-S17	Carrier	Carriage	3 months old male	2012	CC1	CC1-MRSA-IV, WA MRSA-1/57	POS	POS	POS	POS	POS	POS	POS	POS	POS	POS	NEG	NEG	NEG	NEG	NEG	NEG	NEG	NEG	POS	POS	POS	POS	NEG	NEG	NEG	POS
Iasi-10-S23	Carrier	Carriage	30 years old female	2012	CC1	CC1-MRSA-IV, WA MRSA-1/57	POS	POS	POS	POS	POS	POS	POS	POS	POS	POS	NEG	NEG	NEG	NEG	NEG	NEG	NEG	NEG	POS	POS	POS	POS	NEG	NEG	NEG	POS
Iasi-13-S27	Carrier	Carriage	28 years old female	2012	CC1	CC1-MRSA-IV, WA MRSA-1/57	POS	POS	POS	POS	POS	POS	POS	POS	POS	POS	NEG	NEG	NEG	NEG	NEG	NEG	NEG	NEG	POS	POS	POS	POS	NEG	NEG	NEG	POS
Iasi-14-S28	Carrier	Carriage	1 month old female	2012	CC1	CC1-MRSA-IV, WA MRSA-1/57	POS	POS	POS	POS	POS	POS	POS	POS	POS	POS	NEG	NEG	NEG	NEG	NEG	NEG	NEG	NEG	POS	POS	POS	POS	NEG	NEG	NEG	POS
Iasi-15-S29	Carrier	Carriage	23 years old male	2012	CC1	CC1-MRSA-IV, WA MRSA-1/57	POS	POS	POS	POS	POS	POS	POS	POS	POS	POS	NEG	NEG	NEG	NEG	NEG	NEG	NEG	NEG	POS	POS	POS	POS	NEG	NEG	NEG	POS
Iasi-25-P10	Carrier	Carriage	5 months old male from rural area with measles complicated by pneumonia	2012	CC1	CC1-MRSA-IV, WA MRSA-1/57	POS	POS	POS	POS	POS	POS	POS	POS	POS	POS	NEG	NEG	NEG	NEG	NEG	NEG	NEG	NEG	POS	POS	POS	POS	NEG	NEG	NEG	POS
Iasi-26-P11	Carrier	Carriage	3 years old male from urban area with gastroenteritis, Candida stomatitis	2012	CC1	CC1-MRSA-IV, WA MRSA-1/57	POS	POS	POS	POS	POS	POS	POS	POS	POS	POS	NEG	NEG	NEG	NEG	NEG	NEG	NEG	NEG	POS	POS	POS	POS	NEG	NEG	NEG	POS
Iasi-32-P19	Carrier	Carriage	5 months old male from rural area with herpetic stomatitis	2012	CC1	CC1-MRSA-IV, WA MRSA-1/57	POS	POS	POS	POS	POS	POS	POS	POS	POS	POS	NEG	NEG	NEG	NEG	NEG	NEG	NEG	NEG	POS	POS	POS	POS	NEG	NEG	NEG	POS
Iasi-33-P22	Carrier	Carriage			CC1	CC1-MRSA-IV, WA MRSA-1/57	POS	POS	POS	POS	POS	POS	POS	POS	POS	POS	NEG	NEG	NEG	NEG	NEG	NEG	NEG	NEG	POS	POS	POS	POS	NEG	NEG	NEG	POS
Iasi-41-P44	Carrier	Carriage	10 years old male from urban area with erythemato-pultaceous angina	2012	CC1	CC1-MRSA-IV, WA MRSA-1/57	POS	POS	POS	POS	POS	POS	POS	POS	POS	POS	NEG	NEG	NEG	NEG	NEG	NEG	NEG	NEG	POS	POS	POS	POS	NEG	NEG	NEG	POS
Iasi-45-G515	Carrier	Carriage	46 years old male from urban area with psychiatric disabilities	2012	CC1	CC1-MRSA-IV, WA MRSA-1/57	POS	POS	POS	POS	POS	POS	POS	POS	POS	POS	NEG	NEG	NEG	NEG	NEG	NEG	NEG	NEG	POS	POS	POS	POS	NEG	NEG	NEG	POS
Iasi-02-39	misc. MRSA	sputum, Sepsis	73 years old Male from rural area with tbc, hipoanabolic syndrome	2009	CC1	CC1-MRSA-IV, WA MRSA-1/57	POS	POS	POS	POS	POS	POS	POS	POS	POS	POS	NEG	NEG	NEG	NEG	NEG	NEG	NEG	NEG	POS	POS	POS	POS	NEG	NEG	NEG	POS
Iasi-24-263	misc. MRSA	CSF, Sepsis	44 years old Male from urban area with diabetes	2011	CC1	CC1-MRSA-IV, WA MRSA-1/57	POS	POS	POS	POS	POS	POS	POS	POS	POS	POS	NEG	NEG	NEG	NEG	NEG	NEG	NEG	NEG	POS	POS	POS	POS	NEG	NEG	NEG	POS
Iasi-26-285	misc. MRSA	sputum, Interstitial pneumonia	16 years old Male from urban area	2011	CC1	CC1-MRSA-IV, WA MRSA-1/57	POS	POS	POS	POS	POS	POS	POS	POS	POS	POS	NEG	NEG	NEG	NEG	NEG	NEG	NEG	NEG	POS	POS	POS	POS	NEG	NEG	NEG	POS
Iasi-30-306	misc. MRSA	sputum, Sepsis. lobar pneumonia	70 years old Male from rural area with hepatocarcim, chronic hepatitis	2011	CC1	CC1-MRSA-IV, WA MRSA-1/57	POS	POS	POS	POS	POS	POS	POS	POS	POS	POS	NEG	NEG	NEG	NEG	NEG	NEG	NEG	NEG	POS	POS	POS	POS	NEG	NEG	NEG	POS
Iasi-41-390	misc. MRSA	sputum,	56 years old Male	2012	CC1	CC1-MRSA-IV, WA MRSA-1/57	POS	POS	POS	POS	POS	POS	POS	POS	POS	POS	NEG	NEG	NEG	NEG	NEG	NEG	NEG	NEG	POS	POS	POS	POS	NEG	NEG	NEG	POS
Iasi-47-407	misc. MRSA	CSF, Meningitis	3 years old Female from rural area	2012	CC1	CC1-MRSA-IV, WA MRSA-1/57	POS	POS	POS	POS	POS	POS	POS	POS	POS	POS	NEG	NEG	NEG	NEG	NEG	NEG	NEG	NEG	POS	POS	POS	POS	NEG	NEG	NEG	POS
Iasi-55-408CP	misc. MRSA				CC1	CC1-MRSA-IV, WA MRSA-1/57	POS	POS	POS	POS	POS	POS	POS	POS	POS	POS	NEG	NEG	NEG	NEG	NEG	NEG	NEG	NEG	POS	POS	POS	POS	NEG	NEG	NEG	POS
Iasi-15-151	BSI	blood culture, Sepsis	74 years old Male from urban area with diabetes, BPOC - chronic obstructive lung disease	2010	CC5	CC5-MSSA	POS	POS	POS	POS	POS	POS	POS	POS	POS	POS	NEG	NEG	NEG	NEG	POS	POS	POS	POS	NEG	NEG	NEG	NEG	NEG	NEG	NEG	POS
Iasi-46-386	BSI	blood culture, Sepsis	84 years old female patient from urban area	I 2012	CC5	CC5-MSSA	POS	POS	POS	POS	POS	POS	POS	POS	POS	POS	NEG	NEG	NEG	NEG	POS	POS	POS	POS	NEG	NEG	NEG	NEG	NEG	NEG	NEG	POS
Iasi-21-195	SSTI	pus, Staph and Strep skin infection	9 years old male from rural area	2008	CC5	CC5-MSSA	POS	POS	POS	POS	POS	POS	POS	POS	POS	POS	NEG	NEG	NEG	NEG	POS	POS	POS	POS	NEG	NEG	NEG	NEG	NEG	NEG	NEG	POS
Iasi-37-375	SSTI	pus, sepsis/leg wound	female	2011	CC5	CC5-MSSA	POS	POS	POS	POS	POS	POS	POS	POS	POS	POS	NEG	NEG	NEG	NEG	POS	POS	POS	POS	NEG	NEG	NEG	NEG	NEG	NEG	NEG	POS
Iasi-45-415	SSTI	pus, Calf cellulitis	75 years old male	2012	CC5	CC5-MSSA	POS	POS	POS	POS	POS	POS	POS	POS	POS	POS	NEG	NEG	NEG	NEG	POS	POS	POS	POS	NEG	NEG	NEG	NEG	NEG	NEG	NEG	POS
Iasi-09-S19	Carrier	Carriage	35 years old male	2012	CC5	CC5-MSSA	POS	POS	POS	POS	POS	POS	POS	POS	POS	POS	NEG	NEG	NEG	NEG	POS	POS	POS	POS	NEG	NEG	NEG	NEG	NEG	NEG	NEG	POS
Iasi-53-434	BSI	blood culture, Sepsis	65 years old male patient from urban area with coronary heart disease, cardiac arrhythmia, renal failure, thrombophlebitis, leg cellulitis	VI 2012	CC5	CC5-MSSA [PVL+]	POS	POS	POS	POS	POS	POS	POS	POS	POS	POS	NEG	NEG	NEG	NEG	POS	POS	POS	POS	NEG	NEG	NEG	NEG	NEG	NEG	NEG	POS
Iasi-03-40	misc. MRSA	sputum, Flu	43 years old Female from urban area	2009	CC5	ST5/ST225-MRSA-II, Rhine-Hesse EMRSA	POS	POS	POS	POS	POS	POS	POS	POS	POS	POS	NEG	NEG	NEG	NEG	POS	POS	POS	POS	NEG	NEG	NEG	NEG	NEG	NEG	NEG	POS
Iasi-04-43	misc. MRSA	sputum, Interstitial pneumonia	43 years old Female from urban area	2009	CC5	ST5/ST225-MRSA-II, Rhine-Hesse EMRSA	POS	POS	POS	POS	POS	POS	POS	POS	POS	POS	NEG	NEG	NEG	NEG	POS	POS	POS	POS	NEG	NEG	NEG	NEG	NEG	NEG	NEG	POS
Iasi-01-20	misc. MRSA				CC5	CC5-MRSA-IV	POS	POS	POS	POS	POS	POS	POS	POS	POS	POS	NEG	NEG	NEG	NEG	POS	POS	POS	POS	NEG	NEG	NEG	NEG	NEG	NEG	NEG	POS
Iasi-35-228	BSI	2.blood culture (+CSF), Sepsis	76 years old female patient from urban area with diabetes	X 2010	CC6	CC6-MSSA	POS	POS	POS	POS	POS	POS	POS	POS	POS	POS	POS	POS	POS	POS	NEG	NEG	NEG	NEG	NEG	NEG	NEG	NEG	AMB	POS	NEG	POS
Iasi-38-293	BSI	blood culture, Sepsis. Meningitis	46 years old female patient from urban area	IV 2011	CC7	CC7-MSSA	POS	POS	POS	POS	POS	POS	POS	POS	POS	POS	POS	POS	POS	POS	NEG	NEG	NEG	NEG	NEG	NEG	NEG	NEG	POS	POS	NEG	POS
Iasi-31-P18	Carrier	Carriage	18 years old female from urban area with viral exanthem	2012	CC7	CC7-MSSA	POS	POS	POS	POS	POS	POS	POS	POS	POS	POS	POS	POS	POS	POS	NEG	NEG	NEG	NEG	NEG	NEG	NEG	NEG	AMB	POS	NEG	POS
Iasi-40-P42	Carrier	Carriage	23 years old male from rural area with rubella	2012	CC7	CC7-MSSA	POS	POS	POS	POS	POS	POS	POS	POS	POS	POS	POS	POS	POS	POS	NEG	NEG	NEG	NEG	NEG	NEG	NEG	NEG	POS	POS	NEG	POS
Iasi-40-303	BSI	2.blood culture, Sepsis	73 years old male patient from rural area	IV 2011	CC8	CC8-MSSA	POS	POS	POS	POS	POS	POS	POS	POS	POS	POS	POS	POS	POS	POS	NEG	NEG	NEG	NEG	NEG	NEG	NEG	NEG	AMB	POS	NEG	POS
Iasi-48-422	SSTI	pus, infected wound	female	2012	CC8	CC8-MSSA	POS	POS	POS	POS	POS	POS	POS	POS	POS	POS	POS	POS	POS	POS	NEG	NEG	NEG	NEG	NEG	NEG	NEG	NEG	AMB	POS	NEG	POS
Iasi-54-435	BSI	blood culture, Sepsis	56 years old male patient from rural area	VI 2012	CC8	CC8-MSSA [PVL+]	POS	POS	POS	POS	POS	POS	POS	POS	POS	POS	POS	POS	POS	POS	NEG	NEG	NEG	NEG	NEG	NEG	NEG	NEG	POS	POS	NEG	POS
Iasi-16-161	SSTI	pus, Calf cellulitis	42 years old male from urban area	2010	CC8	CC8-MSSA [PVL+]	POS	POS	POS	POS	POS	POS	POS	POS	POS	POS	POS	POS	POS	POS	NEG	NEG	NEG	NEG	NEG	NEG	NEG	NEG	AMB	POS	NEG	POS
Iasi-27-221	SSTI	pus, Staphylococcal skin infection	35 years old male from urban area	2010	CC8	CC8-MSSA [PVL+]	POS	POS	POS	POS	POS	POS	POS	POS	POS	POS	POS	POS	POS	POS	NEG	NEG	NEG	NEG	NEG	NEG	NEG	NEG	AMB	POS	NEG	POS
Iasi-09-85	BSI	blood culture, Sepsis	66 years old Female from urban area with liver cirrhosis	2009	CC8	ST8-MRSA-IV [PVL+/ACME+], USA300	POS	POS	POS	POS	POS	POS	POS	POS	POS	POS	POS	POS	POS	POS	NEG	NEG	NEG	NEG	NEG	NEG	NEG	NEG	NEG	AMB	NEG	POS
Iasi-17-189	BSI	blood culture,	74 years old Male	2008	CC8	ST8-MRSA-IV [PVL+/ACME+], USA300	POS	POS	POS	POS	POS	POS	POS	POS	POS	POS	POS	POS	POS	POS	NEG	NEG	NEG	NEG	NEG	NEG	NEG	NEG	NEG	POS	NEG	POS
Iasi-20-200	BSI	2. blood culture, Sepsis	16 years old Male from rural area	2008	CC8	ST8-MRSA-IV [PVL+/ACME+], USA300	POS	POS	POS	POS	POS	POS	POS	POS	POS	POS	POS	POS	POS	POS	NEG	NEG	NEG	NEG	NEG	NEG	NEG	NEG	NEG	AMB	NEG	POS
Iasi-34-321	BSI	blood culture, Sepsis	52 years old Female from rural area	2011	CC8	ST8-MRSA-IV [PVL+/ACME+], USA300	POS	POS	POS	POS	POS	POS	POS	POS	POS	POS	POS	POS	POS	POS	NEG	NEG	NEG	NEG	NEG	NEG	NEG	NEG	AMB	POS	NEG	POS
Iasi-04-57	SSTI	pus, Staphylococcal skin infection	23 years old female from rural area withhipoanabolic syndrome	2009	CC8	ST8-MRSA-IV [PVL+/ACME+], USA300	POS	POS	POS	POS	POS	POS	POS	POS	POS	POS	POS	POS	POS	POS	NEG	NEG	NEG	NEG	NEG	NEG	NEG	NEG	AMB	POS	NEG	POS
Iasi-05-59	SSTI	pus, Staphylococcal skin infection	41 years old female from urban area	2009	CC8	ST8-MRSA-IV [PVL+/ACME+], USA300	POS	POS	POS	POS	POS	POS	POS	POS	POS	POS	POS	POS	POS	POS	NEG	NEG	NEG	NEG	NEG	NEG	NEG	NEG	AMB	POS	NEG	POS
Iasi-06-64	SSTI	pus, Staphylococcal skin infection	1 years old female from urban area	2009	CC8	ST8-MRSA-IV [PVL+/ACME+], USA300	POS	POS	POS	POS	POS	POS	POS	POS	POS	POS	POS	POS	POS	POS	NEG	NEG	NEG	NEG	NEG	NEG	NEG	NEG	AMB	POS	NEG	POS
Iasi-10-108	SSTI	pus, Bilateral mastitis	17 years old Female from rural area	2009	CC8	ST8-MRSA-IV [PVL+/ACME+], USA300	POS	POS	POS	POS	POS	POS	POS	POS	POS	POS	POS	POS	POS	POS	NEG	NEG	NEG	NEG	NEG	NEG	NEG	NEG	NEG	AMB	NEG	POS
Iasi-12-117	SSTI	pus, Staphylococcal skin infection/ nasal furuncle	22 years old male from urban area	2008	CC8	ST8-MRSA-IV [PVL+/ACME+], USA300	POS	POS	POS	POS	POS	POS	POS	POS	POS	POS	POS	POS	POS	POS	NEG	NEG	NEG	NEG	NEG	NEG	NEG	NEG	AMB	POS	NEG	POS
Iasi-12-138	SSTI	pus, Sepsis, meningitis	0,5 years old Male from rural area	2010	CC8	ST8-MRSA-IV [PVL+/ACME+], USA300	POS	POS	POS	POS	POS	POS	POS	POS	POS	POS	POS	POS	POS	POS	NEG	NEG	NEG	NEG	NEG	NEG	NEG	NEG	AMB	POS	NEG	POS
Iasi-13-140	SSTI	pus, Sepsis, meningitis	1 years old Male from urban area	2010	CC8	ST8-MRSA-IV [PVL+/ACME+], USA300	POS	POS	POS	POS	POS	POS	POS	POS	POS	POS	POS	POS	POS	POS	NEG	NEG	NEG	NEG	NEG	NEG	NEG	NEG	NEG	AMB	NEG	POS
Iasi-15-152	SSTI	pus, Calf cellulitis	29 years old female from rural area	2010	CC8	ST8-MRSA-IV [PVL+/ACME+], USA300	POS	POS	POS	POS	POS	POS	POS	POS	POS	POS	POS	POS	POS	POS	NEG	NEG	NEG	NEG	NEG	NEG	NEG	NEG	AMB	POS	NEG	POS
Iasi-17-168	SSTI	pus, staph infection of the face	13 years old male from rural area	2008	CC8	ST8-MRSA-IV [PVL+/ACME+], USA300	POS	POS	POS	POS	POS	POS	POS	POS	POS	POS	POS	POS	POS	POS	NEG	NEG	NEG	NEG	NEG	NEG	NEG	NEG	AMB	POS	NEG	POS
Iasi-20-193	SSTI	pus, pus collection (calf)/sepsis	22 years old male from urban area	2010	CC8	ST8-MRSA-IV [PVL+/ACME+], USA300	POS	POS	POS	POS	POS	POS	POS	POS	POS	POS	POS	POS	POS	POS	NEG	NEG	NEG	NEG	NEG	NEG	NEG	NEG	AMB	POS	NEG	POS
Iasi-26-218	SSTI	pus, Staphylococcal skin infection	29 years old male from urban area	2010	CC8	ST8-MRSA-IV [PVL+/ACME+], USA300	POS	POS	POS	POS	POS	POS	POS	POS	POS	POS	POS	POS	POS	POS	NEG	NEG	NEG	NEG	NEG	NEG	NEG	NEG	AMB	POS	NEG	POS
Iasi-28-335	SSTI	pus, meningitis / pustule (hallux)	49 years old female from urban area with hypochromic anemia, hipoanabolic syndrome	2011	CC8	ST8-MRSA-IV [PVL+/ACME+], USA300	NEG	POS	POS	POS	POS	POS	POS	POS	POS	POS	POS	POS	POS	POS	NEG	NEG	NEG	NEG	NEG	NEG	NEG	NEG	NEG	AMB	NEG	POS
Iasi-29-299b	SSTI	pus (drainage tube discharge),	59 years old Female from urban area with chronic hepatitis	2011	CC8	ST8-MRSA-IV [PVL+/ACME+], USA300	POS	POS	POS	POS	POS	POS	POS	POS	POS	POS	POS	POS	POS	POS	NEG	NEG	NEG	NEG	NEG	NEG	NEG	NEG	AMB	POS	NEG	POS
Iasi-32-364	SSTI	pus, furuncle/sepsis	44 years old female from urban area	2011	CC8	ST8-MRSA-IV [PVL+/ACME+], USA300	POS	POS	POS	POS	POS	POS	POS	POS	POS	POS	POS	POS	POS	POS	NEG	NEG	NEG	NEG	NEG	NEG	NEG	NEG	AMB	POS	NEG	POS
Iasi-369	SSTI	SSTI			CC8	ST8-MRSA-IV [PVL+/ACME+], USA300	POS	POS	POS	POS	POS	POS	POS	POS	POS	POS	POS	POS	POS	POS	NEG	NEG	NEG	NEG	NEG	NEG	NEG	NEG	AMB	POS	NEG	POS
Iasi-03-S11	Carrier	Carriage	31 years old male	2012	CC8	ST8-MRSA-IV [PVL+/ACME+], USA300	POS	POS	POS	POS	POS	POS	POS	POS	POS	POS	POS	POS	POS	POS	NEG	NEG	NEG	NEG	NEG	NEG	NEG	NEG	AMB	POS	NEG	POS
Iasi-19-S35	Carrier	Carriage	28 years old female	2012	CC8	ST8-MRSA-IV [PVL+/ACME+], USA300	POS	POS	POS	POS	POS	POS	POS	POS	POS	POS	POS	POS	POS	POS	NEG	NEG	NEG	NEG	NEG	NEG	NEG	NEG	POS	POS	NEG	POS
Iasi-20-P1	Carrier	Carriage	5 years old male from urban area with Scarlet fever (control)	2012	CC8	ST8-MRSA-IV [PVL+/ACME+], USA300	POS	POS	POS	POS	POS	POS	POS	POS	POS	POS	POS	POS	POS	POS	NEG	NEG	NEG	NEG	NEG	NEG	NEG	NEG	AMB	POS	NEG	POS
Iasi-35-P24	Carrier	Carriage	10 months old female from rural area with measles , acute respiratory failure, right lung pneumothorax	2012	CC8	ST8-MRSA-IV [PVL+/ACME+], USA300	POS	POS	POS	POS	POS	POS	POS	POS	POS	POS	POS	POS	POS	POS	NEG	NEG	NEG	NEG	NEG	NEG	NEG	NEG	AMB	POS	NEG	POS
Iasi-36-P25	Carrier	Carriage	5 years old female from urban area with allergodermia	2012	CC8	ST8-MRSA-IV [PVL+/ACME+], USA300	POS	POS	POS	POS	POS	POS	POS	POS	POS	POS	POS	POS	POS	POS	NEG	NEG	NEG	NEG	NEG	NEG	NEG	NEG	AMB	POS	NEG	POS
Iasi-14-150	SSTI	pus, Staphylococcal skin infection	22 years old female from urban area	2010	CC8	ST8-MRSA-IV [PVL+/ACME-]	POS	POS	POS	POS	POS	POS	POS	POS	POS	POS	POS	POS	POS	POS	NEG	NEG	NEG	NEG	NEG	NEG	NEG	NEG	AMB	POS	NEG	POS
Iasi-07-54	BSI	2. Blood culture, Sepsis	59 years old Male from urban area	2009	CC8	ST239-MRSA-III, Vienna/Hungarian/Brazilian	POS	POS	POS	POS	POS	POS	POS	POS	POS	POS	POS	POS	POS	POS	NEG	NEG	NEG	NEG	NEG	NEG	NEG	NEG	AMB	POS	NEG	POS
Iasi-16-188	BSI	Blood culture, Sepsis	56 years old Male from rural area with diabetes, chronic alcoholism	2008	CC8	ST239-MRSA-III, Vienna/Hungarian/Brazilian	POS	POS	POS	POS	POS	POS	POS	POS	POS	POS	POS	POS	POS	POS	NEG	NEG	NEG	NEG	NEG	NEG	NEG	NEG	AMB	POS	NEG	POS
Iasi-32-318	BSI	Blood culture, Sepsis	56 years old Male from urban area with malaria	2011	CC8	ST239-MRSA-III, Vienna/Hungarian/Brazilian	POS	POS	POS	POS	POS	POS	POS	POS	POS	POS	POS	POS	POS	POS	NEG	NEG	NEG	NEG	NEG	NEG	NEG	NEG	NEG	AMB	NEG	POS
Iasi-42-397	BSI	Blood culture, Sepsis	61 years old Male from urban area	2012	CC8	ST239-MRSA-III, Vienna/Hungarian/Brazilian	POS	POS	POS	POS	POS	POS	POS	POS	POS	POS	POS	POS	POS	POS	NEG	NEG	NEG	NEG	NEG	NEG	NEG	NEG	NEG	AMB	NEG	POS
Iasi-48-408	BSI	Blood culture, Meningitis	Male	2012	CC8	ST239-MRSA-III, Vienna/Hungarian/Brazilian	POS	POS	POS	POS	POS	POS	POS	POS	POS	POS	POS	POS	POS	POS	NEG	NEG	NEG	NEG	NEG	NEG	NEG	NEG	NEG	POS	NEG	POS
Iasi-49-430	BSI	2.blood culture, Sepsis	62 years old male patient from rural area	VI 2012	CC8	ST239-MRSA-III, Vienna/Hungarian/Brazilian	POS	POS	POS	POS	POS	POS	POS	POS	POS	POS	POS	POS	POS	POS	NEG	NEG	NEG	NEG	NEG	NEG	NEG	NEG	AMB	POS	NEG	POS
Iasi-52-52	BSI	Blood culture, sepsis	59 years old male from urban area	2009	CC8	ST239-MRSA-III, Vienna/Hungarian/Brazilian	POS	POS	POS	POS	POS	POS	POS	POS	POS	POS	POS	POS	POS	POS	NEG	NEG	NEG	NEG	NEG	NEG	NEG	NEG	AMB	POS	NEG	POS
Iasi-01-2	SSTI	SSTI			CC8	ST239-MRSA-III, Vienna/Hungarian/Brazilian	POS	POS	POS	POS	POS	POS	POS	POS	POS	POS	POS	POS	POS	POS	NEG	NEG	NEG	NEG	NEG	NEG	NEG	NEG	AMB	POS	NEG	POS
Iasi-10-101	SSTI	pus, infected wound	47 years old male from urban area with chronic hepatitis C	2009	CC8	ST239-MRSA-III, Vienna/Hungarian/Brazilian	POS	POS	POS	POS	POS	POS	POS	POS	POS	POS	POS	POS	POS	POS	NEG	NEG	NEG	NEG	NEG	NEG	NEG	NEG	AMB	POS	NEG	POS
Iasi-11-106	SSTI	pus (2.), tuberculous meningoencephalitis. Infected	44 years old female from rural area with tuberculous meningoencephalitis	2009	CC8	ST239-MRSA-III, Vienna/Hungarian/Brazilian	POS	POS	POS	POS	POS	POS	POS	POS	POS	POS	POS	POS	POS	POS	NEG	NEG	NEG	NEG	NEG	NEG	NEG	NEG	AMB	POS	NEG	POS
Iasi-49-414	SSTI	pus, Sepsis	56 years old Female from urban area	2012	CC8	ST239-MRSA-III, Vienna/Hungarian/Brazilian	POS	POS	POS	POS	POS	POS	POS	POS	POS	POS	POS	POS	POS	POS	NEG	NEG	NEG	NEG	NEG	NEG	NEG	NEG	AMB	POS	NEG	POS

Isolate	Group	Sample type / Diagnosis	Patient Characteristics	Year	CC	Strain	SPECIES MARKER							REGULATORY GENES															REGULATORY GENES			
							Domain 1 of 23S-rRNA	glyceraldehyde 3-phosphate dehydrogenase, locus 1	katalase A	coagulase	thermostable extracellular nuclease	staphylococcal protein A	IgG-binding protein	staphylococcal accessory regulator A	histidine protein kinase, sae locus	sensor protein	accessory gene regulator allele I				accessory gene regulator allele II				accessory gene regulator allele III				accessory gene regulator allele IV			haemolysin delta
							rrnD1 (S. aureus)	gapA	katA	coA	nuc1	spa	sbi	sarA	saeS	vraS	agrI (total)	agrB-I	agrC-I	agrD-I	agrII (total)	agrB-II	agrC-II	agrD-II	agrIII (total)	agrB-III	agrC-III	agrD-III	agrIV (total)	agrB-IV	agrC-IV	hld
Iasi-05-46	misc. MRSA	sputum, Sepsis	77 years old Male from rural area with chronic hepatitis	2009	CC8	ST239-MRSA-III, Vienna/Hungarian/Brazilian	POS	POS	POS	POS	POS	POS	POS	POS	POS	POS	POS	POS	POS	POS	NEG	NEG	NEG	NEG	NEG	NEG	NEG	NEG	AMB	POS	NEG	POS
Iasi-06-47	misc. MRSA	sputum, Sepsis	56 years old Male from rural area with chronic hepatitis	2009	CC8	ST239-MRSA-III, Vienna/Hungarian/Brazilian	POS	POS	POS	POS	POS	POS	POS	POS	POS	POS	POS	POS	POS	POS	NEG	NEG	NEG	NEG	NEG	NEG	NEG	NEG	AMB	POS	NEG	POS
Iasi-08-73	misc. MRSA	CSF, pus, Postoperative meningitis	48 years old Male from urban area with chronic renal failure, operated cerebral aneurysm	2009	CC8	ST239-MRSA-III, Vienna/Hungarian/Brazilian	POS	POS	POS	POS	POS	POS	POS	POS	POS	POS	POS	POS	POS	POS	NEG	NEG	NEG	NEG	NEG	NEG	NEG	NEG	AMB	POS	NEG	POS
Iasi-19-194	misc. MRSA	CSF, Sepsis	47 years old Male from rural area	2010	CC8	ST239-MRSA-III, Vienna/Hungarian/Brazilian	POS	POS	POS	POS	POS	POS	POS	POS	POS	POS	POS	POS	POS	POS	NEG	NEG	NEG	NEG	NEG	NEG	NEG	NEG	NEG	AMB	NEG	POS
Iasi-21-219	misc. MRSA	sputum, Sepsis	76 years old Female from urban area with diabetes	2010	CC8	ST239-MRSA-III, Vienna/Hungarian/Brazilian	POS	POS	POS	POS	POS	POS	POS	POS	POS	POS	POS	POS	POS	POS	NEG	NEG	NEG	NEG	NEG	NEG	NEG	NEG	POS	POS	NEG	POS
Iasi-25-284	misc. MRSA	Sputum, Patient with sepsis and Tbc	51 years old male from rural area	2011	CC8	ST239-MRSA-III, Vienna/Hungarian/Brazilian	POS	POS	POS	POS	POS	POS	POS	POS	POS	POS	POS	POS	POS	POS	NEG	NEG	NEG	NEG	NEG	NEG	NEG	NEG	AMB	POS	NEG	POS
Iasi-284	misc. MRSA	Sputum, Sepsis	51 years old male from rural area with TBC	2011	CC8	ST239-MRSA-III, Vienna/Hungarian/Brazilian	POS	POS	POS	POS	POS	POS	POS	POS	POS	POS	POS	POS	POS	POS	NEG	NEG	NEG	NEG	NEG	NEG	NEG	NEG	AMB	POS	NEG	POS
Iasi-36-336	misc. MRSA	sputum, Sepsis, meningitis	61 years old Male from rural area	2011	CC8	ST239-MRSA-III, Vienna/Hungarian/Brazilian	POS	POS	POS	POS	POS	POS	POS	POS	POS	POS	POS	POS	POS	POS	NEG	NEG	NEG	NEG	NEG	NEG	NEG	NEG	NEG	AMB	NEG	POS
Iasi-43-402	misc. MRSA	sputum,	62 years old Male from rural area with Listeria meningitis	2012	CC8	ST239-MRSA-III, Vienna/Hungarian/Brazilian	POS	POS	POS	POS	POS	POS	POS	POS	POS	POS	POS	POS	POS	POS	NEG	NEG	NEG	NEG	NEG	NEG	NEG	NEG	AMB	POS	NEG	POS
Iasi-44-404	misc. MRSA	sputum, Sepsis	35 years old Male	2012	CC8	ST239-MRSA-III, Vienna/Hungarian/Brazilian	POS	POS	POS	POS	POS	POS	POS	POS	POS	POS	POS	POS	POS	POS	NEG	NEG	NEG	NEG	NEG	NEG	NEG	NEG	NEG	AMB	NEG	POS
Iasi-54-430	misc. MRSA				CC8	ST239-MRSA-III, Vienna/Hungarian/Brazilian	POS	POS	POS	POS	POS	POS	POS	POS	POS	POS	POS	POS	POS	POS	NEG	NEG	NEG	NEG	NEG	NEG	NEG	NEG	POS	POS	NEG	POS
Iasi-24-216	SSTI	pus, Staphylococcal skin infection	59 years old male from urban area with lung cancer	2010	CC10	CC10-MSSA	POS	POS	POS	POS	POS	POS	POS	POS	POS	POS	NEG	NEG	NEG	NEG	POS	POS	POS	POS	NEG	NEG	NEG	NEG	NEG	NEG	NEG	POS
Iasi-05-S13	Carrier	Carriage	30 years old male	2012	CC10	CC10-MSSA	POS	POS	POS	POS	POS	POS	POS	POS	POS	POS	NEG	NEG	NEG	NEG	POS	POS	POS	POS	NEG	NEG	NEG	NEG	NEG	NEG	NEG	POS
Iasi-45-362	BSI	2.blood culture (+CSF), Sepsis. Meningitis	74 years old female patient from urban area	VIII 2011	CC15	CC15-MSSA	POS	POS	POS	POS	POS	POS	POS	POS	POS	POS	NEG	NEG	NEG	NEG	POS	POS	POS	POS	NEG	NEG	NEG	NEG	NEG	NEG	NEG	POS
Iasi-22-199	SSTI	pus, cellulitis	59 years old male from urban area with immunosuppression	2008	CC15	CC15-MSSA	POS	POS	POS	POS	POS	POS	POS	POS	POS	POS	NEG	NEG	NEG	NEG	POS	POS	POS	POS	NEG	NEG	NEG	NEG	NEG	NEG	NEG	POS
Iasi-12-S26	Carrier	Carriage	31 years old male	2012	CC15	CC15-MSSA	POS	POS	POS	POS	POS	POS	POS	POS	POS	POS	NEG	NEG	NEG	NEG	POS	POS	POS	POS	NEG	NEG	NEG	NEG	NEG	NEG	NEG	POS
Iasi-21-P2	Carrier	Carriage	14 years old female from urban area with mononucleosis	2012	CC15	CC15-MSSA	POS	POS	POS	POS	POS	POS	POS	POS	POS	POS	NEG	NEG	NEG	NEG	POS	POS	POS	POS	NEG	NEG	NEG	NEG	NEG	NEG	NEG	POS
Iasi-06-60	BSI	blood culture, Sepsis	21 years old female patient from urban area	X 2009	CC22	CC22-MSSA	POS	POS	POS	POS	POS	POS	POS	POS	POS	POS	POS	POS	POS	POS	NEG	NEG	NEG	NEG	NEG	NEG	NEG	NEG	AMB	POS	NEG	POS
Iasi-12-79	BSI	2.blood culture, Sepsis	43 years old male patient from urban area with chronic hepatitis	VI 2010	CC22	CC22-MSSA	POS	POS	POS	POS	POS	POS	POS	POS	POS	POS	POS	POS	POS	POS	NEG	NEG	NEG	NEG	NEG	NEG	NEG	NEG	AMB	POS	NEG	POS
Iasi-17-90	BSI	2.blood culture, Sepsis	59 years old female patient from urban area	II 2009	CC22	CC22-MSSA	POS	POS	POS	POS	POS	POS	POS	POS	POS	POS	POS	POS	POS	POS	NEG	NEG	NEG	NEG	NEG	NEG	NEG	NEG	NEG	POS	NEG	POS
Iasi-21-105	BSI	blood culture, Sepsis	75 years old male patient from rural area with renal failure, anemia	IV 2009	CC22	CC22-MSSA	POS	POS	POS	POS	POS	POS	POS	POS	POS	POS	POS	POS	POS	POS	NEG	NEG	NEG	NEG	NEG	NEG	NEG	NEG	AMB	POS	NEG	POS
Iasi-22-109	BSI	3.blood culture, Sepsis	41 years old female patient from rural area with renal failure, hypersplenism, central venous catheterization	V 2009	CC22	CC22-MSSA	POS	POS	POS	POS	POS	POS	POS	POS	POS	POS	POS	POS	POS	POS	NEG	NEG	NEG	NEG	NEG	NEG	NEG	NEG	AMB	POS	NEG	POS
Iasi-23-110	BSI	blood culture, Sepsis	24 years old female patient from urban area with diabetes	VI 2009	CC22	CC22-MSSA	POS	POS	POS	POS	POS	POS	POS	POS	POS	POS	POS	POS	POS	POS	NEG	NEG	NEG	NEG	NEG	NEG	NEG	NEG	AMB	POS	NEG	POS
Iasi-24-136	BSI	blood culture, Sepsis	70 years old male patient from rural area	VI 2010	CC22	CC22-MSSA	POS	POS	POS	POS	POS	POS	POS	POS	POS	POS	POS	POS	POS	POS	NEG	NEG	NEG	NEG	NEG	NEG	NEG	NEG	AMB	POS	NEG	POS
Iasi-34-224	BSI	blood culture, Sepsis	57 years old male patient from urban area with lung cancer	IX 2010	CC22	CC22-MSSA	POS	POS	POS	POS	POS	POS	POS	POS	POS	POS	POS	POS	POS	POS	NEG	NEG	NEG	NEG	NEG	NEG	NEG	NEG	AMB	POS	NEG	POS
Iasi-39-297	BSI	blood culture, Lobar pneumonia. Sepsis. infectious	74 years old male patient from rural area	IV 2011	CC22	CC22-MSSA	POS	POS	POS	POS	POS	POS	POS	POS	POS	POS	POS	POS	POS	POS	NEG	NEG	NEG	NEG	NEG	NEG	NEG	NEG	AMB	POS	NEG	POS
Iasi-50-431	BSI	2.blood culture, Sepsis	59 years old male patient from rural area with unclassified chronic hepatitis, mitral valve insufficiency, duodenal ulcer	VI 2012	CC22	CC22-MSSA	POS	POS	POS	POS	POS	POS	POS	POS	POS	POS	POS	POS	POS	POS	NEG	NEG	NEG	NEG	NEG	NEG	NEG	NEG	AMB	POS	NEG	POS
Iasi-51-432	BSI	2.blood culture, Sepsis	26 years old female patient from urban area with pelvic inflamm. disease, mitral valve prolapse, supraventr. tachycardia, psychotic disorder	VI 2012	CC22	CC22-MSSA	POS	POS	POS	POS	POS	POS	POS	POS	POS	POS	POS	POS	POS	POS	NEG	NEG	NEG	NEG	NEG	NEG	NEG	NEG	AMB	POS	NEG	POS
Iasi-08-76	SSTI	pus, Staphylococcal skin infection	56 years old male from urban area with diabetes	2009	CC22	CC22-MSSA	POS	POS	POS	POS	POS	POS	POS	POS	POS	POS	POS	POS	POS	POS	NEG	NEG	NEG	NEG	NEG	NEG	NEG	NEG	NEG	POS	NEG	POS
Iasi-30-350	SSTI	pus, Calf cellulitis	72 years old female from rural area	2011	CC22	CC22-MSSA	POS	POS	POS	POS	POS	POS	POS	POS	POS	POS	POS	POS	POS	POS	NEG	NEG	NEG	NEG	NEG	NEG	NEG	NEG	AMB	POS	NEG	POS
Iasi-33-319	SSTI	pus, Sepsis/suppuration of amputated leg	50 years old Male from urban area	2011	CC22	CC22-MSSA	POS	POS	POS	POS	POS	POS	POS	POS	POS	POS	POS	POS	AMB	POS	NEG	NEG	NEG	NEG	NEG	NEG	NEG	NEG	NEG	AMB	NEG	POS
Iasi-40-383	SSTI	pus, wound	52 years old male from rural area	2012	CC22	CC22-MSSA	POS	POS	POS	POS	POS	POS	POS	POS	POS	POS	POS	POS	POS	POS	NEG	NEG	NEG	NEG	NEG	NEG	NEG	NEG	AMB	POS	NEG	POS
Iasi-49-423	SSTI	pus, Calf cellulitis	67 years old female	2012	CC22	CC22-MSSA	POS	POS	POS	POS	POS	POS	POS	POS	POS	POS	POS	POS	POS	POS	NEG	NEG	NEG	NEG	NEG	NEG	NEG	NEG	AMB	POS	NEG	POS
Iasi-16-S31	Carrier	Carriage	5 years old female	2012	CC22	CC22-MSSA	POS	POS	POS	POS	POS	POS	POS	POS	POS	POS	POS	POS	POS	POS	NEG	NEG	NEG	NEG	NEG	NEG	NEG	NEG	AMB	POS	NEG	POS
Iasi-24-P9	Carrier	Carriage	48 years old male from rural area with erythemato-pultaceous angina	2012	CC22	CC22-MSSA	POS	POS	POS	POS	POS	POS	POS	POS	POS	POS	POS	POS	POS	POS	NEG	NEG	NEG	NEG	NEG	NEG	NEG	NEG	POS	POS	NEG	POS
Iasi-27-P12	Carrier	Carriage	6 years old male from urban area with viral pneumonia	2012	CC22	CC22-MSSA	POS	POS	POS	POS	POS	POS	POS	POS	POS	POS	POS	POS	POS	POS	NEG	NEG	NEG	NEG	NEG	NEG	NEG	NEG	AMB	POS	NEG	POS
Iasi-38-P27	Carrier	Carriage			CC22	CC22-MSSA	POS	POS	POS	POS	POS	POS	POS	POS	POS	POS	POS	POS	POS	POS	NEG	NEG	NEG	NEG	NEG	NEG	NEG	NEG	AMB	POS	NEG	POS
Iasi-42-G511	Carrier	Carriage	32 years old male from urban area with psychiatric disabilities	2012	CC22	CC22-MSSA	POS	POS	POS	POS	POS	POS	POS	POS	POS	POS	POS	POS	POS	POS	NEG	NEG	NEG	NEG	NEG	NEG	NEG	NEG	AMB	POS	NEG	POS
Iasi-46-3N	Carrier	Carriage	8 weeks old male from rural area with Protein-Calorie Malnutrition - PCM	2010	CC22	CC22-MSSA	POS	POS	POS	POS	POS	POS	POS	POS	POS	POS	POS	POS	POS	POS	NEG	NEG	NEG	NEG	NEG	NEG	NEG	NEG	POS	POS	NEG	POS
Iasi-47-42O	Carrier	Carriage	22 weeks old female from rural area with Protein-Calorie Malnutrition - PCM	2010	CC22	CC22-MSSA	POS	POS	POS	POS	POS	POS	POS	POS	POS	POS	POS	POS	POS	POS	NEG	NEG	NEG	NEG	NEG	NEG	NEG	NEG	POS	POS	NEG	POS
Iasi-48-36N	Carrier	Carriage	25 weeks old female from rural area with Protein-Calorie Malnutrition - PCM	2010	CC22	CC22-MSSA	POS	POS	POS	POS	POS	POS	POS	POS	POS	POS	POS	POS	POS	POS	NEG	NEG	NEG	NEG	NEG	NEG	NEG	NEG	POS	POS	NEG	POS
Iasi-49-T3	Carrier	Carriage	6 years old male from urban area with	2012	CC22	CC22-MSSA	POS	POS	POS	POS	POS	POS	POS	POS	POS	POS	POS	POS	POS	POS	NEG	NEG	NEG	NEG	NEG	NEG	NEG	NEG	POS	POS	NEG	POS
Iasi-50-T2	Carrier	Carriage	7 years old male from urban area with	2012	CC22	CC22-MSSA	POS	POS	POS	POS	POS	POS	POS	POS	POS	POS	POS	POS	POS	POS	NEG	NEG	NEG	NEG	NEG	NEG	NEG	NEG	AMB	POS	NEG	POS
Iasi-22-251	BSI	blood culture, Sepsis	75 years old Male from urban area	2010	CC22	CC22-MRSA-IV, UK-15/Barnim	POS	POS	POS	POS	POS	POS	POS	POS	POS	POS	POS	POS	POS	POS	NEG	NEG	NEG	NEG	NEG	NEG	NEG	NEG	AMB	POS	NEG	POS
Iasi-27-158	BSI	2.blood culture, Sepsis	69 years old male patient from urban area with chronic hepatitis, Grade III obesity	VIII 2010	CC25	CC25-MSSA	POS	POS	POS	POS	POS	POS	POS	POS	POS	POS	POS	POS	POS	POS	NEG	NEG	NEG	NEG	NEG	NEG	NEG	NEG	POS	POS	NEG	POS
Iasi-08-70	BSI	2.blood culture (+sputum), Sepsis	83 years old male patient from urban area with heart failure, pacemaker	VIII 2009	CC30	CC30-MSSA	POS	POS	POS	POS	POS	POS	POS	POS	POS	POS	NEG	NEG	NEG	NEG	NEG	NEG	NEG	NEG	POS	POS	POS	POS	NEG	NEG	NEG	POS
Iasi-18-93	BSI	blood culture, Sepsis	51 years old male patient from rural area with chronic alcoholism, renal failure	II 2009	CC30	CC30-MSSA	POS	POS	POS	POS	POS	POS	POS	POS	POS	POS	NEG	NEG	NEG	NEG	NEG	NEG	NEG	NEG	POS	POS	POS	POS	NEG	NEG	NEG	POS
Iasi-28-P14	Carrier	Carriage	32 years old female from Republic of Moldova area with erythemato-pultaceous angina	2012	CC30	CC30-MSSA	POS	POS	POS	POS	POS	POS	POS	POS	POS	POS	NEG	NEG	NEG	NEG	NEG	NEG	NEG	NEG	POS	POS	POS	POS	NEG	NEG	NEG	POS
Iasi-29-P15	Carrier	Carriage	17 years old male from rural area with rubella encephalitis	2012	CC30	CC30-MSSA	POS	POS	POS	POS	POS	POS	POS	POS	POS	POS	NEG	NEG	NEG	NEG	NEG	NEG	NEG	NEG	POS	POS	POS	POS	NEG	NEG	NEG	POS
Iasi-03-19	BSI	2.blood culture, Sepsis	64 years old male patient from urban area with Immune suppressed	II 2010	CC45	CC45-MSSA	POS	POS	POS	POS	POS	POS	POS	POS	POS	POS	POS	POS	AMB	POS	NEG	NEG	NEG	NEG	NEG	NEG	NEG	NEG	NEG	NEG	NEG	POS
Iasi-11-77	BSI	2.blood culture, intracranial hypertension, brain ab	35 years old female patient from area	VII 2009	CC45	CC45-MSSA	POS	POS	POS	POS	POS	POS	POS	POS	POS	POS	POS	POS	AMB	POS	NEG	NEG	NEG	NEG	NEG	NEG	NEG	NEG	NEG	NEG	NEG	POS
Iasi-13-82	BSI	blood culture, Sepsis	72 years old female patient from rural area	I 2009	CC45	CC45-MSSA	POS	POS	POS	POS	POS	POS	POS	POS	POS	POS	POS	POS	AMB	POS	NEG	NEG	NEG	NEG	NEG	NEG	NEG	NEG	NEG	NEG	NEG	POS
Iasi-15-86	BSI	blood culture, Sepsis	48 years old male patient from urban area with diabetes, chronic hepatitis	V 2009	CC45	CC45-MSSA	POS	POS	POS	POS	POS	POS	POS	POS	POS	POS	POS	POS	POS	POS	NEG	NEG	NEG	NEG	NEG	NEG	NEG	NEG	NEG	NEG	NEG	POS
Iasi-20-103	BSI	3.blood culture, Sepsis	55 years old male patient from rural area with chronic hepatitis, renal failure	IV 2009	CC45	CC45-MSSA	POS	POS	POS	POS	POS	POS	POS	POS	POS	POS	POS	POS	POS	POS	NEG	NEG	NEG	NEG	NEG	NEG	NEG	NEG	NEG	NEG	NEG	POS
Iasi-33-222	BSI	2.blood culture, Sepsis	77 years old male patient from rural area with multiple myeloma	IX 2010	CC45	CC45-MSSA	POS	POS	POS	POS	POS	POS	POS	POS	POS	POS	POS	POS	POS	POS	NEG	NEG	NEG	NEG	NEG	NEG	NEG	NEG	NEG	NEG	NEG	POS
Iasi-44-360	BSI	2.blood culture, Sepsis	28 years old male patient from urban area	VIII 2011	CC45	CC45-MSSA	POS	POS	POS	POS	POS	POS	POS	POS	POS	POS	POS	POS	POS	POS	NEG	NEG	NEG	NEG	NEG	NEG	NEG	NEG	NEG	NEG	NEG	POS
Iasi-47-399	BSI	blood culture, Sepsis	68 years old male patient from rural area	II 2012	CC45	CC45-MSSA	POS	POS	POS	POS	POS	POS	POS	POS	POS	POS	POS	POS	POS	POS	NEG	NEG	NEG	NEG	NEG	NEG	NEG	NEG	NEG	NEG	NEG	POS
Iasi-34-368	SSTI	pus, Sepsis/labial abscess	53 years old female from urban area withdiabetes,systemic lupus erythematosus, chronic kidney disease	2011	CC45	CC45-MSSA	POS	POS	POS	POS	POS	POS	POS	POS	POS	POS	POS	POS	POS	POS	NEG	NEG	NEG	NEG	NEG	NEG	NEG	NEG	NEG	NEG	NEG	POS
Iasi-36-371	SSTI	pus, flictena	70 years old male from rural area with fibrosis, pulmonary silicosis, hemangioma	2011	CC45	CC45-MSSA	POS	POS	POS	POS	POS	POS	POS	POS	POS	POS	POS	POS	POS	POS	NEG	NEG	NEG	NEG	NEG	NEG	NEG	NEG	NEG	NEG	NEG	POS
Iasi-04-S12	Carrier	Carriage	32 years old male	2012	CC45	CC45-MSSA	POS	POS	POS	POS	POS	POS	POS	POS	POS	POS	POS	POS	POS	POS	NEG	NEG	NEG	NEG	NEG	NEG	NEG	NEG	NEG	NEG	NEG	POS
Iasi-06-S15	Carrier	Carriage	29 years old male	2012	CC45	CC45-MSSA	POS	POS	POS	POS	POS	POS	POS	POS	POS	POS	POS	POS	POS	POS	NEG	NEG	NEG	NEG	NEG	NEG	NEG	NEG	NEG	NEG	NEG	POS
Iasi-07-S16	Carrier	Carriage	2 years old male	2012	CC45	CC45-MSSA	POS	POS	POS	POS	POS	POS	POS	POS	POS	POS	POS	POS	POS	POS	NEG	NEG	NEG	NEG	NEG	NEG	NEG	NEG	NEG	NEG	NEG	POS
Iasi-11-S24	Carrier	Carriage	8 years old male	2012	CC45	CC45-MSSA	POS	POS	POS	POS	POS	POS	POS	POS	POS	POS	POS	POS	POS	POS	NEG	NEG	NEG	NEG	NEG	NEG	NEG	NEG	NEG	NEG	NEG	POS
Iasi-22-P4	Carrier	Carriage	22 years old male from urban area with rubella	2012	CC45	CC45-MSSA	POS	POS	POS	POS	POS	POS	POS	POS	POS	POS	POS	POS	POS	POS	NEG	NEG	NEG	NEG	NEG	NEG	NEG	NEG	NEG	NEG	NEG	POS
Iasi-34-P23	Carrier	Carriage	52 years old female from urban area with chronic obstructive lung disease, chronic B hepatitis	2012	CC45	CC45-MSSA	POS	POS	POS	POS	POS	POS	POS	POS	POS	POS	POS	POS	POS	POS	NEG	NEG	NEG	NEG	NEG	NEG	NEG	NEG	NEG	NEG	NEG	POS
Iasi-37-P26	Carrier	Carriage	9 years old male from urban area with chickenpox, severe mental deficiency	2012	CC45	CC45-MSSA	POS	POS	POS	POS	POS	POS	POS	POS	POS	POS	POS	POS	POS	POS	NEG	NEG	NEG	NEG	NEG	NEG	NEG	NEG	NEG	NEG	NEG	POS
Iasi-30-196	BSI	blood culture, Severe sepsis. Bronchopneumonia. n	7 years old female patient from rural area with hypochromic anemia, thrombocytopenia, staturo-ponderal delay	VIII 2008	CC45	CC45-MSSA [PVL+]	POS	POS	POS	POS	POS	POS	POS	POS	POS	POS	POS	POS	POS	POS	NEG	NEG	NEG	NEG	NEG	AMB	NEG	NEG	NEG	NEG	NEG	POS
Iasi-27-298	BSI	Blood culture, Sepsis	65 years old Female from urban area with chronic hepatitis	2011	CC80	CC80-MRSA-IV [PVL+], European caMRSA	POS	POS	POS	POS	POS	POS	POS	POS	POS	POS	NEG	NEG	NEG	NEG	NEG	NEG	NEG	NEG	POS	POS	POS	POS	NEG	NEG	NEG	POS
Iasi-37-363	BSI	Blood culture, Sepsis	75 years old Male from urban area with sigmoid cancer,metastases	2011	CC80	CC80-MRSA-IV [PVL+], European caMRSA	POS	POS	POS	POS	POS	POS	POS	POS	POS	POS	NEG	NEG	NEG	NEG	NEG	NEG	NEG	NEG	POS	POS	POS	POS	NEG	NEG	NEG	POS
Iasi-38-372	BSI	Blood culture, Sepsis	44 years old Male from urban area with cirrhosis, chronic hepatitis	2011	CC80	CC80-MRSA-IV [PVL+], European caMRSA	POS	POS	POS	POS	POS	POS	POS	POS	POS	POS	NEG	NEG	NEG	NEG	NEG	NEG	NEG	NEG	POS	POS	POS	POS	NEG	NEG	NEG	POS
Iasi-39-374	BSI	2. Blood culture, Sepsis	44 years old Male from urban area	2011	CC80	CC80-MRSA-IV [PVL+], European caMRSA	POS	POS	POS	POS	POS	POS	POS	POS	POS	POS	NEG	NEG	NEG	NEG	NEG	NEG	NEG	NEG	POS	POS	POS	POS	NEG	NEG	NEG	POS
Iasi-28-299	SSTI	pus (conjunctival exudate), Sepsis	1 years old Female from urban area	2011	CC80	CC80-MRSA-IV [PVL+], European caMRSA	POS	POS	POS	POS	POS	POS	POS	POS	POS	POS	NEG	NEG	NEG	NEG	NEG	NEG	NEG	NEG	POS	POS	POS	POS	NEG	NEG	NEG	POS
Iasi-07-65	BSI	2.blood culture, Sepsis	71 years old male patient from urban area with renal failure	IX 2009	CC88	CC88-MSSA	POS	POS	POS	POS	POS	POS	POS	POS	POS	POS	NEG	NEG	NEG	NEG	NEG	NEG	NEG	NEG	POS	POS	POS	POS	NEG	NEG	NEG	POS
Iasi-19-99	BSI	2.blood culture, Sepsis	67 years old female patient from rural area with liver cirrhosis	III 2009	CC97	CC97-MSSA	POS	POS	POS	POS	POS	POS	POS	POS	POS	POS	POS	POS	POS	POS	NEG	NEG	NEG	NEG	NEG	NEG	NEG	NEG	NEG	AMB	NEG	POS
Iasi-25-217	SSTI	pus, sacral eschar	40 years old female from rural area with multiple sclerosis, spastic quadriplegia	2010	CC97	CC97-MSSA	POS	POS	POS	POS	POS	POS	POS	POS	POS	POS	POS	POS	POS	POS	NEG	NEG	NEG	NEG	NEG	NEG	NEG	NEG	AMB	POS	NEG	POS
Iasi-23-P5	Carrier	Carriage	63 years old male from rural area with erythematous angina	2012	CC97	CC97-MSSA	POS	POS	POS	POS	POS	POS	POS	POS	POS	POS	POS	POS	POS	POS	NEG	NEG	NEG	NEG	NEG	NEG	NEG	NEG	AMB	POS	NEG	POS
Iasi-43-G513	Carrier	Carriage	53 years old male from urban area with psychiatric disabilities	2012	CC97	CC97-MSSA	POS	POS	POS	POS	POS	POS	POS	POS	POS	POS	POS	POS	POS	POS	NEG	NEG	NEG	NEG	NEG	NEG	NEG	NEG	AMB	POS	NEG	POS
Iasi-44-G514	Carrier	Carriage	36 years old male from urban area with psychiatric disabilities	2012	CC97	CC97-MSSA	POS	POS	POS	POS	POS	POS	POS	POS	POS	POS	POS	POS	POS	POS	NEG	NEG	NEG	NEG	NEG	NEG	NEG	NEG	AMB	POS	NEG	POS
Iasi-28-172	BSI	blood culture, Sepsis	77 years old female patient from rural area with chronic pyelonephritis, heart failure, pacemaker	VII 2008	CC121	CC121-MSSA	POS	POS	POS	POS	POS	POS	POS	POS	POS	POS	NEG	POS	POS	NEG	NEG	NEG	NEG	NEG	NEG	NEG	NEG	NEG	POS	POS	POS	POS
Iasi-42-358	BSI	blood culture, secondarily infected chickenpox lesi	4 years old female patient from rural area	VII 2011	CC121	CC121-MSSA	POS	POS	POS	POS	POS	POS	POS	POS	POS	POS	NEG	POS	POS	NEG	NEG	NEG	NEG	NEG	NEG	NEG	NEG	NEG	POS	POS	POS	POS
Iasi-48-403	BSI	blood culture, Sepsis	83 years old female patient from rural area	II 2012	CC121	CC121-MSSA	POS	POS	POS	POS	POS	POS	POS	POS	POS	POS	NEG	AMB	POS	NEG	NEG	NEG	NEG	NEG	NEG	NEG	NEG	NEG	POS	POS	POS	POS
Iasi-26-153	BSI	blood culture, Sepsis	18 years old male patient from rural area with acute hepatitis (drug induced)	VIII 2010	CC121	CC121-MSSA [PVL+]	POS	POS	POS	POS	POS	POS	POS	POS	POS	POS	NEG	POS	POS	NEG	NEG	NEG	NEG	NEG	NEG	NEG	NEG	NEG	POS	POS	POS	POS
Iasi-29-190	BSI	blood culture, Sepsis	79 years old male patient from urban area with renal failure	XI 2008	CC121	CC121-MSSA [PVL+]	POS	POS	POS	POS	POS	POS	POS	POS	POS	POS	NEG	POS	POS	NEG	NEG	NEG	NEG	NEG	NEG	NEG	NEG	NEG	POS	POS	POS	POS
Iasi-31-201	BSI	blood culture, Sepsis. Meningitis	32 years old male patient from rural area with chronic hepatitis	IX 2008	CC121	CC121-MSSA [PVL+]	POS	POS	POS	POS	POS	POS	POS	POS	POS	POS	NEG	AMB	POS	NEG	NEG	NEG	NEG	NEG	NEG	NEG	NEG	NEG	POS	POS	POS	POS
Iasi-37-289	BSI	blood culture, Sepsis	78 years old female patient from rural area with chronic hepatitis, heart failure	III 2011	CC121	CC121-MSSA [PVL+]	POS	POS	POS	POS	POS	POS	POS	POS	POS	POS	NEG	POS	POS	NEG	NEG	NEG	NEG	NEG	NEG	NEG	NEG	NEG	POS	POS	POS	POS
Iasi-41-334	BSI	blood culture, Sepsis. Meningitis	48 years old female patient from urban area	VI 2011	CC121	CC121-MSSA [PVL+]	POS	POS	POS	POS	POS	POS	POS	POS	POS	POS	NEG	POS	POS	NEG	NEG	NEG	NEG	NEG	NEG	NEG	NEG	NEG	POS	POS	POS	POS
Iasi-52-433	BSI	blood culture, Sepsis	1 years old male patient from rural area with Catheter-related bacteremia (post-operatively for inguinal hernia)	VI 2012	CC121	CC121-MSSA [PVL+]	POS	POS	POS	POS	POS	POS	POS	POS	POS	POS	NEG	NEG	POS	NEG	NEG	NEG	NEG	NEG	NEG	NEG	NEG	NEG	POS	POS	POS	POS
Iasi-07-66	SSTI	pus, Staphylococcal skin infection	56 years old female from rural area with Stroke, hemiplegia	2009	CC121	CC121-MSSA [PVL+]	POS	POS	POS	POS	POS	POS	POS	POS	POS	POS	NEG	NEG	POS	NEG	NEG	NEG	NEG	NEG	NEG	NEG	NEG	NEG	POS	POS	POS	POS
Iasi-23-211	SSTI	pus, staph infection of the face. Otitis with effusion	33 years old female from urban area	2010	CC121	CC121-MSSA [PVL+]	POS	POS	POS	POS	POS	POS	POS	POS	POS	POS	NEG	AMB	POS	NEG	NEG	NEG	NEG	NEG	NEG	NEG	NEG	NEG	POS	POS	POS	POS
Iasi-43-401	SSTI	pus, occipital carbuncle	76 years old male from urban area	2012	CC121	CC121-MSSA [PVL+]	POS	POS	POS	POS	POS	POS	POS	POS	POS	POS	NEG	AMB	POS	NEG	NEG	NEG	NEG	NEG	NEG	NEG	NEG	NEG	POS	POS	POS	POS
Iasi-47-418	SSTI	pus, calf furuncle	44 years old female	2012	CC121	CC121-MSSA [PVL+]	POS	POS	POS	POS	POS	POS	POS	POS	POS	POS	NEG	AMB	POS	NEG	NEG	NEG	NEG	NEG	NEG	NEG	NEG	NEG	POS	POS	POS	POS
Iasi-18-169	SSTI	pus, Staphylococcal skin infection	55 years old female from urban area	2008	CC133	CC133-MSSA	POS	POS	POS	POS	POS	POS	POS	POS	POS	POS	POS	POS	POS	POS	NEG	NEG	NEG	NEG	NEG	NEG	NEG	NEG	AMB	POS	NEG	POS
Iasi-18-S34	Carrier	Carriage	32 years old male	2012	CC188	CC188-MSSA	POS	POS	POS	POS	POS	POS	POS	POS	POS	POS	POS	POS	POS	POS	NEG	NEG	NEG	NEG	NEG	NEG	NEG	NEG	POS	POS	NEG	POS
Iasi-36-276	BSI	2.blood culture (+CSF), Sepsis. Meningitis. septic sh	53 years old male patient from urban area	II 2011	CC398	ST291/813-MSSA	POS	POS	POS	NEG	POS	POS	POS	POS	POS	POS	POS	POS	POS	POS	NEG	NEG	NEG	NEG	NEG	NEG	NEG	NEG	NEG	NEG	NEG	POS
Iasi-151	BSI	Blood culture, sepsis	74 years old male from urban area with diabetes, BPOC - chronic obstructive lung disease, valvular prosthesis	2010	CC398	CC398-MRSA-V	POS	POS	POS	POS	POS	POS	POS	POS	POS	POS	POS	POS	POS	POS	NEG	NEG	NEG	NEG	NEG	NEG	NEG	NEG	NEG	NEG	NEG	POS
Iasi-02-11	BSI	2.blood culture, Sepsis	63 years old male patient from urban area with chronic hepatitis	V 2010	CC1021	CC1021-MSSA	POS	POS	POS	POS	POS	POS	POS	POS	POS	POS	NEG	NEG	NEG	NEG	NEG	NEG	NEG	NEG	POS	POS	POS	POS	NEG	NEG	NEG	POS

Isolate	Group	METHICILLIN RESISTANCE AND SCCmec TYPING																										RESISTANCE : PENICILLINASE			RESISTANCE : MLS-ANTIBIOTICS												RESISTANCE : AMINOGLYSOSIDES			RESISTANCE : MISCELLANEOUS GENES	
		alternate penicillin binding protein 2, defining MRSA	alternate mec allele	truncated signal transducer protein MecR1	glycerophosphoryl diester phosphodi-esterase, associated with mecA	cassette chromosome recombinase genes A/B-1		plasmin-sensitive surface protein	hypothetical protein from SCCmec elements	cassette chromosome recombinase genes A/B-2		potassium-trans-locating ATPase A, chain 2	potassium-transporting ATPase B, chain 1	potassium-trans-locating ATPase C, chain 2	sensor kinase protein	KDP operon transcrip-tional regulatory protein	methicillin-resistance regulatory protein	signal transducer protein MecR1	homolog of xylose repressor, associated with SCCmec-elements	cassette chromosome recombinase genes A/B-3		mercury resistance operon		cassette chromosome recombinase genes "ccrAA" (hypothetical) and ccrC		cassette chromosome recombinase genes A/B-4		beta-lactamase	beta lactamase repressor (inhibitor)	beta-lactamase regulatory protein	rRNA adenine N-6-methyl-transferase, erythromycin/clindamycin resistance	erythro-mycin/clindamycin resistance	erythro-mycin/clindamycin resistance	Linco-samid-Nucleo-tidyltrans-ferase	energy-dependent efflux of erythro-mycin	macrolide efflux protein A	probable lysylphos-phatidyl-glycerol synthetase	virginia-mycin A acetyltransferase	acetyl-transferase inactivating streptogramin A	ATP binding protein, streptogramin-A-resistance		virginiamycin B hydrolase	bifunctional enzyme Aac/Aph, gentamicin resistance	amino-glycoside adenyl-transferase,tobramycin resistance	3'5'-aminog-lycoside phospho-transferase, neo-/kanamycin resistance	strepto-thricine-acetyl-transferase	dihydro-folate reductase type 1
		mecA	mecC	delta_mecR	ugpQ	ccrA-1	ccrB-1	plsSCC (COL)	Q9XB68-dcs	ccrA-2	ccrB-2	kdpA-SCC	kdpB-SCC	kdpC-SCC	kdpD-SCC	kdpE-SCC	mecI	mecR	xylR	ccrA-3	ccrB-3	merA	merB	ccrAA (MRSAZH47)	ccrC (85-2082)	ccrA-4	ccrB-4	blaZ	blaI	blaR	erm(A)	erm(B)	erm(C)	lnu(A)	msr(A)	mef(A)	mph(C)	vat(A)	vat(B)	vga(A)	vga(A) (BM 3327)	vgB(A)	aacA-aphD	aadD	aphA3	sat	dfrS1
Iasi-01-4	BSI	NEG		NEG	NEG	NEG	NEG	NEG	NEG	NEG	NEG	NEG	NEG	NEG	NEG	NEG	NEG	NEG	NEG	NEG	NEG	NEG	NEG	NEG	NEG	NEG	NEG	POS	POS	POS	NEG	NEG	POS	NEG	NEG	NEG	NEG	NEG	NEG	NEG	NEG	NEG	NEG	NEG	NEG	NEG	NEG
Iasi-05-32	BSI	NEG		NEG	NEG	NEG	NEG	NEG	NEG	NEG	NEG	NEG	NEG	NEG	NEG	NEG	NEG	NEG	NEG	NEG	NEG	NEG	NEG	NEG	NEG	NEG	NEG	POS	POS	POS	NEG	NEG	POS	NEG	NEG	NEG	NEG	NEG	NEG	NEG	NEG	NEG	NEG	NEG	POS	POS	NEG
Iasi-16-88	BSI	NEG		NEG	NEG	NEG	NEG	NEG	NEG	NEG	NEG	NEG	NEG	NEG	NEG	NEG	NEG	NEG	NEG	NEG	NEG	NEG	NEG	NEG	NEG	NEG	NEG	POS	POS	POS	NEG	NEG	NEG	NEG	NEG	NEG	NEG	NEG	NEG	NEG	NEG	NEG	NEG	NEG	NEG	NEG	NEG
Iasi-25-141	BSI	NEG		NEG	NEG	NEG	NEG	NEG	NEG	NEG	NEG	NEG	NEG	NEG	NEG	NEG	NEG	NEG	NEG	NEG	NEG	NEG	NEG	NEG	NEG	NEG	NEG	POS	POS	POS	NEG	NEG	POS	NEG	NEG	NEG	NEG	NEG	NEG	AMB	NEG	NEG	NEG	NEG	POS	POS	NEG
Iasi-32-215	BSI	NEG		NEG	NEG	NEG	NEG	NEG	NEG	NEG	NEG	NEG	NEG	NEG	NEG	NEG	NEG	NEG	NEG	NEG	NEG	NEG	NEG	NEG	NEG	NEG	NEG	POS	POS	POS	NEG	NEG	POS	NEG	NEG	NEG	NEG	NEG	NEG	AMB	NEG	NEG	NEG	NEG	POS	POS	NEG
Iasi-43-359	BSI	NEG	NEG	NEG	NEG	NEG	NEG	NEG	NEG	NEG	NEG	NEG	NEG	NEG	NEG	NEG	NEG	NEG	NEG	NEG	NEG	NEG	NEG	NEG	NEG	NEG	NEG	NEG	NEG	NEG	NEG	NEG	NEG	NEG	NEG	NEG	NEG	NEG	NEG	NEG	NEG	NEG	NEG	NEG	NEG	NEG	NEG
Iasi-03-27	SSTI	NEG		NEG	NEG	NEG	NEG	NEG	NEG	NEG	NEG	NEG	NEG	NEG	NEG	NEG	NEG	NEG	NEG	NEG	NEG	NEG	NEG	NEG	NEG	NEG	NEG	POS	POS	POS	NEG	NEG	POS	NEG	NEG	NEG	NEG	NEG	NEG	NEG	NEG	NEG	NEG	NEG	POS	POS	NEG
Iasi-13-149	SSTI	NEG		NEG	NEG	NEG	NEG	NEG	NEG	NEG	NEG	NEG	NEG	NEG	NEG	NEG	NEG	NEG	NEG	NEG	NEG	NEG	NEG	NEG	NEG	NEG	NEG	POS	POS	POS	NEG	NEG	NEG	NEG	NEG	NEG	NEG	NEG	NEG	NEG	NEG	NEG	NEG	NEG	NEG	NEG	NEG
Iasi-29-337	SSTI	NEG		NEG	NEG	NEG	NEG	NEG	NEG	NEG	NEG	NEG	NEG	NEG	NEG	NEG	NEG	NEG	NEG	NEG	NEG	NEG	NEG	NEG	NEG	NEG	NEG	POS	POS	POS	NEG	NEG	POS	NEG	NEG	NEG	NEG	NEG	NEG	NEG	NEG	NEG	POS	NEG	POS	POS	POS
Iasi-31-355	SSTI	NEG		NEG	NEG	NEG	NEG	NEG	NEG	NEG	NEG	NEG	NEG	NEG	NEG	NEG	NEG	NEG	NEG	NEG	NEG	NEG	NEG	NEG	NEG	NEG	NEG	POS	POS	POS	NEG	NEG	POS	NEG	NEG	NEG	NEG	NEG	NEG	NEG	NEG	NEG	NEG	NEG	POS	AMB	NEG
Iasi-38-377	SSTI	NEG		NEG	NEG	NEG	NEG	NEG	NEG	NEG	NEG	NEG	NEG	NEG	NEG	NEG	NEG	NEG	NEG	NEG	NEG	NEG	NEG	NEG	NEG	NEG	NEG	POS	POS	POS	NEG	NEG	POS	NEG	NEG	NEG	NEG	NEG	NEG	NEG	NEG	NEG	NEG	NEG	POS	POS	NEG
Iasi-41-388	SSTI	NEG		NEG	NEG	NEG	NEG	NEG	NEG	NEG	NEG	NEG	NEG	NEG	NEG	NEG	NEG	NEG	NEG	NEG	NEG	NEG	NEG	NEG	NEG	NEG	NEG	POS	POS	POS	NEG	NEG	POS	NEG	NEG	NEG	NEG	NEG	NEG	NEG	NEG	NEG	NEG	NEG	POS	POS	NEG
Iasi-46-417	SSTI	NEG		NEG	NEG	NEG	NEG	NEG	NEG	NEG	NEG	NEG	NEG	NEG	NEG	NEG	NEG	NEG	NEG	NEG	NEG	NEG	NEG	NEG	NEG	NEG	NEG	POS	POS	POS	NEG	NEG	POS	NEG	NEG	NEG	NEG	NEG	NEG	NEG	NEG	NEG	NEG	NEG	POS	POS	NEG
Iasi-02-S9	Carrier	NEG		NEG	NEG	NEG	AMB	NEG	NEG	NEG	NEG	NEG	NEG	NEG	NEG	NEG	NEG	NEG	NEG	NEG	NEG	NEG	NEG	NEG	NEG	NEG	NEG	NEG	NEG	NEG	NEG	NEG	NEG	NEG	NEG	NEG	NEG	NEG	NEG	AMB	NEG	NEG	NEG	NEG	NEG	NEG	NEG
Iasi-17-S33	Carrier	NEG		NEG	NEG	NEG	NEG	NEG	NEG	NEG	NEG	NEG	NEG	NEG	NEG	NEG	NEG	NEG	NEG	NEG	NEG	NEG	NEG	NEG	NEG	NEG	NEG	POS	POS	POS	NEG	NEG	POS	NEG	NEG	NEG	NEG	NEG	NEG	NEG	NEG	NEG	NEG	NEG	POS	POS	NEG
Iasi-30-P17	Carrier	NEG		NEG	NEG	NEG	NEG	NEG	NEG	NEG	NEG	NEG	NEG	NEG	NEG	NEG	NEG	NEG	NEG	NEG	NEG	NEG	NEG	NEG	NEG	NEG	NEG	POS	POS	POS	NEG	NEG	POS	NEG	NEG	NEG	NEG	NEG	NEG	NEG	NEG	NEG	NEG	NEG	POS	POS	AMB
Iasi-39-P28	Carrier	NEG		NEG	NEG	NEG	NEG	NEG	NEG	NEG	NEG	NEG	NEG	NEG	NEG	NEG	NEG	NEG	NEG	NEG	NEG	NEG	NEG	NEG	NEG	NEG	NEG	POS	POS	POS	NEG	NEG	POS	NEG	NEG	NEG	NEG	NEG	NEG	NEG	NEG	NEG	NEG	NEG	POS	POS	NEG
Iasi-04-26	BSI	POS		POS	POS	NEG	NEG	NEG	NEG	POS	POS	NEG	NEG	NEG	NEG	NEG	NEG	NEG	NEG	NEG	NEG	NEG	NEG	NEG	NEG	NEG	NEG	POS	POS	POS	NEG	NEG	POS	NEG	NEG	NEG	NEG	NEG	NEG	NEG	NEG	NEG	NEG	NEG	POS	POS	NEG
Iasi-09-72	BSI	POS		POS	POS	NEG	NEG	NEG	NEG	POS	POS	NEG	NEG	NEG	NEG	NEG	NEG	NEG	NEG	NEG	NEG	NEG	NEG	NEG	NEG	NEG	NEG	POS	POS	POS	NEG	NEG	POS	NEG	NEG	NEG	NEG	NEG	NEG	NEG	NEG	NEG	NEG	NEG	POS	POS	NEG
Iasi-11-131	BSI	POS		POS	POS	NEG	NEG	NEG	NEG	POS	POS	NEG	NEG	NEG	NEG	NEG	NEG	NEG	NEG	NEG	NEG	NEG	NEG	NEG	NEG	NEG	NEG	POS	POS	POS	NEG	NEG	POS	NEG	NEG	NEG	NEG	NEG	NEG	NEG	NEG	NEG	NEG	NEG	POS	POS	NEG
Iasi-14-143	BSI	POS		POS	POS	NEG	NEG	NEG	NEG	POS	POS	NEG	NEG	NEG	NEG	NEG	NEG	NEG	NEG	NEG	NEG	NEG	NEG	NEG	NEG	NEG	NEG	POS	POS	POS	NEG	NEG	POS	NEG	NEG	NEG	AMB	NEG	NEG	NEG	NEG	NEG	NEG	NEG	POS	POS	NEG
Iasi-18-192	BSI	POS		POS	POS	NEG	NEG	NEG	NEG	POS	POS	NEG	NEG	NEG	NEG	NEG	NEG	NEG	NEG	NEG	NEG	NEG	NEG	NEG	NEG	NEG	NEG	POS	POS	POS	NEG	NEG	POS	NEG	NEG	NEG	NEG	NEG	NEG	NEG	NEG	NEG	NEG	NEG	POS	POS	NEG
Iasi-23-253	BSI	POS		POS	POS	NEG	NEG	NEG	NEG	POS	POS	NEG	NEG	NEG	NEG	NEG	NEG	NEG	NEG	AMB	NEG	NEG	NEG	NEG	NEG	NEG	NEG	POS	POS	POS	NEG	NEG	POS	NEG	NEG	NEG	NEG	NEG	NEG	AMB	NEG	NEG	NEG	NEG	POS	POS	NEG
Iasi-35-322	BSI	POS		POS	POS	NEG	NEG	NEG	NEG	POS	POS	NEG	NEG	NEG	NEG	NEG	NEG	NEG	NEG	NEG	NEG	NEG	NEG	NEG	NEG	NEG	NEG	POS	POS	POS	NEG	NEG	POS	NEG	NEG	NEG	NEG	NEG	NEG	NEG	NEG	NEG	NEG	NEG	POS	POS	NEG
Iasi-40-380	BSI	POS		POS	POS	NEG	NEG	NEG	NEG	POS	POS	NEG	NEG	NEG	NEG	NEG	NEG	NEG	NEG	NEG	NEG	NEG	NEG	NEG	NEG	NEG	NEG	POS	POS	POS	NEG	NEG	POS	NEG	NEG	NEG	NEG	NEG	NEG	NEG	NEG	NEG	NEG	NEG	POS	POS	NEG
Iasi-46-406	BSI	POS		POS	POS	NEG	NEG	NEG	NEG	POS	POS	NEG	NEG	NEG	NEG	NEG	NEG	NEG	NEG	NEG	NEG	NEG	NEG	NEG	NEG	NEG	NEG	POS	POS	POS	NEG	NEG	POS	NEG	NEG	NEG	NEG	NEG	NEG	NEG	NEG	NEG	NEG	NEG	POS	POS	NEG
Iasi-02-6	SSTI	POS		POS	POS	NEG	NEG	NEG	NEG	POS	POS	NEG	NEG	NEG	NEG	NEG	NEG	NEG	NEG	NEG	NEG	NEG	NEG	NEG	NEG	NEG	NEG	POS	POS	POS	NEG	NEG	POS	NEG	NEG	NEG	NEG	NEG	NEG	NEG	NEG	NEG	NEG	NEG	POS	POS	NEG
Iasi-09-97	SSTI	POS		POS	POS	NEG	NEG	NEG	NEG	POS	POS	NEG	NEG	NEG	NEG	NEG	NEG	NEG	NEG	NEG	NEG	NEG	NEG	NEG	NEG	NEG	NEG	POS	POS	POS	NEG	NEG	POS	NEG	NEG	NEG	NEG	NEG	NEG	NEG	NEG	NEG	POS	NEG	POS	POS	NEG
Iasi-19-182	SSTI	POS		POS	POS	NEG	NEG	NEG	NEG	POS	POS	NEG	NEG	NEG	NEG	NEG	NEG	NEG	NEG	AMB	NEG	NEG	NEG	NEG	NEG	NEG	NEG	POS	POS	POS	NEG	NEG	POS	NEG	NEG	NEG	NEG	NEG	NEG	NEG	NEG	NEG	NEG	NEG	POS	POS	NEG
Iasi-31-314	SSTI	POS		POS	POS	NEG	NEG	NEG	NEG	POS	POS	NEG	NEG	NEG	NEG	NEG	NEG	NEG	NEG	NEG	NEG	NEG	NEG	NEG	NEG	NEG	NEG	POS	POS	POS	NEG	NEG	POS	NEG	NEG	NEG	NEG	NEG	NEG	NEG	NEG	NEG	NEG	NEG	POS	POS	NEG
Iasi-33-367	SSTI	POS		POS	POS	NEG	NEG	NEG	NEG	POS	POS	NEG	NEG	NEG	NEG	NEG	NEG	NEG	NEG	AMB	NEG	NEG	NEG	NEG	NEG	NEG	NEG	POS	POS	POS	NEG	NEG	POS	NEG	NEG	NEG	NEG	NEG	NEG	NEG	NEG	NEG	NEG	NEG	POS	POS	NEG
Iasi-39-378	SSTI	POS		POS	POS	NEG	NEG	NEG	NEG	POS	POS	NEG	NEG	NEG	NEG	NEG	NEG	NEG	NEG	POS	NEG	NEG	NEG	NEG	NEG	NEG	NEG	POS	POS	POS	NEG	NEG	POS	NEG	NEG	NEG	NEG	NEG	NEG	NEG	NEG	NEG	NEG	NEG	POS	POS	NEG
Iasi-42-394	SSTI	POS		POS	POS	NEG	NEG	NEG	NEG	POS	POS	NEG	NEG	NEG	NEG	NEG	NEG	NEG	NEG	AMB	NEG	NEG	NEG	NEG	NEG	NEG	NEG	POS	POS	POS	NEG	NEG	POS	NEG	NEG	NEG	NEG	NEG	NEG	NEG	NEG	NEG	NEG	NEG	POS	POS	NEG
Iasi-44-409	SSTI	POS		POS	POS	NEG	NEG	NEG	NEG	POS	POS	NEG	NEG	NEG	NEG	NEG	NEG	NEG	NEG	AMB	NEG	NEG	NEG	NEG	NEG	NEG	NEG	POS	POS	POS	NEG	NEG	POS	NEG	NEG	NEG	NEG	NEG	NEG	NEG	NEG	NEG	POS	NEG	POS	POS	NEG
Iasi-45-405	SSTI	POS		POS	POS	NEG	NEG	NEG	NEG	POS	POS	NEG	NEG	NEG	NEG	NEG	NEG	NEG	NEG	NEG	NEG	NEG	NEG	NEG	NEG	NEG	NEG	POS	POS	POS	NEG	NEG	POS	NEG	NEG	NEG	NEG	NEG	NEG	NEG	NEG	NEG	NEG	NEG	POS	POS	NEG
Iasi-50-425	SSTI	POS		POS	POS	NEG	NEG	NEG	NEG	POS	POS	NEG	NEG	NEG	NEG	NEG	NEG	NEG	NEG	NEG	NEG	NEG	NEG	NEG	NEG	NEG	NEG	POS	POS	POS	NEG	NEG	POS	NEG	NEG	NEG	NEG	NEG	NEG	NEG	NEG	NEG	NEG	NEG	POS	POS	NEG
Iasi-50-428	SSTI	POS		POS	POS	NEG	NEG	NEG	NEG	POS	POS	NEG	NEG	NEG	NEG	NEG	NEG	NEG	NEG	AMB	NEG	NEG	NEG	NEG	NEG	NEG	NEG	POS	POS	POS	NEG	NEG	POS	NEG	NEG	NEG	NEG	NEG	NEG	NEG	NEG	NEG	NEG	NEG	POS	POS	NEG
Iasi-51-37	SSTI	POS		POS	POS	NEG	NEG	NEG	NEG	POS	POS	NEG	NEG	NEG	NEG	NEG	NEG	NEG	NEG	NEG	NEG	NEG	NEG	NEG	NEG	NEG	NEG	POS	POS	POS	NEG	NEG	POS	NEG	NEG	NEG	NEG	NEG	NEG	NEG	NEG	NEG	NEG	NEG	POS	POS	NEG
Iasi-01-S8	Carrier	POS		POS	POS	NEG	NEG	NEG	NEG	POS	POS	NEG	NEG	NEG	NEG	NEG	NEG	NEG	NEG	NEG	NEG	NEG	NEG	NEG	NEG	NEG	NEG	POS	POS	POS	NEG	NEG	POS	NEG	NEG	NEG	NEG	NEG	NEG	AMB	NEG	NEG	NEG	NEG	POS	POS	NEG
Iasi-08-S17	Carrier	POS		POS	POS	NEG	NEG	NEG	NEG	POS	POS	NEG	NEG	NEG	NEG	NEG	NEG	NEG	NEG	NEG	NEG	NEG	NEG	NEG	NEG	NEG	NEG	POS	POS	POS	NEG	NEG	POS	NEG	NEG	NEG	NEG	NEG	NEG	NEG	NEG	NEG	NEG	NEG	POS	POS	NEG
Iasi-10-S23	Carrier	POS		POS	POS	NEG	NEG	NEG	NEG	POS	POS	NEG	NEG	NEG	NEG	NEG	NEG	NEG	NEG	NEG	NEG	NEG	NEG	NEG	NEG	NEG	NEG	POS	POS	POS	NEG	NEG	POS	NEG	NEG	NEG	NEG	NEG	NEG	NEG	NEG	NEG	NEG	NEG	POS	POS	NEG
Iasi-13-S27	Carrier	POS		POS	POS	NEG	NEG	NEG	NEG	POS	POS	NEG	NEG	NEG	NEG	NEG	NEG	NEG	NEG	NEG	NEG	NEG	NEG	NEG	NEG	NEG	NEG	POS	POS	POS	NEG	NEG	POS	NEG	NEG	NEG	NEG	NEG	NEG	AMB	NEG	NEG	NEG	NEG	POS	POS	NEG
Iasi-14-S28	Carrier	POS		POS	POS	NEG	NEG	NEG	NEG	POS	POS	NEG	NEG	NEG	NEG	NEG	NEG	NEG	NEG	NEG	NEG	NEG	NEG	NEG	NEG	NEG	NEG	POS	POS	POS	NEG	NEG	POS	NEG	NEG	NEG	NEG	NEG	NEG	NEG	NEG	NEG	NEG	NEG	POS	POS	NEG
Iasi-15-S29	Carrier	POS		POS	POS	NEG	NEG	NEG	NEG	POS	POS	NEG	NEG	NEG	NEG	NEG	NEG	AMB	NEG	NEG	NEG	NEG	NEG	NEG	NEG	NEG	NEG	POS	POS	POS	NEG	NEG	POS	NEG	NEG	NEG	NEG	NEG	NEG	AMB	NEG	NEG	NEG	NEG	POS	POS	NEG
Iasi-25-P10	Carrier	POS		POS	POS	NEG	NEG	NEG	NEG	POS	POS	NEG	NEG	NEG	NEG	NEG	NEG	AMB	NEG	NEG	NEG	NEG	NEG	NEG	NEG	NEG	NEG	POS	POS	POS	NEG	NEG	POS	NEG	NEG	NEG	NEG	NEG	NEG	AMB	NEG	NEG	POS	NEG	POS	POS	NEG
Iasi-26-P11	Carrier	POS		POS	POS	NEG	NEG	NEG	NEG	POS	POS	NEG	NEG	NEG	NEG	NEG	NEG	NEG	NEG	NEG	NEG	NEG	NEG	NEG	NEG	NEG	NEG	POS	POS	POS	NEG	NEG	POS	NEG	POS	NEG	POS	NEG	NEG	NEG	NEG	NEG	POS	NEG	POS	POS	NEG
Iasi-32-P19	Carrier	POS		POS	POS	NEG	NEG	NEG	NEG	POS	POS	NEG	NEG	NEG	NEG	NEG	NEG	NEG	NEG	NEG	NEG	NEG	NEG	NEG	NEG	NEG	NEG	POS	POS	POS	NEG	NEG	POS	NEG	NEG	NEG	NEG	NEG	NEG	NEG	NEG	NEG	POS	NEG	POS	POS	NEG
Iasi-33-P22	Carrier	POS		POS	POS	NEG	NEG	NEG	NEG	POS	POS	NEG	NEG	NEG	NEG	NEG	NEG	NEG	NEG	NEG	NEG	NEG	NEG	NEG	NEG	NEG	NEG	POS	POS	POS	NEG	NEG	POS	NEG	NEG	NEG	NEG	NEG	NEG	NEG	NEG	NEG	NEG	NEG	POS	POS	NEG
Iasi-41-P44	Carrier	POS		POS	POS	NEG	NEG	NEG	NEG	POS	POS	NEG	NEG	NEG	NEG	NEG	NEG	NEG	NEG	NEG	NEG	NEG	NEG	NEG	NEG	NEG	NEG	POS	POS	POS	NEG	NEG	POS	NEG	NEG	NEG	NEG	NEG	NEG	NEG	NEG	NEG	NEG	NEG	POS	POS	NEG
Iasi-45-G515	Carrier	POS		POS	POS	NEG	NEG	NEG	NEG	POS	POS	NEG	NEG	NEG	NEG	NEG	NEG	NEG	NEG	NEG	NEG	NEG	NEG	NEG	NEG	NEG	NEG	POS	POS	POS	NEG	NEG	POS	NEG	NEG	NEG	NEG	NEG	NEG	NEG	NEG	NEG	NEG	NEG	POS	POS	NEG
Iasi-02-39	misc. MRSA	POS		POS	POS	NEG	NEG	NEG	NEG	POS	POS	NEG	NEG	NEG	NEG	NEG	NEG	NEG	NEG	NEG	NEG	NEG	NEG	NEG	NEG	NEG	NEG	POS	POS	POS	NEG	NEG	POS	NEG	NEG	NEG	NEG	NEG	NEG	NEG	NEG	NEG	NEG	NEG	POS	POS	NEG
Iasi-24-263	misc. MRSA	POS		POS	POS	NEG	NEG	NEG	NEG	POS	POS	NEG	NEG	NEG	NEG	NEG	NEG	NEG	NEG	NEG	NEG	NEG	NEG	NEG	NEG	NEG	NEG	POS	POS	POS	NEG	NEG	POS	NEG	NEG	NEG	NEG	NEG	NEG	NEG	NEG	NEG	NEG	NEG	POS	POS	NEG
Iasi-26-285	misc. MRSA	POS		POS	POS	NEG	NEG	NEG	NEG	POS	POS	NEG	NEG	NEG	NEG	NEG	NEG	NEG	NEG	NEG	NEG	NEG	NEG	NEG	NEG	NEG	NEG	POS	POS	POS	NEG	POS	POS	NEG	NEG	NEG	NEG	NEG	NEG	NEG	NEG	NEG	NEG	NEG	POS	POS	NEG
Iasi-30-306	misc. MRSA	POS		POS	POS	NEG	NEG	NEG	NEG	POS	POS	NEG	NEG	NEG	NEG	NEG	NEG	NEG	NEG	AMB	NEG	NEG	NEG	NEG	NEG	NEG	NEG	POS	POS	POS	NEG	NEG	POS	NEG	NEG	NEG	NEG	NEG	NEG	AMB	NEG	NEG	NEG	NEG	POS	POS	NEG
Iasi-41-390	misc. MRSA	POS		POS	POS	NEG	NEG	NEG	NEG	POS	POS	NEG	NEG	NEG	NEG	NEG	NEG	NEG	NEG	NEG	NEG	NEG	NEG	NEG	NEG	NEG	NEG	POS	POS	POS	NEG	NEG	POS	NEG	NEG	NEG	NEG	NEG	NEG	NEG	NEG	NEG	NEG	NEG	POS	POS	NEG
Iasi-47-407	misc. MRSA	POS		POS	POS	NEG	NEG	NEG	NEG	POS	POS	NEG	NEG	NEG	NEG	NEG	NEG	NEG	NEG	NEG	NEG	NEG	NEG	NEG	NEG	NEG	NEG	POS	POS	POS	NEG	NEG	POS	NEG	NEG	NEG	NEG	NEG	NEG	NEG	NEG	NEG	NEG	NEG	POS	POS	NEG
Iasi-55-408CP	misc. MRSA	POS		POS	POS	NEG	NEG	NEG	NEG	POS	POS	NEG	NEG	NEG	NEG	NEG	NEG	NEG	NEG	NEG	NEG	NEG	NEG	NEG	NEG	NEG	NEG	POS	POS	POS	NEG	NEG	POS	NEG	NEG	NEG	NEG	NEG	NEG	NEG	NEG	NEG	NEG	NEG	POS	POS	NEG
Iasi-15-151	BSI	NEG		NEG	NEG	NEG	NEG	NEG	NEG	NEG	NEG	NEG	NEG	NEG	NEG	NEG	NEG	NEG	NEG	NEG	NEG	NEG	NEG	NEG	NEG	NEG	NEG	POS	POS	POS	NEG	NEG	POS	NEG	NEG	NEG	NEG	NEG	NEG	NEG	NEG	NEG	NEG	NEG	NEG	NEG	NEG
Iasi-46-386	BSI	NEG		NEG	NEG	NEG	NEG	NEG	NEG	NEG	NEG	NEG	NEG	NEG	NEG	NEG	NEG	AMB	NEG	NEG	NEG	NEG	NEG	NEG	NEG	NEG	NEG	NEG	AMB	NEG	POS	NEG	NEG	NEG	NEG	NEG	NEG	NEG	NEG	AMB	NEG	NEG	NEG	NEG	NEG	NEG	NEG
Iasi-21-195	SSTI	NEG		NEG	NEG	POS	NEG	NEG	NEG	NEG	NEG	NEG	NEG	NEG	NEG	NEG	NEG	NEG	NEG	NEG	NEG	NEG	NEG	NEG	NEG	NEG	NEG	POS	POS	POS	NEG	NEG	POS	NEG	NEG	NEG	NEG	NEG	NEG	NEG	NEG	NEG	NEG	NEG	NEG	NEG	NEG
Iasi-37-375	SSTI	NEG		NEG	NEG	NEG	NEG	NEG	NEG	NEG	NEG	NEG	NEG	NEG	NEG	NEG	NEG	NEG	NEG	NEG	NEG	NEG	NEG	NEG	NEG	NEG	NEG	NEG	NEG	NEG	NEG	NEG	NEG	NEG	NEG	NEG	NEG	NEG	NEG	NEG	NEG	NEG	NEG	NEG	NEG	NEG	NEG
Iasi-45-415	SSTI	NEG		NEG	NEG	NEG	NEG	NEG	NEG	NEG	NEG	NEG	NEG	NEG	NEG	NEG	NEG	NEG	NEG	NEG	NEG	NEG	NEG	NEG	NEG	NEG	NEG	POS	POS	POS	NEG	NEG	NEG	NEG	NEG	NEG	NEG	NEG	NEG	NEG	NEG	NEG	NEG	NEG	NEG	NEG	NEG
Iasi-09-S19	Carrier	NEG		NEG	NEG	NEG	AMB	NEG	NEG	NEG	NEG	NEG	NEG	NEG	NEG	NEG	NEG	AMB	NEG	NEG	NEG	NEG	NEG	NEG	NEG	NEG	NEG	NEG	AMB	NEG	POS	NEG	NEG	NEG	NEG	NEG	NEG	NEG	NEG	AMB	NEG	NEG	POS	NEG	NEG	NEG	NEG
Iasi-53-434	BSI	NEG		NEG	NEG	NEG	NEG	NEG	NEG	NEG	NEG	NEG	NEG	NEG	NEG	NEG	NEG	NEG	NEG	NEG	NEG	NEG	NEG	NEG	NEG	NEG	NEG	NEG	AMB	NEG	POS	NEG	NEG	NEG	NEG	NEG	NEG	NEG	NEG	AMB	NEG	NEG	NEG	NEG	NEG	NEG	NEG
Iasi-03-40	misc. MRSA	POS		POS	POS	NEG	NEG	NEG	POS	POS	POS	POS	POS	POS	POS	POS	POS	POS	POS	NEG	NEG	NEG	NEG	NEG	NEG	NEG	NEG	POS	POS	POS	POS	NEG	NEG	NEG	NEG	NEG	NEG	NEG	NEG	NEG	NEG	NEG	NEG	POS	NEG	NEG	NEG
Iasi-04-43	misc. MRSA	POS		POS	POS	NEG	NEG	NEG	POS	POS	POS	POS	POS	POS	POS	POS	POS	POS	POS	NEG	NEG	NEG	NEG	NEG	NEG	NEG	NEG	POS	POS	POS	POS	NEG	NEG	NEG	NEG	NEG	NEG	NEG	NEG	NEG	NEG	NEG	NEG	POS	NEG	NEG	NEG
Iasi-01-20	misc. MRSA	POS		POS	POS	NEG	NEG	NEG	POS	POS	POS	NEG	NEG	NEG	NEG	NEG	NEG	NEG	NEG	NEG	NEG	NEG	NEG	NEG	NEG	NEG	NEG	POS	POS	POS	NEG	NEG	NEG	NEG	NEG	NEG	NEG	NEG	NEG	NEG	NEG	NEG	NEG	NEG	POS	POS	NEG
Iasi-35-228	BSI	NEG		NEG	NEG	NEG	NEG	NEG	NEG	NEG	NEG	NEG	NEG	NEG	NEG	NEG	NEG	NEG	NEG	NEG	NEG	NEG	NEG	NEG	NEG	NEG	NEG	POS	POS	POS	NEG	NEG	NEG	NEG	NEG	NEG	NEG	NEG	NEG	NEG	NEG	NEG	NEG	NEG	NEG	NEG	NEG
Iasi-38-293	BSI	NEG		NEG	NEG	NEG	NEG	NEG	NEG	NEG	NEG	NEG	NEG	NEG	NEG	NEG	NEG	NEG	NEG	NEG	NEG	NEG	NEG	NEG	NEG	NEG	NEG	POS	POS	POS	NEG	NEG	NEG	NEG	NEG	NEG	NEG	NEG	NEG	AMB	NEG	NEG	NEG	NEG	NEG	NEG	NEG
Iasi-31-P18	Carrier	NEG		NEG	NEG	NEG	NEG	NEG	NEG	NEG	NEG	NEG	NEG	NEG	NEG	NEG	NEG	NEG	NEG	NEG	NEG	NEG	NEG	NEG	NEG	NEG	NEG	POS	POS	POS	NEG	NEG	NEG	NEG	NEG	NEG	NEG	NEG	NEG	NEG	NEG	NEG	NEG	NEG	NEG	NEG	NEG
Iasi-40-P42	Carrier	NEG		NEG	NEG	NEG	AMB	NEG	NEG	NEG	NEG	NEG	NEG	NEG	NEG	NEG	NEG	NEG	NEG	NEG	NEG	NEG	NEG	NEG	NEG	NEG	NEG	POS	POS	POS	NEG	NEG	NEG	NEG	NEG	NEG	NEG	NEG	NEG	AMB	NEG	NEG	NEG	NEG	NEG	NEG	NEG
Iasi-40-303	BSI	NEG		NEG	NEG	NEG	NEG	NEG	NEG	NEG	NEG	NEG	NEG	NEG	NEG	NEG	NEG	NEG	NEG	NEG	NEG	NEG	NEG	NEG	NEG	NEG	NEG	POS	POS	POS	NEG	NEG	NEG	NEG	NEG	NEG	NEG	NEG	NEG	NEG	NEG	NEG	NEG	NEG	NEG	NEG	NEG
Iasi-48-422	SSTI	NEG		NEG	NEG	NEG	NEG	NEG	NEG	NEG	NEG	NEG	NEG	NEG	NEG	NEG	NEG	NEG	NEG	NEG	NEG	NEG	NEG	NEG	NEG	NEG	NEG	POS	POS	POS	NEG	NEG	NEG	NEG	NEG	NEG	NEG	NEG	NEG	NEG	NEG	NEG	NEG	NEG	NEG	NEG	NEG
Iasi-54-435	BSI	NEG		NEG	AMB	NEG	NEG	NEG	NEG	NEG	NEG	NEG	NEG	NEG	NEG	NEG	NEG	NEG	AMB	NEG	NEG	NEG	NEG	NEG	NEG	NEG	NEG	POS	POS	POS	NEG	NEG	NEG	NEG	POS	NEG	POS	NEG	NEG	POS	NEG	NEG	NEG	NEG	POS	POS	AMB
Iasi-16-161	SSTI	NEG		NEG	NEG	NEG	NEG	NEG	NEG	NEG	NEG	NEG	NEG	NEG	NEG	NEG	NEG	NEG	NEG	NEG	NEG	NEG	NEG	NEG	NEG	NEG	NEG	POS	POS	POS	NEG	NEG	NEG	NEG	POS	NEG	POS	NEG	NEG	NEG	NEG	NEG	NEG	NEG	POS	POS	NEG
Iasi-27-221	SSTI	NEG		NEG	NEG	NEG	NEG	NEG	NEG	NEG	NEG	NEG	NEG	NEG	NEG	NEG	NEG	NEG	NEG	NEG	NEG	NEG	NEG	NEG	NEG	NEG	NEG	POS	POS	POS	NEG	NEG	NEG	NEG	POS	NEG	POS	NEG	NEG	NEG	NEG	NEG	NEG	NEG	POS	POS	NEG
Iasi-09-85	BSI	POS		POS	POS	NEG	NEG	NEG	AMB	POS	POS	NEG	NEG	NEG	NEG	NEG	NEG	NEG	NEG	NEG	NEG	NEG	NEG	NEG	NEG	NEG	NEG	POS	POS	POS	NEG	NEG	NEG	NEG	POS	NEG	POS	NEG	NEG	NEG	NEG	NEG	NEG	NEG	POS	POS	NEG
Iasi-17-189	BSI	POS		POS	POS	NEG	NEG	NEG	POS	POS	POS	NEG	NEG	NEG	NEG	NEG	NEG	NEG	NEG	NEG	NEG	NEG	NEG	NEG	NEG	NEG	NEG	POS	POS	POS	NEG	NEG	NEG	NEG	POS	NEG	POS	NEG	NEG	NEG	NEG	NEG	NEG	NEG	POS	POS	NEG
Iasi-20-200	BSI	POS		POS	POS	NEG	NEG	NEG	POS	POS	POS	NEG	NEG	NEG	NEG	NEG	NEG	NEG	NEG	NEG	NEG	NEG	NEG	NEG	NEG	NEG	NEG	POS	POS	POS	NEG	NEG	NEG	NEG	POS	NEG	POS	NEG	NEG	NEG	NEG	NEG	NEG	NEG	POS	POS	NEG
Iasi-34-321	BSI	POS		POS	POS	NEG	NEG	NEG	POS	POS	POS	NEG	NEG	NEG	NEG	NEG	NEG	NEG	NEG	NEG	NEG	NEG	NEG	NEG	NEG	NEG	NEG	POS	POS	POS	NEG	NEG	NEG	NEG	POS	NEG	POS	NEG	NEG	NEG	NEG	NEG	NEG	NEG	POS	POS	NEG
Iasi-04-57	SSTI	POS		POS	POS	NEG	NEG	NEG	POS	POS	POS	NEG	NEG	NEG	NEG	NEG	NEG	NEG	NEG	NEG	NEG	NEG	NEG	NEG	NEG	NEG	NEG	POS	POS	POS	NEG	NEG	NEG	NEG	POS	NEG	POS	NEG	NEG	NEG	NEG	NEG	NEG	NEG	POS	POS	NEG
Iasi-05-59	SSTI	POS		POS	POS	NEG	NEG	NEG	POS	POS	POS	NEG	NEG	NEG	NEG	NEG	NEG	NEG	NEG	NEG	NEG	NEG	NEG	NEG	NEG	NEG	NEG	POS	POS	POS	NEG	NEG	NEG	NEG	POS	NEG	POS	NEG	NEG	NEG	NEG	NEG	NEG	NEG	POS	POS	NEG
Iasi-06-64	SSTI	POS		POS	POS	NEG	NEG	NEG	POS	POS	POS	NEG	NEG	NEG	NEG	NEG	NEG	NEG	NEG	AMB	NEG	NEG	NEG	NEG	NEG	NEG	NEG	POS	POS	POS	NEG	NEG	NEG	NEG	POS	NEG	POS	NEG	NEG	NEG	NEG	NEG	NEG	NEG	POS	POS	NEG
Iasi-10-108	SSTI	POS		POS	POS	NEG	NEG	NEG	POS	POS	POS	NEG	NEG	NEG	NEG	NEG	NEG	NEG	NEG	NEG	NEG	NEG	NEG	NEG	NEG	NEG	NEG	POS	POS	POS	NEG	NEG	NEG	NEG	POS	NEG	POS	NEG	NEG	NEG	NEG	NEG	NEG	NEG	POS	POS	NEG
Iasi-12-117	SSTI	POS		POS	POS	NEG	NEG	NEG	POS	POS	POS	NEG	NEG	NEG	NEG	NEG	NEG	NEG	NEG	AMB	NEG	NEG	NEG	NEG	NEG	NEG	NEG	POS	POS	POS	NEG	NEG	NEG	NEG	POS	NEG	POS	NEG	NEG	NEG	NEG	NEG	NEG	NEG	POS	POS	NEG
Iasi-12-138	SSTI	POS		POS	POS	NEG	NEG	NEG	POS	POS	POS	NEG	NEG	NEG	NEG	NEG	NEG	NEG	NEG	NEG	NEG	NEG	NEG	NEG	NEG	NEG	NEG	POS	POS	POS	NEG	NEG	NEG	NEG	POS	NEG	POS	NEG	NEG	NEG	NEG	NEG	NEG	NEG	POS	POS	NEG
Iasi-13-140	SSTI	POS		POS	POS	NEG	NEG	NEG	POS	POS	POS	NEG	NEG	NEG	NEG	NEG	NEG	NEG	NEG	NEG	NEG	NEG	NEG	NEG	NEG	NEG	NEG	POS	POS	POS	NEG	NEG	NEG	NEG	POS	NEG	POS	NEG	NEG	NEG	NEG	NEG	NEG	NEG	POS	POS	NEG
Iasi-15-152	SSTI	POS		POS	POS	NEG	NEG	NEG	POS	POS	POS	NEG	NEG	NEG	NEG	NEG	NEG	NEG	NEG	NEG	NEG	NEG	NEG	NEG	NEG	NEG	NEG	POS	POS	POS	NEG	NEG	NEG	NEG	POS	NEG	POS	NEG	NEG	NEG	NEG	NEG	NEG	NEG	POS	POS	NEG
Iasi-17-168	SSTI	POS		POS	POS	NEG	NEG	NEG	POS	POS	POS	NEG	NEG	NEG	NEG	NEG	NEG	NEG	NEG	AMB	NEG	NEG	NEG	NEG	NEG	NEG	NEG	POS	POS	POS	NEG	NEG	NEG	NEG	POS	NEG	POS	NEG	NEG	NEG	NEG	NEG	NEG	NEG	POS	POS	NEG
Iasi-20-193	SSTI	POS		POS	POS	NEG	NEG	NEG	POS	POS	POS	NEG	NEG	NEG	NEG	NEG	NEG	NEG	NEG	AMB	NEG	NEG	NEG	NEG	NEG	NEG	NEG	POS	POS	POS	NEG	NEG	NEG	NEG	POS	NEG	POS	NEG	NEG	NEG	NEG	NEG	NEG	NEG	POS	POS	NEG
Iasi-26-218	SSTI	POS		POS	POS	NEG	NEG	NEG	POS	POS	POS	NEG	NEG	NEG	NEG	NEG	NEG	NEG	NEG	NEG	NEG	NEG	NEG	NEG	NEG	NEG	NEG	POS	POS	POS	NEG	NEG	NEG	NEG	NEG	NEG	NEG	NEG	NEG	NEG	NEG	NEG	NEG	NEG	NEG	NEG	NEG
Iasi-28-335	SSTI	POS		POS	POS	NEG	NEG	NEG	POS	POS	POS	NEG	NEG	NEG	NEG	NEG	NEG	NEG	NEG	NEG	NEG	NEG	NEG	NEG	NEG	NEG	NEG	POS	POS	POS	NEG	NEG	NEG	NEG	NEG	NEG	NEG	NEG	NEG	NEG	NEG	NEG	NEG	NEG	NEG	NEG	NEG
Iasi-29-299b	SSTI	POS		POS	POS	NEG	NEG	NEG	POS	POS	POS	NEG	NEG	NEG	NEG	NEG	NEG	NEG	NEG	NEG	NEG	NEG	NEG	NEG	NEG	NEG	NEG	POS	POS	POS	NEG	NEG	NEG	NEG	POS	NEG	POS	NEG	NEG	NEG	NEG	NEG	NEG	NEG	POS	POS	NEG
Iasi-32-364	SSTI	POS		POS	POS	NEG	NEG	NEG	POS	POS	POS	NEG	NEG	NEG	NEG	NEG	NEG	NEG	NEG	AMB	NEG	NEG	NEG	NEG	NEG	NEG	NEG	POS	POS	POS	NEG	NEG	NEG	NEG	POS	NEG	POS	NEG	NEG	NEG	NEG	NEG	NEG	NEG	POS	POS	NEG
Iasi-369	SSTI	POS		POS	POS	NEG	NEG	NEG	POS	POS	POS	NEG	NEG	NEG	NEG	NEG	NEG	NEG	NEG	NEG	NEG	NEG	NEG	NEG	NEG	NEG	NEG	POS	POS	POS	NEG	NEG	NEG	NEG	POS	NEG	POS	NEG	NEG	NEG	NEG	NEG	NEG	NEG	POS	POS	NEG
Iasi-03-S11	Carrier	POS		POS	POS	NEG	NEG	NEG	POS	POS	POS	NEG	NEG	NEG	NEG	NEG	NEG	NEG	NEG	NEG	NEG	NEG	NEG	NEG	NEG	NEG	NEG	POS	POS	POS	NEG	NEG	NEG	NEG	POS	NEG	POS	NEG	NEG	NEG	NEG	NEG	NEG	NEG	POS	POS	NEG
Iasi-19-S35	Carrier	POS		POS	POS	NEG	NEG	NEG	POS	POS	POS	NEG	NEG	NEG	NEG	NEG	NEG	NEG	NEG	NEG	NEG	NEG	NEG	NEG	NEG	NEG	NEG	POS	POS	POS	NEG	NEG	NEG	NEG	POS	NEG	POS	NEG	NEG	AMB	NEG	NEG	NEG	NEG	POS	POS	NEG
Iasi-20-P1	Carrier	POS		POS	POS	NEG	NEG	NEG	POS	POS	POS	NEG	NEG	NEG	NEG	NEG	NEG	NEG	NEG	NEG	NEG	NEG	NEG	NEG	NEG	NEG	NEG	POS	POS	POS	NEG	NEG	NEG	NEG	POS	NEG	POS	NEG	NEG	NEG	NEG	NEG	NEG	NEG	POS	POS	NEG
Iasi-35-P24	Carrier	POS		POS	POS	NEG	NEG	NEG	POS	POS	POS	NEG	NEG	NEG	NEG	NEG	NEG	NEG	NEG	NEG	NEG	NEG	NEG	NEG	NEG	NEG	NEG	POS	POS	POS	NEG	NEG	NEG	NEG	POS	NEG	POS	NEG	NEG	NEG	NEG	NEG	NEG	NEG	POS	POS	NEG
Iasi-36-P25	Carrier	POS		POS	POS	NEG	NEG	NEG	POS	POS	POS	NEG	NEG	NEG	NEG	NEG	NEG	NEG	NEG	NEG	NEG	NEG	NEG	NEG	NEG	NEG	NEG	POS	POS	POS	NEG	NEG	NEG	NEG	POS	NEG	POS	NEG	NEG	NEG	NEG	NEG	NEG	NEG	POS	POS	NEG
Iasi-14-150	SSTI	POS		POS	POS	NEG	NEG	NEG	POS	POS	POS	NEG	NEG	NEG	NEG	NEG	NEG	NEG	NEG	NEG	NEG	NEG	NEG	NEG	NEG	NEG	NEG	POS	POS	POS	NEG	NEG	NEG	NEG	POS	NEG	POS	NEG	NEG	NEG	NEG	NEG	NEG	NEG	POS	POS	NEG
Iasi-07-54	BSI	POS		POS	POS	NEG	AMB	NEG	POS	NEG	NEG	NEG	NEG	NEG	NEG	NEG	POS	POS	POS	POS	POS	NEG	NEG	NEG	POS	NEG	NEG	POS	POS	POS	POS	NEG	NEG	NEG	NEG	NEG	NEG	NEG	NEG	NEG	NEG	NEG	NEG	NEG	NEG	NEG	NEG
Iasi-16-188	BSI	POS		POS	POS	NEG	AMB	NEG	POS	NEG	NEG	NEG	NEG	NEG	NEG	NEG	POS	POS	POS	POS	POS	NEG	NEG	NEG	POS	NEG	NEG	POS	POS	POS	POS	NEG	NEG	NEG	NEG	NEG	NEG	NEG	NEG	NEG	NEG	NEG	POS	NEG	NEG	NEG	NEG
Iasi-32-318	BSI	POS		POS	POS	NEG	NEG	NEG	AMB	NEG	NEG	NEG	NEG	NEG	NEG	NEG	POS	POS	POS	POS	POS	NEG	NEG	NEG	POS	NEG	NEG	POS	POS	POS	POS	NEG	NEG	NEG	NEG	NEG	NEG	NEG	NEG	NEG	NEG	NEG	POS	NEG	NEG	NEG	POS
Iasi-42-397	BSI	POS		POS	POS	NEG	NEG	NEG	AMB	NEG	NEG	NEG	NEG	NEG	NEG	NEG	POS	POS	POS	POS	POS	NEG	NEG	NEG	POS	NEG	NEG	POS	POS	POS	POS	NEG	NEG	NEG	NEG	NEG	NEG	NEG	NEG	NEG	NEG	NEG	POS	NEG	NEG	NEG	NEG
Iasi-48-408	BSI	POS		POS	POS	NEG	AMB	NEG	POS	NEG	NEG	NEG	NEG	NEG	NEG	NEG	POS	POS	POS	POS	POS	NEG	NEG	NEG	POS	NEG	NEG	POS	POS	POS	POS	NEG	NEG	NEG	NEG	NEG	NEG	NEG	NEG	NEG	NEG	NEG	POS	NEG	NEG	NEG	NEG
Iasi-49-430	BSI	POS		POS	POS	NEG	AMB	NEG	POS	NEG	NEG	NEG	NEG	NEG	NEG	NEG	POS	POS	POS	POS	POS	NEG	NEG	NEG	POS	NEG	NEG	POS	POS	POS	POS	NEG	NEG	NEG	NEG	NEG	NEG	NEG	NEG	NEG	NEG	NEG	POS	NEG	NEG	NEG	NEG
Iasi-52-52	BSI	POS		POS	POS	NEG	NEG	NEG	POS	NEG	NEG	NEG	NEG	NEG	NEG	NEG	POS	POS	POS	POS	POS	NEG	NEG	NEG	POS	NEG	NEG	POS	POS	POS	POS	NEG	NEG	NEG	NEG	NEG	NEG	NEG	NEG	NEG	NEG	NEG	POS	NEG	NEG	NEG	NEG
Iasi-01-2	SSTI	POS		POS	POS	NEG	AMB	NEG	POS	NEG	NEG	NEG	NEG	NEG	NEG	NEG	POS	POS	POS	POS	POS	NEG	NEG	NEG	POS	NEG	NEG	POS	POS	POS	POS	NEG	NEG	NEG	NEG	NEG	NEG	NEG	NEG	NEG	NEG	NEG	POS	NEG	NEG	NEG	NEG
Iasi-10-101	SSTI	POS		POS	POS	NEG	NEG	NEG	POS	NEG	NEG	NEG	NEG	NEG	NEG	NEG	POS	POS	POS	POS	POS	NEG	NEG	NEG	POS	NEG	NEG	POS	POS	POS	POS	NEG	NEG	NEG	NEG	NEG	NEG	NEG	NEG	NEG	NEG	NEG	POS	NEG	NEG	NEG	NEG
Iasi-11-106	SSTI	POS		POS	POS	NEG	AMB	NEG	POS	NEG	NEG	NEG	NEG	NEG	NEG	NEG	POS	POS	POS	POS	POS	NEG	NEG	NEG	POS	NEG	NEG	POS	POS	POS	POS	NEG	NEG	NEG	NEG	NEG	NEG	NEG	NEG	NEG	NEG	NEG	POS	NEG	NEG	NEG	NEG
Iasi-49-414	SSTI	POS		POS	POS	NEG	AMB	NEG	POS	NEG	NEG	NEG	NEG	NEG	NEG	NEG	POS	POS	POS	POS	POS	NEG	NEG	NEG	POS	NEG	NEG	POS	POS	POS	POS	NEG	NEG	NEG	NEG	NEG	NEG	NEG	NEG	NEG	NEG	NEG	POS	NEG	NEG	NEG	NEG

Isolate	Group	METHICILLIN RESISTANCE AND SCCmec TYPING																								RESISTANCE : PENICILLINASE			RESISTANCE : MLS-ANTIBIOTICS												RESISTANCE : AMINOGLYSOSIDES			RESISTANCE : MISCELLANEOUS GENES	
		alternate penicillin binding protein 2, defining MRSA	alternate mec allele	truncated signal transducer protein MecR1	glycerophosphoryl diester phosphodi-esterase, associated with mecA	cassette chromosome recombinase genes A/B-1		plasmin-sensitive surface protein	hypothetical protein from SCCmec elements	cassette chromosome recombinase genes A/B-2		potassium-trans-locating ATPase A, chain 2	potassium-transporting ATPase B, chain 1	potassium-trans-locating ATPase C, chain 2	sensor kinase protein	KDP operon transcrip-tional regulatory protein	methicillin-resistance regulatory protein	signal transducer protein MecR1	homolog of xylose repressor, associated with SCCmec-elements	cassette chromosome recombinase genes A/B-3		mercury resistance operon		cassette chromosome recombinase genes "ccrAA" (hypothetical) and ccrC		cassette chromosome recombinase genes A/B-4		beta-lactamase	beta lactamase repressor (inhibitor)	beta-lactamase regulatory protein	rRNA adenine N-6-methyl-transferase, erythromycin/clindamycin resistance	erythro-mycin/clindamycin resistance	erythro-mycin/clindamycin resistance	Linco-samid-Nucleo-tidyltrans-ferase	energy-dependent efflux of erythro-mycin	macrolide efflux protein A	probable lysylphos-phatidyl-glycerol synthetase	virginia-mycin A acetyltransferase	acetyl-transferase inactivating streptogramin A	ATP binding protein, streptogramin-A-resistance		virginiamycin B hydrolase	bifunctional enzyme Aac/Aph, gentamicin resistance	amino-glycoside adenyl-transferase,tobramycin resistance	3'5'-aminog-lycoside phospho-transferase, neo-/kanamycin resistance	strepto-thricine-acetyl-transferase	dihydro-folate reductase type 1
		mecA	mecC	delta_mecR	ugpQ	ccrA-1	ccrB-1	plsSCC (COL)	Q9XB68-dcs	ccrA-2	ccrB-2	kdpA-SCC	kdpB-SCC	kdpC-SCC	kdpD-SCC	kdpE-SCC	mecI	mecR	xylR	ccrA-3	ccrB-3	merA	merB	ccrAA (MRSAZH47)	ccrC (85-2082)	ccrA-4	ccrB-4	blaZ	blaI	blaR	erm(A)	erm(B)	erm(C)	lnu(A)	msr(A)	mef(A)	mph(C)	vat(A)	vat(B)	vga(A)	vga(A) (BM 3327)	vgB(A)	aacA-aphD	aadD	aphA3	sat	dfrS1
Iasi-05-46	misc. MRSA	POS		POS	POS	NEG	AMB	NEG	POS	NEG	NEG	NEG	NEG	NEG	NEG	NEG	POS	POS	POS	POS	POS	NEG	NEG	NEG	POS	NEG	NEG	POS	POS	POS	POS	NEG	NEG	NEG	NEG	NEG	NEG	NEG	NEG	NEG	NEG	NEG	NEG	NEG	NEG	NEG	NEG
Iasi-06-47	misc. MRSA	POS		POS	POS	NEG	AMB	NEG	POS	NEG	NEG	NEG	NEG	NEG	NEG	NEG	POS	POS	POS	POS	POS	NEG	NEG	NEG	POS	NEG	NEG	POS	POS	POS	POS	NEG	NEG	NEG	NEG	NEG	NEG	NEG	NEG	NEG	NEG	NEG	POS	NEG	NEG	NEG	NEG
Iasi-08-73	misc. MRSA	POS		POS	POS	NEG	AMB	NEG	POS	NEG	NEG	NEG	NEG	NEG	NEG	NEG	POS	POS	POS	POS	POS	NEG	NEG	NEG	POS	NEG	NEG	POS	POS	POS	POS	NEG	NEG	NEG	NEG	NEG	NEG	NEG	NEG	NEG	NEG	NEG	POS	NEG	NEG	NEG	NEG
Iasi-19-194	misc. MRSA	POS		POS	POS	NEG	AMB	NEG	POS	NEG	NEG	NEG	NEG	NEG	NEG	NEG	POS	POS	POS	POS	POS	NEG	NEG	NEG	POS	NEG	NEG	POS	POS	POS	POS	NEG	NEG	NEG	NEG	NEG	NEG	NEG	NEG	NEG	NEG	NEG	POS	NEG	NEG	NEG	NEG
Iasi-21-219	misc. MRSA	POS		POS	POS	NEG	AMB	NEG	POS	NEG	NEG	NEG	NEG	NEG	NEG	NEG	POS	POS	POS	POS	POS	NEG	NEG	NEG	POS	NEG	NEG	POS	POS	POS	POS	NEG	NEG	NEG	NEG	NEG	NEG	NEG	NEG	NEG	NEG	NEG	POS	NEG	NEG	NEG	NEG
Iasi-25-284	misc. MRSA	POS		POS	POS	NEG	AMB	NEG	POS	NEG	NEG	NEG	NEG	NEG	NEG	NEG	POS	POS	POS	POS	POS	NEG	NEG	NEG	POS	NEG	NEG	POS	POS	POS	POS	NEG	NEG	NEG	NEG	NEG	NEG	NEG	NEG	NEG	NEG	NEG	POS	NEG	NEG	NEG	NEG
Iasi-284	misc. MRSA	POS		POS	POS	NEG	NEG	NEG	POS	NEG	NEG	NEG	NEG	NEG	NEG	NEG	POS	POS	POS	POS	POS	NEG	NEG	NEG	POS	NEG	NEG	POS	POS	POS	POS	NEG	NEG	NEG	NEG	NEG	NEG	NEG	NEG	NEG	NEG	NEG	POS	NEG	NEG	NEG	NEG
Iasi-36-336	misc. MRSA	POS		POS	POS	NEG	NEG	NEG	POS	NEG	NEG	NEG	NEG	NEG	NEG	NEG	POS	POS	POS	POS	POS	NEG	NEG	NEG	POS	NEG	NEG	POS	POS	POS	POS	NEG	NEG	NEG	NEG	NEG	NEG	NEG	NEG	NEG	NEG	NEG	POS	NEG	NEG	NEG	NEG
Iasi-43-402	misc. MRSA	POS		POS	POS	NEG	POS	NEG	POS	NEG	NEG	NEG	NEG	NEG	NEG	NEG	POS	POS	POS	POS	POS	NEG	NEG	NEG	POS	NEG	NEG	POS	POS	POS	POS	NEG	NEG	NEG	NEG	NEG	NEG	NEG	NEG	NEG	NEG	NEG	POS	NEG	NEG	NEG	NEG
Iasi-44-404	misc. MRSA	POS		POS	POS	NEG	NEG	NEG	AMB	NEG	NEG	NEG	NEG	NEG	NEG	NEG	POS	POS	POS	POS	POS	NEG	NEG	NEG	POS	NEG	NEG	POS	POS	POS	POS	NEG	NEG	NEG	NEG	NEG	NEG	NEG	NEG	NEG	NEG	NEG	POS	NEG	NEG	NEG	NEG
Iasi-54-430	misc. MRSA	POS		POS	POS	NEG	POS	NEG	POS	NEG	NEG	NEG	NEG	NEG	NEG	NEG	POS	POS	POS	POS	POS	NEG	NEG	NEG	POS	NEG	NEG	POS	POS	POS	POS	NEG	AMB	NEG	NEG	NEG	NEG	NEG	NEG	AMB	NEG	NEG	POS	NEG	NEG	NEG	AMB
Iasi-24-216	SSTI	NEG		NEG	NEG	NEG	NEG	NEG	NEG	NEG	NEG	NEG	NEG	NEG	NEG	NEG	NEG	NEG	NEG	NEG	NEG	NEG	NEG	NEG	NEG	NEG	NEG	POS	POS	POS	NEG	NEG	NEG	NEG	NEG	NEG	NEG	NEG	NEG	NEG	NEG	NEG	NEG	NEG	NEG	NEG	NEG
Iasi-05-S13	Carrier	NEG		NEG	NEG	NEG	NEG	NEG	NEG	NEG	NEG	NEG	NEG	NEG	NEG	NEG	NEG	NEG	NEG	NEG	NEG	NEG	NEG	NEG	NEG	NEG	NEG	POS	POS	POS	NEG	NEG	NEG	NEG	NEG	NEG	NEG	NEG	NEG	NEG	NEG	NEG	NEG	NEG	NEG	NEG	NEG
Iasi-45-362	BSI	NEG	NEG	NEG	NEG	NEG	NEG	NEG	NEG	NEG	NEG	NEG	NEG	NEG	NEG	NEG	NEG	NEG	NEG	NEG	NEG	NEG	NEG	NEG	NEG	NEG	NEG	POS	POS	POS	NEG	NEG	NEG	NEG	NEG	NEG	NEG	NEG	NEG	NEG	NEG	NEG	NEG	NEG	NEG	NEG	NEG
Iasi-22-199	SSTI	NEG		NEG	NEG	NEG	NEG	NEG	NEG	NEG	NEG	NEG	NEG	NEG	NEG	NEG	NEG	NEG	NEG	NEG	NEG	NEG	NEG	NEG	NEG	NEG	NEG	POS	POS	POS	NEG	NEG	NEG	NEG	NEG	NEG	NEG	NEG	NEG	NEG	NEG	NEG	NEG	NEG	NEG	NEG	NEG
Iasi-12-S26	Carrier	NEG		NEG	NEG	NEG	NEG	NEG	NEG	NEG	NEG	NEG	NEG	NEG	NEG	NEG	NEG	NEG	NEG	NEG	NEG	NEG	NEG	NEG	NEG	NEG	NEG	NEG	NEG	NEG	NEG	NEG	NEG	NEG	NEG	NEG	NEG	NEG	NEG	AMB	NEG	NEG	NEG	NEG	NEG	NEG	NEG
Iasi-21-P2	Carrier	NEG		NEG	NEG	NEG	NEG	NEG	NEG	NEG	NEG	NEG	NEG	NEG	NEG	NEG	NEG	NEG	NEG	NEG	NEG	NEG	NEG	NEG	NEG	NEG	NEG	POS	POS	POS	NEG	NEG	NEG	NEG	NEG	NEG	NEG	NEG	NEG	AMB	NEG	NEG	NEG	NEG	NEG	NEG	NEG
Iasi-06-60	BSI	NEG		NEG	NEG	NEG	NEG	NEG	NEG	NEG	NEG	NEG	NEG	NEG	NEG	NEG	NEG	NEG	NEG	NEG	NEG	NEG	NEG	NEG	NEG	NEG	NEG	POS	POS	POS	NEG	NEG	NEG	NEG	NEG	NEG	NEG	NEG	NEG	NEG	NEG	NEG	NEG	NEG	NEG	NEG	NEG
Iasi-12-79	BSI	NEG		NEG	NEG	NEG	NEG	NEG	NEG	NEG	NEG	NEG	NEG	NEG	NEG	NEG	NEG	NEG	NEG	NEG	NEG	NEG	NEG	NEG	NEG	NEG	NEG	POS	POS	POS	NEG	NEG	NEG	NEG	NEG	NEG	NEG	NEG	NEG	NEG	NEG	NEG	NEG	NEG	NEG	NEG	NEG
Iasi-17-90	BSI	NEG		NEG	NEG	NEG	NEG	NEG	NEG	NEG	NEG	NEG	NEG	NEG	NEG	NEG	NEG	NEG	NEG	NEG	NEG	NEG	NEG	NEG	NEG	NEG	NEG	POS	POS	POS	NEG	NEG	NEG	NEG	NEG	NEG	NEG	NEG	NEG	NEG	NEG	NEG	NEG	NEG	NEG	NEG	NEG
Iasi-21-105	BSI	NEG		NEG	NEG	NEG	NEG	NEG	NEG	NEG	NEG	NEG	NEG	NEG	NEG	NEG	NEG	NEG	NEG	NEG	NEG	NEG	NEG	NEG	NEG	NEG	NEG	POS	POS	AMB	NEG	NEG	NEG	NEG	NEG	NEG	NEG	NEG	NEG	NEG	NEG	NEG	NEG	NEG	NEG	NEG	NEG
Iasi-22-109	BSI	NEG		NEG	NEG	NEG	NEG	NEG	NEG	NEG	NEG	NEG	NEG	NEG	NEG	NEG	NEG	NEG	NEG	NEG	NEG	NEG	NEG	NEG	NEG	NEG	NEG	POS	POS	POS	NEG	NEG	NEG	NEG	NEG	NEG	NEG	NEG	NEG	AMB	NEG	NEG	NEG	NEG	NEG	NEG	NEG
Iasi-23-110	BSI	NEG		NEG	NEG	NEG	NEG	NEG	NEG	NEG	NEG	NEG	NEG	NEG	NEG	NEG	NEG	NEG	NEG	NEG	NEG	NEG	NEG	NEG	NEG	NEG	NEG	POS	POS	POS	NEG	NEG	NEG	NEG	NEG	NEG	NEG	NEG	NEG	NEG	NEG	NEG	NEG	NEG	NEG	NEG	NEG
Iasi-24-136	BSI	NEG		NEG	NEG	NEG	NEG	NEG	NEG	NEG	NEG	NEG	NEG	NEG	NEG	NEG	NEG	NEG	NEG	NEG	NEG	NEG	NEG	NEG	NEG	NEG	NEG	POS	POS	POS	NEG	NEG	NEG	NEG	NEG	NEG	NEG	NEG	NEG	NEG	NEG	NEG	NEG	NEG	NEG	NEG	NEG
Iasi-34-224	BSI	NEG		NEG	NEG	NEG	NEG	NEG	NEG	NEG	NEG	NEG	NEG	NEG	NEG	NEG	NEG	NEG	NEG	NEG	NEG	NEG	NEG	NEG	NEG	NEG	NEG	POS	POS	POS	NEG	NEG	NEG	NEG	NEG	NEG	NEG	NEG	NEG	NEG	NEG	NEG	NEG	NEG	NEG	NEG	NEG
Iasi-39-297	BSI	NEG		NEG	NEG	NEG	NEG	NEG	NEG	NEG	NEG	NEG	NEG	NEG	NEG	NEG	NEG	NEG	NEG	NEG	NEG	NEG	NEG	NEG	NEG	NEG	NEG	POS	POS	POS	NEG	NEG	NEG	NEG	NEG	NEG	NEG	NEG	NEG	AMB	NEG	NEG	NEG	NEG	NEG	NEG	NEG
Iasi-50-431	BSI	NEG		NEG	NEG	NEG	NEG	NEG	NEG	NEG	NEG	NEG	NEG	NEG	NEG	NEG	NEG	NEG	NEG	NEG	NEG	NEG	NEG	NEG	NEG	NEG	NEG	POS	POS	POS	NEG	NEG	NEG	NEG	NEG	NEG	NEG	NEG	NEG	NEG	NEG	NEG	NEG	NEG	NEG	NEG	NEG
Iasi-51-432	BSI	NEG		NEG	NEG	NEG	NEG	NEG	NEG	NEG	NEG	NEG	NEG	NEG	NEG	NEG	NEG	NEG	NEG	NEG	NEG	NEG	NEG	NEG	NEG	NEG	NEG	POS	POS	POS	NEG	NEG	NEG	NEG	NEG	NEG	NEG	NEG	NEG	NEG	NEG	NEG	NEG	NEG	NEG	NEG	NEG
Iasi-08-76	SSTI	NEG		NEG	NEG	NEG	NEG	NEG	NEG	NEG	NEG	NEG	NEG	NEG	NEG	NEG	NEG	NEG	NEG	NEG	NEG	NEG	NEG	NEG	NEG	NEG	NEG	POS	POS	POS	NEG	NEG	NEG	NEG	NEG	NEG	NEG	NEG	NEG	NEG	NEG	NEG	NEG	NEG	NEG	NEG	NEG
Iasi-30-350	SSTI	NEG		NEG	NEG	NEG	NEG	NEG	NEG	NEG	NEG	NEG	NEG	NEG	NEG	NEG	NEG	NEG	NEG	NEG	NEG	NEG	NEG	NEG	NEG	NEG	NEG	POS	POS	POS	NEG	NEG	NEG	NEG	NEG	NEG	NEG	NEG	NEG	NEG	NEG	NEG	NEG	NEG	NEG	NEG	NEG
Iasi-33-319	SSTI	NEG		NEG	NEG	NEG	NEG	NEG	NEG	NEG	NEG	NEG	NEG	NEG	NEG	NEG	NEG	NEG	NEG	NEG	NEG	NEG	NEG	NEG	NEG	NEG	NEG	POS	POS	POS	NEG	NEG	NEG	NEG	NEG	NEG	NEG	NEG	NEG	NEG	NEG	NEG	NEG	NEG	NEG	NEG	NEG
Iasi-40-383	SSTI	NEG		NEG	NEG	NEG	NEG	NEG	NEG	NEG	NEG	NEG	NEG	NEG	NEG	NEG	NEG	NEG	NEG	NEG	NEG	NEG	NEG	NEG	NEG	NEG	NEG	POS	POS	POS	NEG	NEG	NEG	NEG	NEG	NEG	NEG	NEG	NEG	NEG	NEG	NEG	NEG	NEG	NEG	NEG	NEG
Iasi-49-423	SSTI	NEG		NEG	NEG	NEG	NEG	NEG	NEG	NEG	NEG	NEG	NEG	NEG	NEG	NEG	NEG	NEG	NEG	NEG	NEG	NEG	NEG	NEG	NEG	NEG	NEG	POS	POS	POS	NEG	NEG	NEG	NEG	NEG	NEG	NEG	NEG	NEG	NEG	NEG	NEG	NEG	NEG	NEG	NEG	NEG
Iasi-16-S31	Carrier	NEG		NEG	NEG	NEG	NEG	NEG	NEG	NEG	NEG	NEG	NEG	NEG	NEG	NEG	NEG	NEG	NEG	NEG	NEG	NEG	NEG	NEG	NEG	NEG	NEG	POS	POS	POS	NEG	NEG	NEG	NEG	NEG	NEG	NEG	NEG	NEG	NEG	NEG	NEG	NEG	NEG	NEG	NEG	NEG
Iasi-24-P9	Carrier	NEG		NEG	NEG	NEG	NEG	NEG	NEG	NEG	NEG	NEG	NEG	NEG	NEG	NEG	NEG	NEG	NEG	NEG	NEG	NEG	NEG	NEG	NEG	NEG	NEG	POS	POS	POS	NEG	NEG	NEG	NEG	NEG	NEG	NEG	NEG	NEG	AMB	NEG	NEG	NEG	NEG	NEG	NEG	NEG
Iasi-27-P12	Carrier	NEG		NEG	NEG	NEG	NEG	NEG	NEG	NEG	NEG	NEG	NEG	NEG	NEG	NEG	NEG	NEG	NEG	NEG	NEG	NEG	NEG	NEG	NEG	NEG	NEG	POS	POS	POS	NEG	NEG	NEG	NEG	NEG	NEG	NEG	NEG	NEG	NEG	NEG	NEG	NEG	NEG	NEG	NEG	NEG
Iasi-38-P27	Carrier	NEG		NEG	NEG	NEG	NEG	NEG	NEG	NEG	NEG	NEG	NEG	NEG	NEG	NEG	NEG	NEG	NEG	NEG	NEG	NEG	NEG	NEG	NEG	NEG	NEG	POS	POS	POS	NEG	NEG	NEG	NEG	NEG	NEG	NEG	NEG	NEG	AMB	NEG	NEG	NEG	NEG	NEG	NEG	NEG
Iasi-42-G511	Carrier	NEG		NEG	NEG	NEG	NEG	NEG	NEG	NEG	NEG	NEG	NEG	NEG	NEG	NEG	NEG	NEG	NEG	NEG	NEG	NEG	NEG	NEG	NEG	NEG	NEG	POS	POS	POS	NEG	NEG	NEG	NEG	NEG	NEG	NEG	NEG	NEG	NEG	NEG	NEG	NEG	NEG	NEG	NEG	NEG
Iasi-46-3N	Carrier	NEG		NEG	NEG	NEG	NEG	NEG	NEG	NEG	NEG	NEG	NEG	NEG	NEG	NEG	NEG	NEG	NEG	NEG	NEG	NEG	NEG	NEG	NEG	NEG	NEG	POS	POS	POS	NEG	NEG	NEG	NEG	NEG	NEG	NEG	NEG	NEG	AMB	NEG	NEG	NEG	NEG	NEG	NEG	NEG
Iasi-47-42O	Carrier	NEG		NEG	NEG	NEG	NEG	NEG	NEG	NEG	NEG	NEG	NEG	NEG	NEG	NEG	NEG	NEG	NEG	NEG	NEG	NEG	NEG	NEG	NEG	NEG	NEG	POS	POS	POS	NEG	NEG	NEG	NEG	NEG	NEG	NEG	NEG	NEG	NEG	NEG	NEG	NEG	NEG	NEG	NEG	NEG
Iasi-48-36N	Carrier	NEG		NEG	NEG	NEG	NEG	NEG	NEG	NEG	NEG	NEG	NEG	NEG	NEG	NEG	NEG	NEG	NEG	NEG	NEG	NEG	NEG	NEG	NEG	NEG	NEG	POS	POS	POS	NEG	NEG	NEG	NEG	NEG	NEG	NEG	NEG	NEG	NEG	NEG	NEG	NEG	NEG	NEG	NEG	NEG
Iasi-49-T3	Carrier	NEG		NEG	NEG	NEG	NEG	NEG	NEG	NEG	NEG	NEG	NEG	NEG	NEG	NEG	NEG	NEG	NEG	NEG	NEG	NEG	NEG	NEG	NEG	NEG	NEG	POS	POS	POS	NEG	NEG	POS	NEG	NEG	NEG	NEG	NEG	NEG	NEG	NEG	NEG	NEG	NEG	NEG	NEG	NEG
Iasi-50-T2	Carrier	NEG		NEG	NEG	NEG	NEG	NEG	NEG	NEG	NEG	NEG	NEG	NEG	NEG	NEG	NEG	NEG	NEG	NEG	NEG	NEG	NEG	NEG	NEG	NEG	NEG	POS	POS	POS	NEG	NEG	POS	NEG	NEG	NEG	NEG	NEG	NEG	NEG	NEG	NEG	NEG	NEG	NEG	NEG	NEG
Iasi-22-251	BSI	POS		POS	POS	NEG	NEG	NEG	POS	POS	POS	NEG	NEG	NEG	NEG	NEG	NEG	NEG	NEG	NEG	NEG	NEG	NEG	NEG	NEG	NEG	NEG	POS	POS	POS	NEG	NEG	NEG	NEG	NEG	NEG	NEG	NEG	NEG	NEG	NEG	NEG	NEG	NEG	NEG	NEG	NEG
Iasi-27-158	BSI	NEG		NEG	NEG	NEG	NEG	NEG	NEG	NEG	NEG	NEG	NEG	NEG	NEG	NEG	NEG	NEG	NEG	NEG	NEG	NEG	NEG	NEG	NEG	NEG	NEG	POS	POS	POS	NEG	NEG	NEG	NEG	NEG	NEG	NEG	NEG	NEG	POS	NEG	NEG	NEG	NEG	NEG	NEG	AMB
Iasi-08-70	BSI	NEG		NEG	NEG	NEG	NEG	NEG	NEG	NEG	NEG	NEG	NEG	NEG	NEG	NEG	NEG	NEG	NEG	NEG	NEG	NEG	NEG	NEG	NEG	NEG	NEG	POS	POS	POS	NEG	NEG	NEG	NEG	NEG	NEG	NEG	NEG	NEG	NEG	NEG	NEG	POS	NEG	NEG	NEG	NEG
Iasi-18-93	BSI	NEG		NEG	NEG	NEG	NEG	NEG	NEG	NEG	NEG	NEG	NEG	NEG	NEG	NEG	NEG	NEG	NEG	NEG	NEG	NEG	NEG	NEG	NEG	NEG	NEG	POS	POS	POS	NEG	NEG	NEG	NEG	NEG	NEG	NEG	NEG	NEG	NEG	NEG	NEG	NEG	NEG	NEG	NEG	NEG
Iasi-28-P14	Carrier	NEG		NEG	NEG	NEG	NEG	NEG	NEG	NEG	NEG	NEG	NEG	NEG	NEG	NEG	NEG	NEG	NEG	NEG	NEG	NEG	NEG	NEG	NEG	NEG	NEG	POS	POS	POS	NEG	NEG	NEG	NEG	NEG	NEG	NEG	NEG	NEG	NEG	NEG	NEG	NEG	NEG	NEG	NEG	NEG
Iasi-29-P15	Carrier	NEG		NEG	NEG	NEG	NEG	NEG	NEG	NEG	NEG	NEG	NEG	NEG	NEG	NEG	NEG	NEG	NEG	NEG	NEG	NEG	NEG	NEG	NEG	NEG	NEG	POS	POS	POS	NEG	NEG	NEG	NEG	NEG	NEG	NEG	NEG	NEG	AMB	NEG	NEG	NEG	NEG	NEG	NEG	AMB
Iasi-03-19	BSI	NEG		NEG	NEG	NEG	NEG	NEG	NEG	NEG	NEG	NEG	NEG	NEG	NEG	NEG	NEG	NEG	NEG	NEG	NEG	NEG	NEG	NEG	NEG	NEG	NEG	POS	POS	POS	NEG	NEG	NEG	NEG	NEG	NEG	NEG	NEG	NEG	NEG	NEG	NEG	NEG	NEG	NEG	NEG	NEG
Iasi-11-77	BSI	NEG		NEG	NEG	NEG	NEG	NEG	NEG	NEG	NEG	NEG	NEG	NEG	NEG	NEG	NEG	NEG	NEG	NEG	NEG	NEG	NEG	NEG	NEG	NEG	NEG	POS	POS	POS	NEG	NEG	NEG	NEG	NEG	NEG	NEG	NEG	NEG	NEG	NEG	NEG	NEG	NEG	NEG	NEG	NEG
Iasi-13-82	BSI	NEG		NEG	NEG	NEG	NEG	NEG	NEG	NEG	NEG	NEG	NEG	NEG	NEG	NEG	NEG	NEG	NEG	NEG	NEG	NEG	NEG	NEG	NEG	NEG	NEG	NEG	NEG	NEG	NEG	NEG	POS	NEG	NEG	NEG	NEG	NEG	NEG	NEG	NEG	NEG	NEG	NEG	NEG	NEG	NEG
Iasi-15-86	BSI	NEG		NEG	NEG	NEG	NEG	NEG	NEG	NEG	NEG	NEG	NEG	NEG	NEG	NEG	NEG	NEG	NEG	NEG	NEG	NEG	NEG	NEG	NEG	NEG	NEG	POS	POS	POS	NEG	NEG	NEG	NEG	NEG	NEG	NEG	NEG	NEG	NEG	NEG	NEG	NEG	NEG	NEG	NEG	NEG
Iasi-20-103	BSI	NEG		NEG	NEG	NEG	NEG	NEG	NEG	NEG	NEG	NEG	NEG	NEG	NEG	NEG	NEG	NEG	NEG	NEG	NEG	NEG	NEG	NEG	NEG	NEG	NEG	POS	POS	POS	NEG	NEG	NEG	NEG	NEG	NEG	NEG	NEG	NEG	NEG	NEG	NEG	NEG	NEG	NEG	NEG	NEG
Iasi-33-222	BSI	NEG		NEG	NEG	NEG	NEG	NEG	NEG	NEG	NEG	NEG	NEG	NEG	NEG	NEG	NEG	NEG	NEG	NEG	NEG	NEG	NEG	NEG	NEG	NEG	NEG	POS	POS	POS	NEG	NEG	NEG	NEG	NEG	NEG	NEG	NEG	NEG	NEG	NEG	NEG	NEG	NEG	NEG	NEG	NEG
Iasi-44-360	BSI	NEG	NEG	NEG	NEG	NEG	NEG	NEG	NEG	NEG	NEG	NEG	NEG	NEG	NEG	NEG	NEG	NEG	NEG	NEG	NEG	NEG	NEG	NEG	NEG	NEG	NEG	POS	POS	POS	NEG	NEG	NEG	NEG	NEG	NEG	NEG	NEG	NEG	NEG	NEG	NEG	NEG	NEG	NEG	NEG	NEG
Iasi-47-399	BSI	NEG		NEG	NEG	NEG	NEG	NEG	NEG	NEG	NEG	NEG	NEG	NEG	NEG	NEG	NEG	NEG	NEG	NEG	NEG	NEG	NEG	NEG	NEG	NEG	NEG	POS	POS	POS	NEG	NEG	NEG	NEG	NEG	NEG	NEG	NEG	NEG	NEG	NEG	NEG	NEG	NEG	NEG	NEG	NEG
Iasi-34-368	SSTI	NEG		NEG	NEG	NEG	NEG	NEG	NEG	NEG	NEG	NEG	NEG	NEG	NEG	NEG	NEG	NEG	NEG	NEG	NEG	NEG	NEG	NEG	NEG	NEG	NEG	NEG	NEG	NEG	NEG	NEG	NEG	NEG	NEG	NEG	NEG	NEG	NEG	NEG	NEG	NEG	NEG	NEG	NEG	NEG	NEG
Iasi-36-371	SSTI	NEG		NEG	NEG	NEG	NEG	NEG	NEG	NEG	NEG	NEG	NEG	NEG	NEG	NEG	NEG	NEG	NEG	NEG	NEG	NEG	NEG	NEG	NEG	NEG	NEG	POS	POS	POS	NEG	NEG	NEG	NEG	NEG	NEG	NEG	NEG	NEG	NEG	NEG	NEG	NEG	NEG	NEG	NEG	NEG
Iasi-04-S12	Carrier	NEG		NEG	NEG	NEG	AMB	NEG	NEG	NEG	NEG	NEG	NEG	NEG	NEG	NEG	NEG	NEG	NEG	NEG	NEG	NEG	NEG	NEG	NEG	NEG	NEG	POS	POS	POS	NEG	NEG	NEG	NEG	NEG	NEG	NEG	NEG	NEG	NEG	NEG	NEG	NEG	POS	NEG	NEG	NEG
Iasi-06-S15	Carrier	NEG		NEG	NEG	NEG	NEG	NEG	NEG	NEG	NEG	NEG	NEG	NEG	NEG	NEG	NEG	NEG	NEG	NEG	NEG	NEG	NEG	NEG	NEG	NEG	NEG	NEG	NEG	NEG	NEG	NEG	NEG	NEG	NEG	NEG	NEG	NEG	NEG	NEG	NEG	NEG	NEG	NEG	NEG	NEG	NEG
Iasi-07-S16	Carrier	NEG		NEG	NEG	NEG	NEG	NEG	NEG	NEG	NEG	NEG	NEG	NEG	NEG	NEG	NEG	NEG	NEG	NEG	NEG	NEG	NEG	NEG	NEG	NEG	NEG	NEG	NEG	NEG	NEG	NEG	NEG	NEG	NEG	NEG	NEG	NEG	NEG	NEG	NEG	NEG	NEG	NEG	NEG	NEG	NEG
Iasi-11-S24	Carrier	NEG		NEG	NEG	NEG	NEG	NEG	NEG	NEG	NEG	NEG	NEG	NEG	NEG	NEG	NEG	NEG	NEG	NEG	NEG	NEG	NEG	NEG	NEG	NEG	NEG	POS	POS	POS	NEG	NEG	NEG	NEG	NEG	NEG	NEG	NEG	NEG	AMB	NEG	NEG	NEG	NEG	NEG	NEG	NEG
Iasi-22-P4	Carrier	NEG		NEG	NEG	NEG	NEG	NEG	NEG	NEG	NEG	NEG	NEG	NEG	NEG	NEG	NEG	NEG	NEG	NEG	NEG	NEG	NEG	NEG	NEG	NEG	NEG	POS	POS	POS	NEG	NEG	NEG	NEG	NEG	NEG	NEG	NEG	NEG	NEG	NEG	NEG	NEG	NEG	NEG	NEG	NEG
Iasi-34-P23	Carrier	NEG		NEG	NEG	NEG	NEG	NEG	NEG	NEG	NEG	NEG	NEG	NEG	NEG	NEG	NEG	NEG	NEG	NEG	NEG	NEG	NEG	NEG	NEG	NEG	NEG	POS	POS	POS	NEG	NEG	NEG	NEG	NEG	NEG	NEG	NEG	NEG	NEG	NEG	NEG	NEG	NEG	NEG	NEG	NEG
Iasi-37-P26	Carrier	NEG		NEG	NEG	NEG	AMB	NEG	NEG	NEG	NEG	NEG	NEG	NEG	NEG	NEG	NEG	NEG	NEG	NEG	NEG	NEG	NEG	NEG	NEG	NEG	NEG	POS	POS	POS	NEG	NEG	NEG	NEG	NEG	NEG	NEG	NEG	NEG	AMB	NEG	NEG	NEG	NEG	NEG	NEG	NEG
Iasi-30-196	BSI	NEG		NEG	AMB	NEG	NEG	NEG	NEG	NEG	NEG	NEG	NEG	NEG	NEG	NEG	NEG	NEG	NEG	NEG	NEG	NEG	NEG	NEG	NEG	NEG	NEG	POS	POS	POS	NEG	NEG	NEG	NEG	NEG	NEG	NEG	NEG	NEG	AMB	NEG	NEG	NEG	NEG	NEG	NEG	AMB
Iasi-27-298	BSI	POS		POS	POS	NEG	NEG	NEG	AMB	POS	POS	NEG	NEG	NEG	NEG	NEG	NEG	NEG	NEG	NEG	NEG	NEG	NEG	NEG	NEG	NEG	NEG	POS	POS	POS	NEG	NEG	NEG	NEG	NEG	NEG	NEG	NEG	NEG	NEG	NEG	NEG	NEG	NEG	POS	POS	NEG
Iasi-37-363	BSI	POS		POS	POS	NEG	NEG	NEG	POS	POS	POS	NEG	NEG	NEG	NEG	NEG	NEG	NEG	NEG	NEG	NEG	NEG	NEG	NEG	NEG	NEG	NEG	POS	POS	POS	NEG	NEG	NEG	NEG	NEG	NEG	NEG	NEG	NEG	NEG	NEG	NEG	NEG	NEG	POS	POS	NEG
Iasi-38-372	BSI	POS		POS	POS	NEG	NEG	NEG	POS	POS	POS	NEG	NEG	NEG	NEG	NEG	NEG	NEG	NEG	NEG	NEG	NEG	NEG	NEG	NEG	NEG	NEG	POS	POS	POS	NEG	NEG	NEG	NEG	NEG	NEG	NEG	NEG	NEG	NEG	NEG	NEG	NEG	NEG	POS	POS	NEG
Iasi-39-374	BSI	POS		POS	POS	NEG	NEG	NEG	POS	POS	POS	NEG	NEG	NEG	NEG	NEG	NEG	NEG	NEG	NEG	NEG	NEG	NEG	NEG	NEG	NEG	NEG	POS	POS	POS	NEG	NEG	NEG	NEG	NEG	NEG	NEG	NEG	NEG	NEG	NEG	NEG	NEG	NEG	POS	POS	NEG
Iasi-28-299	SSTI	POS		POS	POS	NEG	NEG	NEG	POS	POS	POS	NEG	NEG	NEG	NEG	NEG	NEG	NEG	NEG	NEG	NEG	NEG	NEG	NEG	NEG	NEG	NEG	POS	POS	POS	NEG	NEG	NEG	NEG	NEG	NEG	NEG	NEG	NEG	NEG	NEG	NEG	NEG	NEG	POS	POS	NEG
Iasi-07-65	BSI	NEG		NEG	NEG	NEG	NEG	NEG	NEG	NEG	NEG	NEG	NEG	NEG	NEG	NEG	NEG	NEG	NEG	NEG	NEG	NEG	NEG	NEG	NEG	NEG	NEG	POS	POS	POS	POS	NEG	NEG	NEG	NEG	NEG	NEG	NEG	NEG	NEG	NEG	NEG	NEG	NEG	NEG	NEG	NEG
Iasi-19-99	BSI	NEG		NEG	NEG	NEG	NEG	NEG	NEG	NEG	NEG	NEG	NEG	NEG	NEG	NEG	NEG	NEG	NEG	NEG	NEG	NEG	NEG	NEG	NEG	NEG	NEG	POS	POS	POS	NEG	NEG	NEG	NEG	NEG	NEG	NEG	NEG	NEG	NEG	NEG	NEG	NEG	NEG	NEG	NEG	NEG
Iasi-25-217	SSTI	NEG		NEG	NEG	NEG	AMB	NEG	NEG	NEG	NEG	NEG	NEG	NEG	NEG	NEG	NEG	NEG	NEG	NEG	NEG	NEG	NEG	NEG	NEG	NEG	NEG	NEG	NEG	NEG	NEG	NEG	NEG	NEG	NEG	NEG	NEG	NEG	NEG	NEG	NEG	NEG	NEG	NEG	NEG	NEG	NEG
Iasi-23-P5	Carrier	NEG		NEG	NEG	NEG	NEG	NEG	NEG	NEG	NEG	NEG	NEG	NEG	NEG	NEG	NEG	NEG	NEG	NEG	NEG	NEG	NEG	NEG	NEG	NEG	NEG	POS	POS	POS	NEG	NEG	NEG	NEG	NEG	NEG	NEG	NEG	NEG	NEG	NEG	NEG	NEG	NEG	NEG	NEG	NEG
Iasi-43-G513	Carrier	NEG		NEG	NEG	NEG	NEG	NEG	NEG	NEG	NEG	NEG	NEG	NEG	NEG	NEG	NEG	NEG	NEG	NEG	NEG	NEG	NEG	NEG	NEG	NEG	NEG	POS	POS	POS	NEG	NEG	NEG	NEG	NEG	NEG	NEG	NEG	NEG	NEG	NEG	NEG	NEG	NEG	NEG	NEG	NEG
Iasi-44-G514	Carrier	NEG		NEG	NEG	NEG	NEG	NEG	NEG	NEG	NEG	NEG	NEG	NEG	NEG	NEG	NEG	NEG	NEG	NEG	NEG	NEG	NEG	NEG	NEG	NEG	NEG	POS	POS	POS	NEG	NEG	NEG	NEG	NEG	NEG	NEG	NEG	NEG	AMB	NEG	NEG	NEG	NEG	NEG	NEG	NEG
Iasi-28-172	BSI	NEG		NEG	NEG	NEG	NEG	NEG	NEG	NEG	NEG	NEG	NEG	NEG	NEG	NEG	NEG	NEG	NEG	NEG	NEG	NEG	NEG	NEG	NEG	NEG	NEG	POS	POS	POS	NEG	NEG	NEG	NEG	NEG	NEG	NEG	NEG	NEG	AMB	NEG	NEG	NEG	NEG	NEG	NEG	NEG
Iasi-42-358	BSI	NEG		NEG	NEG	NEG	NEG	NEG	NEG	NEG	NEG	NEG	NEG	NEG	NEG	NEG	NEG	NEG	NEG	NEG	NEG	NEG	NEG	NEG	NEG	NEG	NEG	POS	POS	POS	NEG	NEG	NEG	NEG	NEG	NEG	NEG	NEG	NEG	AMB	NEG	NEG	NEG	NEG	NEG	NEG	AMB
Iasi-48-403	BSI	NEG		NEG	NEG	NEG	NEG	NEG	NEG	NEG	NEG	NEG	NEG	NEG	NEG	NEG	NEG	NEG	NEG	NEG	NEG	NEG	NEG	NEG	NEG	NEG	NEG	POS	POS	POS	NEG	NEG	NEG	NEG	NEG	NEG	NEG	NEG	NEG	NEG	NEG	NEG	NEG	NEG	NEG	NEG	NEG
Iasi-26-153	BSI	NEG	NEG	NEG	NEG	NEG	NEG	NEG	NEG	NEG	NEG	NEG	NEG	NEG	NEG	NEG	NEG	NEG	NEG	NEG	NEG	NEG	NEG	NEG	NEG	NEG	NEG	POS	POS	POS	NEG	NEG	NEG	NEG	NEG	NEG	NEG	NEG	NEG	NEG	NEG	NEG	NEG	NEG	NEG	NEG	NEG
Iasi-29-190	BSI	NEG		NEG	NEG	NEG	NEG	NEG	NEG	NEG	NEG	NEG	NEG	NEG	NEG	NEG	NEG	NEG	NEG	NEG	NEG	NEG	NEG	NEG	NEG	NEG	NEG	POS	POS	POS	NEG	NEG	NEG	NEG	NEG	NEG	NEG	NEG	NEG	NEG	NEG	NEG	NEG	NEG	NEG	NEG	NEG
Iasi-31-201	BSI	NEG	NEG	NEG	NEG	NEG	NEG	NEG	NEG	NEG	NEG	NEG	NEG	NEG	NEG	NEG	NEG	NEG	NEG	NEG	NEG	NEG	NEG	NEG	NEG	NEG	NEG	POS	POS	POS	NEG	NEG	NEG	NEG	NEG	NEG	NEG	NEG	NEG	NEG	NEG	NEG	NEG	NEG	NEG	NEG	NEG
Iasi-37-289	BSI	NEG		NEG	NEG	NEG	NEG	NEG	NEG	NEG	NEG	NEG	NEG	NEG	NEG	NEG	NEG	NEG	NEG	NEG	NEG	NEG	NEG	NEG	NEG	NEG	NEG	POS	POS	POS	NEG	NEG	NEG	NEG	NEG	NEG	NEG	NEG	NEG	AMB	NEG	NEG	NEG	NEG	NEG	NEG	NEG
Iasi-41-334	BSI	NEG		NEG	AMB	NEG	NEG	NEG	NEG	NEG	NEG	NEG	NEG	NEG	NEG	NEG	NEG	NEG	NEG	NEG	NEG	NEG	NEG	NEG	NEG	NEG	NEG	POS	POS	POS	NEG	NEG	NEG	NEG	NEG	NEG	NEG	NEG	NEG	POS	NEG	NEG	NEG	NEG	NEG	NEG	AMB
Iasi-52-433	BSI	NEG	NEG	NEG	NEG	NEG	NEG	NEG	NEG	NEG	NEG	NEG	NEG	NEG	NEG	NEG	NEG	NEG	NEG	NEG	NEG	NEG	NEG	NEG	NEG	NEG	NEG	POS	POS	POS	NEG	NEG	NEG	NEG	NEG	NEG	NEG	NEG	NEG	NEG	NEG	NEG	NEG	NEG	NEG	NEG	NEG
Iasi-07-66	SSTI	NEG		NEG	NEG	NEG	NEG	NEG	NEG	NEG	NEG	NEG	NEG	NEG	NEG	NEG	NEG	NEG	NEG	NEG	NEG	NEG	NEG	NEG	NEG	NEG	NEG	POS	POS	POS	NEG	NEG	NEG	NEG	NEG	NEG	NEG	NEG	NEG	NEG	NEG	NEG	NEG	NEG	NEG	NEG	NEG
Iasi-23-211	SSTI	NEG		NEG	NEG	NEG	NEG	NEG	NEG	NEG	NEG	NEG	NEG	NEG	NEG	NEG	NEG	NEG	NEG	NEG	NEG	NEG	NEG	NEG	NEG	NEG	NEG	POS	POS	POS	NEG	NEG	NEG	NEG	NEG	NEG	NEG	NEG	NEG	NEG	NEG	NEG	NEG	NEG	NEG	NEG	NEG
Iasi-43-401	SSTI	NEG		NEG	NEG	NEG	NEG	NEG	NEG	NEG	NEG	NEG	NEG	NEG	NEG	NEG	NEG	NEG	NEG	NEG	NEG	NEG	NEG	NEG	NEG	NEG	NEG	POS	POS	POS	NEG	NEG	NEG	NEG	NEG	NEG	NEG	NEG	NEG	NEG	NEG	NEG	NEG	NEG	NEG	NEG	NEG
Iasi-47-418	SSTI	NEG		NEG	NEG	NEG	NEG	NEG	NEG	NEG	NEG	NEG	NEG	NEG	NEG	NEG	NEG	NEG	NEG	NEG	NEG	NEG	NEG	NEG	NEG	NEG	NEG	POS	POS	POS	NEG	NEG	NEG	NEG	NEG	NEG	NEG	NEG	NEG	NEG	NEG	NEG	NEG	NEG	NEG	NEG	NEG
Iasi-18-169	SSTI	NEG		NEG	NEG	NEG	NEG	NEG	NEG	NEG	NEG	NEG	NEG	NEG	NEG	NEG	NEG	NEG	NEG	NEG	NEG	NEG	NEG	NEG	NEG	NEG	NEG	NEG	NEG	NEG	NEG	NEG	NEG	NEG	NEG	NEG	NEG	NEG	NEG	NEG	NEG	NEG	NEG	NEG	NEG	NEG	NEG
Iasi-18-S34	Carrier	NEG		NEG	NEG	NEG	NEG	NEG	NEG	NEG	NEG	NEG	NEG	NEG	NEG	NEG	NEG	NEG	NEG	NEG	NEG	NEG	NEG	NEG	NEG	NEG	NEG	POS	POS	POS	NEG	NEG	NEG	NEG	NEG	NEG	NEG	NEG	NEG	AMB	NEG	NEG	NEG	NEG	NEG	NEG	NEG
Iasi-36-276	BSI	NEG		NEG	NEG	NEG	NEG	NEG	NEG	NEG	NEG	NEG	NEG	NEG	NEG	NEG	NEG	NEG	NEG	NEG	NEG	NEG	NEG	NEG	NEG	NEG	NEG	POS	POS	POS	NEG	NEG	NEG	NEG	NEG	NEG	NEG	NEG	NEG	NEG	NEG	NEG	NEG	NEG	NEG	NEG	NEG
Iasi-151	BSI	POS		NEG	POS	NEG	POS	NEG	NEG	NEG	NEG	NEG	NEG	NEG	NEG	NEG	NEG	NEG	NEG	NEG	NEG	NEG	NEG	POS	POS	NEG	NEG	POS	POS	POS	NEG	NEG	POS	NEG	NEG	NEG	NEG	NEG	NEG	AMB	NEG	NEG	NEG	POS	NEG	AMB	NEG
Iasi-02-11	BSI	NEG	NEG	NEG	NEG	NEG	NEG	NEG	NEG	NEG	NEG	NEG	NEG	NEG	NEG	NEG	NEG	NEG	NEG	NEG	NEG	NEG	NEG	NEG	NEG	NEG	NEG	POS	POS	POS	NEG	NEG	NEG	NEG	NEG	NEG	NEG	NEG	NEG	NEG	NEG	NEG	NEG	NEG	NEG	NEG	NEG

Isolate	Group	RESISTANCE : MISCELLANEOUS GENES														RESISTANCE : EFFLUX SYSTEMS								RESISTANCE : GLYCOPEPTIDES			VIRULENCE : TOX.SCHOCK.TOXIN			VIRULENCE : ENTEROTOXINS																
		fusidic acid resistence	hypothetical protein associated with fusidic acid resistance	mupirocin resistence protein	tetrazyklin-resistance		chloramphenicol acetyltransferase					23S rRNA methyltransferase	chloramphenicol/florfenicol exporter	metallothiol transferase		quaternary ammonium compound resistance protein A	quaternary ammonium compound resistance protein C						Transport-/Effluxprotein	vancomycin resistance gene	vancomycin resistance gene from enterococci and Clostridium	teicoplanin resistance gene from enterococci	toxic shock syndrome toxin 1			enterotoxin A	enterotoxin A, allele from strain 320E	enterotoxin A, allele from strain N315 = enterotoxin P	enterotoxin B	enterotoxin C	enterotoxin D	enterotoxin E	enterotoxin G	enterotoxin H	enterotoxin I	enterotoxin J	enterotoxin K	enterotoxin L	enterotoxin M	enterotoxin N		enterotoxin O
		far1	fusC (Q6GD50)	mupA	tet(K)	tet(M)	cat (total)	cat (pC221)	cat (pc223)	cat (pMC524/pC194)	cat (pSBK203R)	cfr	fexA	fosB	fosB (plasmid)	qacA	qacC (total)	qacC (consensus)	qacC (equine)	qacC (SA5)	qacC (Ssap)	qacC (ST94)	sdrM	vanA	vanB	vanZ	tst1 (consensus)	tst1 ("human" allele)	tst1 ("bovine" allele)	sea	sea (320E)	sea (N315) / sep	seb	sec	sed	see	seg	seh	sei	sej	sek	sel	selm	seln (consensus)	seln (other than RF122)	selo
Iasi-01-4	BSI	NEG	NEG	NEG	POS	NEG	NEG	NEG	NEG	NEG	NEG	NEG	NEG	NEG	NEG	NEG	NEG	NEG	NEG	NEG	NEG	NEG	POS	NEG	NEG	NEG	NEG	NEG	NEG	NEG	NEG	NEG	NEG	NEG	NEG	NEG	NEG	POS	NEG	NEG	NEG	NEG	NEG	NEG	NEG	NEG
Iasi-05-32	BSI	NEG	NEG	NEG	POS	NEG	NEG	NEG	NEG	NEG	NEG	NEG	NEG	NEG	NEG	NEG	NEG	NEG	NEG	NEG	NEG	NEG	POS	NEG	NEG	NEG	NEG	NEG	NEG	NEG	NEG	NEG	NEG	NEG	NEG	NEG	NEG	POS	NEG	NEG	NEG	NEG	NEG	NEG	NEG	NEG
Iasi-16-88	BSI	NEG	NEG	NEG	NEG	NEG	NEG	NEG	NEG	NEG	NEG	NEG	NEG	NEG	NEG	NEG	NEG	NEG	NEG	NEG	NEG	NEG	POS	NEG	NEG	NEG	NEG	NEG	NEG	NEG	NEG	NEG	NEG	NEG	NEG	NEG	NEG	POS	NEG	NEG	NEG	NEG	NEG	NEG	NEG	NEG
Iasi-25-141	BSI	NEG	NEG	NEG	POS	NEG	NEG	NEG	NEG	NEG	NEG	NEG	NEG	AMB	NEG	NEG	NEG	NEG	NEG	NEG	NEG	NEG	POS	NEG	NEG	NEG	NEG	NEG	NEG	NEG	NEG	NEG	NEG	NEG	NEG	NEG	NEG	POS	NEG	NEG	NEG	NEG	NEG	NEG	NEG	NEG
Iasi-32-215	BSI	NEG	NEG	NEG	POS	NEG	NEG	NEG	NEG	NEG	NEG	NEG	NEG	AMB	NEG	NEG	NEG	NEG	NEG	NEG	NEG	NEG	POS	NEG	NEG	NEG	AMB	AMB	NEG	NEG	NEG	NEG	NEG	NEG	NEG	NEG	NEG	POS	NEG	NEG	NEG	NEG	NEG	NEG	NEG	NEG
Iasi-43-359	BSI	NEG	NEG	NEG	NEG	NEG	NEG	NEG	NEG	NEG	NEG	NEG	NEG	NEG	NEG	NEG	NEG	NEG	NEG	NEG	NEG	NEG	POS	NEG	NEG	NEG	NEG	NEG	NEG	NEG	NEG	NEG	NEG	NEG	NEG	NEG	NEG	POS	NEG	NEG	NEG	NEG	NEG	NEG	NEG	NEG
Iasi-03-27	SSTI	NEG	NEG	NEG	POS	NEG	NEG	NEG	NEG	NEG	NEG	NEG	NEG	NEG	NEG	NEG	NEG	NEG	NEG	NEG	NEG	NEG	POS	NEG	NEG	NEG	NEG	NEG	NEG	NEG	NEG	NEG	NEG	NEG	NEG	NEG	NEG	POS	NEG	NEG	NEG	NEG	NEG	NEG	NEG	NEG
Iasi-13-149	SSTI	NEG	NEG	NEG	POS	NEG	NEG	NEG	NEG	NEG	NEG	NEG	NEG	NEG	NEG	NEG	NEG	NEG	NEG	NEG	NEG	NEG	POS	NEG	NEG	NEG	NEG	NEG	NEG	NEG	NEG	NEG	NEG	NEG	NEG	NEG	NEG	POS	NEG	NEG	NEG	NEG	NEG	NEG	NEG	NEG
Iasi-29-337	SSTI	NEG	NEG	NEG	POS	NEG	POS	POS	NEG	NEG	NEG	NEG	NEG	NEG	NEG	NEG	POS	POS	NEG	NEG	NEG	NEG	POS	NEG	NEG	NEG	NEG	NEG	NEG	NEG	NEG	NEG	NEG	NEG	NEG	NEG	NEG	POS	NEG	NEG	NEG	NEG	NEG	NEG	NEG	NEG
Iasi-31-355	SSTI	NEG	NEG	NEG	POS	NEG	NEG	NEG	NEG	NEG	NEG	NEG	NEG	NEG	NEG	NEG	NEG	NEG	NEG	NEG	NEG	NEG	POS	NEG	NEG	NEG	NEG	NEG	NEG	NEG	NEG	NEG	NEG	NEG	NEG	NEG	NEG	POS	NEG	NEG	NEG	NEG	NEG	NEG	NEG	NEG
Iasi-38-377	SSTI	NEG	NEG	NEG	POS	NEG	NEG	NEG	NEG	NEG	NEG	NEG	NEG	NEG	NEG	NEG	NEG	NEG	NEG	NEG	NEG	NEG	POS	NEG	NEG	NEG	NEG	NEG	NEG	NEG	NEG	NEG	NEG	NEG	NEG	NEG	NEG	POS	NEG	NEG	NEG	NEG	NEG	NEG	NEG	NEG
Iasi-41-388	SSTI	NEG	POS	NEG	POS	NEG	NEG	NEG	NEG	NEG	NEG	NEG	NEG	NEG	NEG	NEG	NEG	NEG	NEG	NEG	NEG	NEG	POS	NEG	NEG	NEG	NEG	NEG	NEG	NEG	NEG	NEG	NEG	NEG	NEG	NEG	NEG	POS	NEG	NEG	NEG	NEG	NEG	NEG	NEG	NEG
Iasi-46-417	SSTI	NEG	NEG	NEG	POS	NEG	NEG	NEG	NEG	NEG	NEG	NEG	NEG	NEG	NEG	NEG	NEG	NEG	NEG	NEG	NEG	NEG	POS	NEG	NEG	NEG	NEG	NEG	NEG	NEG	NEG	NEG	NEG	NEG	NEG	NEG	NEG	POS	NEG	NEG	NEG	NEG	NEG	NEG	NEG	NEG
Iasi-02-S9	Carrier	NEG	NEG	NEG	NEG	NEG	NEG	NEG	NEG	NEG	NEG	NEG	NEG	AMB	NEG	NEG	NEG	NEG	NEG	NEG	NEG	NEG	POS	NEG	NEG	NEG	NEG	NEG	NEG	NEG	NEG	NEG	NEG	NEG	NEG	NEG	NEG	POS	NEG	NEG	NEG	NEG	NEG	NEG	NEG	NEG
Iasi-17-S33	Carrier	NEG	NEG	NEG	POS	NEG	NEG	NEG	NEG	NEG	NEG	NEG	NEG	NEG	NEG	NEG	NEG	NEG	NEG	NEG	NEG	NEG	POS	NEG	NEG	NEG	NEG	NEG	NEG	NEG	NEG	NEG	NEG	NEG	NEG	NEG	NEG	POS	NEG	NEG	NEG	NEG	NEG	NEG	NEG	NEG
Iasi-30-P17	Carrier	NEG	NEG	NEG	POS	NEG	NEG	NEG	NEG	NEG	NEG	NEG	NEG	AMB	NEG	NEG	NEG	NEG	NEG	NEG	NEG	NEG	POS	NEG	NEG	NEG	NEG	AMB	NEG	NEG	NEG	NEG	NEG	NEG	NEG	NEG	NEG	POS	NEG	NEG	NEG	NEG	NEG	NEG	NEG	NEG
Iasi-39-P28	Carrier	NEG	NEG	NEG	POS	NEG	NEG	NEG	NEG	NEG	NEG	NEG	NEG	NEG	NEG	NEG	NEG	NEG	NEG	NEG	NEG	NEG	POS	NEG	NEG	NEG	NEG	NEG	NEG	NEG	NEG	NEG	NEG	NEG	NEG	NEG	NEG	POS	NEG	NEG	NEG	NEG	NEG	NEG	NEG	NEG
Iasi-04-26	BSI	NEG	NEG	NEG	POS	NEG	NEG	NEG	NEG	NEG	NEG	NEG	NEG	NEG	NEG	NEG	NEG	NEG	NEG	NEG	NEG	NEG	POS	NEG	NEG	NEG	NEG	NEG	NEG	NEG	NEG	NEG	NEG	NEG	NEG	NEG	NEG	POS	NEG	NEG	NEG	NEG	NEG	NEG	NEG	NEG
Iasi-09-72	BSI	NEG	NEG	NEG	POS	NEG	POS	POS	NEG	NEG	NEG	NEG	NEG	NEG	NEG	NEG	NEG	NEG	NEG	NEG	NEG	NEG	POS	NEG	NEG	NEG	NEG	NEG	NEG	NEG	NEG	NEG	NEG	NEG	NEG	NEG	NEG	POS	NEG	NEG	NEG	NEG	NEG	NEG	NEG	NEG
Iasi-11-131	BSI	NEG	NEG	NEG	POS	NEG	NEG	NEG	NEG	NEG	NEG	NEG	NEG	NEG	NEG	NEG	NEG	NEG	NEG	NEG	NEG	NEG	POS	NEG	NEG	NEG	NEG	NEG	NEG	NEG	NEG	NEG	NEG	NEG	NEG	NEG	NEG	POS	NEG	NEG	NEG	NEG	NEG	NEG	NEG	NEG
Iasi-14-143	BSI	NEG	NEG	NEG	POS	NEG	NEG	NEG	NEG	NEG	NEG	NEG	NEG	NEG	NEG	NEG	NEG	NEG	NEG	NEG	NEG	NEG	POS	NEG	NEG	NEG	NEG	NEG	NEG	NEG	NEG	NEG	NEG	NEG	NEG	NEG	NEG	POS	NEG	NEG	NEG	NEG	NEG	NEG	NEG	NEG
Iasi-18-192	BSI	NEG	NEG	NEG	NEG	NEG	NEG	NEG	NEG	NEG	NEG	NEG	NEG	NEG	NEG	NEG	NEG	NEG	NEG	NEG	NEG	NEG	POS	NEG	NEG	NEG	NEG	NEG	NEG	NEG	NEG	NEG	NEG	NEG	NEG	NEG	NEG	POS	NEG	NEG	NEG	NEG	NEG	NEG	NEG	NEG
Iasi-23-253	BSI	NEG	NEG	NEG	POS	NEG	NEG	NEG	NEG	NEG	NEG	NEG	NEG	AMB	NEG	NEG	NEG	NEG	NEG	NEG	NEG	NEG	POS	NEG	NEG	NEG	NEG	NEG	NEG	NEG	NEG	NEG	NEG	NEG	NEG	NEG	NEG	POS	NEG	NEG	NEG	NEG	NEG	NEG	NEG	NEG
Iasi-35-322	BSI	NEG	NEG	NEG	POS	NEG	NEG	NEG	NEG	NEG	NEG	NEG	NEG	NEG	NEG	NEG	NEG	NEG	NEG	NEG	NEG	NEG	POS	NEG	NEG	NEG	NEG	NEG	NEG	NEG	NEG	NEG	NEG	NEG	NEG	NEG	NEG	POS	NEG	NEG	NEG	NEG	NEG	NEG	NEG	NEG
Iasi-40-380	BSI	NEG	NEG	NEG	POS	NEG	NEG	NEG	NEG	NEG	NEG	NEG	NEG	NEG	NEG	NEG	NEG	NEG	NEG	NEG	NEG	NEG	POS	NEG	NEG	NEG	NEG	NEG	NEG	NEG	NEG	NEG	NEG	NEG	NEG	NEG	NEG	POS	NEG	NEG	NEG	NEG	NEG	NEG	NEG	NEG
Iasi-46-406	BSI	NEG	NEG	NEG	NEG	NEG	NEG	NEG	NEG	NEG	NEG	NEG	NEG	NEG	NEG	NEG	NEG	NEG	NEG	NEG	NEG	NEG	POS	NEG	NEG	NEG	NEG	NEG	NEG	NEG	NEG	NEG	NEG	NEG	NEG	NEG	NEG	POS	NEG	NEG	NEG	NEG	NEG	NEG	NEG	NEG
Iasi-02-6	SSTI	NEG	NEG	NEG	POS	NEG	NEG	NEG	NEG	NEG	NEG	NEG	NEG	NEG	NEG	NEG	NEG	NEG	NEG	NEG	NEG	NEG	POS	NEG	NEG	NEG	NEG	NEG	NEG	NEG	NEG	NEG	NEG	NEG	NEG	NEG	NEG	POS	NEG	NEG	NEG	NEG	NEG	NEG	NEG	NEG
Iasi-09-97	SSTI	NEG	NEG	NEG	POS	NEG	NEG	NEG	NEG	NEG	NEG	NEG	NEG	NEG	NEG	NEG	NEG	NEG	NEG	NEG	NEG	NEG	POS	NEG	NEG	NEG	NEG	NEG	NEG	NEG	NEG	NEG	NEG	NEG	NEG	NEG	NEG	POS	NEG	NEG	NEG	NEG	NEG	NEG	NEG	NEG
Iasi-19-182	SSTI	NEG	NEG	NEG	POS	NEG	NEG	NEG	NEG	NEG	NEG	NEG	NEG	NEG	NEG	NEG	NEG	NEG	NEG	NEG	NEG	NEG	POS	NEG	NEG	NEG	NEG	NEG	NEG	NEG	NEG	NEG	NEG	NEG	NEG	NEG	NEG	POS	NEG	NEG	NEG	NEG	NEG	NEG	NEG	NEG
Iasi-31-314	SSTI	NEG	NEG	NEG	POS	NEG	NEG	NEG	NEG	NEG	NEG	NEG	NEG	NEG	NEG	NEG	NEG	NEG	NEG	NEG	NEG	NEG	POS	NEG	NEG	NEG	NEG	NEG	NEG	NEG	NEG	NEG	NEG	NEG	NEG	NEG	NEG	POS	NEG	NEG	NEG	NEG	NEG	NEG	NEG	NEG
Iasi-33-367	SSTI	NEG	NEG	NEG	POS	NEG	NEG	NEG	NEG	NEG	NEG	NEG	NEG	NEG	NEG	NEG	NEG	NEG	NEG	NEG	NEG	NEG	POS	NEG	NEG	NEG	NEG	NEG	NEG	NEG	NEG	NEG	NEG	NEG	NEG	NEG	NEG	POS	NEG	NEG	NEG	NEG	NEG	NEG	NEG	NEG
Iasi-39-378	SSTI	NEG	NEG	NEG	POS	NEG	NEG	NEG	NEG	NEG	NEG	NEG	NEG	NEG	NEG	NEG	NEG	NEG	NEG	NEG	NEG	NEG	POS	NEG	NEG	NEG	NEG	NEG	NEG	NEG	NEG	NEG	NEG	NEG	NEG	NEG	NEG	POS	NEG	NEG	NEG	NEG	NEG	NEG	NEG	NEG
Iasi-42-394	SSTI	NEG	NEG	NEG	POS	NEG	NEG	NEG	NEG	NEG	NEG	NEG	NEG	NEG	NEG	NEG	NEG	NEG	NEG	NEG	NEG	NEG	POS	NEG	NEG	NEG	NEG	NEG	NEG	NEG	NEG	NEG	NEG	NEG	NEG	NEG	NEG	POS	NEG	NEG	NEG	NEG	NEG	NEG	NEG	NEG
Iasi-44-409	SSTI	NEG	NEG	NEG	NEG	NEG	NEG	NEG	NEG	NEG	NEG	NEG	NEG	NEG	NEG	POS	NEG	NEG	NEG	NEG	NEG	NEG	POS	NEG	NEG	NEG	NEG	NEG	NEG	NEG	NEG	NEG	NEG	NEG	NEG	NEG	NEG	POS	NEG	NEG	NEG	NEG	NEG	NEG	NEG	NEG
Iasi-45-405	SSTI	NEG	NEG	NEG	POS	NEG	NEG	NEG	NEG	NEG	NEG	NEG	NEG	NEG	NEG	NEG	NEG	NEG	NEG	NEG	NEG	NEG	POS	NEG	NEG	NEG	NEG	NEG	NEG	NEG	NEG	NEG	NEG	NEG	NEG	NEG	NEG	POS	NEG	NEG	NEG	NEG	NEG	NEG	NEG	NEG
Iasi-50-425	SSTI	NEG	NEG	NEG	POS	NEG	NEG	NEG	NEG	NEG	NEG	NEG	NEG	NEG	NEG	NEG	NEG	NEG	NEG	NEG	NEG	NEG	POS	NEG	NEG	NEG	NEG	NEG	NEG	NEG	NEG	NEG	NEG	NEG	NEG	NEG	NEG	POS	NEG	NEG	NEG	NEG	NEG	NEG	NEG	NEG
Iasi-50-428	SSTI	NEG	NEG	NEG	POS	NEG	NEG	NEG	NEG	NEG	NEG	NEG	NEG	NEG	NEG	NEG	NEG	NEG	NEG	NEG	NEG	NEG	POS	NEG	NEG	NEG	NEG	NEG	NEG	NEG	NEG	NEG	NEG	NEG	NEG	NEG	NEG	POS	NEG	NEG	NEG	NEG	NEG	NEG	NEG	NEG
Iasi-51-37	SSTI	NEG	NEG	NEG	POS	NEG	NEG	NEG	NEG	NEG	NEG	NEG	NEG	NEG	NEG	NEG	NEG	NEG	NEG	NEG	NEG	NEG	POS	NEG	NEG	NEG	NEG	NEG	NEG	NEG	NEG	NEG	NEG	NEG	NEG	NEG	NEG	POS	NEG	NEG	NEG	NEG	NEG	NEG	NEG	NEG
Iasi-01-S8	Carrier	NEG	NEG	NEG	POS	NEG	NEG	NEG	NEG	NEG	NEG	NEG	NEG	AMB	NEG	NEG	NEG	NEG	NEG	NEG	NEG	NEG	POS	NEG	NEG	NEG	NEG	NEG	NEG	NEG	NEG	NEG	NEG	NEG	NEG	NEG	NEG	POS	NEG	NEG	NEG	NEG	NEG	NEG	NEG	NEG
Iasi-08-S17	Carrier	NEG	NEG	NEG	POS	NEG	NEG	NEG	NEG	NEG	NEG	NEG	NEG	NEG	NEG	NEG	NEG	NEG	NEG	NEG	NEG	NEG	POS	NEG	NEG	NEG	NEG	NEG	NEG	NEG	NEG	NEG	NEG	NEG	NEG	NEG	NEG	POS	NEG	NEG	NEG	NEG	NEG	NEG	NEG	NEG
Iasi-10-S23	Carrier	NEG	NEG	NEG	POS	NEG	NEG	NEG	NEG	NEG	NEG	NEG	NEG	NEG	NEG	NEG	NEG	NEG	NEG	NEG	NEG	NEG	POS	NEG	NEG	NEG	NEG	NEG	NEG	NEG	NEG	NEG	NEG	NEG	NEG	NEG	NEG	POS	NEG	NEG	NEG	NEG	NEG	NEG	NEG	NEG
Iasi-13-S27	Carrier	NEG	NEG	NEG	POS	NEG	NEG	NEG	NEG	NEG	NEG	NEG	NEG	AMB	NEG	NEG	NEG	NEG	NEG	NEG	NEG	NEG	POS	NEG	NEG	NEG	NEG	NEG	NEG	NEG	NEG	NEG	NEG	NEG	NEG	NEG	NEG	POS	NEG	NEG	NEG	NEG	NEG	NEG	NEG	NEG
Iasi-14-S28	Carrier	NEG	NEG	NEG	POS	NEG	NEG	NEG	NEG	NEG	NEG	NEG	NEG	NEG	NEG	NEG	NEG	NEG	NEG	NEG	NEG	NEG	POS	NEG	NEG	NEG	NEG	NEG	NEG	NEG	NEG	NEG	NEG	NEG	NEG	NEG	NEG	POS	NEG	NEG	NEG	NEG	NEG	NEG	NEG	NEG
Iasi-15-S29	Carrier	NEG	NEG	NEG	POS	NEG	NEG	NEG	NEG	NEG	NEG	NEG	NEG	AMB	NEG	NEG	NEG	NEG	NEG	NEG	NEG	NEG	POS	NEG	NEG	NEG	NEG	NEG	NEG	NEG	NEG	NEG	NEG	NEG	NEG	NEG	NEG	POS	NEG	NEG	NEG	NEG	NEG	NEG	NEG	NEG
Iasi-25-P10	Carrier	NEG	NEG	NEG	NEG	NEG	NEG	NEG	NEG	NEG	NEG	NEG	NEG	AMB	NEG	POS	NEG	NEG	NEG	NEG	NEG	NEG	POS	NEG	NEG	NEG	NEG	NEG	NEG	NEG	NEG	NEG	NEG	NEG	NEG	NEG	NEG	POS	NEG	NEG	NEG	NEG	NEG	NEG	NEG	NEG
Iasi-26-P11	Carrier	NEG	NEG	NEG	POS	NEG	NEG	NEG	NEG	NEG	NEG	NEG	NEG	NEG	NEG	POS	NEG	NEG	NEG	NEG	NEG	NEG	POS	NEG	NEG	NEG	NEG	NEG	NEG	NEG	NEG	NEG	NEG	NEG	NEG	NEG	NEG	POS	NEG	NEG	NEG	NEG	NEG	NEG	NEG	NEG
Iasi-32-P19	Carrier	NEG	NEG	NEG	POS	NEG	NEG	NEG	NEG	NEG	NEG	NEG	NEG	NEG	NEG	POS	NEG	NEG	NEG	NEG	NEG	NEG	POS	NEG	NEG	NEG	NEG	NEG	NEG	NEG	NEG	NEG	NEG	NEG	NEG	NEG	NEG	POS	NEG	NEG	NEG	NEG	NEG	NEG	NEG	NEG
Iasi-33-P22	Carrier	NEG	NEG	NEG	POS	NEG	NEG	NEG	NEG	NEG	NEG	NEG	NEG	NEG	NEG	NEG	NEG	NEG	NEG	NEG	NEG	NEG	POS	NEG	NEG	NEG	NEG	NEG	NEG	NEG	NEG	NEG	NEG	NEG	NEG	NEG	NEG	POS	NEG	NEG	NEG	NEG	NEG	NEG	NEG	NEG
Iasi-41-P44	Carrier	NEG	NEG	NEG	POS	NEG	NEG	NEG	NEG	NEG	NEG	NEG	NEG	NEG	NEG	NEG	NEG	NEG	NEG	NEG	NEG	NEG	POS	NEG	NEG	NEG	NEG	NEG	NEG	NEG	NEG	NEG	NEG	NEG	NEG	NEG	NEG	POS	NEG	NEG	NEG	NEG	NEG	NEG	NEG	NEG
Iasi-45-G515	Carrier	NEG	NEG	NEG	POS	NEG	NEG	NEG	NEG	NEG	NEG	NEG	NEG	NEG	NEG	NEG	NEG	NEG	NEG	NEG	NEG	NEG	POS	NEG	NEG	NEG	NEG	NEG	NEG	NEG	NEG	NEG	NEG	NEG	NEG	NEG	NEG	POS	NEG	NEG	NEG	NEG	NEG	NEG	NEG	NEG
Iasi-02-39	misc. MRSA	NEG	NEG	NEG	POS	NEG	NEG	NEG	NEG	NEG	NEG	NEG	NEG	NEG	NEG	NEG	NEG	NEG	NEG	NEG	NEG	NEG	POS	NEG	NEG	NEG	NEG	NEG	NEG	NEG	NEG	NEG	NEG	NEG	NEG	NEG	NEG	POS	NEG	NEG	NEG	NEG	NEG	NEG	NEG	NEG
Iasi-24-263	misc. MRSA	NEG	NEG	NEG	POS	NEG	NEG	NEG	NEG	NEG	NEG	NEG	NEG	NEG	NEG	NEG	NEG	NEG	NEG	NEG	NEG	NEG	POS	NEG	NEG	NEG	NEG	NEG	NEG	NEG	NEG	NEG	NEG	NEG	NEG	NEG	NEG	POS	NEG	NEG	NEG	NEG	NEG	NEG	NEG	NEG
Iasi-26-285	misc. MRSA	NEG	NEG	NEG	POS	NEG	NEG	NEG	NEG	NEG	NEG	NEG	NEG	NEG	NEG	NEG	NEG	NEG	NEG	NEG	NEG	NEG	POS	NEG	NEG	NEG	NEG	NEG	NEG	NEG	NEG	NEG	NEG	NEG	NEG	NEG	NEG	POS	NEG	NEG	NEG	NEG	NEG	NEG	NEG	NEG
Iasi-30-306	misc. MRSA	NEG	NEG	NEG	POS	NEG	NEG	NEG	NEG	NEG	NEG	NEG	NEG	AMB	NEG	NEG	NEG	NEG	NEG	NEG	NEG	NEG	POS	NEG	NEG	NEG	NEG	NEG	NEG	NEG	NEG	NEG	NEG	NEG	NEG	NEG	NEG	POS	NEG	NEG	NEG	NEG	NEG	NEG	NEG	NEG
Iasi-41-390	misc. MRSA	NEG	NEG	NEG	POS	NEG	NEG	NEG	NEG	NEG	NEG	NEG	NEG	NEG	NEG	NEG	NEG	NEG	NEG	NEG	NEG	NEG	POS	NEG	NEG	NEG	NEG	NEG	NEG	NEG	NEG	NEG	NEG	NEG	NEG	NEG	NEG	POS	NEG	NEG	NEG	NEG	NEG	NEG	NEG	NEG
Iasi-47-407	misc. MRSA	NEG	NEG	NEG	POS	NEG	NEG	NEG	NEG	NEG	NEG	NEG	NEG	NEG	NEG	NEG	NEG	NEG	NEG	NEG	NEG	NEG	POS	NEG	NEG	NEG	NEG	NEG	NEG	NEG	NEG	NEG	NEG	NEG	NEG	NEG	NEG	POS	NEG	NEG	NEG	NEG	NEG	NEG	NEG	NEG
Iasi-55-408CP	misc. MRSA	NEG	NEG	NEG	POS	NEG	NEG	NEG	NEG	NEG	NEG	NEG	NEG	NEG	NEG	NEG	NEG	NEG	NEG	NEG	NEG	NEG	POS	NEG	NEG	NEG	NEG	NEG	NEG	NEG	NEG	NEG	NEG	NEG	NEG	NEG	NEG	POS	NEG	NEG	NEG	NEG	NEG	NEG	NEG	NEG
Iasi-15-151	BSI	NEG	NEG	NEG	NEG	NEG	NEG	NEG	NEG	NEG	NEG	NEG	NEG	POS	AMB	NEG	NEG	NEG	NEG	NEG	NEG	NEG	POS	NEG	NEG	NEG	NEG	NEG	NEG	NEG	NEG	POS	NEG	NEG	NEG	NEG	POS	NEG	POS	NEG	NEG	NEG	POS	POS	POS	POS
Iasi-46-386	BSI	NEG	NEG	NEG	NEG	NEG	NEG	NEG	NEG	NEG	NEG	NEG	AMB	POS	AMB	NEG	NEG	NEG	NEG	NEG	NEG	NEG	POS	NEG	NEG	NEG	NEG	NEG	NEG	POS	AMB	NEG	NEG	NEG	NEG	NEG	POS	NEG	POS	NEG	NEG	NEG	POS	POS	POS	POS
Iasi-21-195	SSTI	NEG	POS	NEG	NEG	POS	NEG	NEG	NEG	NEG	NEG	NEG	NEG	POS	NEG	NEG	NEG	NEG	NEG	NEG	NEG	NEG	POS	NEG	NEG	NEG	NEG	NEG	NEG	NEG	NEG	POS	NEG	NEG	NEG	NEG	POS	NEG	POS	NEG	NEG	NEG	POS	POS	POS	POS
Iasi-37-375	SSTI	NEG	NEG	NEG	NEG	NEG	NEG	NEG	NEG	NEG	NEG	NEG	NEG	POS	NEG	NEG	NEG	NEG	NEG	NEG	NEG	NEG	POS	NEG	NEG	NEG	NEG	NEG	NEG	NEG	NEG	NEG	NEG	NEG	NEG	NEG	POS	NEG	POS	NEG	NEG	NEG	POS	POS	POS	POS
Iasi-45-415	SSTI	NEG	NEG	NEG	NEG	POS	NEG	NEG	NEG	NEG	NEG	NEG	NEG	POS	NEG	NEG	NEG	NEG	NEG	NEG	NEG	NEG	POS	NEG	NEG	NEG	NEG	NEG	NEG	NEG	NEG	NEG	NEG	NEG	NEG	NEG	POS	NEG	POS	NEG	NEG	NEG	POS	POS	POS	POS
Iasi-09-S19	Carrier	NEG	NEG	NEG	NEG	NEG	NEG	NEG	NEG	NEG	NEG	NEG	NEG	POS	AMB	NEG	NEG	NEG	NEG	NEG	NEG	NEG	POS	NEG	NEG	NEG	NEG	NEG	NEG	NEG	NEG	NEG	NEG	NEG	NEG	NEG	POS	NEG	POS	NEG	NEG	NEG	POS	POS	POS	POS
Iasi-53-434	BSI	NEG	NEG	NEG	POS	NEG	NEG	NEG	NEG	NEG	NEG	NEG	AMB	POS	AMB	NEG	NEG	NEG	NEG	NEG	NEG	NEG	POS	NEG	NEG	NEG	NEG	NEG	NEG	POS	AMB	NEG	NEG	NEG	NEG	NEG	POS	NEG	POS	NEG	NEG	NEG	POS	POS	POS	POS
Iasi-03-40	misc. MRSA	NEG	NEG	NEG	NEG	NEG	NEG	NEG	NEG	NEG	NEG	NEG	NEG	POS	AMB	NEG	NEG	NEG	NEG	NEG	NEG	NEG	POS	NEG	NEG	NEG	NEG	NEG	NEG	NEG	NEG	NEG	NEG	NEG	POS	NEG	POS	NEG	POS	POS	NEG	NEG	POS	POS	POS	POS
Iasi-04-43	misc. MRSA	NEG	NEG	NEG	NEG	NEG	NEG	NEG	NEG	NEG	NEG	NEG	NEG	POS	NEG	NEG	NEG	NEG	NEG	NEG	NEG	NEG	POS	NEG	NEG	NEG	NEG	NEG	NEG	NEG	NEG	NEG	NEG	NEG	POS	NEG	POS	NEG	POS	POS	NEG	NEG	POS	POS	POS	POS
Iasi-01-20	misc. MRSA	NEG	NEG	NEG	POS	NEG	NEG	NEG	NEG	NEG	NEG	NEG	NEG	POS	NEG	NEG	NEG	NEG	NEG	NEG	NEG	NEG	POS	NEG	NEG	NEG	NEG	NEG	NEG	NEG	NEG	NEG	NEG	NEG	POS	NEG	POS	NEG	POS	POS	NEG	NEG	POS	POS	POS	POS
Iasi-35-228	BSI	NEG	NEG	NEG	NEG	NEG	NEG	NEG	NEG	NEG	NEG	NEG	NEG	POS	AMB	NEG	NEG	NEG	NEG	NEG	NEG	NEG	POS	NEG	NEG	NEG	NEG	NEG	NEG	POS	NEG	NEG	NEG	NEG	NEG	NEG	NEG	NEG	NEG	NEG	NEG	NEG	NEG	NEG	NEG	NEG
Iasi-38-293	BSI	NEG	NEG	NEG	NEG	NEG	NEG	NEG	NEG	NEG	NEG	NEG	NEG	AMB	NEG	NEG	NEG	NEG	NEG	NEG	NEG	NEG	POS	NEG	NEG	NEG	AMB	NEG	NEG	NEG	NEG	POS	NEG	NEG	NEG	NEG	NEG	NEG	NEG	NEG	NEG	NEG	NEG	NEG	NEG	NEG
Iasi-31-P18	Carrier	NEG	NEG	NEG	NEG	NEG	NEG	NEG	NEG	NEG	NEG	NEG	NEG	AMB	NEG	NEG	NEG	NEG	NEG	NEG	NEG	NEG	POS	NEG	NEG	NEG	NEG	NEG	NEG	NEG	NEG	POS	NEG	NEG	NEG	NEG	NEG	NEG	NEG	NEG	NEG	NEG	NEG	NEG	NEG	NEG
Iasi-40-P42	Carrier	NEG	NEG	NEG	NEG	NEG	NEG	NEG	NEG	NEG	NEG	NEG	NEG	AMB	NEG	NEG	NEG	NEG	NEG	NEG	NEG	NEG	POS	NEG	NEG	NEG	NEG	NEG	NEG	NEG	NEG	POS	NEG	NEG	NEG	NEG	NEG	NEG	NEG	NEG	NEG	NEG	NEG	NEG	NEG	NEG
Iasi-40-303	BSI	NEG	NEG	NEG	NEG	NEG	NEG	NEG	NEG	NEG	NEG	NEG	NEG	POS	AMB	NEG	NEG	NEG	NEG	NEG	NEG	NEG	POS	NEG	NEG	NEG	POS	POS	NEG	NEG	NEG	POS	NEG	NEG	NEG	NEG	NEG	NEG	NEG	NEG	NEG	NEG	NEG	NEG	NEG	NEG
Iasi-48-422	SSTI	NEG	NEG	NEG	NEG	NEG	NEG	NEG	NEG	NEG	NEG	NEG	NEG	POS	NEG	NEG	NEG	NEG	NEG	NEG	NEG	NEG	POS	NEG	NEG	NEG	NEG	NEG	NEG	NEG	NEG	NEG	NEG	NEG	NEG	NEG	NEG	NEG	NEG	NEG	NEG	NEG	NEG	NEG	NEG	NEG
Iasi-54-435	BSI	NEG	NEG	NEG	NEG	NEG	NEG	NEG	NEG	NEG	NEG	NEG	NEG	POS	POS	NEG	NEG	NEG	NEG	NEG	NEG	NEG	POS	NEG	NEG	NEG	AMB	NEG	NEG	NEG	NEG	NEG	NEG	NEG	NEG	NEG	NEG	NEG	NEG	NEG	NEG	NEG	NEG	NEG	NEG	NEG
Iasi-16-161	SSTI	NEG	NEG	NEG	NEG	NEG	NEG	NEG	NEG	NEG	NEG	NEG	NEG	POS	NEG	NEG	NEG	NEG	NEG	NEG	NEG	NEG	POS	NEG	NEG	NEG	NEG	NEG	NEG	NEG	NEG	NEG	NEG	NEG	NEG	NEG	NEG	NEG	NEG	NEG	POS	NEG	NEG	NEG	NEG	NEG
Iasi-27-221	SSTI	NEG	NEG	NEG	NEG	NEG	NEG	NEG	NEG	NEG	NEG	NEG	NEG	POS	NEG	NEG	NEG	NEG	NEG	NEG	NEG	NEG	POS	NEG	NEG	NEG	NEG	NEG	NEG	NEG	NEG	NEG	NEG	NEG	NEG	NEG	NEG	NEG	NEG	NEG	POS	NEG	NEG	NEG	NEG	NEG
Iasi-09-85	BSI	NEG	NEG	NEG	NEG	NEG	NEG	NEG	NEG	NEG	NEG	NEG	NEG	POS	NEG	NEG	NEG	NEG	NEG	NEG	NEG	NEG	POS	NEG	NEG	NEG	NEG	NEG	NEG	NEG	NEG	NEG	NEG	NEG	NEG	NEG	NEG	NEG	NEG	NEG	POS	NEG	NEG	NEG	NEG	NEG
Iasi-17-189	BSI	NEG	NEG	NEG	NEG	NEG	NEG	NEG	NEG	NEG	NEG	NEG	NEG	POS	NEG	NEG	NEG	NEG	NEG	NEG	NEG	NEG	POS	NEG	NEG	NEG	NEG	NEG	NEG	NEG	NEG	NEG	NEG	NEG	NEG	NEG	NEG	NEG	NEG	NEG	POS	NEG	NEG	NEG	NEG	NEG
Iasi-20-200	BSI	NEG	NEG	NEG	NEG	NEG	NEG	NEG	NEG	NEG	NEG	NEG	NEG	POS	NEG	NEG	NEG	NEG	NEG	NEG	NEG	NEG	POS	NEG	NEG	NEG	NEG	NEG	NEG	NEG	NEG	NEG	NEG	NEG	NEG	NEG	NEG	NEG	NEG	NEG	POS	NEG	NEG	NEG	NEG	NEG
Iasi-34-321	BSI	NEG	NEG	NEG	NEG	NEG	NEG	NEG	NEG	NEG	NEG	NEG	NEG	POS	NEG	NEG	NEG	NEG	NEG	NEG	NEG	NEG	POS	NEG	NEG	NEG	NEG	NEG	NEG	NEG	NEG	NEG	NEG	NEG	NEG	NEG	NEG	NEG	NEG	NEG	POS	NEG	NEG	NEG	NEG	NEG
Iasi-04-57	SSTI	NEG	NEG	NEG	NEG	NEG	NEG	NEG	NEG	NEG	NEG	NEG	NEG	POS	NEG	NEG	NEG	NEG	NEG	NEG	NEG	NEG	POS	NEG	NEG	NEG	NEG	NEG	NEG	NEG	NEG	NEG	NEG	NEG	NEG	NEG	NEG	NEG	NEG	NEG	POS	NEG	NEG	NEG	NEG	NEG
Iasi-05-59	SSTI	NEG	NEG	NEG	NEG	NEG	NEG	NEG	NEG	NEG	NEG	NEG	NEG	POS	NEG	NEG	NEG	NEG	NEG	NEG	NEG	NEG	POS	NEG	NEG	NEG	NEG	NEG	NEG	NEG	NEG	NEG	NEG	NEG	NEG	NEG	NEG	NEG	NEG	NEG	POS	NEG	NEG	NEG	NEG	NEG
Iasi-06-64	SSTI	NEG	NEG	NEG	NEG	NEG	NEG	NEG	NEG	NEG	NEG	NEG	NEG	POS	NEG	NEG	NEG	NEG	NEG	NEG	NEG	NEG	POS	NEG	NEG	NEG	NEG	NEG	NEG	NEG	NEG	NEG	NEG	NEG	NEG	NEG	NEG	NEG	NEG	NEG	POS	NEG	NEG	NEG	NEG	NEG
Iasi-10-108	SSTI	NEG	NEG	NEG	NEG	NEG	NEG	NEG	NEG	NEG	NEG	NEG	NEG	POS	#ZAHL!	NEG	NEG	NEG	NEG	NEG	NEG	NEG	POS	NEG	NEG	NEG	NEG	NEG	NEG	NEG	NEG	NEG	NEG	NEG	NEG	NEG	NEG	NEG	NEG	NEG	POS	NEG	NEG	NEG	NEG	NEG
Iasi-12-117	SSTI	NEG	NEG	NEG	NEG	NEG	NEG	NEG	NEG	NEG	NEG	NEG	NEG	POS	NEG	NEG	NEG	NEG	NEG	NEG	NEG	NEG	POS	NEG	NEG	NEG	NEG	NEG	NEG	NEG	NEG	NEG	NEG	NEG	NEG	NEG	NEG	NEG	NEG	NEG	POS	NEG	NEG	NEG	NEG	NEG
Iasi-12-138	SSTI	NEG	NEG	NEG	NEG	NEG	NEG	NEG	NEG	NEG	NEG	NEG	NEG	POS	NEG	NEG	NEG	NEG	NEG	NEG	NEG	NEG	POS	NEG	NEG	NEG	NEG	NEG	NEG	NEG	NEG	NEG	NEG	NEG	NEG	NEG	NEG	NEG	NEG	NEG	POS	NEG	NEG	NEG	NEG	NEG
Iasi-13-140	SSTI	NEG	NEG	NEG	NEG	NEG	NEG	NEG	NEG	NEG	NEG	NEG	NEG	POS	NEG	NEG	NEG	NEG	NEG	NEG	NEG	NEG	POS	NEG	NEG	NEG	NEG	NEG	NEG	NEG	NEG	NEG	NEG	NEG	NEG	NEG	NEG	NEG	NEG	NEG	POS	NEG	NEG	NEG	NEG	NEG
Iasi-15-152	SSTI	NEG	NEG	NEG	NEG	NEG	NEG	NEG	NEG	NEG	NEG	NEG	NEG	POS	NEG	NEG	NEG	NEG	NEG	NEG	NEG	NEG	POS	NEG	NEG	NEG	NEG	NEG	NEG	NEG	NEG	NEG	NEG	NEG	NEG	NEG	NEG	NEG	NEG	NEG	POS	NEG	NEG	NEG	NEG	NEG
Iasi-17-168	SSTI	NEG	NEG	NEG	NEG	NEG	NEG	NEG	NEG	NEG	NEG	NEG	NEG	POS	NEG	NEG	NEG	NEG	NEG	NEG	NEG	NEG	POS	NEG	NEG	NEG	NEG	NEG	NEG	NEG	NEG	NEG	NEG	NEG	NEG	NEG	NEG	NEG	NEG	NEG	POS	NEG	NEG	NEG	NEG	NEG
Iasi-20-193	SSTI	NEG	NEG	NEG	NEG	NEG	NEG	NEG	NEG	NEG	NEG	NEG	NEG	POS	NEG	NEG	NEG	NEG	NEG	NEG	NEG	NEG	POS	NEG	NEG	NEG	NEG	NEG	NEG	NEG	NEG	NEG	NEG	NEG	NEG	NEG	NEG	NEG	NEG	NEG	POS	NEG	NEG	NEG	NEG	NEG
Iasi-26-218	SSTI	NEG	NEG	NEG	NEG	NEG	NEG	NEG	NEG	NEG	NEG	NEG	NEG	POS	NEG	NEG	NEG	NEG	NEG	NEG	NEG	NEG	POS	NEG	NEG	NEG	NEG	NEG	NEG	NEG	NEG	NEG	NEG	NEG	NEG	NEG	NEG	NEG	NEG	NEG	POS	NEG	NEG	NEG	NEG	NEG
Iasi-28-335	SSTI	NEG	NEG	NEG	POS	NEG	NEG	NEG	NEG	NEG	NEG	NEG	NEG	POS	NEG	POS	NEG	NEG	NEG	NEG	NEG	NEG	POS	NEG	NEG	NEG	NEG	NEG	NEG	NEG	NEG	NEG	NEG	NEG	NEG	NEG	NEG	NEG	NEG	NEG	POS	NEG	NEG	NEG	NEG	NEG
Iasi-29-299b	SSTI	NEG	NEG	NEG	NEG	NEG	NEG	NEG	NEG	NEG	NEG	NEG	NEG	POS	AMB	NEG	NEG	NEG	NEG	NEG	NEG	NEG	POS	NEG	NEG	NEG	NEG	NEG	NEG	NEG	NEG	NEG	NEG	NEG	NEG	NEG	NEG	NEG	NEG	NEG	POS	NEG	NEG	NEG	NEG	NEG
Iasi-32-364	SSTI	NEG	NEG	NEG	NEG	NEG	NEG	NEG	NEG	NEG	NEG	NEG	NEG	POS	NEG	NEG	NEG	NEG	NEG	NEG	NEG	NEG	POS	NEG	NEG	NEG	NEG	NEG	NEG	NEG	NEG	NEG	NEG	NEG	NEG	NEG	NEG	NEG	NEG	NEG	POS	NEG	NEG	NEG	NEG	NEG
Iasi-369	SSTI	NEG	NEG	NEG	NEG	NEG	NEG	NEG	NEG	NEG	NEG	NEG	NEG	POS	NEG	NEG	NEG	NEG	NEG	NEG	NEG	NEG	POS	NEG	NEG	NEG	NEG	NEG	NEG	NEG	NEG	NEG	NEG	NEG	NEG	NEG	NEG	NEG	NEG	NEG	POS	NEG	NEG	NEG	NEG	NEG
Iasi-03-S11	Carrier	NEG	NEG	NEG	NEG	NEG	NEG	NEG	NEG	NEG	NEG	NEG	NEG	POS	AMB	NEG	NEG	NEG	NEG	NEG	NEG	NEG	POS	NEG	NEG	NEG	NEG	NEG	NEG	NEG	NEG	NEG	NEG	NEG	NEG	NEG	NEG	NEG	NEG	NEG	POS	NEG	NEG	NEG	NEG	NEG
Iasi-19-S35	Carrier	NEG	NEG	NEG	NEG	NEG	NEG	NEG	NEG	NEG	NEG	NEG	NEG	POS	AMB	NEG	NEG	NEG	NEG	NEG	NEG	NEG	POS	NEG	NEG	NEG	NEG	NEG	NEG	NEG	NEG	NEG	NEG	NEG	NEG	NEG	NEG	NEG	NEG	NEG	POS	NEG	NEG	NEG	NEG	NEG
Iasi-20-P1	Carrier	NEG	NEG	NEG	NEG	NEG	NEG	NEG	NEG	NEG	NEG	NEG	NEG	POS	AMB	NEG	NEG	NEG	NEG	NEG	NEG	NEG	POS	NEG	NEG	NEG	NEG	NEG	NEG	NEG	NEG	NEG	NEG	NEG	NEG	NEG	NEG	NEG	NEG	NEG	POS	NEG	NEG	NEG	NEG	NEG
Iasi-35-P24	Carrier	NEG	NEG	NEG	NEG	NEG	NEG	NEG	NEG	NEG	NEG	NEG	NEG	POS	NEG	NEG	NEG	NEG	NEG	NEG	NEG	NEG	POS	NEG	NEG	NEG	NEG	NEG	NEG	NEG	NEG	NEG	NEG	NEG	NEG	NEG	NEG	NEG	NEG	NEG	POS	NEG	NEG	NEG	NEG	NEG
Iasi-36-P25	Carrier	NEG	NEG	NEG	NEG	NEG	NEG	NEG	NEG	NEG	NEG	NEG	NEG	POS	AMB	NEG	NEG	NEG	NEG	NEG	NEG	NEG	POS	NEG	NEG	NEG	NEG	NEG	NEG	NEG	NEG	NEG	NEG	NEG	NEG	NEG	NEG	NEG	NEG	NEG	POS	NEG	NEG	NEG	NEG	NEG
Iasi-14-150	SSTI	NEG	NEG	NEG	NEG	NEG	NEG	NEG	NEG	NEG	NEG	NEG	NEG	POS	NEG	NEG	NEG	NEG	NEG	NEG	NEG	NEG	POS	NEG	NEG	NEG	NEG	NEG	NEG	NEG	NEG	NEG	NEG	NEG	NEG	NEG	NEG	NEG	NEG	NEG	POS	NEG	NEG	NEG	NEG	NEG
Iasi-07-54	BSI	NEG	NEG	NEG	NEG	POS	NEG	NEG	NEG	NEG	NEG	NEG	NEG	POS	NEG	NEG	NEG	NEG	NEG	NEG	NEG	NEG	POS	NEG	NEG	NEG	NEG	NEG	NEG	POS	NEG	NEG	NEG	NEG	NEG	NEG	NEG	NEG	NEG	NEG	POS	NEG	NEG	NEG	NEG	NEG
Iasi-16-188	BSI	NEG	NEG	NEG	NEG	POS	NEG	NEG	NEG	NEG	NEG	NEG	NEG	POS	NEG	NEG	NEG	NEG	NEG	NEG	NEG	NEG	POS	NEG	NEG	NEG	NEG	NEG	NEG	POS	NEG	NEG	NEG	NEG	NEG	NEG	NEG	NEG	NEG	NEG	POS	NEG	NEG	NEG	NEG	NEG
Iasi-32-318	BSI	NEG	NEG	NEG	NEG	POS	NEG	NEG	NEG	NEG	NEG	NEG	NEG	POS	NEG	NEG	NEG	NEG	NEG	NEG	NEG	NEG	POS	NEG	NEG	NEG	NEG	NEG	NEG	POS	NEG	NEG	NEG	NEG	NEG	NEG	NEG	NEG	NEG	NEG	POS	NEG	NEG	NEG	NEG	NEG
Iasi-42-397	BSI	NEG	NEG	NEG	NEG	POS	NEG	NEG	NEG	NEG	NEG	NEG	NEG	POS	NEG	NEG	NEG	NEG	NEG	NEG	NEG	NEG	POS	NEG	NEG	NEG	NEG	NEG	NEG	POS	NEG	NEG	NEG	NEG	NEG	NEG	NEG	NEG	NEG	NEG	POS	NEG	NEG	NEG	NEG	NEG
Iasi-48-408	BSI	NEG	NEG	NEG	NEG	POS	NEG	NEG	NEG	NEG	NEG	NEG	NEG	POS	NEG	NEG	NEG	NEG	NEG	NEG	NEG	NEG	POS	NEG	NEG	NEG	NEG	NEG	NEG	POS	NEG	NEG	NEG	NEG	NEG	NEG	NEG	NEG	NEG	NEG	POS	NEG	NEG	NEG	NEG	NEG
Iasi-49-430	BSI	NEG	NEG	NEG	NEG	POS	NEG	NEG	NEG	NEG	NEG	NEG	NEG	POS	AMB	NEG	NEG	NEG	NEG	NEG	NEG	NEG	POS	NEG	NEG	NEG	NEG	NEG	NEG	POS	AMB	NEG	NEG	NEG	NEG	NEG	NEG	NEG	NEG	NEG	POS	NEG	NEG	NEG	NEG	NEG
Iasi-52-52	BSI	NEG	NEG	NEG	NEG	POS	NEG	NEG	NEG	NEG	NEG	NEG	NEG	POS	NEG	NEG	NEG	NEG	NEG	NEG	NEG	NEG	POS	NEG	NEG	NEG	NEG	NEG	NEG	POS	NEG	NEG	NEG	NEG	NEG	NEG	NEG	NEG	NEG	NEG	POS	NEG	NEG	NEG	NEG	NEG
Iasi-01-2	SSTI	NEG	NEG	NEG	NEG	POS	NEG	NEG	NEG	NEG	NEG	NEG	NEG	POS	NEG	NEG	NEG	NEG	NEG	NEG	NEG	NEG	POS	NEG	NEG	NEG	NEG	NEG	NEG	POS	NEG	NEG	NEG	NEG	NEG	NEG	NEG	NEG	NEG	NEG	POS	NEG	NEG	NEG	NEG	NEG
Iasi-10-101	SSTI	NEG	NEG	NEG	NEG	POS	NEG	NEG	NEG	NEG	NEG	NEG	NEG	POS	NEG	NEG	POS	POS	NEG	NEG	NEG	NEG	POS	NEG	NEG	NEG	NEG	NEG	NEG	POS	NEG	NEG	NEG	NEG	NEG	NEG	NEG	NEG	NEG	NEG	POS	NEG	NEG	NEG	NEG	NEG
Iasi-11-106	SSTI	NEG	NEG	NEG	NEG	POS	NEG	NEG	NEG	NEG	NEG	NEG	NEG	POS	NEG	NEG	NEG	NEG	NEG	NEG	NEG	NEG	POS	NEG	NEG	NEG	NEG	NEG	NEG	POS	NEG	NEG	NEG	NEG	NEG	NEG	NEG	NEG	NEG	NEG	POS	NEG	NEG	NEG	NEG	NEG
Iasi-49-414	SSTI	NEG	NEG	NEG	NEG	POS	NEG	NEG	NEG	NEG	NEG	NEG	NEG	POS	AMB	NEG	NEG	NEG	NEG	NEG	NEG	NEG	POS	NEG	NEG	NEG	NEG	NEG	NEG	POS	NEG	NEG	NEG	NEG	NEG	NEG	NEG	NEG	NEG	NEG	POS	NEG	NEG	NEG	NEG	NEG

Isolate	Group	RESISTANCE : MISCELLANEOUS GENES														RESISTANCE : EFFLUX SYSTEMS								RESISTANCE : GLYCOPEPTIDES			VIRULENCE : TOX.SCHOCK.TOXIN			VIRULENCE : ENTEROTOXINS																
		fusidic acid resistence	hypothetical protein associated with fusidic acid resistance	mupirocin resistence protein	tetrazyklin-resistance		chloramphenicol acetyltransferase					23S rRNA methyltransferase	chloramphenicol/florfenicol exporter	metallothiol transferase		quaternary ammonium compound resistance protein A	quaternary ammonium compound resistance protein C						Transport-/Effluxprotein	vancomycin resistance gene	vancomycin resistance gene from enterococci and Clostridium	teicoplanin resistance gene from enterococci	toxic shock syndrome toxin 1			enterotoxin A	enterotoxin A, allele from strain 320E	enterotoxin A, allele from strain N315 = entero-toxin P	enterotoxin B	enterotoxin C	enterotoxin D	enterotoxin E	enterotoxin G	enterotoxin H	enterotoxin I	enterotoxin J	enterotoxin K	enterotoxin L	enterotoxin M	enterotoxin N		enterotoxin O
		far1	fusC (Q6GD50)	mupA	tet(K)	tet(M)	cat (total)	cat (pC221)	cat (pc223)	cat (pMC524/pC194)	cat (pSBK203R)	cfr	fexA	fosB	fosB (plasmid)	qacA	qacC (total)	qacC (consensus)	qacC (equine)	qacC (SA5)	qacC (Ssap)	qacC (ST94)	sdrM	vanA	vanB	vanZ	tst1 (consensus)	tst1 ("human" allele)	tst1 ("bovine" allele)	sea	sea (320E)	sea (N315) / sep	seb	sec	sed	see	seg	seh	sei	sej	sek	sel	selm	seln (consensus)	seln (other than RF122)	selo
Iasi-05-46	misc. MRSA	NEG	NEG	NEG	NEG	POS	NEG	NEG	NEG	NEG	NEG	NEG	NEG	POS	NEG	NEG	NEG	NEG	NEG	NEG	NEG	NEG	POS	NEG	NEG	NEG	NEG	NEG	NEG	POS	NEG	NEG	NEG	NEG	NEG	NEG	NEG	NEG	NEG	NEG	POS	NEG	NEG	NEG	NEG	NEG
Iasi-06-47	misc. MRSA	NEG	NEG	NEG	NEG	POS	NEG	NEG	NEG	NEG	NEG	NEG	NEG	POS	NEG	NEG	NEG	NEG	NEG	NEG	NEG	NEG	POS	NEG	NEG	NEG	NEG	NEG	NEG	POS	NEG	NEG	NEG	NEG	NEG	NEG	NEG	NEG	NEG	NEG	POS	NEG	NEG	NEG	NEG	NEG
Iasi-08-73	misc. MRSA	NEG	NEG	NEG	NEG	POS	NEG	NEG	NEG	NEG	NEG	NEG	NEG	POS	NEG	NEG	NEG	NEG	NEG	NEG	NEG	NEG	POS	NEG	NEG	NEG	NEG	NEG	NEG	POS	NEG	NEG	NEG	NEG	NEG	NEG	NEG	NEG	NEG	NEG	POS	NEG	NEG	NEG	NEG	NEG
Iasi-19-194	misc. MRSA	NEG	NEG	NEG	NEG	POS	NEG	NEG	NEG	NEG	NEG	NEG	NEG	POS	NEG	NEG	NEG	NEG	NEG	NEG	NEG	NEG	POS	NEG	NEG	NEG	NEG	NEG	NEG	POS	NEG	NEG	NEG	NEG	NEG	NEG	NEG	NEG	NEG	NEG	POS	NEG	NEG	NEG	NEG	NEG
Iasi-21-219	misc. MRSA	NEG	NEG	NEG	NEG	POS	NEG	NEG	NEG	NEG	NEG	NEG	NEG	POS	NEG	NEG	NEG	NEG	NEG	NEG	NEG	NEG	POS	NEG	NEG	NEG	NEG	NEG	NEG	POS	NEG	NEG	NEG	NEG	NEG	NEG	NEG	NEG	NEG	NEG	POS	NEG	NEG	NEG	NEG	NEG
Iasi-25-284	misc. MRSA	NEG	NEG	NEG	NEG	POS	NEG	NEG	NEG	NEG	NEG	NEG	NEG	POS	NEG	NEG	NEG	NEG	NEG	NEG	NEG	NEG	POS	NEG	NEG	NEG	NEG	NEG	NEG	POS	NEG	NEG	NEG	NEG	NEG	NEG	NEG	NEG	NEG	NEG	POS	NEG	NEG	NEG	NEG	NEG
Iasi-284	misc. MRSA	NEG	NEG	NEG	NEG	POS	NEG	NEG	NEG	NEG	NEG	NEG	NEG	POS	NEG	NEG	NEG	NEG	NEG	NEG	NEG	NEG	POS	NEG	NEG	NEG	NEG	NEG	NEG	POS	NEG	NEG	NEG	NEG	NEG	NEG	NEG	NEG	NEG	NEG	POS	NEG	NEG	NEG	NEG	NEG
Iasi-36-336	misc. MRSA	NEG	NEG	NEG	NEG	POS	NEG	NEG	NEG	NEG	NEG	NEG	NEG	POS	NEG	NEG	NEG	NEG	NEG	NEG	NEG	NEG	POS	NEG	NEG	NEG	NEG	NEG	NEG	POS	NEG	NEG	NEG	NEG	NEG	NEG	NEG	NEG	NEG	NEG	POS	NEG	NEG	NEG	NEG	NEG
Iasi-43-402	misc. MRSA	NEG	NEG	NEG	NEG	POS	NEG	NEG	NEG	NEG	NEG	NEG	NEG	POS	NEG	NEG	NEG	NEG	NEG	NEG	NEG	NEG	POS	NEG	NEG	NEG	NEG	NEG	NEG	POS	NEG	NEG	NEG	NEG	NEG	NEG	NEG	NEG	NEG	NEG	POS	NEG	NEG	NEG	NEG	NEG
Iasi-44-404	misc. MRSA	NEG	NEG	NEG	NEG	POS	NEG	NEG	NEG	NEG	NEG	NEG	NEG	POS	NEG	NEG	NEG	NEG	NEG	NEG	NEG	NEG	POS	NEG	NEG	NEG	NEG	NEG	NEG	POS	NEG	NEG	NEG	NEG	NEG	NEG	NEG	NEG	NEG	NEG	POS	NEG	NEG	NEG	NEG	NEG
Iasi-54-430	misc. MRSA	NEG	NEG	NEG	NEG	POS	NEG	NEG	NEG	NEG	NEG	NEG	NEG	POS	AMB	NEG	NEG	NEG	NEG	NEG	NEG	NEG	POS	NEG	NEG	NEG	NEG	AMB	NEG	POS	AMB	NEG	NEG	NEG	NEG	NEG	NEG	NEG	NEG	NEG	POS	NEG	NEG	NEG	NEG	NEG
Iasi-24-216	SSTI	NEG	NEG	NEG	NEG	NEG	NEG	NEG	NEG	NEG	NEG	NEG	NEG	NEG	NEG	NEG	NEG	NEG	NEG	NEG	NEG	NEG	POS	NEG	NEG	NEG	NEG	NEG	NEG	NEG	NEG	NEG	NEG	NEG	NEG	NEG	POS	NEG	POS	NEG	NEG	NEG	POS	POS	POS	POS
Iasi-05-S13	Carrier	NEG	NEG	NEG	NEG	NEG	NEG	NEG	NEG	NEG	NEG	NEG	NEG	NEG	NEG	NEG	NEG	NEG	NEG	NEG	NEG	NEG	POS	NEG	NEG	NEG	NEG	NEG	NEG	NEG	NEG	NEG	NEG	NEG	NEG	NEG	POS	NEG	POS	NEG	NEG	NEG	POS	POS	POS	POS
Iasi-45-362	BSI	NEG	NEG	NEG	NEG	NEG	NEG	NEG	NEG	NEG	NEG	NEG	NEG	POS	NEG	NEG	NEG	NEG	NEG	NEG	NEG	NEG	POS	NEG	NEG	NEG	NEG	NEG	NEG	NEG	NEG	NEG	NEG	NEG	NEG	NEG	NEG	NEG	NEG	NEG	NEG	NEG	NEG	NEG	NEG	NEG
Iasi-22-199	SSTI	NEG	NEG	NEG	POS	NEG	NEG	NEG	NEG	NEG	NEG	NEG	NEG	POS	NEG	NEG	NEG	NEG	NEG	NEG	NEG	NEG	POS	NEG	NEG	NEG	NEG	NEG	NEG	NEG	NEG	NEG	NEG	NEG	NEG	NEG	NEG	NEG	NEG	NEG	NEG	NEG	NEG	NEG	NEG	NEG
Iasi-12-S26	Carrier	NEG	NEG	NEG	NEG	NEG	NEG	NEG	NEG	NEG	NEG	NEG	NEG	POS	AMB	NEG	NEG	NEG	NEG	NEG	NEG	NEG	POS	NEG	NEG	NEG	NEG	NEG	NEG	NEG	NEG	NEG	NEG	NEG	NEG	NEG	NEG	NEG	NEG	NEG	NEG	NEG	NEG	NEG	NEG	NEG
Iasi-21-P2	Carrier	NEG	NEG	NEG	POS	NEG	NEG	NEG	NEG	NEG	NEG	NEG	NEG	POS	AMB	NEG	NEG	NEG	NEG	NEG	NEG	NEG	POS	NEG	NEG	NEG	NEG	NEG	NEG	NEG	NEG	NEG	NEG	NEG	NEG	NEG	NEG	NEG	NEG	NEG	NEG	NEG	NEG	NEG	NEG	NEG
Iasi-06-60	BSI	NEG	NEG	NEG	NEG	NEG	NEG	NEG	NEG	NEG	NEG	NEG	NEG	NEG	NEG	NEG	NEG	NEG	NEG	NEG	NEG	NEG	NEG	NEG	NEG	NEG	NEG	NEG	NEG	NEG	NEG	NEG	NEG	NEG	NEG	NEG	POS	NEG	POS	NEG	NEG	NEG	POS	POS	POS	POS
Iasi-12-79	BSI	NEG	NEG	NEG	NEG	NEG	NEG	NEG	NEG	NEG	NEG	NEG	NEG	NEG	NEG	NEG	NEG	NEG	NEG	NEG	NEG	NEG	NEG	NEG	NEG	NEG	NEG	NEG	NEG	NEG	NEG	NEG	NEG	NEG	NEG	NEG	POS	NEG	POS	NEG	NEG	NEG	POS	POS	POS	POS
Iasi-17-90	BSI	NEG	NEG	NEG	NEG	NEG	NEG	NEG	NEG	NEG	NEG	NEG	NEG	NEG	NEG	NEG	NEG	NEG	NEG	NEG	NEG	NEG	NEG	NEG	NEG	NEG	NEG	NEG	NEG	NEG	NEG	NEG	NEG	NEG	NEG	NEG	POS	NEG	POS	NEG	NEG	NEG	POS	POS	POS	POS
Iasi-21-105	BSI	NEG	NEG	NEG	NEG	NEG	NEG	NEG	NEG	NEG	NEG	NEG	NEG	NEG	NEG	NEG	NEG	NEG	NEG	NEG	NEG	NEG	NEG	NEG	NEG	NEG	NEG	NEG	NEG	NEG	NEG	NEG	NEG	NEG	NEG	NEG	POS	NEG	POS	NEG	NEG	NEG	POS	POS	POS	POS
Iasi-22-109	BSI	NEG	NEG	NEG	NEG	NEG	NEG	NEG	NEG	NEG	NEG	NEG	NEG	AMB	NEG	NEG	NEG	NEG	NEG	NEG	NEG	NEG	NEG	NEG	NEG	NEG	NEG	NEG	NEG	NEG	NEG	NEG	NEG	NEG	NEG	NEG	POS	NEG	POS	NEG	NEG	NEG	POS	POS	POS	POS
Iasi-23-110	BSI	NEG	NEG	NEG	NEG	NEG	NEG	NEG	NEG	NEG	NEG	NEG	NEG	NEG	NEG	NEG	NEG	NEG	NEG	NEG	NEG	NEG	NEG	NEG	NEG	NEG	NEG	NEG	NEG	NEG	NEG	NEG	NEG	NEG	NEG	NEG	POS	NEG	POS	NEG	NEG	NEG	POS	POS	POS	POS
Iasi-24-136	BSI	NEG	NEG	NEG	NEG	NEG	NEG	NEG	NEG	NEG	NEG	NEG	NEG	NEG	NEG	NEG	NEG	NEG	NEG	NEG	NEG	NEG	NEG	NEG	NEG	NEG	NEG	NEG	NEG	NEG	NEG	NEG	NEG	NEG	NEG	NEG	POS	NEG	POS	NEG	NEG	NEG	POS	POS	POS	POS
Iasi-34-224	BSI	NEG	NEG	NEG	NEG	NEG	NEG	NEG	NEG	NEG	NEG	NEG	NEG	NEG	NEG	NEG	NEG	NEG	NEG	NEG	NEG	NEG	NEG	NEG	NEG	NEG	POS	POS	NEG	NEG	NEG	NEG	NEG	NEG	NEG	NEG	POS	NEG	POS	NEG	NEG	NEG	POS	POS	POS	POS
Iasi-39-297	BSI	NEG	NEG	NEG	POS	NEG	NEG	NEG	NEG	NEG	NEG	NEG	NEG	AMB	NEG	NEG	NEG	NEG	NEG	NEG	NEG	NEG	NEG	NEG	NEG	NEG	NEG	NEG	NEG	NEG	NEG	NEG	NEG	NEG	NEG	NEG	POS	NEG	POS	NEG	NEG	NEG	POS	POS	POS	POS
Iasi-50-431	BSI	NEG	NEG	NEG	NEG	NEG	NEG	NEG	NEG	NEG	NEG	NEG	NEG	NEG	NEG	NEG	NEG	NEG	NEG	NEG	NEG	NEG	NEG	NEG	NEG	NEG	NEG	NEG	NEG	NEG	NEG	NEG	NEG	NEG	NEG	NEG	POS	NEG	POS	NEG	NEG	NEG	POS	POS	POS	POS
Iasi-51-432	BSI	NEG	NEG	NEG	NEG	NEG	NEG	NEG	NEG	NEG	NEG	NEG	NEG	NEG	NEG	NEG	NEG	NEG	NEG	NEG	NEG	NEG	NEG	NEG	NEG	NEG	NEG	NEG	NEG	NEG	NEG	NEG	NEG	NEG	NEG	NEG	POS	NEG	POS	NEG	NEG	NEG	POS	POS	POS	POS
Iasi-08-76	SSTI	NEG	NEG	NEG	NEG	NEG	NEG	NEG	NEG	NEG	NEG	NEG	NEG	NEG	NEG	NEG	NEG	NEG	NEG	NEG	NEG	NEG	NEG	NEG	NEG	NEG	NEG	NEG	NEG	NEG	NEG	NEG	NEG	NEG	NEG	NEG	POS	NEG	POS	NEG	NEG	NEG	POS	POS	POS	POS
Iasi-30-350	SSTI	NEG	NEG	NEG	NEG	NEG	NEG	NEG	NEG	NEG	NEG	NEG	NEG	NEG	NEG	NEG	NEG	NEG	NEG	NEG	NEG	NEG	NEG	NEG	NEG	NEG	NEG	NEG	NEG	NEG	NEG	NEG	NEG	NEG	NEG	NEG	POS	NEG	POS	NEG	NEG	NEG	POS	POS	POS	POS
Iasi-33-319	SSTI	NEG	NEG	NEG	NEG	NEG	NEG	NEG	NEG	NEG	NEG	NEG	NEG	NEG	NEG	NEG	NEG	NEG	NEG	NEG	NEG	NEG	NEG	NEG	NEG	NEG	NEG	NEG	NEG	NEG	NEG	NEG	NEG	NEG	NEG	NEG	POS	NEG	POS	NEG	NEG	NEG	AMB	POS	POS	POS
Iasi-40-383	SSTI	NEG	NEG	NEG	NEG	NEG	NEG	NEG	NEG	NEG	NEG	NEG	NEG	NEG	NEG	NEG	NEG	NEG	NEG	NEG	NEG	NEG	NEG	NEG	NEG	NEG	NEG	NEG	NEG	NEG	NEG	NEG	NEG	NEG	NEG	NEG	POS	NEG	POS	NEG	NEG	NEG	POS	POS	POS	POS
Iasi-49-423	SSTI	NEG	NEG	NEG	NEG	NEG	NEG	NEG	NEG	NEG	NEG	NEG	NEG	NEG	NEG	NEG	NEG	NEG	NEG	NEG	NEG	NEG	NEG	NEG	NEG	NEG	NEG	NEG	NEG	NEG	NEG	NEG	NEG	NEG	NEG	NEG	POS	NEG	POS	NEG	NEG	NEG	POS	POS	POS	POS
Iasi-16-S31	Carrier	NEG	NEG	NEG	NEG	NEG	NEG	NEG	NEG	NEG	NEG	NEG	NEG	NEG	NEG	NEG	NEG	NEG	NEG	NEG	NEG	NEG	NEG	NEG	NEG	NEG	NEG	NEG	NEG	NEG	NEG	NEG	NEG	NEG	NEG	NEG	POS	NEG	POS	NEG	NEG	NEG	POS	POS	POS	POS
Iasi-24-P9	Carrier	NEG	NEG	NEG	NEG	NEG	NEG	NEG	NEG	NEG	NEG	NEG	NEG	NEG	NEG	NEG	NEG	NEG	NEG	NEG	NEG	NEG	NEG	NEG	NEG	NEG	NEG	NEG	NEG	NEG	NEG	NEG	NEG	NEG	NEG	NEG	POS	NEG	POS	NEG	NEG	NEG	POS	POS	POS	POS
Iasi-27-P12	Carrier	NEG	NEG	NEG	NEG	NEG	NEG	NEG	NEG	NEG	NEG	NEG	NEG	NEG	NEG	NEG	NEG	NEG	NEG	NEG	NEG	NEG	NEG	NEG	NEG	NEG	NEG	NEG	NEG	NEG	NEG	NEG	NEG	NEG	NEG	NEG	POS	NEG	POS	NEG	NEG	NEG	POS	POS	POS	POS
Iasi-38-P27	Carrier	NEG	NEG	NEG	NEG	NEG	NEG	NEG	NEG	NEG	NEG	NEG	NEG	AMB	NEG	NEG	NEG	NEG	NEG	NEG	NEG	NEG	NEG	NEG	NEG	NEG	NEG	NEG	NEG	NEG	NEG	NEG	NEG	NEG	NEG	NEG	POS	NEG	POS	NEG	NEG	NEG	POS	POS	POS	POS
Iasi-42-G511	Carrier	NEG	NEG	NEG	NEG	NEG	NEG	NEG	NEG	NEG	NEG	NEG	NEG	NEG	NEG	NEG	NEG	NEG	NEG	NEG	NEG	NEG	NEG	NEG	NEG	NEG	NEG	NEG	NEG	NEG	NEG	NEG	NEG	NEG	NEG	NEG	POS	NEG	POS	NEG	NEG	NEG	POS	POS	POS	POS
Iasi-46-3N	Carrier	NEG	NEG	NEG	NEG	NEG	NEG	NEG	NEG	NEG	NEG	NEG	NEG	AMB	NEG	NEG	NEG	NEG	NEG	NEG	NEG	NEG	NEG	NEG	NEG	NEG	NEG	NEG	NEG	NEG	NEG	NEG	NEG	NEG	NEG	NEG	POS	NEG	POS	NEG	NEG	NEG	POS	POS	POS	POS
Iasi-47-42O	Carrier	NEG	NEG	NEG	NEG	NEG	NEG	NEG	NEG	NEG	NEG	NEG	NEG	NEG	NEG	NEG	NEG	NEG	NEG	NEG	NEG	NEG	NEG	NEG	NEG	NEG	NEG	NEG	NEG	NEG	NEG	NEG	NEG	NEG	NEG	NEG	POS	NEG	POS	NEG	NEG	NEG	POS	POS	POS	POS
Iasi-48-36N	Carrier	NEG	NEG	NEG	NEG	NEG	NEG	NEG	NEG	NEG	NEG	NEG	NEG	NEG	NEG	NEG	NEG	NEG	NEG	NEG	NEG	NEG	NEG	NEG	NEG	NEG	NEG	NEG	NEG	NEG	NEG	NEG	NEG	NEG	NEG	NEG	POS	NEG	POS	NEG	NEG	NEG	POS	POS	POS	POS
Iasi-49-T3	Carrier	NEG	NEG	NEG	NEG	NEG	NEG	NEG	NEG	NEG	NEG	NEG	NEG	NEG	NEG	NEG	NEG	NEG	NEG	NEG	NEG	NEG	NEG	NEG	NEG	NEG	NEG	NEG	NEG	NEG	NEG	NEG	NEG	NEG	NEG	NEG	POS	NEG	POS	NEG	NEG	NEG	POS	POS	POS	POS
Iasi-50-T2	Carrier	NEG	NEG	NEG	NEG	NEG	NEG	NEG	NEG	NEG	NEG	NEG	NEG	NEG	NEG	NEG	NEG	NEG	NEG	NEG	NEG	NEG	NEG	NEG	NEG	NEG	NEG	AMB	NEG	NEG	NEG	NEG	NEG	NEG	NEG	NEG	POS	NEG	POS	NEG	NEG	NEG	POS	POS	POS	POS
Iasi-22-251	BSI	NEG	NEG	NEG	NEG	NEG	NEG	NEG	NEG	NEG	NEG	NEG	NEG	NEG	NEG	NEG	NEG	NEG	NEG	NEG	NEG	NEG	NEG	NEG	NEG	NEG	NEG	NEG	NEG	NEG	NEG	NEG	NEG	NEG	NEG	NEG	POS	NEG	POS	NEG	NEG	NEG	POS	POS	POS	POS
Iasi-27-158	BSI	NEG	NEG	NEG	NEG	NEG	NEG	NEG	NEG	NEG	NEG	NEG	NEG	POS	POS	NEG	NEG	NEG	NEG	NEG	NEG	NEG	POS	NEG	NEG	NEG	AMB	NEG	NEG	NEG	NEG	NEG	POS	POS	NEG	NEG	POS	NEG	POS	NEG	NEG	POS	POS	POS	POS	POS
Iasi-08-70	BSI	NEG	NEG	NEG	NEG	NEG	NEG	NEG	NEG	NEG	NEG	NEG	NEG	POS	NEG	NEG	NEG	NEG	NEG	NEG	NEG	NEG	POS	NEG	NEG	NEG	NEG	NEG	NEG	NEG	NEG	NEG	NEG	NEG	NEG	NEG	POS	NEG	POS	NEG	NEG	NEG	POS	POS	POS	POS
Iasi-18-93	BSI	NEG	NEG	NEG	NEG	NEG	NEG	NEG	NEG	NEG	NEG	NEG	NEG	POS	NEG	NEG	NEG	NEG	NEG	NEG	NEG	NEG	POS	NEG	NEG	NEG	NEG	NEG	NEG	POS	NEG	NEG	NEG	NEG	NEG	NEG	POS	NEG	POS	NEG	NEG	NEG	POS	POS	POS	POS
Iasi-28-P14	Carrier	NEG	NEG	NEG	NEG	NEG	NEG	NEG	NEG	NEG	NEG	NEG	NEG	POS	NEG	NEG	NEG	NEG	NEG	NEG	NEG	NEG	POS	NEG	NEG	NEG	POS	POS	NEG	POS	NEG	NEG	NEG	NEG	NEG	NEG	POS	NEG	POS	NEG	NEG	NEG	POS	POS	POS	POS
Iasi-29-P15	Carrier	NEG	NEG	NEG	NEG	NEG	NEG	NEG	NEG	NEG	NEG	NEG	NEG	POS	AMB	NEG	NEG	NEG	NEG	NEG	NEG	NEG	POS	NEG	NEG	NEG	POS	POS	NEG	POS	NEG	NEG	NEG	NEG	NEG	NEG	POS	NEG	POS	NEG	NEG	NEG	POS	POS	POS	POS
Iasi-03-19	BSI	NEG	NEG	NEG	NEG	NEG	NEG	NEG	NEG	NEG	NEG	NEG	NEG	NEG	NEG	NEG	NEG	NEG	NEG	NEG	NEG	NEG	POS	NEG	NEG	NEG	POS	POS	NEG	NEG	NEG	NEG	NEG	POS	NEG	NEG	POS	NEG	POS	NEG	NEG	POS	POS	POS	POS	POS
Iasi-11-77	BSI	NEG	NEG	NEG	NEG	NEG	NEG	NEG	NEG	NEG	NEG	NEG	NEG	NEG	NEG	NEG	NEG	NEG	NEG	NEG	NEG	NEG	POS	NEG	NEG	NEG	NEG	NEG	NEG	NEG	NEG	NEG	NEG	POS	NEG	NEG	POS	NEG	POS	NEG	NEG	POS	POS	POS	POS	POS
Iasi-13-82	BSI	NEG	NEG	NEG	NEG	NEG	NEG	NEG	NEG	NEG	NEG	NEG	NEG	NEG	NEG	NEG	NEG	NEG	NEG	NEG	NEG	NEG	POS	NEG	NEG	NEG	POS	POS	NEG	NEG	NEG	NEG	NEG	POS	NEG	NEG	POS	NEG	POS	NEG	NEG	POS	POS	POS	POS	POS
Iasi-15-86	BSI	NEG	NEG	NEG	NEG	NEG	NEG	NEG	NEG	NEG	NEG	NEG	NEG	NEG	NEG	NEG	NEG	NEG	NEG	NEG	NEG	NEG	POS	NEG	NEG	NEG	NEG	NEG	NEG	NEG	NEG	NEG	NEG	POS	NEG	NEG	POS	NEG	POS	NEG	NEG	POS	POS	POS	POS	POS
Iasi-20-103	BSI	NEG	NEG	NEG	POS	NEG	NEG	NEG	NEG	NEG	NEG	NEG	NEG	NEG	NEG	NEG	NEG	NEG	NEG	NEG	NEG	NEG	POS	NEG	NEG	NEG	NEG	NEG	NEG	NEG	NEG	NEG	NEG	POS	NEG	NEG	POS	NEG	POS	NEG	NEG	POS	POS	POS	POS	POS
Iasi-33-222	BSI	NEG	NEG	NEG	POS	NEG	NEG	NEG	NEG	NEG	NEG	NEG	NEG	AMB	NEG	NEG	NEG	NEG	NEG	NEG	NEG	NEG	POS	NEG	NEG	NEG	POS	POS	NEG	NEG	NEG	NEG	NEG	POS	NEG	NEG	POS	NEG	POS	NEG	NEG	POS	POS	POS	POS	POS
Iasi-44-360	BSI	NEG	NEG	NEG	NEG	NEG	NEG	NEG	NEG	NEG	NEG	NEG	NEG	NEG	NEG	NEG	NEG	NEG	NEG	NEG	NEG	NEG	POS	NEG	NEG	NEG	POS	POS	NEG	NEG	NEG	NEG	NEG	POS	NEG	NEG	POS	NEG	POS	NEG	NEG	POS	POS	POS	POS	POS
Iasi-47-399	BSI	NEG	NEG	NEG	NEG	NEG	NEG	NEG	NEG	NEG	NEG	NEG	NEG	AMB	NEG	NEG	NEG	NEG	NEG	NEG	NEG	NEG	POS	NEG	NEG	NEG	NEG	NEG	NEG	NEG	NEG	NEG	NEG	NEG	NEG	NEG	POS	NEG	POS	NEG	NEG	NEG	POS	POS	POS	POS
Iasi-34-368	SSTI	NEG	NEG	NEG	NEG	NEG	NEG	NEG	NEG	NEG	NEG	NEG	NEG	NEG	NEG	NEG	NEG	NEG	NEG	NEG	NEG	NEG	POS	NEG	NEG	NEG	NEG	NEG	NEG	NEG	NEG	NEG	NEG	NEG	NEG	NEG	POS	NEG	POS	NEG	NEG	NEG	POS	POS	POS	POS
Iasi-36-371	SSTI	NEG	NEG	NEG	POS	NEG	NEG	NEG	NEG	NEG	NEG	NEG	NEG	NEG	NEG	NEG	NEG	NEG	NEG	NEG	NEG	NEG	POS	NEG	NEG	NEG	NEG	NEG	NEG	NEG	NEG	NEG	NEG	POS	NEG	NEG	POS	NEG	POS	NEG	NEG	POS	POS	POS	POS	POS
Iasi-04-S12	Carrier	NEG	NEG	NEG	POS	NEG	NEG	NEG	NEG	NEG	NEG	NEG	NEG	NEG	NEG	NEG	NEG	NEG	NEG	NEG	NEG	NEG	POS	NEG	NEG	NEG	NEG	NEG	NEG	NEG	NEG	NEG	NEG	POS	NEG	NEG	POS	NEG	POS	NEG	NEG	POS	POS	POS	POS	POS
Iasi-06-S15	Carrier	NEG	NEG	NEG	NEG	NEG	NEG	NEG	NEG	NEG	NEG	NEG	NEG	NEG	NEG	NEG	NEG	NEG	NEG	NEG	NEG	NEG	POS	NEG	NEG	NEG	NEG	NEG	NEG	NEG	NEG	NEG	NEG	POS	NEG	NEG	POS	NEG	POS	NEG	NEG	POS	POS	POS	POS	POS
Iasi-07-S16	Carrier	NEG	NEG	NEG	NEG	NEG	NEG	NEG	NEG	NEG	NEG	NEG	NEG	NEG	NEG	NEG	NEG	NEG	NEG	NEG	NEG	NEG	POS	NEG	NEG	NEG	NEG	NEG	NEG	NEG	NEG	NEG	NEG	POS	NEG	NEG	POS	NEG	POS	NEG	NEG	POS	POS	POS	POS	POS
Iasi-11-S24	Carrier	NEG	NEG	NEG	NEG	NEG	NEG	NEG	NEG	NEG	NEG	NEG	NEG	AMB	NEG	NEG	NEG	NEG	NEG	NEG	NEG	NEG	POS	NEG	NEG	NEG	POS	POS	NEG	NEG	NEG	NEG	NEG	POS	NEG	NEG	POS	NEG	POS	NEG	NEG	POS	POS	POS	POS	POS
Iasi-22-P4	Carrier	NEG	NEG	NEG	NEG	NEG	NEG	NEG	NEG	NEG	NEG	NEG	NEG	NEG	NEG	NEG	NEG	NEG	NEG	NEG	NEG	NEG	POS	NEG	NEG	NEG	NEG	NEG	NEG	NEG	NEG	NEG	NEG	POS	NEG	NEG	POS	NEG	POS	NEG	NEG	POS	POS	POS	POS	POS
Iasi-34-P23	Carrier	NEG	NEG	NEG	NEG	NEG	NEG	NEG	NEG	NEG	NEG	NEG	NEG	NEG	NEG	NEG	NEG	NEG	NEG	NEG	NEG	NEG	POS	NEG	NEG	NEG	NEG	NEG	NEG	NEG	NEG	NEG	NEG	NEG	NEG	NEG	POS	NEG	POS	NEG	NEG	NEG	POS	POS	POS	POS
Iasi-37-P26	Carrier	NEG	NEG	NEG	NEG	NEG	NEG	NEG	NEG	NEG	NEG	NEG	NEG	AMB	NEG	NEG	NEG	NEG	NEG	NEG	NEG	NEG	POS	NEG	NEG	NEG	NEG	NEG	NEG	NEG	NEG	NEG	NEG	POS	NEG	NEG	POS	NEG	POS	NEG	NEG	POS	POS	POS	POS	POS
Iasi-30-196	BSI	NEG	NEG	NEG	NEG	NEG	NEG	NEG	NEG	NEG	NEG	NEG	AMB	POS	NEG	NEG	NEG	NEG	NEG	NEG	NEG	NEG	POS	NEG	NEG	NEG	AMB	NEG	NEG	NEG	NEG	NEG	POS	POS	NEG	NEG	POS	NEG	POS	NEG	NEG	POS	POS	POS	POS	POS
Iasi-27-298	BSI	POS	NEG	NEG	POS	NEG	NEG	NEG	NEG	NEG	NEG	NEG	NEG	NEG	NEG	NEG	NEG	NEG	NEG	NEG	NEG	NEG	POS	NEG	NEG	NEG	NEG	NEG	NEG	NEG	NEG	NEG	NEG	NEG	NEG	NEG	NEG	NEG	NEG	NEG	NEG	NEG	NEG	NEG	NEG	NEG
Iasi-37-363	BSI	NEG	NEG	NEG	POS	NEG	NEG	NEG	NEG	NEG	NEG	NEG	NEG	NEG	NEG	NEG	NEG	NEG	NEG	NEG	NEG	NEG	POS	NEG	NEG	NEG	NEG	NEG	NEG	NEG	NEG	NEG	NEG	NEG	NEG	NEG	NEG	NEG	NEG	NEG	NEG	NEG	NEG	NEG	NEG	NEG
Iasi-38-372	BSI	POS	NEG	NEG	POS	NEG	NEG	NEG	NEG	NEG	NEG	NEG	NEG	NEG	NEG	NEG	NEG	NEG	NEG	NEG	NEG	NEG	POS	NEG	NEG	NEG	NEG	NEG	NEG	NEG	NEG	NEG	NEG	NEG	NEG	NEG	NEG	NEG	NEG	NEG	NEG	NEG	NEG	NEG	NEG	NEG
Iasi-39-374	BSI	POS	NEG	NEG	POS	NEG	NEG	NEG	NEG	NEG	NEG	NEG	NEG	NEG	NEG	NEG	NEG	NEG	NEG	NEG	NEG	NEG	POS	NEG	NEG	NEG	NEG	NEG	NEG	NEG	NEG	NEG	NEG	NEG	NEG	NEG	NEG	NEG	NEG	NEG	NEG	NEG	NEG	NEG	NEG	NEG
Iasi-28-299	SSTI	POS	NEG	NEG	POS	NEG	NEG	NEG	NEG	NEG	NEG	NEG	NEG	NEG	NEG	NEG	NEG	NEG	NEG	NEG	NEG	NEG	POS	NEG	NEG	NEG	NEG	NEG	NEG	NEG	NEG	NEG	NEG	NEG	NEG	NEG	NEG	NEG	NEG	NEG	NEG	NEG	NEG	NEG	NEG	NEG
Iasi-07-65	BSI	NEG	NEG	NEG	NEG	NEG	NEG	NEG	NEG	NEG	NEG	NEG	NEG	NEG	NEG	NEG	NEG	NEG	NEG	NEG	NEG	NEG	POS	NEG	NEG	NEG	NEG	NEG	NEG	NEG	NEG	NEG	NEG	POS	NEG	NEG	NEG	NEG	NEG	NEG	NEG	POS	NEG	NEG	NEG	NEG
Iasi-19-99	BSI	NEG	NEG	NEG	NEG	NEG	NEG	NEG	NEG	NEG	NEG	NEG	NEG	NEG	NEG	NEG	NEG	NEG	NEG	NEG	NEG	NEG	POS	NEG	NEG	NEG	NEG	NEG	NEG	NEG	NEG	NEG	NEG	NEG	NEG	NEG	NEG	NEG	NEG	NEG	NEG	NEG	NEG	NEG	NEG	NEG
Iasi-25-217	SSTI	NEG	NEG	NEG	NEG	NEG	NEG	NEG	NEG	NEG	NEG	NEG	NEG	NEG	NEG	NEG	NEG	NEG	NEG	NEG	NEG	NEG	POS	NEG	NEG	NEG	NEG	NEG	NEG	NEG	NEG	NEG	NEG	NEG	NEG	NEG	NEG	NEG	NEG	NEG	NEG	NEG	NEG	NEG	NEG	NEG
Iasi-23-P5	Carrier	NEG	NEG	NEG	NEG	NEG	NEG	NEG	NEG	NEG	NEG	NEG	NEG	NEG	NEG	NEG	NEG	NEG	NEG	NEG	NEG	NEG	POS	NEG	NEG	NEG	NEG	NEG	NEG	NEG	NEG	NEG	NEG	NEG	NEG	NEG	NEG	NEG	NEG	NEG	NEG	NEG	NEG	NEG	NEG	NEG
Iasi-43-G513	Carrier	NEG	NEG	NEG	NEG	NEG	NEG	NEG	NEG	NEG	NEG	NEG	NEG	AMB	NEG	NEG	NEG	NEG	NEG	NEG	NEG	NEG	POS	NEG	NEG	NEG	NEG	NEG	NEG	NEG	NEG	NEG	NEG	NEG	NEG	NEG	NEG	NEG	NEG	NEG	NEG	NEG	NEG	NEG	NEG	NEG
Iasi-44-G514	Carrier	NEG	NEG	NEG	NEG	NEG	NEG	NEG	NEG	NEG	NEG	NEG	NEG	AMB	NEG	NEG	NEG	NEG	NEG	NEG	NEG	NEG	POS	NEG	NEG	NEG	NEG	NEG	NEG	NEG	NEG	NEG	NEG	NEG	NEG	NEG	NEG	NEG	NEG	NEG	NEG	NEG	NEG	NEG	NEG	NEG
Iasi-28-172	BSI	NEG	NEG	NEG	NEG	NEG	NEG	NEG	NEG	NEG	NEG	NEG	NEG	POS	AMB	NEG	NEG	NEG	NEG	NEG	NEG	NEG	POS	NEG	NEG	NEG	NEG	AMB	NEG	NEG	NEG	NEG	NEG	NEG	NEG	NEG	POS	NEG	POS	NEG	NEG	NEG	POS	POS	POS	POS
Iasi-42-358	BSI	NEG	NEG	NEG	POS	NEG	NEG	NEG	NEG	NEG	NEG	NEG	AMB	POS	AMB	NEG	NEG	NEG	NEG	NEG	NEG	NEG	POS	NEG	NEG	NEG	AMB	NEG	NEG	NEG	NEG	NEG	NEG	NEG	NEG	NEG	POS	NEG	POS	NEG	NEG	NEG	POS	POS	POS	POS
Iasi-48-403	BSI	NEG	NEG	NEG	NEG	NEG	NEG	NEG	NEG	NEG	NEG	NEG	NEG	POS	NEG	NEG	NEG	NEG	NEG	NEG	NEG	NEG	POS	NEG	NEG	NEG	NEG	NEG	NEG	NEG	NEG	NEG	NEG	NEG	NEG	NEG	POS	NEG	POS	NEG	NEG	NEG	POS	POS	POS	POS
Iasi-26-153	BSI	NEG	NEG	NEG	NEG	NEG	NEG	NEG	NEG	NEG	NEG	NEG	NEG	POS	NEG	NEG	NEG	NEG	NEG	NEG	NEG	NEG	POS	NEG	NEG	NEG	NEG	NEG	NEG	NEG	NEG	NEG	NEG	NEG	NEG	NEG	POS	NEG	POS	NEG	NEG	NEG	POS	POS	POS	POS
Iasi-29-190	BSI	NEG	NEG	NEG	NEG	NEG	NEG	NEG	NEG	NEG	NEG	NEG	NEG	POS	NEG	NEG	NEG	NEG	NEG	NEG	NEG	NEG	POS	NEG	NEG	NEG	NEG	NEG	NEG	NEG	NEG	NEG	NEG	NEG	NEG	NEG	POS	NEG	POS	NEG	NEG	NEG	POS	POS	POS	POS
Iasi-31-201	BSI	NEG	NEG	NEG	NEG	NEG	NEG	NEG	NEG	NEG	NEG	NEG	NEG	POS	NEG	NEG	NEG	NEG	NEG	NEG	NEG	NEG	POS	NEG	NEG	NEG	NEG	NEG	NEG	NEG	NEG	NEG	NEG	NEG	NEG	NEG	POS	NEG	POS	NEG	NEG	NEG	POS	POS	POS	POS
Iasi-37-289	BSI	NEG	NEG	NEG	NEG	NEG	NEG	NEG	NEG	NEG	NEG	NEG	NEG	POS	AMB	NEG	NEG	NEG	NEG	NEG	NEG	NEG	POS	NEG	NEG	NEG	NEG	NEG	NEG	NEG	NEG	NEG	NEG	NEG	NEG	NEG	POS	NEG	POS	NEG	NEG	NEG	POS	POS	POS	POS
Iasi-41-334	BSI	NEG	NEG	NEG	NEG	NEG	NEG	NEG	NEG	NEG	NEG	NEG	AMB	POS	POS	NEG	NEG	NEG	NEG	NEG	NEG	NEG	POS	NEG	NEG	NEG	AMB	NEG	NEG	NEG	NEG	NEG	NEG	NEG	NEG	NEG	POS	NEG	POS	NEG	NEG	NEG	POS	POS	POS	POS
Iasi-52-433	BSI	NEG	NEG	NEG	NEG	NEG	NEG	NEG	NEG	NEG	NEG	NEG	NEG	POS	NEG	NEG	NEG	NEG	NEG	NEG	NEG	NEG	POS	NEG	NEG	NEG	NEG	NEG	NEG	NEG	NEG	NEG	NEG	NEG	NEG	NEG	POS	NEG	POS	NEG	NEG	NEG	POS	POS	POS	POS
Iasi-07-66	SSTI	NEG	NEG	NEG	NEG	NEG	NEG	NEG	NEG	NEG	NEG	NEG	NEG	POS	NEG	NEG	NEG	NEG	NEG	NEG	NEG	NEG	POS	NEG	NEG	NEG	NEG	NEG	NEG	NEG	NEG	NEG	NEG	NEG	NEG	NEG	POS	NEG	POS	NEG	NEG	NEG	POS	POS	POS	POS
Iasi-23-211	SSTI	NEG	NEG	NEG	POS	NEG	NEG	NEG	NEG	NEG	NEG	NEG	NEG	POS	NEG	NEG	NEG	NEG	NEG	NEG	NEG	NEG	POS	NEG	NEG	NEG	NEG	NEG	NEG	NEG	NEG	NEG	NEG	NEG	NEG	NEG	POS	NEG	POS	NEG	NEG	NEG	POS	POS	POS	POS
Iasi-43-401	SSTI	NEG	NEG	NEG	POS	NEG	NEG	NEG	NEG	NEG	NEG	NEG	NEG	POS	NEG	NEG	NEG	NEG	NEG	NEG	NEG	NEG	POS	NEG	NEG	NEG	NEG	NEG	NEG	NEG	NEG	NEG	NEG	NEG	NEG	NEG	POS	NEG	POS	NEG	NEG	NEG	POS	POS	POS	POS
Iasi-47-418	SSTI	NEG	NEG	NEG	NEG	NEG	NEG	NEG	NEG	NEG	NEG	NEG	NEG	POS	NEG	NEG	NEG	NEG	NEG	NEG	NEG	NEG	POS	NEG	NEG	NEG	NEG	NEG	NEG	NEG	NEG	NEG	NEG	NEG	NEG	NEG	POS	NEG	POS	NEG	NEG	NEG	POS	POS	POS	POS
Iasi-18-169	SSTI	NEG	NEG	NEG	NEG	NEG	NEG	NEG	NEG	NEG	NEG	NEG	NEG	POS	NEG	NEG	NEG	NEG	NEG	NEG	NEG	NEG	POS	NEG	NEG	NEG	NEG	NEG	NEG	NEG	NEG	NEG	NEG	NEG	NEG	NEG	NEG	NEG	NEG	NEG	NEG	NEG	NEG	NEG	NEG	NEG
Iasi-18-S34	Carrier	NEG	NEG	NEG	NEG	NEG	NEG	NEG	NEG	NEG	NEG	NEG	NEG	NEG	NEG	NEG	NEG	NEG	NEG	NEG	NEG	NEG	POS	NEG	NEG	NEG	NEG	NEG	NEG	NEG	NEG	NEG	NEG	NEG	NEG	NEG	NEG	NEG	NEG	NEG	NEG	NEG	NEG	NEG	NEG	NEG
Iasi-36-276	BSI	NEG	NEG	NEG	NEG	NEG	NEG	NEG	NEG	NEG	NEG	NEG	NEG	AMB	NEG	NEG	NEG	NEG	NEG	NEG	NEG	NEG	POS	NEG	NEG	NEG	NEG	NEG	NEG	NEG	NEG	NEG	NEG	NEG	NEG	NEG	NEG	NEG	NEG	NEG	NEG	NEG	NEG	NEG	NEG	NEG
Iasi-151	BSI	NEG	NEG	NEG	POS	POS	NEG	NEG	NEG	NEG	NEG	NEG	NEG	POS	NEG	NEG	NEG	NEG	NEG	NEG	NEG	NEG	POS	NEG	NEG	NEG	NEG	AMB	NEG	NEG	NEG	NEG	NEG	NEG	NEG	NEG	NEG	NEG	NEG	NEG	NEG	NEG	NEG	NEG	NEG	NEG
Iasi-02-11	BSI	NEG	NEG	NEG	NEG	NEG	NEG	NEG	NEG	NEG	NEG	NEG	NEG	NEG	NEG	NEG	NEG	NEG	NEG	NEG	NEG	NEG	POS	NEG	NEG	NEG	NEG	NEG	NEG	NEG	NEG	NEG	POS	NEG	NEG	NEG	NEG	NEG	POS	NEG	POS	NEG	POS	AMB	POS	POS

Isolate	Group	VIRULENCE : ENTEROTOXINS					VIRULENCE : HLG AND LEUKOCIDINS													VIRULENCE : HAEMOLYSINS								VIRULENCE : HLB-CONV PHAGES			VIRULENCE : EXFOL.TOXINS			VIRULENCE : EPITHEL. DIFF. INHIB			VIRULENCE : ACME LOCUS				VIRULENCE : PROTEASES					
		egc cluster	enterotoxin Q	enterotoxin R	enterotoxin U		haemolysin gamma / leukocidin, component B	haemolysin gamma / leukocidin, component C		haemolysin gamma	Panton Valentine leukocidin F component	Panton Valentine leukocidin S component	F component from hypothetical leukocidin from ruminants	S component from hypothetical leukocidin from ruminants	leukocidin D component	leukocidin E component	leukocidin/ haemolysin toxin family protein	leukocidin/haemolysin toxin family protein		putative membrane protein	haemolysin alpha	putative membrane protein		haemolysin beta				staphylo-kinase	chemotaxis-inhibiting protein (CHIPS)	Staphyl. Comple-ment inhibitor	exfoliative toxin serotype A	exfoliative toxin serotype B	exfoliative toxin D	epidermal cell differen-tiation inhibitor	epidermal cell differen-tiation inhibitor B	epidermal cell differen-tiation inhibitor C	ACME-locus	ACME-locus: ornithincarb-amoyltrans-ferase	ACME-locus: carbamat-kinase	ACME-locus: arginine/orni-thine-antiporter	aureolysin			serin- protease A	serin- protease B	serin- protease E
		egc (total)	seq	ser	selu	ORF CM14	lukF	lukS	lukS (ST22+ST45)	hlgA	lukF-PV	lukS-PV	lukF-PV (P83)	lukM	lukD	lukE	lukX	lukY	lukY (ST30+ST45)	hl	hla	hlIII (consensus)	hlIII (other than RF122)	hlb_probe 1	hlb_probe 2	hlb_probe 3	un-disrupted hlb	sak	chp	scn	etA	etB	etD	edinA	edinB	edinC	arcA-SCC	arcB-SCC	arcC-SCC	arcD-SCC	aur (consensus)	aur (other than MRSA252)	aur (MRSA252)	splA	splB	splE
Iasi-01-4	BSI	NEG	NEG	NEG	NEG	NEG	POS	POS	AMB	POS	NEG	NEG	NEG	NEG	POS	POS	POS	POS	NEG	POS	POS	POS	POS	POS	POS	POS	NEG	POS	NEG	POS	NEG	NEG	NEG	NEG	NEG	NEG	NEG	NEG	NEG	NEG	POS	POS	NEG	POS	POS	NEG
Iasi-05-32	BSI	NEG	NEG	NEG	NEG	NEG	POS	POS	POS	POS	NEG	NEG	NEG	NEG	POS	POS	POS	POS	NEG	POS	POS	POS	POS	POS	POS	POS	POS	NEG	NEG	NEG	NEG	NEG	NEG	NEG	NEG	NEG	NEG	NEG	NEG	NEG	POS	POS	NEG	POS	POS	NEG
Iasi-16-88	BSI	NEG	NEG	NEG	NEG	NEG	POS	POS	POS	POS	NEG	NEG	NEG	NEG	POS	POS	POS	POS	NEG	POS	POS	POS	POS	POS	POS	POS	NEG	POS	NEG	POS	NEG	NEG	NEG	NEG	NEG	NEG	NEG	NEG	NEG	NEG	POS	POS	NEG	POS	POS	NEG
Iasi-25-141	BSI	NEG	NEG	NEG	NEG	NEG	POS	POS	POS	POS	NEG	NEG	NEG	NEG	POS	POS	POS	POS	NEG	POS	POS	POS	POS	POS	POS	POS	NEG	POS	NEG	POS	NEG	NEG	NEG	NEG	NEG	NEG	NEG	NEG	NEG	NEG	POS	POS	NEG	POS	POS	NEG
Iasi-32-215	BSI	NEG	NEG	NEG	NEG	NEG	POS	POS	POS	POS	NEG	NEG	AMB	NEG	POS	POS	POS	POS	NEG	POS	POS	POS	POS	POS	POS	POS	AMB	POS	NEG	POS	NEG	NEG	NEG	NEG	NEG	NEG	NEG	NEG	NEG	NEG	POS	POS	NEG	POS	POS	NEG
Iasi-43-359	BSI	NEG	NEG	NEG	NEG	NEG	POS	POS	POS	POS	NEG	NEG	NEG	NEG	POS	POS	POS	POS	NEG	POS	POS	POS	POS	POS	POS	POS	NEG	POS	NEG	POS	NEG	NEG	NEG	NEG	NEG	NEG	NEG	NEG	NEG	NEG	POS	POS	NEG	POS	POS	POS
Iasi-03-27	SSTI	NEG	NEG	NEG	NEG	NEG	POS	POS	POS	POS	NEG	NEG	NEG	NEG	POS	POS	POS	POS	NEG	POS	POS	POS	POS	POS	POS	POS	NEG	POS	NEG	POS	NEG	NEG	NEG	NEG	NEG	NEG	NEG	NEG	NEG	NEG	POS	POS	NEG	POS	POS	NEG
Iasi-13-149	SSTI	NEG	NEG	NEG	NEG	NEG	POS	POS	POS	POS	NEG	NEG	NEG	NEG	POS	POS	POS	POS	NEG	POS	POS	POS	POS	POS	POS	POS	NEG	POS	NEG	POS	NEG	NEG	NEG	NEG	NEG	NEG	NEG	NEG	NEG	NEG	POS	POS	NEG	POS	POS	NEG
Iasi-29-337	SSTI	NEG	NEG	NEG	NEG	NEG	POS	POS	NEG	POS	NEG	NEG	NEG	NEG	POS	POS	AMB	POS	NEG	POS	POS	POS	POS	POS	POS	POS	NEG	POS	NEG	POS	NEG	NEG	NEG	NEG	NEG	NEG	NEG	NEG	NEG	NEG	POS	POS	NEG	POS	POS	NEG
Iasi-31-355	SSTI	NEG	NEG	NEG	NEG	NEG	POS	POS	POS	POS	NEG	NEG	NEG	NEG	POS	POS	POS	POS	NEG	POS	POS	POS	POS	POS	POS	POS	POS	NEG	NEG	NEG	NEG	NEG	NEG	NEG	NEG	NEG	NEG	NEG	NEG	NEG	POS	POS	NEG	POS	POS	NEG
Iasi-38-377	SSTI	NEG	NEG	NEG	NEG	NEG	POS	POS	POS	POS	NEG	NEG	NEG	NEG	POS	POS	POS	POS	NEG	POS	POS	POS	POS	POS	POS	POS	POS	NEG	NEG	NEG	NEG	NEG	NEG	NEG	NEG	NEG	NEG	NEG	NEG	NEG	POS	POS	NEG	POS	POS	NEG
Iasi-41-388	SSTI	NEG	NEG	NEG	NEG	NEG	POS	POS	POS	POS	NEG	NEG	NEG	NEG	POS	POS	POS	POS	NEG	POS	POS	POS	POS	POS	POS	POS	NEG	POS	NEG	POS	NEG	NEG	NEG	NEG	NEG	NEG	NEG	NEG	NEG	NEG	POS	POS	NEG	POS	POS	NEG
Iasi-46-417	SSTI	NEG	NEG	NEG	NEG	NEG	POS	POS	POS	POS	NEG	NEG	NEG	NEG	POS	POS	POS	POS	NEG	POS	POS	POS	POS	POS	POS	POS	NEG	POS	NEG	POS	NEG	NEG	NEG	NEG	NEG	NEG	NEG	NEG	NEG	NEG	POS	POS	NEG	POS	POS	NEG
Iasi-02-S9	Carrier	NEG	NEG	NEG	NEG	NEG	POS	POS	POS	POS	NEG	NEG	NEG	NEG	POS	POS	POS	POS	NEG	POS	POS	POS	POS	POS	POS	POS	POS	NEG	NEG	NEG	NEG	NEG	NEG	NEG	NEG	NEG	NEG	NEG	NEG	NEG	POS	POS	NEG	POS	POS	NEG
Iasi-17-S33	Carrier	NEG	NEG	NEG	NEG	NEG	POS	POS	POS	POS	NEG	NEG	NEG	NEG	POS	POS	POS	POS	NEG	POS	POS	POS	POS	POS	POS	POS	NEG	POS	NEG	POS	NEG	NEG	NEG	NEG	NEG	NEG	NEG	NEG	NEG	NEG	POS	POS	NEG	POS	POS	NEG
Iasi-30-P17	Carrier	NEG	NEG	NEG	NEG	NEG	POS	POS	POS	POS	NEG	NEG	NEG	NEG	POS	POS	POS	POS	NEG	POS	POS	POS	POS	POS	POS	POS	POS	NEG	NEG	NEG	NEG	NEG	NEG	NEG	NEG	NEG	NEG	NEG	NEG	NEG	POS	POS	NEG	POS	POS	NEG
Iasi-39-P28	Carrier	NEG	NEG	NEG	NEG	NEG	POS	POS	POS	POS	NEG	NEG	NEG	NEG	POS	POS	POS	POS	NEG	POS	POS	POS	POS	POS	POS	POS	AMB	POS	NEG	POS	NEG	NEG	NEG	NEG	NEG	NEG	NEG	NEG	NEG	NEG	POS	POS	NEG	POS	POS	NEG
Iasi-04-26	BSI	NEG	NEG	NEG	NEG	NEG	POS	POS	POS	POS	NEG	NEG	NEG	NEG	POS	POS	POS	POS	NEG	POS	POS	POS	POS	POS	POS	POS	NEG	POS	NEG	POS	NEG	NEG	NEG	NEG	NEG	NEG	NEG	NEG	NEG	NEG	POS	POS	NEG	POS	POS	NEG
Iasi-09-72	BSI	NEG	NEG	NEG	NEG	NEG	POS	POS	AMB	POS	NEG	NEG	NEG	NEG	POS	POS	POS	POS	NEG	POS	POS	POS	POS	POS	POS	POS	NEG	POS	NEG	POS	NEG	NEG	NEG	NEG	NEG	NEG	NEG	NEG	NEG	NEG	POS	POS	NEG	POS	POS	NEG
Iasi-11-131	BSI	NEG	NEG	NEG	NEG	NEG	POS	POS	AMB	POS	NEG	NEG	NEG	NEG	POS	POS	POS	POS	NEG	POS	POS	POS	POS	POS	POS	POS	NEG	POS	NEG	POS	NEG	NEG	NEG	NEG	NEG	NEG	NEG	NEG	NEG	NEG	POS	POS	NEG	POS	POS	NEG
Iasi-14-143	BSI	NEG	NEG	NEG	NEG	NEG	POS	POS	AMB	POS	NEG	NEG	NEG	NEG	POS	POS	POS	POS	NEG	POS	POS	POS	POS	POS	POS	POS	NEG	POS	NEG	POS	NEG	NEG	NEG	NEG	NEG	NEG	NEG	NEG	NEG	NEG	POS	POS	NEG	POS	POS	NEG
Iasi-18-192	BSI	NEG	NEG	NEG	NEG	NEG	POS	POS	AMB	POS	NEG	NEG	NEG	NEG	POS	POS	POS	POS	NEG	POS	POS	POS	POS	POS	POS	POS	NEG	POS	NEG	POS	NEG	NEG	NEG	NEG	NEG	NEG	NEG	NEG	NEG	NEG	POS	POS	NEG	POS	POS	NEG
Iasi-23-253	BSI	NEG	NEG	NEG	NEG	NEG	POS	POS	POS	POS	NEG	NEG	NEG	NEG	POS	POS	POS	POS	NEG	POS	POS	POS	POS	POS	POS	POS	POS	POS	NEG	POS	NEG	NEG	NEG	NEG	NEG	NEG	NEG	NEG	NEG	NEG	POS	POS	NEG	POS	POS	NEG
Iasi-35-322	BSI	NEG	NEG	NEG	NEG	NEG	POS	POS	POS	POS	NEG	NEG	NEG	NEG	POS	POS	POS	POS	NEG	POS	POS	POS	POS	POS	POS	POS	NEG	POS	NEG	POS	NEG	NEG	NEG	NEG	NEG	NEG	NEG	NEG	NEG	NEG	POS	POS	NEG	POS	POS	NEG
Iasi-40-380	BSI	NEG	NEG	NEG	NEG	NEG	POS	POS	POS	POS	NEG	NEG	NEG	NEG	POS	POS	POS	POS	NEG	POS	POS	POS	POS	POS	POS	POS	NEG	POS	NEG	POS	NEG	NEG	NEG	NEG	NEG	NEG	NEG	NEG	NEG	NEG	POS	POS	NEG	POS	POS	NEG
Iasi-46-406	BSI	NEG	NEG	NEG	NEG	NEG	POS	POS	AMB	POS	NEG	NEG	NEG	NEG	POS	POS	POS	POS	NEG	POS	POS	POS	POS	POS	POS	POS	NEG	POS	NEG	POS	NEG	NEG	NEG	NEG	NEG	NEG	NEG	NEG	NEG	NEG	POS	POS	NEG	POS	POS	NEG
Iasi-02-6	SSTI	NEG	NEG	NEG	NEG	NEG	POS	POS	AMB	POS	NEG	NEG	NEG	NEG	POS	POS	POS	POS	NEG	POS	POS	POS	POS	POS	POS	POS	NEG	POS	NEG	POS	NEG	NEG	NEG	NEG	NEG	NEG	NEG	NEG	NEG	NEG	POS	POS	NEG	POS	POS	NEG
Iasi-09-97	SSTI	NEG	NEG	NEG	NEG	NEG	POS	POS	AMB	POS	NEG	NEG	NEG	NEG	POS	POS	POS	POS	NEG	POS	POS	POS	POS	POS	POS	POS	NEG	POS	NEG	POS	NEG	NEG	NEG	NEG	NEG	NEG	NEG	NEG	NEG	NEG	POS	POS	NEG	POS	POS	NEG
Iasi-19-182	SSTI	NEG	NEG	NEG	NEG	NEG	POS	POS	AMB	POS	NEG	NEG	NEG	NEG	POS	POS	POS	POS	NEG	POS	POS	POS	POS	POS	POS	POS	NEG	POS	NEG	POS	NEG	NEG	NEG	NEG	NEG	NEG	NEG	NEG	NEG	NEG	POS	POS	NEG	POS	POS	NEG
Iasi-31-314	SSTI	NEG	NEG	NEG	NEG	NEG	POS	POS	AMB	POS	NEG	NEG	NEG	NEG	POS	POS	POS	POS	NEG	POS	POS	POS	POS	POS	POS	POS	NEG	POS	NEG	POS	NEG	NEG	NEG	NEG	NEG	NEG	NEG	NEG	NEG	NEG	POS	POS	NEG	POS	POS	NEG
Iasi-33-367	SSTI	NEG	NEG	NEG	NEG	NEG	POS	POS	AMB	POS	NEG	NEG	NEG	NEG	POS	POS	POS	POS	NEG	POS	POS	POS	POS	POS	POS	POS	POS	NEG	NEG	NEG	NEG	NEG	NEG	NEG	NEG	NEG	NEG	NEG	NEG	NEG	POS	POS	NEG	POS	POS	NEG
Iasi-39-378	SSTI	NEG	NEG	NEG	NEG	NEG	POS	POS	POS	POS	NEG	NEG	NEG	NEG	POS	POS	POS	POS	NEG	POS	POS	POS	POS	POS	POS	POS	NEG	POS	NEG	POS	NEG	NEG	NEG	NEG	NEG	NEG	NEG	NEG	NEG	NEG	POS	POS	NEG	POS	POS	NEG
Iasi-42-394	SSTI	NEG	NEG	NEG	NEG	NEG	POS	POS	POS	POS	NEG	NEG	NEG	NEG	POS	POS	POS	POS	NEG	POS	POS	POS	POS	POS	POS	POS	POS	NEG	NEG	NEG	NEG	NEG	NEG	NEG	NEG	NEG	NEG	NEG	NEG	NEG	POS	POS	NEG	POS	POS	NEG
Iasi-44-409	SSTI	NEG	NEG	NEG	NEG	NEG	POS	POS	POS	POS	NEG	NEG	NEG	NEG	POS	POS	POS	POS	NEG	POS	POS	POS	POS	POS	POS	POS	NEG	POS	NEG	POS	NEG	NEG	NEG	NEG	NEG	NEG	NEG	NEG	NEG	NEG	POS	POS	NEG	POS	POS	NEG
Iasi-45-405	SSTI	NEG	NEG	NEG	NEG	NEG	POS	POS	POS	POS	NEG	NEG	NEG	NEG	POS	POS	POS	POS	NEG	POS	POS	POS	POS	POS	POS	POS	NEG	POS	NEG	POS	NEG	NEG	NEG	NEG	NEG	NEG	NEG	NEG	NEG	NEG	POS	POS	NEG	POS	POS	NEG
Iasi-50-425	SSTI	NEG	NEG	NEG	NEG	NEG	POS	POS	POS	POS	NEG	NEG	NEG	NEG	POS	POS	POS	POS	NEG	POS	POS	POS	POS	POS	POS	POS	NEG	POS	NEG	POS	NEG	NEG	NEG	NEG	NEG	NEG	NEG	NEG	NEG	NEG	POS	POS	NEG	POS	POS	NEG
Iasi-50-428	SSTI	NEG	NEG	NEG	NEG	NEG	POS	POS	POS	POS	NEG	NEG	NEG	NEG	POS	POS	POS	POS	NEG	POS	POS	POS	POS	POS	POS	POS	POS	NEG	NEG	NEG	NEG	NEG	NEG	NEG	NEG	NEG	NEG	NEG	NEG	NEG	POS	POS	NEG	POS	POS	NEG
Iasi-51-37	SSTI	NEG	NEG	NEG	NEG	NEG	POS	POS	POS	POS	NEG	NEG	NEG	NEG	POS	POS	POS	POS	NEG	POS	POS	POS	POS	POS	POS	POS	NEG	POS	NEG	POS	NEG	NEG	NEG	NEG	NEG	NEG	NEG	NEG	NEG	NEG	POS	POS	NEG	POS	POS	NEG
Iasi-01-S8	Carrier	NEG	NEG	NEG	NEG	NEG	POS	POS	POS	POS	NEG	NEG	NEG	NEG	POS	POS	POS	POS	NEG	POS	POS	POS	POS	POS	POS	POS	AMB	POS	NEG	POS	NEG	NEG	NEG	NEG	NEG	NEG	NEG	NEG	NEG	NEG	POS	POS	NEG	POS	POS	NEG
Iasi-08-S17	Carrier	NEG	NEG	NEG	NEG	NEG	POS	POS	POS	POS	NEG	NEG	NEG	NEG	POS	POS	POS	POS	NEG	POS	POS	POS	POS	POS	POS	POS	NEG	POS	NEG	POS	NEG	NEG	NEG	NEG	NEG	NEG	NEG	NEG	NEG	NEG	POS	POS	NEG	POS	POS	NEG
Iasi-10-S23	Carrier	NEG	NEG	NEG	NEG	NEG	POS	POS	POS	POS	NEG	NEG	NEG	NEG	POS	POS	POS	POS	NEG	POS	POS	POS	POS	POS	POS	POS	POS	NEG	NEG	NEG	NEG	NEG	NEG	NEG	NEG	NEG	NEG	NEG	NEG	NEG	POS	POS	NEG	POS	POS	NEG
Iasi-13-S27	Carrier	NEG	NEG	NEG	NEG	NEG	POS	POS	POS	POS	NEG	NEG	NEG	NEG	POS	POS	POS	POS	NEG	POS	POS	POS	POS	POS	POS	POS	AMB	POS	NEG	POS	NEG	NEG	NEG	NEG	NEG	NEG	NEG	NEG	NEG	NEG	POS	POS	NEG	POS	POS	NEG
Iasi-14-S28	Carrier	NEG	NEG	NEG	NEG	NEG	POS	POS	POS	POS	NEG	NEG	NEG	NEG	POS	POS	POS	POS	NEG	POS	POS	POS	POS	POS	POS	POS	POS	NEG	NEG	NEG	NEG	NEG	NEG	NEG	NEG	NEG	NEG	NEG	NEG	NEG	POS	POS	NEG	POS	POS	NEG
Iasi-15-S29	Carrier	NEG	NEG	NEG	NEG	NEG	POS	POS	POS	POS	NEG	NEG	NEG	NEG	POS	POS	POS	POS	NEG	POS	POS	POS	POS	POS	POS	POS	POS	POS	NEG	POS	NEG	NEG	NEG	NEG	NEG	NEG	NEG	NEG	NEG	NEG	POS	POS	NEG	POS	POS	NEG
Iasi-25-P10	Carrier	NEG	NEG	NEG	NEG	NEG	POS	POS	POS	POS	NEG	NEG	AMB	NEG	POS	POS	POS	POS	NEG	POS	POS	POS	POS	POS	POS	POS	NEG	POS	NEG	POS	NEG	NEG	NEG	NEG	NEG	NEG	NEG	NEG	NEG	NEG	POS	POS	NEG	POS	POS	NEG
Iasi-26-P11	Carrier	NEG	NEG	NEG	NEG	NEG	POS	POS	POS	POS	NEG	NEG	NEG	NEG	POS	POS	POS	POS	NEG	POS	POS	POS	POS	POS	POS	POS	NEG	POS	NEG	POS	NEG	NEG	NEG	NEG	NEG	NEG	NEG	NEG	NEG	NEG	POS	POS	NEG	POS	POS	NEG
Iasi-32-P19	Carrier	NEG	NEG	NEG	NEG	NEG	POS	POS	POS	POS	NEG	NEG	NEG	NEG	POS	POS	POS	POS	NEG	POS	POS	POS	POS	POS	POS	POS	AMB	POS	NEG	POS	NEG	NEG	NEG	NEG	NEG	NEG	NEG	NEG	NEG	NEG	POS	POS	NEG	POS	POS	NEG
Iasi-33-P22	Carrier	NEG	NEG	NEG	NEG	NEG	POS	POS	POS	POS	NEG	NEG	NEG	NEG	POS	POS	POS	POS	NEG	POS	POS	POS	POS	POS	POS	POS	POS	NEG	NEG	NEG	NEG	NEG	NEG	NEG	NEG	NEG	NEG	NEG	NEG	NEG	POS	POS	NEG	POS	POS	NEG
Iasi-41-P44	Carrier	NEG	NEG	NEG	NEG	NEG	POS	POS	POS	POS	NEG	NEG	NEG	NEG	POS	POS	POS	POS	NEG	POS	POS	POS	POS	POS	POS	POS	NEG	POS	NEG	POS	NEG	NEG	NEG	NEG	NEG	NEG	NEG	NEG	NEG	NEG	POS	POS	NEG	POS	POS	NEG
Iasi-45-G515	Carrier	NEG	NEG	NEG	NEG	NEG	POS	POS	POS	POS	NEG	NEG	NEG	NEG	POS	POS	POS	POS	NEG	POS	POS	POS	POS	POS	POS	POS	NEG	POS	NEG	POS	NEG	NEG	NEG	NEG	NEG	NEG	NEG	NEG	NEG	NEG	POS	POS	NEG	POS	POS	NEG
Iasi-02-39	misc. MRSA	NEG	NEG	NEG	NEG	NEG	POS	POS	AMB	POS	NEG	NEG	NEG	NEG	POS	POS	POS	POS	NEG	POS	POS	POS	POS	POS	POS	POS	NEG	POS	NEG	POS	NEG	NEG	NEG	NEG	NEG	NEG	NEG	NEG	NEG	NEG	POS	POS	NEG	POS	POS	NEG
Iasi-24-263	misc. MRSA	NEG	NEG	NEG	NEG	NEG	POS	POS	POS	POS	NEG	NEG	NEG	NEG	POS	POS	POS	POS	NEG	POS	POS	POS	POS	POS	POS	POS	POS	NEG	NEG	NEG	NEG	NEG	NEG	NEG	NEG	NEG	NEG	NEG	NEG	NEG	POS	POS	NEG	POS	POS	NEG
Iasi-26-285	misc. MRSA	NEG	NEG	NEG	NEG	NEG	POS	POS	POS	POS	NEG	NEG	NEG	NEG	POS	POS	POS	POS	NEG	POS	POS	POS	POS	POS	POS	POS	NEG	POS	NEG	POS	NEG	NEG	NEG	NEG	NEG	NEG	NEG	NEG	NEG	NEG	POS	POS	NEG	POS	POS	NEG
Iasi-30-306	misc. MRSA	NEG	NEG	NEG	NEG	NEG	POS	POS	POS	POS	NEG	NEG	NEG	NEG	POS	POS	POS	POS	NEG	POS	POS	POS	POS	POS	POS	POS	POS	POS	NEG	POS	NEG	NEG	NEG	NEG	NEG	NEG	NEG	NEG	NEG	NEG	POS	POS	NEG	POS	POS	NEG
Iasi-41-390	misc. MRSA	NEG	NEG	NEG	NEG	NEG	POS	POS	POS	POS	NEG	NEG	NEG	NEG	POS	POS	POS	POS	NEG	POS	POS	POS	POS	POS	POS	POS	NEG	POS	NEG	POS	NEG	NEG	NEG	NEG	NEG	NEG	NEG	NEG	NEG	NEG	POS	POS	NEG	POS	POS	NEG
Iasi-47-407	misc. MRSA	NEG	NEG	NEG	NEG	NEG	POS	POS	POS	POS	NEG	NEG	AMB	NEG	POS	POS	POS	POS	NEG	POS	POS	POS	POS	POS	POS	POS	NEG	POS	NEG	POS	NEG	NEG	NEG	NEG	NEG	NEG	NEG	NEG	NEG	NEG	POS	POS	NEG	POS	POS	NEG
Iasi-55-408CP	misc. MRSA	NEG	NEG	NEG	NEG	NEG	POS	POS	POS	POS	NEG	NEG	NEG	NEG	POS	POS	POS	POS	NEG	POS	POS	POS	POS	POS	POS	POS	NEG	POS	NEG	POS	NEG	NEG	NEG	NEG	NEG	NEG	NEG	NEG	NEG	NEG	POS	POS	NEG	POS	POS	NEG
Iasi-15-151	BSI	POS	NEG	NEG	POS	NEG	POS	POS	POS	POS	NEG	NEG	NEG	NEG	POS	POS	POS	POS	NEG	POS	POS	POS	POS	POS	POS	POS	AMB	POS	POS	POS	NEG	NEG	NEG	NEG	NEG	NEG	NEG	NEG	NEG	NEG	POS	POS	NEG	POS	POS	NEG
Iasi-46-386	BSI	POS	NEG	NEG	POS	NEG	POS	POS	POS	POS	NEG	NEG	NEG	NEG	POS	POS	POS	POS	NEG	POS	POS	POS	POS	POS	POS	POS	POS	POS	POS	POS	NEG	NEG	NEG	NEG	NEG	NEG	NEG	NEG	NEG	NEG	POS	POS	NEG	POS	POS	NEG
Iasi-21-195	SSTI	POS	NEG	NEG	POS	NEG	POS	POS	AMB	POS	NEG	NEG	NEG	NEG	POS	POS	POS	POS	NEG	POS	POS	POS	POS	POS	POS	AMB	NEG	POS	POS	POS	NEG	NEG	NEG	POS	NEG	NEG	NEG	NEG	NEG	NEG	POS	POS	NEG	POS	POS	NEG
Iasi-37-375	SSTI	POS	NEG	NEG	POS	NEG	POS	POS	POS	POS	NEG	NEG	NEG	NEG	POS	POS	POS	POS	NEG	POS	POS	POS	POS	POS	POS	POS	NEG	POS	NEG	POS	NEG	NEG	NEG	NEG	NEG	NEG	NEG	NEG	NEG	NEG	POS	POS	NEG	POS	POS	NEG
Iasi-45-415	SSTI	POS	NEG	NEG	POS	NEG	POS	POS	POS	POS	NEG	NEG	NEG	NEG	POS	POS	POS	POS	NEG	POS	POS	POS	POS	POS	POS	POS	NEG	POS	POS	POS	NEG	NEG	NEG	NEG	NEG	NEG	NEG	NEG	NEG	NEG	POS	POS	NEG	POS	POS	NEG
Iasi-09-S19	Carrier	POS	NEG	NEG	POS	NEG	POS	POS	POS	POS	NEG	NEG	NEG	NEG	POS	POS	POS	POS	NEG	POS	POS	POS	POS	POS	POS	POS	POS	NEG	AMB	NEG	NEG	NEG	NEG	NEG	NEG	NEG	NEG	NEG	NEG	NEG	POS	POS	NEG	POS	POS	NEG
Iasi-53-434	BSI	POS	NEG	NEG	POS	NEG	POS	POS	POS	POS	POS	POS	NEG	NEG	POS	POS	POS	POS	NEG	POS	POS	POS	POS	POS	POS	POS	NEG	POS	POS	POS	NEG	NEG	NEG	NEG	NEG	NEG	NEG	NEG	NEG	NEG	POS	POS	NEG	POS	POS	NEG
Iasi-03-40	misc. MRSA	POS	NEG	POS	POS	NEG	POS	POS	POS	POS	NEG	NEG	NEG	NEG	POS	POS	POS	POS	NEG	POS	POS	POS	POS	POS	POS	POS	AMB	POS	POS	POS	NEG	NEG	NEG	NEG	NEG	NEG	NEG	NEG	NEG	NEG	POS	POS	NEG	POS	POS	NEG
Iasi-04-43	misc. MRSA	POS	NEG	POS	POS	NEG	POS	POS	AMB	POS	NEG	NEG	NEG	NEG	POS	POS	POS	POS	NEG	POS	POS	POS	POS	POS	POS	POS	NEG	POS	POS	POS	NEG	NEG	NEG	NEG	NEG	NEG	NEG	NEG	NEG	NEG	POS	POS	NEG	POS	POS	NEG
Iasi-01-20	misc. MRSA	POS	NEG	POS	POS	NEG	POS	POS	POS	POS	NEG	NEG	NEG	NEG	POS	POS	POS	POS	NEG	POS	POS	POS	POS	POS	POS	AMB	POS	NEG	NEG	NEG	NEG	NEG	NEG	NEG	NEG	NEG	NEG	NEG	NEG	NEG	POS	POS	NEG	POS	POS	NEG
Iasi-35-228	BSI	NEG	NEG	NEG	NEG	NEG	POS	POS	POS	POS	NEG	NEG	NEG	NEG	POS	POS	POS	POS	NEG	POS	POS	POS	POS	POS	POS	POS	NEG	POS	NEG	POS	NEG	NEG	NEG	NEG	NEG	NEG	NEG	NEG	NEG	NEG	POS	POS	NEG	POS	POS	POS
Iasi-38-293	BSI	NEG	NEG	NEG	NEG	NEG	POS	POS	POS	POS	NEG	NEG	AMB	NEG	POS	POS	POS	POS	NEG	POS	POS	POS	POS	POS	POS	POS	POS	POS	NEG	POS	NEG	NEG	NEG	NEG	NEG	NEG	NEG	NEG	NEG	NEG	POS	POS	NEG	POS	POS	POS
Iasi-31-P18	Carrier	NEG	NEG	NEG	NEG	NEG	POS	POS	POS	POS	NEG	NEG	NEG	NEG	POS	POS	POS	POS	NEG	POS	POS	POS	POS	POS	POS	POS	AMB	POS	NEG	POS	NEG	NEG	NEG	NEG	NEG	NEG	NEG	NEG	NEG	NEG	POS	POS	NEG	POS	POS	POS
Iasi-40-P42	Carrier	NEG	NEG	NEG	NEG	NEG	POS	POS	POS	POS	NEG	NEG	AMB	NEG	POS	POS	POS	POS	NEG	POS	POS	POS	POS	POS	POS	POS	POS	POS	NEG	POS	NEG	NEG	NEG	NEG	NEG	NEG	NEG	NEG	NEG	NEG	POS	POS	NEG	POS	POS	POS
Iasi-40-303	BSI	NEG	NEG	NEG	NEG	NEG	POS	POS	POS	POS	NEG	NEG	NEG	NEG	POS	POS	POS	POS	NEG	POS	POS	POS	POS	POS	POS	POS	AMB	POS	NEG	POS	NEG	NEG	NEG	NEG	NEG	NEG	NEG	NEG	NEG	NEG	POS	POS	NEG	POS	POS	POS
Iasi-48-422	SSTI	NEG	NEG	NEG	NEG	NEG	POS	POS	AMB	POS	NEG	NEG	NEG	NEG	POS	POS	POS	POS	NEG	POS	POS	POS	POS	POS	POS	POS	NEG	POS	NEG	POS	NEG	NEG	NEG	NEG	NEG	NEG	NEG	NEG	NEG	NEG	POS	POS	NEG	POS	POS	POS
Iasi-54-435	BSI	NEG	NEG	NEG	NEG	NEG	POS	POS	POS	POS	POS	POS	AMB	NEG	POS	POS	POS	POS	NEG	POS	POS	POS	POS	POS	POS	POS	POS	POS	POS	POS	NEG	NEG	NEG	NEG	NEG	NEG	NEG	NEG	NEG	NEG	POS	POS	NEG	POS	POS	POS
Iasi-16-161	SSTI	NEG	POS	NEG	NEG	NEG	POS	POS	POS	POS	POS	POS	NEG	NEG	POS	POS	POS	POS	NEG	POS	POS	POS	POS	POS	POS	POS	NEG	POS	POS	POS	NEG	NEG	NEG	NEG	NEG	NEG	NEG	NEG	NEG	NEG	POS	POS	NEG	POS	POS	POS
Iasi-27-221	SSTI	NEG	POS	NEG	NEG	NEG	POS	POS	AMB	POS	POS	POS	NEG	NEG	POS	POS	POS	POS	NEG	POS	POS	POS	POS	POS	POS	POS	NEG	POS	POS	POS	NEG	NEG	NEG	NEG	NEG	NEG	NEG	NEG	NEG	NEG	POS	POS	NEG	POS	POS	POS
Iasi-09-85	BSI	NEG	AMB	NEG	NEG	NEG	POS	POS	NEG	POS	POS	POS	NEG	NEG	POS	POS	POS	POS	NEG	POS	POS	POS	POS	POS	POS	POS	NEG	POS	POS	POS	NEG	NEG	NEG	NEG	NEG	NEG	POS	POS	POS	POS	POS	POS	NEG	POS	POS	POS
Iasi-17-189	BSI	NEG	POS	NEG	NEG	NEG	POS	POS	AMB	POS	POS	POS	NEG	NEG	POS	POS	POS	POS	NEG	POS	POS	POS	POS	POS	POS	POS	NEG	POS	POS	POS	NEG	NEG	NEG	NEG	NEG	NEG	POS	POS	POS	POS	POS	POS	NEG	POS	POS	POS
Iasi-20-200	BSI	NEG	POS	NEG	NEG	NEG	POS	POS	AMB	POS	POS	POS	NEG	NEG	POS	POS	POS	POS	NEG	POS	POS	POS	POS	POS	POS	POS	NEG	POS	POS	POS	NEG	NEG	NEG	NEG	NEG	NEG	POS	POS	POS	POS	POS	POS	NEG	POS	POS	POS
Iasi-34-321	BSI	NEG	POS	NEG	NEG	NEG	POS	POS	AMB	POS	POS	POS	NEG	NEG	POS	POS	POS	POS	NEG	POS	POS	POS	POS	POS	POS	POS	NEG	POS	POS	POS	NEG	NEG	NEG	NEG	NEG	NEG	POS	POS	POS	POS	POS	POS	NEG	POS	POS	POS
Iasi-04-57	SSTI	NEG	POS	NEG	NEG	NEG	POS	POS	AMB	POS	POS	POS	NEG	NEG	POS	POS	POS	POS	NEG	POS	POS	POS	POS	POS	POS	POS	NEG	POS	POS	POS	NEG	NEG	NEG	NEG	NEG	NEG	POS	POS	POS	POS	POS	POS	NEG	POS	POS	POS
Iasi-05-59	SSTI	NEG	POS	NEG	NEG	NEG	POS	POS	AMB	POS	POS	POS	NEG	NEG	POS	POS	POS	POS	NEG	POS	POS	POS	POS	POS	POS	POS	NEG	POS	POS	POS	NEG	NEG	NEG	NEG	NEG	NEG	POS	POS	POS	POS	POS	POS	NEG	POS	POS	POS
Iasi-06-64	SSTI	NEG	POS	NEG	NEG	NEG	POS	POS	AMB	POS	POS	POS	NEG	NEG	POS	POS	POS	POS	NEG	POS	POS	POS	POS	POS	POS	POS	AMB	POS	POS	POS	NEG	NEG	NEG	NEG	NEG	NEG	POS	POS	POS	POS	POS	POS	NEG	POS	POS	POS
Iasi-10-108	SSTI	NEG	AMB	NEG	NEG	NEG	POS	POS	AMB	POS	POS	POS	NEG	NEG	POS	POS	POS	POS	NEG	POS	POS	POS	POS	POS	POS	POS	NEG	POS	POS	POS	NEG	NEG	NEG	NEG	NEG	NEG	POS	POS	POS	POS	POS	POS	NEG	POS	POS	POS
Iasi-12-117	SSTI	NEG	POS	NEG	NEG	NEG	POS	POS	POS	POS	POS	POS	NEG	NEG	POS	POS	POS	POS	NEG	POS	POS	POS	POS	POS	POS	POS	AMB	POS	POS	POS	NEG	NEG	NEG	NEG	NEG	NEG	POS	POS	POS	POS	POS	POS	NEG	POS	POS	POS
Iasi-12-138	SSTI	NEG	POS	NEG	NEG	NEG	POS	POS	AMB	POS	POS	POS	NEG	NEG	POS	POS	POS	POS	NEG	POS	POS	POS	POS	POS	POS	POS	AMB	POS	POS	POS	NEG	NEG	NEG	NEG	NEG	NEG	POS	POS	POS	POS	POS	POS	NEG	POS	POS	POS
Iasi-13-140	SSTI	NEG	AMB	NEG	NEG	NEG	POS	POS	NEG	POS	POS	POS	NEG	NEG	POS	POS	POS	POS	NEG	POS	POS	POS	POS	POS	POS	POS	NEG	POS	POS	POS	NEG	NEG	NEG	NEG	NEG	NEG	POS	POS	POS	POS	POS	POS	NEG	POS	POS	POS
Iasi-15-152	SSTI	NEG	POS	NEG	NEG	NEG	POS	POS	AMB	POS	POS	POS	NEG	NEG	POS	POS	POS	POS	NEG	POS	POS	POS	POS	POS	POS	POS	NEG	POS	POS	POS	NEG	NEG	NEG	NEG	NEG	NEG	POS	POS	POS	POS	POS	POS	NEG	POS	POS	POS
Iasi-17-168	SSTI	NEG	POS	NEG	NEG	NEG	POS	POS	POS	POS	POS	POS	NEG	NEG	POS	POS	POS	POS	NEG	POS	POS	POS	POS	POS	POS	POS	NEG	POS	POS	POS	NEG	NEG	NEG	NEG	NEG	NEG	POS	POS	POS	POS	POS	POS	NEG	POS	POS	POS
Iasi-20-193	SSTI	NEG	POS	NEG	NEG	NEG	POS	POS	POS	POS	POS	POS	NEG	NEG	POS	POS	POS	POS	NEG	POS	POS	POS	POS	POS	POS	POS	NEG	POS	POS	POS	NEG	NEG	NEG	NEG	NEG	NEG	POS	POS	POS	POS	POS	POS	NEG	POS	POS	POS
Iasi-26-218	SSTI	NEG	POS	NEG	NEG	NEG	POS	POS	AMB	POS	POS	POS	NEG	NEG	POS	POS	POS	POS	NEG	POS	POS	POS	POS	POS	POS	POS	NEG	POS	POS	POS	NEG	NEG	NEG	NEG	NEG	NEG	POS	POS	POS	POS	POS	POS	NEG	POS	POS	POS
Iasi-28-335	SSTI	NEG	POS	NEG	NEG	NEG	POS	POS	NEG	POS	POS	POS	NEG	NEG	POS	POS	POS	POS	NEG	POS	POS	POS	POS	POS	POS	POS	NEG	POS	POS	POS	NEG	NEG	NEG	NEG	NEG	NEG	POS	POS	POS	POS	POS	POS	NEG	POS	POS	POS
Iasi-29-299b	SSTI	NEG	POS	NEG	NEG	NEG	POS	POS	POS	POS	POS	POS	NEG	NEG	POS	POS	POS	POS	NEG	POS	POS	POS	POS	POS	POS	POS	AMB	POS	POS	POS	NEG	NEG	NEG	NEG	NEG	NEG	POS	POS	POS	POS	POS	POS	NEG	POS	POS	POS
Iasi-32-364	SSTI	NEG	POS	NEG	NEG	NEG	POS	POS	AMB	POS	POS	POS	NEG	NEG	POS	POS	POS	POS	NEG	POS	POS	POS	POS	POS	POS	POS	NEG	POS	POS	POS	NEG	NEG	NEG	NEG	NEG	NEG	POS	POS	POS	POS	POS	POS	NEG	POS	POS	POS
Iasi-369	SSTI	NEG	POS	NEG	NEG	NEG	POS	POS	AMB	POS	POS	POS	NEG	NEG	POS	POS	POS	POS	NEG	POS	POS	POS	POS	POS	POS	POS	NEG	POS	POS	POS	NEG	NEG	NEG	NEG	NEG	NEG	POS	POS	POS	POS	POS	POS	NEG	POS	POS	POS
Iasi-03-S11	Carrier	NEG	POS	NEG	NEG	NEG	POS	POS	POS	POS	POS	POS	NEG	NEG	POS	POS	POS	POS	NEG	POS	POS	POS	POS	POS	POS	POS	POS	NEG	NEG	NEG	NEG	NEG	NEG	NEG	NEG	NEG	POS	POS	POS	POS	POS	POS	NEG	POS	POS	POS
Iasi-19-S35	Carrier	NEG	POS	NEG	NEG	NEG	POS	POS	POS	POS	POS	POS	NEG	NEG	POS	POS	POS	POS	NEG	POS	POS	POS	POS	POS	POS	POS	POS	POS	POS	POS	NEG	NEG	NEG	NEG	NEG	NEG	POS	POS	POS	POS	POS	POS	NEG	POS	POS	POS
Iasi-20-P1	Carrier	NEG	POS	NEG	NEG	NEG	POS	POS	POS	POS	POS	POS	NEG	NEG	POS	POS	POS	POS	NEG	POS	POS	POS	POS	POS	POS	POS	AMB	POS	POS	POS	NEG	NEG	NEG	NEG	NEG	NEG	POS	POS	POS	POS	POS	POS	NEG	POS	POS	POS
Iasi-35-P24	Carrier	NEG	POS	NEG	NEG	NEG	POS	POS	POS	POS	POS	POS	NEG	NEG	POS	POS	POS	POS	NEG	POS	POS	POS	POS	POS	POS	POS	AMB	POS	POS	POS	NEG	NEG	NEG	NEG	NEG	NEG	POS	POS	POS	POS	POS	POS	NEG	POS	POS	POS
Iasi-36-P25	Carrier	NEG	POS	NEG	NEG	NEG	POS	POS	POS	POS	POS	POS	NEG	NEG	POS	POS	POS	POS	NEG	POS	POS	POS	POS	POS	POS	POS	AMB	POS	POS	POS	NEG	NEG	NEG	NEG	NEG	NEG	POS	POS	POS	POS	POS	POS	NEG	POS	POS	POS
Iasi-14-150	SSTI	NEG	POS	NEG	NEG	NEG	POS	POS	AMB	POS	POS	POS	NEG	NEG	POS	POS	POS	POS	NEG	POS	POS	POS	POS	POS	POS	POS	NEG	POS	POS	POS	NEG	NEG	NEG	NEG	NEG	NEG	NEG	NEG	NEG	NEG	POS	POS	NEG	POS	POS	POS
Iasi-07-54	BSI	NEG	POS	NEG	NEG	NEG	POS	POS	POS	POS	NEG	NEG	NEG	NEG	POS	POS	POS	POS	NEG	POS	POS	POS	POS	POS	POS	POS	AMB	POS	NEG	POS	NEG	NEG	NEG	NEG	NEG	NEG	NEG	NEG	NEG	NEG	POS	NEG	POS	POS	POS	NEG
Iasi-16-188	BSI	NEG	POS	NEG	NEG	NEG	POS	POS	AMB	POS	NEG	NEG	NEG	NEG	POS	POS	POS	POS	NEG	POS	POS	POS	POS	POS	POS	POS	AMB	POS	NEG	POS	NEG	NEG	NEG	NEG	NEG	NEG	NEG	NEG	NEG	NEG	POS	NEG	POS	POS	POS	NEG
Iasi-32-318	BSI	NEG	POS	NEG	NEG	NEG	POS	POS	AMB	POS	NEG	NEG	NEG	NEG	POS	POS	POS	POS	NEG	POS	POS	POS	POS	POS	POS	POS	POS	NEG	NEG	NEG	NEG	NEG	NEG	NEG	NEG	NEG	NEG	NEG	NEG	NEG	POS	NEG	POS	POS	POS	NEG
Iasi-42-397	BSI	NEG	POS	NEG	NEG	NEG	POS	POS	NEG	POS	NEG	NEG	NEG	NEG	POS	POS	POS	POS	NEG	POS	POS	POS	POS	POS	POS	POS	NEG	POS	NEG	POS	NEG	NEG	NEG	NEG	NEG	NEG	NEG	NEG	NEG	NEG	POS	NEG	POS	POS	POS	NEG
Iasi-48-408	BSI	NEG	POS	NEG	NEG	NEG	POS	POS	AMB	POS	NEG	NEG	NEG	NEG	POS	POS	POS	POS	NEG	POS	POS	POS	POS	POS	POS	POS	NEG	POS	NEG	POS	NEG	NEG	NEG	NEG	NEG	NEG	NEG	NEG	NEG	NEG	POS	NEG	POS	POS	POS	NEG
Iasi-49-430	BSI	NEG	POS	NEG	NEG	NEG	POS	POS	POS	POS	NEG	NEG	NEG	NEG	POS	POS	POS	POS	NEG	POS	POS	POS	POS	POS	POS	POS	AMB	POS	NEG	POS	NEG	NEG	NEG	NEG	NEG	NEG	NEG	NEG	NEG	NEG	POS	NEG	POS	POS	POS	NEG
Iasi-52-52	BSI	NEG	POS	NEG	NEG	NEG	POS	POS	AMB	POS	NEG	NEG	NEG	NEG	POS	POS	POS	POS	NEG	POS	POS	POS	POS	POS	POS	POS	AMB	POS	NEG	POS	NEG	NEG	NEG	NEG	NEG	NEG	NEG	NEG	NEG	NEG	POS	NEG	POS	POS	POS	NEG
Iasi-01-2	SSTI	NEG	POS	NEG	NEG	NEG	POS	POS	POS	POS	NEG	NEG	NEG	NEG	POS	POS	POS	POS	NEG	POS	POS	POS	POS	POS	POS	POS	NEG	NEG	NEG	NEG	NEG	NEG	NEG	NEG	NEG	NEG	NEG	NEG	NEG	NEG	POS	NEG	POS	POS	POS	NEG
Iasi-10-101	SSTI	NEG	POS	NEG	NEG	NEG	POS	POS	AMB	POS	NEG	NEG	NEG	NEG	POS	POS	POS	POS	NEG	POS	POS	POS	POS	POS	POS	POS	NEG	POS	NEG	POS	NEG	NEG	NEG	NEG	NEG	NEG	NEG	NEG	NEG	NEG	POS	NEG	POS	POS	POS	NEG
Iasi-11-106	SSTI	NEG	POS	NEG	NEG	NEG	POS	POS	POS	POS	NEG	NEG	NEG	NEG	POS	POS	POS	POS	NEG	POS	POS	POS	POS	POS	POS	POS	NEG	POS	NEG	POS	NEG	NEG	NEG	NEG	NEG	NEG	NEG	NEG	NEG	NEG	POS	NEG	POS	POS	POS	NEG
Iasi-49-414	SSTI	NEG	POS	NEG	NEG	NEG	POS	POS	POS	POS	NEG	NEG	NEG	NEG	POS	POS	POS	POS	NEG	POS	POS	POS	POS	POS	POS	POS	AMB	POS	NEG	POS	NEG	NEG	NEG	NEG	NEG	NEG	NEG	NEG	NEG	NEG	POS	NEG	POS	POS	POS	NEG

Isolate	Group	VIRULENCE : ENTEROTOXINS					VIRULENCE : HLG AND LEUKOCIDINS													VIRULENCE : HAEMOLYSINS								VIRULENCE : HLB-CONV PHAGES			VIRULENCE : EXFOL.TOXINS			VIRULENCE : EPITHEL. DIFF. INHIB			VIRULENCE : ACME LOCUS				VIRULENCE : PROTEASES					
		egc cluster	enterotoxin Q	enterotoxin R	enterotoxin U		haemolysin gamma / leukocidin, component B	haemolysin gamma / leukocidin, component C		haemolysin gamma	Panton Valentine leukocidin F component	Panton Valentine leukocidin S component	F component from hypothetical leukocidin from ruminants	S component from hypothetical leukocidin from ruminants	leukocidin D component	leukocidin E component	leukocidin/ haemolysin toxin family protein	leukocidin/haemolysin toxin family protein		putative membrane protein	haemolysin alpha	putative membrane protein		haemolysin beta				staphylo-kinase	chemotaxis-inhibiting protein (CHIPS)	Staphyl. Comple-ment inhibitor	exfoliative toxin serotype A	exfoliative toxin serotype B	exfoliative toxin D	epidermal cell differen-tiation inhibitor	epidermal cell differen-tiation inhibitor B	epidermal cell differen-tiation inhibitor C	ACME-locus	ACME-locus: ornithincarb-amoyltrans-ferase	ACME-locus: carbamat-kinase	ACME-locus: arginine/orni-thine-antiporter	aureolysin			serin- protease A	serin- protease B	serin- protease E
		egc (total)	seq	ser	selu	ORF CM14	lukF	lukS	lukS (ST22+ST45)	hlgA	lukF-PV	lukS-PV	lukF-PV (P83)	lukM	lukD	lukE	lukX	lukY	lukY (ST30+ST45)	hl	hla	hlIII (consensus)	hlIII (other than RF122)	hlb_probe 1	hlb_probe 2	hlb_probe 3	un-disrupted hlb	sak	chp	scn	etA	etB	etD	edinA	edinB	edinC	arcA-SCC	arcB-SCC	arcC-SCC	arcD-SCC	aur (consensus)	aur (other than MRSA252)	aur (MRSA252)	splA	splB	splE
Iasi-05-46	misc. MRSA	NEG	POS	NEG	NEG	NEG	POS	POS	AMB	POS	NEG	NEG	NEG	NEG	POS	POS	POS	POS	NEG	POS	POS	POS	POS	POS	POS	POS	AMB	POS	NEG	POS	NEG	NEG	NEG	NEG	NEG	NEG	NEG	NEG	NEG	NEG	POS	NEG	POS	POS	POS	NEG
Iasi-06-47	misc. MRSA	NEG	POS	NEG	NEG	NEG	POS	POS	POS	POS	NEG	NEG	NEG	NEG	POS	POS	POS	POS	NEG	POS	POS	POS	POS	POS	POS	POS	AMB	POS	NEG	POS	NEG	NEG	NEG	NEG	NEG	NEG	NEG	NEG	NEG	NEG	POS	NEG	POS	POS	POS	NEG
Iasi-08-73	misc. MRSA	NEG	POS	NEG	NEG	NEG	POS	POS	POS	POS	NEG	NEG	NEG	NEG	POS	POS	POS	POS	NEG	POS	POS	POS	POS	POS	POS	POS	NEG	POS	NEG	POS	NEG	NEG	NEG	NEG	NEG	NEG	NEG	NEG	NEG	NEG	POS	NEG	POS	POS	POS	NEG
Iasi-19-194	misc. MRSA	NEG	POS	NEG	NEG	NEG	POS	POS	AMB	POS	NEG	NEG	NEG	NEG	POS	POS	POS	POS	NEG	POS	POS	POS	POS	POS	POS	POS	AMB	POS	NEG	POS	NEG	NEG	NEG	NEG	NEG	NEG	NEG	NEG	NEG	NEG	POS	NEG	POS	POS	POS	NEG
Iasi-21-219	misc. MRSA	NEG	POS	NEG	NEG	NEG	POS	POS	AMB	POS	NEG	NEG	NEG	NEG	POS	POS	POS	POS	NEG	POS	POS	POS	POS	POS	POS	POS	AMB	POS	NEG	POS	NEG	NEG	NEG	NEG	NEG	NEG	NEG	NEG	NEG	NEG	POS	NEG	POS	POS	POS	NEG
Iasi-25-284	misc. MRSA	NEG	POS	NEG	NEG	NEG	POS	POS	POS	POS	NEG	NEG	NEG	NEG	POS	POS	POS	POS	NEG	POS	POS	POS	POS	POS	POS	POS	AMB	POS	NEG	POS	NEG	NEG	NEG	NEG	NEG	NEG	NEG	NEG	NEG	NEG	POS	NEG	POS	POS	POS	NEG
Iasi-284	misc. MRSA	NEG	POS	NEG	NEG	NEG	POS	POS	AMB	POS	NEG	NEG	NEG	NEG	POS	POS	POS	POS	NEG	POS	POS	POS	POS	POS	POS	POS	AMB	POS	NEG	POS	NEG	NEG	NEG	NEG	NEG	NEG	NEG	NEG	NEG	NEG	POS	NEG	POS	POS	POS	NEG
Iasi-36-336	misc. MRSA	NEG	POS	NEG	NEG	NEG	POS	POS	AMB	POS	NEG	NEG	NEG	NEG	POS	POS	POS	POS	NEG	POS	POS	POS	POS	POS	POS	POS	NEG	POS	NEG	POS	NEG	NEG	NEG	NEG	NEG	NEG	NEG	NEG	NEG	NEG	POS	NEG	POS	POS	POS	NEG
Iasi-43-402	misc. MRSA	NEG	POS	NEG	NEG	NEG	POS	POS	POS	POS	NEG	NEG	NEG	NEG	POS	POS	POS	POS	NEG	POS	POS	POS	POS	POS	POS	POS	POS	POS	NEG	POS	NEG	NEG	NEG	NEG	NEG	NEG	NEG	NEG	NEG	NEG	POS	NEG	POS	POS	POS	NEG
Iasi-44-404	misc. MRSA	NEG	POS	NEG	NEG	NEG	POS	POS	NEG	POS	NEG	NEG	NEG	NEG	POS	POS	POS	POS	NEG	POS	POS	POS	POS	POS	POS	POS	NEG	POS	NEG	POS	NEG	NEG	NEG	NEG	NEG	NEG	NEG	NEG	NEG	NEG	POS	NEG	POS	POS	POS	NEG
Iasi-54-430	misc. MRSA	NEG	POS	NEG	AMB	NEG	POS	POS	POS	POS	NEG	NEG	NEG	NEG	POS	POS	POS	POS	NEG	POS	POS	POS	POS	POS	POS	POS	POS	POS	NEG	POS	NEG	NEG	NEG	NEG	NEG	NEG	NEG	NEG	NEG	NEG	POS	NEG	POS	POS	POS	NEG
Iasi-24-216	SSTI	POS	NEG	NEG	POS	NEG	POS	POS	POS	POS	NEG	NEG	NEG	NEG	NEG	POS	POS	POS	NEG	POS	POS	POS	NEG	POS	POS	NEG	NEG	POS	POS	POS	NEG	NEG	NEG	NEG	NEG	NEG	NEG	NEG	NEG	NEG	POS	NEG	POS	POS	POS	NEG
Iasi-05-S13	Carrier	POS	NEG	NEG	POS	NEG	POS	POS	POS	POS	NEG	NEG	NEG	NEG	NEG	POS	POS	POS	NEG	POS	POS	POS	NEG	POS	POS	NEG	NEG	POS	POS	POS	NEG	NEG	NEG	NEG	NEG	NEG	NEG	NEG	NEG	NEG	POS	NEG	POS	POS	POS	NEG
Iasi-45-362	BSI	NEG	NEG	NEG	NEG	NEG	POS	POS	POS	POS	NEG	NEG	NEG	NEG	POS	POS	POS	POS	NEG	POS	POS	POS	POS	NEG	NEG	POS	NEG	NEG	POS	POS	NEG	NEG	NEG	NEG	NEG	NEG	NEG	NEG	NEG	NEG	POS	POS	NEG	POS	POS	POS
Iasi-22-199	SSTI	NEG	NEG	NEG	NEG	NEG	POS	POS	AMB	POS	NEG	NEG	NEG	NEG	POS	POS	POS	POS	NEG	POS	POS	POS	POS	NEG	NEG	POS	NEG	NEG	POS	POS	NEG	NEG	NEG	NEG	NEG	NEG	NEG	NEG	NEG	NEG	POS	POS	NEG	POS	POS	POS
Iasi-12-S26	Carrier	NEG	NEG	NEG	NEG	NEG	POS	POS	POS	POS	NEG	NEG	NEG	NEG	POS	POS	POS	POS	NEG	POS	POS	POS	POS	NEG	NEG	POS	AMB	NEG	POS	POS	NEG	NEG	NEG	NEG	NEG	NEG	NEG	NEG	NEG	NEG	POS	POS	NEG	POS	POS	POS
Iasi-21-P2	Carrier	NEG	NEG	NEG	NEG	NEG	POS	POS	POS	POS	NEG	NEG	NEG	NEG	POS	POS	POS	POS	NEG	POS	POS	POS	POS	NEG	NEG	POS	AMB	NEG	POS	POS	NEG	NEG	NEG	NEG	NEG	NEG	NEG	NEG	NEG	NEG	POS	POS	NEG	POS	POS	POS
Iasi-06-60	BSI	POS	NEG	NEG	POS	NEG	POS	POS	POS	POS	NEG	NEG	NEG	NEG	NEG	NEG	POS	POS	NEG	POS	POS	NEG	NEG	POS	POS	POS	AMB	POS	NEG	POS	NEG	NEG	NEG	NEG	NEG	NEG	NEG	NEG	NEG	NEG	POS	NEG	POS	NEG	NEG	NEG
Iasi-12-79	BSI	POS	NEG	NEG	POS	NEG	POS	POS	POS	POS	NEG	NEG	NEG	NEG	NEG	NEG	POS	POS	NEG	POS	POS	NEG	NEG	POS	POS	POS	NEG	POS	POS	POS	NEG	NEG	NEG	NEG	NEG	NEG	NEG	NEG	NEG	NEG	POS	NEG	POS	NEG	NEG	NEG
Iasi-17-90	BSI	POS	NEG	NEG	POS	NEG	POS	POS	POS	POS	NEG	NEG	NEG	NEG	NEG	NEG	POS	POS	NEG	POS	POS	NEG	NEG	POS	POS	POS	NEG	POS	POS	POS	NEG	NEG	NEG	NEG	NEG	NEG	NEG	NEG	NEG	NEG	POS	NEG	POS	NEG	NEG	NEG
Iasi-21-105	BSI	POS	NEG	NEG	POS	NEG	POS	AMB	POS	POS	NEG	NEG	NEG	NEG	NEG	NEG	POS	POS	NEG	POS	POS	NEG	NEG	POS	POS	POS	NEG	POS	POS	POS	NEG	NEG	NEG	NEG	NEG	NEG	NEG	NEG	NEG	NEG	POS	NEG	POS	NEG	NEG	NEG
Iasi-22-109	BSI	POS	NEG	NEG	POS	NEG	POS	POS	POS	POS	NEG	NEG	NEG	NEG	NEG	NEG	POS	POS	NEG	POS	POS	NEG	NEG	POS	POS	POS	AMB	POS	POS	POS	NEG	NEG	NEG	NEG	NEG	NEG	NEG	NEG	NEG	NEG	POS	NEG	POS	NEG	NEG	NEG
Iasi-23-110	BSI	POS	NEG	NEG	POS	NEG	POS	POS	POS	POS	NEG	NEG	NEG	NEG	NEG	NEG	POS	POS	NEG	POS	POS	NEG	NEG	POS	POS	POS	POS	POS	POS	POS	NEG	NEG	NEG	NEG	NEG	NEG	NEG	NEG	NEG	NEG	POS	NEG	POS	NEG	NEG	NEG
Iasi-24-136	BSI	POS	NEG	NEG	POS	NEG	POS	POS	POS	POS	NEG	NEG	NEG	NEG	NEG	NEG	POS	POS	NEG	POS	POS	NEG	NEG	POS	POS	POS	NEG	POS	POS	POS	NEG	NEG	NEG	NEG	NEG	NEG	NEG	NEG	NEG	NEG	POS	NEG	POS	NEG	NEG	NEG
Iasi-34-224	BSI	POS	NEG	NEG	POS	NEG	POS	POS	POS	POS	NEG	NEG	NEG	NEG	NEG	NEG	POS	POS	NEG	POS	POS	NEG	NEG	POS	POS	POS	NEG	POS	POS	POS	NEG	NEG	NEG	NEG	NEG	NEG	NEG	NEG	NEG	NEG	POS	NEG	POS	NEG	NEG	NEG
Iasi-39-297	BSI	POS	NEG	NEG	POS	NEG	POS	POS	POS	POS	NEG	NEG	NEG	NEG	NEG	NEG	POS	POS	NEG	POS	POS	NEG	NEG	POS	POS	POS	AMB	POS	POS	POS	NEG	NEG	NEG	NEG	NEG	NEG	NEG	NEG	NEG	NEG	POS	NEG	POS	NEG	NEG	NEG
Iasi-50-431	BSI	POS	NEG	NEG	POS	NEG	POS	POS	POS	POS	NEG	NEG	NEG	NEG	NEG	NEG	POS	POS	NEG	POS	POS	NEG	NEG	POS	POS	POS	NEG	POS	POS	POS	NEG	NEG	NEG	NEG	NEG	NEG	NEG	NEG	NEG	NEG	POS	NEG	POS	NEG	NEG	NEG
Iasi-51-432	BSI	POS	NEG	NEG	POS	NEG	POS	POS	POS	POS	NEG	NEG	NEG	NEG	NEG	NEG	POS	POS	NEG	POS	POS	NEG	NEG	POS	POS	POS	AMB	POS	POS	POS	NEG	NEG	NEG	NEG	NEG	NEG	NEG	NEG	NEG	NEG	POS	NEG	POS	NEG	NEG	NEG
Iasi-08-76	SSTI	POS	NEG	NEG	POS	NEG	POS	AMB	POS	POS	NEG	NEG	NEG	NEG	NEG	NEG	POS	POS	NEG	POS	POS	NEG	NEG	POS	POS	POS	NEG	POS	POS	POS	NEG	NEG	NEG	NEG	NEG	NEG	NEG	NEG	NEG	NEG	POS	NEG	POS	NEG	NEG	NEG
Iasi-30-350	SSTI	POS	NEG	NEG	POS	NEG	POS	POS	POS	POS	NEG	NEG	NEG	NEG	NEG	NEG	POS	POS	NEG	POS	POS	NEG	NEG	POS	POS	POS	NEG	POS	POS	POS	NEG	NEG	NEG	NEG	NEG	NEG	NEG	NEG	NEG	NEG	POS	NEG	POS	NEG	NEG	NEG
Iasi-33-319	SSTI	POS	NEG	NEG	POS	NEG	AMB	NEG	POS	AMB	NEG	NEG	NEG	NEG	NEG	NEG	POS	POS	NEG	POS	AMB	NEG	NEG	POS	POS	POS	NEG	POS	NEG	POS	NEG	NEG	NEG	NEG	NEG	NEG	NEG	NEG	NEG	NEG	POS	NEG	POS	NEG	NEG	NEG
Iasi-40-383	SSTI	POS	NEG	NEG	POS	NEG	POS	POS	POS	POS	NEG	NEG	NEG	NEG	NEG	NEG	POS	POS	NEG	POS	POS	NEG	NEG	POS	POS	POS	NEG	POS	POS	POS	NEG	NEG	NEG	NEG	NEG	NEG	NEG	NEG	NEG	NEG	POS	NEG	POS	NEG	NEG	NEG
Iasi-49-423	SSTI	POS	NEG	NEG	POS	NEG	POS	POS	POS	POS	NEG	NEG	NEG	NEG	NEG	NEG	POS	POS	NEG	POS	POS	NEG	NEG	POS	POS	POS	NEG	POS	POS	POS	NEG	NEG	NEG	NEG	NEG	NEG	NEG	NEG	NEG	NEG	POS	NEG	POS	NEG	NEG	NEG
Iasi-16-S31	Carrier	POS	NEG	NEG	POS	NEG	POS	POS	POS	POS	NEG	NEG	NEG	NEG	NEG	NEG	POS	POS	NEG	POS	POS	NEG	NEG	POS	POS	POS	AMB	POS	POS	POS	NEG	NEG	NEG	NEG	NEG	NEG	NEG	NEG	NEG	NEG	POS	NEG	POS	NEG	NEG	NEG
Iasi-24-P9	Carrier	POS	NEG	NEG	POS	NEG	POS	POS	POS	POS	NEG	NEG	NEG	NEG	NEG	NEG	POS	POS	NEG	POS	POS	NEG	NEG	POS	POS	POS	AMB	POS	POS	POS	NEG	NEG	NEG	NEG	NEG	NEG	NEG	NEG	NEG	NEG	POS	NEG	POS	NEG	NEG	NEG
Iasi-27-P12	Carrier	POS	NEG	NEG	POS	NEG	POS	POS	POS	POS	NEG	NEG	NEG	NEG	NEG	NEG	POS	POS	NEG	POS	POS	NEG	NEG	POS	POS	POS	NEG	POS	POS	POS	NEG	NEG	NEG	NEG	NEG	NEG	NEG	NEG	NEG	NEG	POS	NEG	POS	NEG	NEG	NEG
Iasi-38-P27	Carrier	POS	NEG	NEG	POS	NEG	POS	POS	POS	POS	NEG	NEG	NEG	NEG	NEG	NEG	POS	POS	NEG	POS	POS	NEG	NEG	POS	POS	POS	AMB	POS	POS	POS	NEG	NEG	NEG	NEG	NEG	NEG	NEG	NEG	NEG	NEG	POS	NEG	POS	NEG	NEG	NEG
Iasi-42-G511	Carrier	POS	NEG	NEG	POS	NEG	POS	POS	POS	POS	NEG	NEG	NEG	NEG	NEG	NEG	POS	POS	NEG	POS	POS	NEG	NEG	POS	POS	POS	AMB	POS	POS	POS	NEG	NEG	NEG	NEG	NEG	NEG	NEG	NEG	NEG	NEG	POS	NEG	POS	NEG	NEG	NEG
Iasi-46-3N	Carrier	POS	NEG	NEG	POS	NEG	POS	POS	POS	POS	NEG	NEG	NEG	NEG	NEG	NEG	POS	POS	NEG	POS	POS	NEG	NEG	POS	POS	POS	POS	POS	POS	POS	NEG	NEG	NEG	NEG	NEG	NEG	NEG	NEG	NEG	NEG	POS	NEG	POS	NEG	NEG	NEG
Iasi-47-42O	Carrier	POS	NEG	NEG	POS	NEG	POS	POS	POS	POS	NEG	NEG	NEG	NEG	NEG	NEG	POS	POS	NEG	POS	POS	NEG	NEG	POS	POS	POS	AMB	POS	POS	POS	NEG	NEG	NEG	NEG	NEG	NEG	NEG	NEG	NEG	NEG	POS	NEG	POS	NEG	NEG	NEG
Iasi-48-36N	Carrier	POS	NEG	NEG	POS	NEG	POS	POS	POS	POS	NEG	NEG	NEG	NEG	NEG	NEG	POS	POS	NEG	POS	POS	NEG	NEG	POS	POS	POS	AMB	POS	POS	POS	NEG	NEG	NEG	NEG	NEG	NEG	NEG	NEG	NEG	NEG	POS	NEG	POS	NEG	NEG	NEG
Iasi-49-T3	Carrier	POS	NEG	NEG	POS	NEG	POS	POS	POS	POS	NEG	NEG	NEG	NEG	NEG	NEG	POS	POS	NEG	POS	POS	NEG	NEG	POS	POS	POS	AMB	POS	POS	POS	NEG	NEG	NEG	NEG	NEG	NEG	NEG	NEG	NEG	NEG	POS	NEG	POS	NEG	NEG	NEG
Iasi-50-T2	Carrier	POS	NEG	NEG	POS	NEG	POS	POS	POS	POS	NEG	NEG	NEG	NEG	NEG	NEG	POS	POS	NEG	POS	POS	NEG	NEG	POS	POS	POS	AMB	POS	POS	POS	NEG	NEG	NEG	NEG	NEG	NEG	NEG	NEG	NEG	NEG	POS	NEG	POS	NEG	NEG	NEG
Iasi-22-251	BSI	POS	NEG	NEG	POS	NEG	POS	POS	POS	POS	NEG	NEG	NEG	NEG	NEG	NEG	POS	POS	NEG	POS	POS	NEG	NEG	POS	POS	POS	NEG	POS	POS	POS	NEG	NEG	NEG	NEG	NEG	NEG	NEG	NEG	NEG	NEG	POS	NEG	POS	NEG	NEG	NEG
Iasi-27-158	BSI	POS	NEG	NEG	POS	NEG	POS	POS	POS	POS	NEG	NEG	NEG	NEG	POS	POS	POS	POS	NEG	POS	POS	POS	POS	POS	POS	POS	POS	POS	POS	POS	NEG	NEG	POS	NEG	POS	NEG	NEG	NEG	NEG	NEG	POS	POS	NEG	POS	POS	NEG
Iasi-08-70	BSI	POS	NEG	NEG	POS	NEG	POS	POS	POS	POS	NEG	NEG	NEG	NEG	NEG	NEG	POS	NEG	POS	POS	POS	POS	POS	POS	POS	POS	NEG	POS	NEG	POS	NEG	NEG	NEG	NEG	NEG	NEG	NEG	NEG	NEG	NEG	POS	NEG	POS	NEG	NEG	POS
Iasi-18-93	BSI	POS	NEG	NEG	POS	NEG	POS	POS	POS	POS	NEG	NEG	NEG	NEG	NEG	NEG	POS	NEG	POS	POS	POS	POS	POS	POS	POS	POS	NEG	POS	POS	POS	NEG	NEG	NEG	NEG	NEG	NEG	NEG	NEG	NEG	NEG	POS	NEG	POS	NEG	NEG	POS
Iasi-28-P14	Carrier	POS	NEG	NEG	POS	NEG	POS	POS	POS	POS	NEG	NEG	NEG	NEG	NEG	NEG	POS	NEG	POS	POS	POS	POS	POS	AMB	POS	NEG	NEG	POS	POS	POS	NEG	NEG	NEG	NEG	NEG	NEG	NEG	NEG	NEG	NEG	POS	NEG	POS	NEG	NEG	POS
Iasi-29-P15	Carrier	POS	NEG	NEG	POS	NEG	POS	POS	POS	POS	NEG	NEG	NEG	NEG	NEG	NEG	POS	NEG	POS	POS	POS	POS	POS	POS	POS	POS	POS	POS	POS	POS	NEG	NEG	NEG	NEG	NEG	NEG	NEG	NEG	NEG	NEG	POS	NEG	POS	NEG	NEG	POS
Iasi-03-19	BSI	POS	NEG	NEG	POS	NEG	POS	AMB	POS	POS	NEG	NEG	NEG	NEG	NEG	NEG	POS	NEG	POS	POS	POS	POS	NEG	NEG	NEG	NEG	NEG	POS	POS	POS	NEG	NEG	NEG	NEG	NEG	NEG	NEG	NEG	NEG	NEG	NEG	NEG	POS	NEG	NEG	NEG
Iasi-11-77	BSI	POS	NEG	NEG	POS	NEG	POS	AMB	POS	POS	NEG	NEG	NEG	NEG	NEG	NEG	POS	NEG	POS	POS	POS	POS	NEG	NEG	NEG	NEG	NEG	POS	POS	POS	NEG	NEG	NEG	NEG	NEG	NEG	NEG	NEG	NEG	NEG	NEG	NEG	POS	NEG	NEG	NEG
Iasi-13-82	BSI	POS	NEG	NEG	POS	NEG	POS	AMB	POS	POS	NEG	NEG	NEG	NEG	NEG	NEG	POS	NEG	POS	POS	POS	POS	NEG	NEG	NEG	NEG	NEG	POS	POS	POS	NEG	NEG	NEG	NEG	NEG	NEG	NEG	NEG	NEG	NEG	NEG	NEG	POS	NEG	NEG	NEG
Iasi-15-86	BSI	POS	NEG	NEG	POS	NEG	POS	POS	POS	POS	NEG	NEG	NEG	NEG	NEG	NEG	POS	NEG	POS	POS	POS	POS	NEG	NEG	NEG	NEG	NEG	POS	POS	POS	NEG	NEG	NEG	NEG	NEG	NEG	NEG	NEG	NEG	NEG	POS	NEG	POS	NEG	NEG	NEG
Iasi-20-103	BSI	POS	NEG	NEG	POS	NEG	POS	AMB	POS	POS	NEG	NEG	NEG	NEG	NEG	NEG	POS	NEG	POS	POS	POS	POS	NEG	NEG	NEG	NEG	NEG	POS	POS	POS	NEG	NEG	NEG	NEG	NEG	NEG	NEG	NEG	NEG	NEG	AMB	NEG	POS	NEG	NEG	NEG
Iasi-33-222	BSI	POS	NEG	NEG	POS	NEG	POS	POS	POS	POS	NEG	NEG	NEG	NEG	NEG	NEG	POS	NEG	POS	POS	POS	POS	NEG	NEG	NEG	NEG	NEG	POS	POS	POS	NEG	NEG	NEG	NEG	NEG	NEG	NEG	NEG	NEG	NEG	POS	NEG	POS	NEG	NEG	NEG
Iasi-44-360	BSI	POS	NEG	NEG	POS	NEG	POS	POS	POS	POS	NEG	NEG	NEG	NEG	NEG	NEG	POS	NEG	POS	POS	POS	POS	NEG	NEG	NEG	NEG	NEG	POS	POS	POS	NEG	NEG	NEG	NEG	NEG	NEG	NEG	NEG	NEG	NEG	POS	NEG	POS	NEG	NEG	NEG
Iasi-47-399	BSI	POS	NEG	NEG	POS	NEG	POS	POS	POS	POS	NEG	NEG	NEG	NEG	NEG	NEG	POS	NEG	POS	POS	POS	POS	NEG	NEG	NEG	NEG	NEG	POS	POS	POS	NEG	NEG	NEG	NEG	NEG	NEG	NEG	NEG	NEG	NEG	POS	NEG	POS	NEG	NEG	NEG
Iasi-34-368	SSTI	POS	NEG	NEG	POS	NEG	POS	POS	POS	POS	NEG	NEG	NEG	NEG	NEG	NEG	POS	NEG	POS	POS	POS	POS	NEG	NEG	NEG	NEG	NEG	POS	POS	POS	NEG	NEG	NEG	NEG	NEG	NEG	NEG	NEG	NEG	NEG	AMB	NEG	POS	NEG	NEG	NEG
Iasi-36-371	SSTI	POS	NEG	NEG	POS	NEG	POS	AMB	POS	POS	NEG	NEG	NEG	NEG	NEG	NEG	POS	NEG	POS	POS	POS	POS	NEG	NEG	NEG	NEG	NEG	POS	POS	POS	NEG	NEG	NEG	NEG	NEG	NEG	NEG	NEG	NEG	NEG	AMB	NEG	POS	NEG	NEG	NEG
Iasi-04-S12	Carrier	POS	NEG	NEG	POS	NEG	POS	POS	POS	POS	NEG	NEG	NEG	NEG	NEG	NEG	POS	NEG	POS	POS	POS	POS	NEG	NEG	NEG	NEG	NEG	POS	POS	POS	NEG	NEG	NEG	NEG	NEG	NEG	NEG	NEG	NEG	NEG	POS	NEG	POS	NEG	NEG	NEG
Iasi-06-S15	Carrier	POS	NEG	NEG	POS	NEG	POS	POS	POS	POS	NEG	NEG	NEG	NEG	NEG	NEG	POS	NEG	POS	POS	POS	POS	NEG	NEG	NEG	NEG	NEG	POS	POS	POS	NEG	NEG	NEG	NEG	NEG	NEG	NEG	NEG	NEG	NEG	POS	NEG	POS	NEG	NEG	NEG
Iasi-07-S16	Carrier	POS	NEG	NEG	POS	NEG	POS	POS	POS	POS	NEG	NEG	NEG	NEG	NEG	NEG	POS	NEG	POS	POS	POS	POS	NEG	NEG	NEG	NEG	NEG	POS	POS	POS	NEG	NEG	NEG	NEG	NEG	NEG	NEG	NEG	NEG	NEG	POS	NEG	POS	NEG	NEG	NEG
Iasi-11-S24	Carrier	POS	NEG	NEG	POS	NEG	POS	POS	POS	POS	NEG	NEG	NEG	NEG	NEG	NEG	POS	NEG	POS	POS	POS	POS	NEG	NEG	NEG	NEG	AMB	POS	POS	POS	NEG	NEG	NEG	NEG	NEG	NEG	NEG	NEG	NEG	NEG	POS	NEG	POS	NEG	NEG	NEG
Iasi-22-P4	Carrier	POS	NEG	NEG	POS	NEG	POS	POS	POS	POS	NEG	NEG	NEG	NEG	NEG	NEG	POS	NEG	POS	POS	POS	POS	NEG	NEG	NEG	NEG	POS	NEG	NEG	NEG	NEG	NEG	NEG	NEG	NEG	NEG	NEG	NEG	NEG	NEG	POS	NEG	POS	NEG	NEG	NEG
Iasi-34-P23	Carrier	POS	NEG	NEG	POS	NEG	POS	POS	POS	POS	NEG	NEG	NEG	NEG	NEG	NEG	POS	NEG	POS	POS	POS	POS	NEG	NEG	NEG	NEG	NEG	POS	POS	POS	NEG	NEG	NEG	NEG	NEG	NEG	NEG	NEG	NEG	NEG	POS	NEG	POS	NEG	NEG	NEG
Iasi-37-P26	Carrier	POS	NEG	NEG	POS	NEG	POS	POS	POS	POS	NEG	NEG	NEG	NEG	NEG	NEG	POS	NEG	POS	POS	POS	POS	NEG	NEG	NEG	NEG	AMB	POS	POS	POS	NEG	NEG	NEG	NEG	NEG	NEG	NEG	NEG	NEG	NEG	POS	NEG	POS	NEG	NEG	NEG
Iasi-30-196	BSI	POS	NEG	NEG	POS	NEG	POS	POS	POS	POS	POS	POS	NEG	NEG	NEG	NEG	POS	NEG	POS	POS	POS	POS	NEG	NEG	NEG	NEG	AMB	POS	POS	POS	NEG	NEG	NEG	NEG	NEG	NEG	NEG	NEG	NEG	NEG	POS	NEG	POS	NEG	NEG	NEG
Iasi-27-298	BSI	NEG	NEG	NEG	NEG	NEG	POS	POS	NEG	POS	POS	POS	NEG	NEG	POS	POS	POS	POS	NEG	POS	POS	POS	POS	POS	POS	AMB	NEG	POS	NEG	POS	NEG	NEG	POS	NEG	POS	NEG	NEG	NEG	NEG	NEG	POS	POS	NEG	POS	POS	NEG
Iasi-37-363	BSI	NEG	NEG	NEG	NEG	NEG	POS	POS	AMB	POS	POS	POS	NEG	NEG	POS	POS	POS	POS	NEG	POS	POS	POS	POS	POS	POS	POS	NEG	POS	NEG	POS	NEG	NEG	POS	NEG	POS	NEG	NEG	NEG	NEG	NEG	POS	POS	NEG	POS	POS	NEG
Iasi-38-372	BSI	NEG	NEG	NEG	NEG	NEG	POS	POS	AMB	POS	POS	POS	NEG	NEG	POS	POS	POS	POS	NEG	POS	POS	POS	POS	POS	POS	POS	NEG	POS	NEG	POS	NEG	NEG	POS	NEG	POS	NEG	NEG	NEG	NEG	NEG	POS	POS	NEG	POS	POS	NEG
Iasi-39-374	BSI	NEG	NEG	NEG	NEG	NEG	POS	POS	POS	POS	POS	POS	NEG	NEG	POS	POS	POS	POS	NEG	POS	POS	POS	POS	POS	POS	POS	POS	POS	AMB	POS	NEG	NEG	POS	NEG	POS	NEG	NEG	NEG	NEG	NEG	POS	POS	NEG	POS	POS	NEG
Iasi-28-299	SSTI	NEG	NEG	NEG	NEG	NEG	POS	POS	AMB	POS	POS	POS	NEG	NEG	POS	POS	POS	POS	NEG	POS	POS	POS	POS	POS	POS	POS	NEG	POS	NEG	POS	NEG	NEG	POS	NEG	POS	NEG	NEG	NEG	NEG	NEG	POS	POS	NEG	POS	POS	NEG
Iasi-07-65	BSI	NEG	NEG	NEG	NEG	NEG	POS	POS	POS	POS	NEG	NEG	NEG	NEG	POS	POS	POS	POS	NEG	POS	POS	POS	POS	POS	POS	POS	NEG	POS	NEG	POS	NEG	NEG	NEG	NEG	NEG	NEG	NEG	NEG	NEG	NEG	POS	POS	NEG	POS	POS	NEG
Iasi-19-99	BSI	NEG	NEG	NEG	NEG	NEG	POS	POS	POS	POS	NEG	NEG	NEG	NEG	POS	POS	POS	POS	NEG	POS	POS	POS	POS	POS	POS	POS	NEG	POS	NEG	POS	NEG	NEG	NEG	NEG	NEG	NEG	NEG	NEG	NEG	NEG	POS	POS	NEG	POS	POS	POS
Iasi-25-217	SSTI	NEG	NEG	NEG	NEG	NEG	POS	POS	POS	POS	NEG	NEG	NEG	NEG	POS	POS	POS	POS	NEG	POS	POS	POS	POS	POS	POS	POS	POS	NEG	NEG	NEG	NEG	NEG	NEG	NEG	NEG	NEG	NEG	NEG	NEG	NEG	POS	POS	NEG	POS	POS	POS
Iasi-23-P5	Carrier	NEG	NEG	NEG	NEG	NEG	POS	POS	POS	POS	NEG	NEG	NEG	NEG	POS	POS	POS	POS	NEG	POS	POS	POS	POS	POS	POS	POS	NEG	POS	NEG	POS	NEG	NEG	NEG	NEG	NEG	NEG	NEG	NEG	NEG	NEG	POS	POS	NEG	POS	POS	NEG
Iasi-43-G513	Carrier	NEG	NEG	NEG	NEG	NEG	POS	POS	POS	POS	NEG	NEG	NEG	NEG	POS	POS	POS	POS	NEG	POS	POS	POS	POS	POS	POS	POS	AMB	POS	NEG	POS	NEG	NEG	NEG	NEG	NEG	NEG	NEG	NEG	NEG	NEG	POS	POS	NEG	POS	POS	POS
Iasi-44-G514	Carrier	NEG	NEG	NEG	NEG	NEG	POS	POS	POS	POS	NEG	NEG	NEG	NEG	POS	POS	POS	POS	NEG	POS	POS	POS	POS	POS	POS	POS	POS	POS	AMB	POS	NEG	NEG	NEG	NEG	NEG	NEG	NEG	NEG	NEG	NEG	POS	POS	NEG	POS	POS	POS
Iasi-28-172	BSI	POS	NEG	NEG	POS	POS	POS	POS	POS	POS	NEG	NEG	NEG	NEG	POS	POS	POS	POS	NEG	POS	POS	POS	NEG	POS	POS	POS	POS	POS	NEG	POS	NEG	POS	NEG	NEG	NEG	POS	NEG	NEG	NEG	NEG	POS	POS	NEG	POS	POS	NEG
Iasi-42-358	BSI	POS	NEG	NEG	POS	POS	POS	POS	POS	POS	NEG	NEG	NEG	NEG	POS	POS	POS	POS	NEG	POS	POS	POS	NEG	POS	POS	POS	POS	POS	NEG	POS	POS	POS	NEG	NEG	NEG	POS	NEG	NEG	NEG	NEG	POS	POS	NEG	POS	POS	NEG
Iasi-48-403	BSI	POS	NEG	NEG	POS	POS	POS	POS	POS	POS	NEG	NEG	NEG	NEG	POS	POS	POS	POS	NEG	POS	POS	POS	NEG	POS	POS	POS	AMB	POS	NEG	POS	NEG	NEG	NEG	NEG	NEG	NEG	NEG	NEG	NEG	NEG	POS	POS	NEG	POS	POS	NEG
Iasi-26-153	BSI	POS	NEG	NEG	POS	POS	POS	POS	POS	POS	POS	POS	NEG	NEG	POS	POS	POS	POS	NEG	POS	POS	POS	NEG	POS	POS	POS	NEG	POS	NEG	POS	NEG	NEG	NEG	NEG	NEG	NEG	NEG	NEG	NEG	NEG	POS	POS	NEG	POS	POS	NEG
Iasi-29-190	BSI	POS	NEG	NEG	POS	POS	POS	POS	POS	POS	POS	POS	NEG	NEG	POS	POS	POS	POS	NEG	POS	POS	POS	NEG	POS	POS	POS	AMB	POS	NEG	POS	NEG	NEG	NEG	NEG	NEG	NEG	NEG	NEG	NEG	NEG	POS	POS	NEG	POS	POS	NEG
Iasi-31-201	BSI	POS	NEG	NEG	POS	POS	POS	POS	POS	POS	POS	POS	NEG	NEG	POS	POS	POS	POS	NEG	POS	POS	POS	NEG	POS	POS	POS	NEG	POS	NEG	POS	NEG	NEG	NEG	NEG	NEG	NEG	NEG	NEG	NEG	NEG	POS	POS	NEG	POS	POS	NEG
Iasi-37-289	BSI	POS	NEG	NEG	POS	POS	POS	POS	POS	POS	POS	POS	NEG	NEG	POS	POS	POS	POS	NEG	POS	POS	POS	NEG	POS	POS	POS	POS	POS	NEG	POS	NEG	NEG	NEG	NEG	NEG	NEG	NEG	NEG	NEG	NEG	POS	POS	NEG	POS	POS	NEG
Iasi-41-334	BSI	POS	NEG	NEG	POS	POS	POS	POS	POS	POS	POS	POS	NEG	NEG	POS	POS	POS	POS	NEG	POS	POS	POS	NEG	POS	POS	POS	POS	POS	NEG	POS	NEG	NEG	NEG	NEG	NEG	NEG	NEG	NEG	NEG	NEG	POS	POS	NEG	POS	POS	NEG
Iasi-52-433	BSI	POS	NEG	NEG	POS	POS	POS	POS	AMB	POS	POS	POS	NEG	NEG	POS	POS	POS	POS	NEG	POS	POS	POS	NEG	POS	POS	POS	NEG	POS	NEG	POS	NEG	NEG	NEG	NEG	NEG	NEG	NEG	NEG	NEG	NEG	POS	POS	NEG	POS	POS	NEG
Iasi-07-66	SSTI	POS	NEG	NEG	POS	POS	POS	POS	NEG	POS	POS	POS	NEG	NEG	AMB	POS	POS	POS	NEG	POS	POS	POS	NEG	POS	POS	POS	NEG	POS	NEG	POS	NEG	NEG	NEG	NEG	NEG	NEG	NEG	NEG	NEG	NEG	POS	POS	NEG	POS	POS	NEG
Iasi-23-211	SSTI	POS	NEG	NEG	POS	POS	POS	POS	POS	POS	POS	POS	NEG	NEG	POS	POS	POS	POS	NEG	POS	POS	POS	NEG	POS	POS	POS	AMB	POS	NEG	POS	NEG	NEG	NEG	NEG	NEG	NEG	NEG	NEG	NEG	NEG	POS	POS	NEG	POS	POS	NEG
Iasi-43-401	SSTI	POS	NEG	NEG	POS	POS	POS	POS	POS	POS	POS	POS	NEG	NEG	POS	POS	POS	POS	NEG	POS	POS	POS	NEG	POS	POS	POS	NEG	POS	NEG	POS	NEG	NEG	NEG	NEG	NEG	NEG	NEG	NEG	NEG	NEG	POS	POS	NEG	POS	POS	NEG
Iasi-47-418	SSTI	POS	NEG	NEG	POS	POS	POS	POS	POS	POS	POS	POS	NEG	NEG	POS	POS	POS	POS	NEG	POS	POS	POS	NEG	POS	POS	POS	NEG	POS	NEG	POS	NEG	NEG	NEG	NEG	NEG	NEG	NEG	NEG	NEG	NEG	POS	POS	NEG	POS	POS	NEG
Iasi-18-169	SSTI	NEG	NEG	NEG	NEG	NEG	POS	POS	POS	POS	NEG	NEG	NEG	NEG	POS	AMB	POS	POS	NEG	POS	POS	POS	NEG	POS	POS	POS	POS	NEG	NEG	NEG	NEG	NEG	NEG	NEG	NEG	NEG	NEG	NEG	NEG	NEG	POS	POS	NEG	POS	POS	NEG
Iasi-18-S34	Carrier	NEG	NEG	NEG	NEG	NEG	POS	POS	POS	POS	NEG	NEG	NEG	NEG	POS	POS	POS	POS	NEG	POS	POS	POS	POS	POS	POS	POS	AMB	POS	AMB	POS	NEG	NEG	NEG	NEG	NEG	NEG	NEG	NEG	NEG	NEG	POS	POS	NEG	POS	POS	POS
Iasi-36-276	BSI	NEG	NEG	NEG	NEG	NEG	POS	POS	POS	POS	NEG	NEG	NEG	NEG	NEG	POS	POS	POS	NEG	POS	POS	POS	POS	NEG	NEG	NEG	POS	POS	POS	POS	NEG	NEG	POS	NEG	POS	NEG	NEG	NEG	NEG	NEG	POS	NEG	POS	POS	POS	POS
Iasi-151	BSI	NEG	NEG	NEG	NEG	NEG	POS	POS	POS	POS	NEG	NEG	NEG	NEG	NEG	NEG	POS	POS	NEG	POS	POS	POS	POS	NEG	NEG	NEG	POS	NEG	NEG	NEG	NEG	NEG	NEG	NEG	NEG	NEG	NEG	NEG	NEG	NEG	POS	NEG	POS	NEG	NEG	NEG
Iasi-02-11	BSI	POS	POS	NEG	POS	NEG	POS	POS	POS	POS	NEG	NEG	NEG	NEG	POS	POS	POS	POS	NEG	POS	POS	POS	NEG	POS	POS	POS	AMB	POS	POS	POS	NEG	NEG	NEG	POS	NEG	NEG	NEG	NEG	NEG	NEG	POS	POS	NEG	POS	POS	NEG

Isolate	Group	VIRULENCE : PROTEASES				VIRULENCE : STAPHYLOCOCCAL SUPERANTIGEN/ENTEROTOXIN-LIKE GENES (SET/SSL)																																										
		glutamylendopeptidase	staphopain B, protease	staphopain A (staphylopain A), protease		staphyl. exotoxin-like protein	staphylococcal superantigen-like protein 1												staphylococcal superantigen-like protein 2		staphylococcal superantigen-like protein 3			staphylococcal superantigen-like protein 4		staphylococcal superantigen-like protein 5				staphylococcal superantigen-like protein 6		staphylococcal superantigen-like protein 7			staphylococcal superantigen-like protein 8		staphylococcal superantigen-like protein 9			staphylococcal superantigen-like protein 10			staphylococcal superantigene-like protein 11			
		sspA	sspB	sspP (consensus)	sspP (other than ST93)	setC	ssl01/set6_probe1_11	ssl01/set6_probe2_11	ssl01/set6_probe1_12	ssl01/set6_probe2_12	ssl01/set6_probe4_11	ssl01/set6_probeRF122	ssl01/set6 (COL)	ssl01/set6 (Mu50+N315)	ssl01/set6 (MW2+MSSA476)	ssl01/set6 (MRSA252)	ssl01/set6 (RF122)	ssl01/set6 (other alleles)	ssl02/set7	ssl02/set7 (MRSA252)	ssl03/set8_probe 1	ssl03/set8_probe 2	ssl03/set8 (MRSA252, SAR0424)	ssl04/set9	ssl04/set9 (MRSA252, SAR0425)	ssl05/set3_probe 1	ssl05/set3 (RF122, probe-611)	ssl05/set3_probe 2 (612)	ssl05/set3 (MRSA252)	ssl06/set21	ssl06 (NCTC8325+MW2)	ssl07/set1	ssl07/set1 (MRSA252)	ssl07/set1 (AF188836)	ssl08/set12_probe 1	ssl08/set12_probe 2	ssl09/set5_probe 1	ssl09/set5_probe 2	ssl09/set5 (MRSA252)	ssl10/set4	ssl10 (RF122)	ssl10/set4 (MRSA252)	ssl11/set2 (COL)	ssl11+set2(Mu50+N315)	ssl11+set2(MW2+MSSA476)	ssl11/set2 (MRSA252)
Iasi-01-4	BSI	POS	POS	POS	POS	POS	NEG	POS	NEG	POS	NEG	NEG	NEG	NEG	POS	NEG	NEG	NEG	POS	NEG	POS	POS	NEG	POS	NEG	POS	AMB	POS	NEG	POS	POS	POS	AMB	NEG	POS	POS	POS	POS	NEG	POS	NEG	AMB	NEG	NEG	POS	NEG
Iasi-05-32	BSI	POS	POS	POS	POS	POS	NEG	POS	NEG	POS	NEG	NEG	NEG	NEG	POS	NEG	NEG	NEG	POS	AMB	POS	POS	NEG	POS	NEG	POS	AMB	POS	NEG	POS	POS	AMB	POS	AMB	POS	POS	POS	POS	NEG	POS	AMB	AMB	NEG	NEG	POS	NEG
Iasi-16-88	BSI	POS	POS	POS	POS	POS	NEG	POS	AMB	POS	NEG	NEG	NEG	NEG	POS	NEG	NEG	NEG	POS	AMB	POS	POS	NEG	POS	NEG	POS	POS	AMB	NEG	POS	POS	AMB	POS	AMB	POS	POS	POS	POS	AMB	POS	AMB	AMB	NEG	NEG	POS	NEG
Iasi-25-141	BSI	POS	POS	POS	POS	POS	NEG	POS	AMB	POS	NEG	NEG	NEG	NEG	POS	NEG	NEG	NEG	POS	AMB	POS	POS	NEG	POS	NEG	POS	POS	AMB	NEG	POS	POS	AMB	POS	AMB	POS	POS	POS	POS	AMB	POS	AMB	AMB	NEG	NEG	POS	NEG
Iasi-32-215	BSI	POS	POS	POS	POS	POS	AMB	POS	AMB	POS	AMB	NEG	NEG	NEG	POS	NEG	NEG	NEG	POS	AMB	POS	POS	NEG	POS	NEG	POS	POS	AMB	NEG	POS	POS	AMB	POS	AMB	POS	POS	POS	POS	AMB	AMB	AMB	POS	NEG	NEG	POS	NEG
Iasi-43-359	BSI	POS	POS	POS	POS	POS	NEG	POS	NEG	POS	NEG	NEG	NEG	NEG	POS	NEG	NEG	NEG	POS	AMB	POS	POS	NEG	POS	NEG	POS	AMB	POS	NEG	POS	POS	POS	AMB	AMB	POS	POS	POS	POS	NEG	POS	AMB	AMB	NEG	NEG	POS	NEG
Iasi-03-27	SSTI	POS	POS	POS	POS	POS	NEG	POS	NEG	POS	NEG	NEG	NEG	NEG	POS	NEG	NEG	NEG	POS	AMB	POS	POS	NEG	POS	NEG	POS	AMB	POS	NEG	POS	POS	POS	AMB	NEG	POS	POS	POS	POS	NEG	POS	NEG	AMB	NEG	NEG	POS	NEG
Iasi-13-149	SSTI	POS	POS	POS	POS	POS	NEG	POS	NEG	POS	NEG	NEG	NEG	NEG	POS	NEG	NEG	NEG	POS	AMB	POS	POS	NEG	POS	NEG	POS	AMB	POS	NEG	POS	POS	POS	AMB	NEG	POS	POS	POS	POS	NEG	POS	NEG	AMB	NEG	NEG	POS	NEG
Iasi-29-337	SSTI	POS	POS	POS	POS	POS	NEG	POS	NEG	POS	NEG	NEG	NEG	NEG	POS	NEG	NEG	NEG	POS	NEG	POS	POS	NEG	POS	NEG	POS	NEG	POS	NEG	POS	POS	POS	NEG	NEG	POS	POS	POS	POS	NEG	POS	NEG	NEG	NEG	NEG	POS	NEG
Iasi-31-355	SSTI	POS	POS	POS	POS	POS	AMB	POS	NEG	POS	NEG	NEG	NEG	NEG	POS	NEG	NEG	NEG	POS	AMB	POS	POS	NEG	POS	NEG	POS	AMB	POS	NEG	POS	POS	POS	AMB	AMB	POS	POS	POS	POS	NEG	POS	NEG	AMB	NEG	NEG	POS	NEG
Iasi-38-377	SSTI	POS	POS	POS	POS	POS	NEG	POS	NEG	POS	NEG	NEG	NEG	NEG	POS	NEG	NEG	NEG	POS	AMB	POS	POS	NEG	POS	NEG	POS	AMB	POS	NEG	POS	POS	POS	AMB	AMB	POS	POS	POS	POS	NEG	POS	NEG	AMB	NEG	NEG	POS	NEG
Iasi-41-388	SSTI	POS	POS	POS	POS	POS	NEG	POS	NEG	POS	NEG	NEG	NEG	NEG	POS	NEG	NEG	NEG	POS	AMB	POS	POS	NEG	POS	NEG	POS	AMB	POS	NEG	POS	POS	POS	AMB	AMB	POS	POS	POS	POS	NEG	POS	AMB	AMB	NEG	NEG	POS	NEG
Iasi-46-417	SSTI	POS	POS	POS	POS	POS	NEG	POS	NEG	POS	NEG	NEG	NEG	NEG	POS	NEG	NEG	NEG	POS	AMB	POS	POS	NEG	POS	NEG	POS	AMB	POS	NEG	POS	POS	POS	AMB	AMB	POS	POS	POS	POS	NEG	POS	AMB	AMB	NEG	NEG	POS	NEG
Iasi-02-S9	Carrier	POS	POS	POS	POS	POS	NEG	POS	NEG	POS	NEG	NEG	NEG	NEG	POS	NEG	NEG	NEG	POS	AMB	POS	POS	NEG	POS	POS	POS	AMB	POS	NEG	POS	POS	AMB	POS	AMB	POS	POS	POS	POS	AMB	POS	AMB	AMB	NEG	NEG	POS	NEG
Iasi-17-S33	Carrier	POS	POS	POS	POS	POS	NEG	POS	NEG	POS	NEG	NEG	NEG	NEG	POS	NEG	NEG	NEG	POS	AMB	POS	POS	NEG	POS	NEG	POS	AMB	POS	NEG	POS	POS	AMB	POS	AMB	POS	POS	POS	POS	NEG	POS	AMB	AMB	NEG	NEG	POS	NEG
Iasi-30-P17	Carrier	POS	POS	POS	POS	POS	NEG	POS	NEG	POS	NEG	NEG	NEG	NEG	POS	NEG	NEG	NEG	POS	AMB	POS	POS	NEG	POS	NEG	POS	AMB	POS	NEG	POS	POS	AMB	POS	AMB	POS	POS	POS	POS	NEG	POS	AMB	AMB	NEG	NEG	POS	NEG
Iasi-39-P28	Carrier	POS	POS	POS	POS	POS	NEG	POS	NEG	POS	NEG	NEG	NEG	NEG	POS	NEG	NEG	NEG	POS	AMB	POS	POS	NEG	POS	NEG	POS	AMB	POS	NEG	POS	POS	POS	AMB	AMB	POS	POS	POS	POS	NEG	POS	AMB	AMB	NEG	NEG	POS	NEG
Iasi-04-26	BSI	POS	POS	POS	POS	POS	NEG	POS	NEG	POS	NEG	NEG	NEG	NEG	POS	NEG	NEG	NEG	POS	AMB	POS	POS	NEG	POS	NEG	POS	POS	AMB	NEG	POS	POS	AMB	POS	AMB	POS	POS	POS	POS	AMB	POS	AMB	AMB	NEG	NEG	POS	NEG
Iasi-09-72	BSI	POS	POS	POS	POS	POS	NEG	POS	NEG	POS	NEG	NEG	NEG	NEG	POS	NEG	NEG	NEG	POS	NEG	POS	POS	NEG	POS	NEG	POS	AMB	POS	NEG	POS	POS	POS	AMB	NEG	POS	POS	POS	POS	NEG	POS	NEG	AMB	NEG	NEG	POS	NEG
Iasi-11-131	BSI	POS	POS	POS	POS	POS	NEG	POS	NEG	POS	NEG	NEG	NEG	NEG	POS	NEG	NEG	NEG	POS	NEG	POS	POS	NEG	POS	NEG	POS	AMB	POS	NEG	POS	POS	POS	AMB	NEG	POS	POS	POS	POS	NEG	POS	NEG	AMB	NEG	NEG	POS	NEG
Iasi-14-143	BSI	POS	POS	POS	POS	POS	AMB	POS	NEG	POS	NEG	NEG	NEG	NEG	POS	NEG	NEG	NEG	POS	NEG	POS	POS	NEG	POS	NEG	POS	AMB	POS	NEG	POS	POS	POS	AMB	NEG	POS	POS	POS	POS	NEG	POS	NEG	AMB	NEG	NEG	POS	NEG
Iasi-18-192	BSI	POS	POS	POS	POS	POS	NEG	POS	NEG	POS	NEG	NEG	NEG	NEG	POS	NEG	NEG	NEG	POS	AMB	POS	POS	NEG	POS	NEG	POS	AMB	POS	NEG	POS	POS	POS	AMB	NEG	POS	POS	POS	POS	NEG	POS	NEG	AMB	NEG	NEG	POS	NEG
Iasi-23-253	BSI	POS	POS	POS	POS	POS	NEG	POS	AMB	POS	NEG	NEG	NEG	NEG	POS	NEG	NEG	NEG	POS	AMB	POS	POS	NEG	POS	NEG	POS	AMB	POS	NEG	POS	POS	AMB	POS	AMB	POS	POS	POS	POS	AMB	POS	AMB	AMB	NEG	NEG	POS	NEG
Iasi-35-322	BSI	POS	POS	POS	POS	POS	NEG	POS	NEG	POS	NEG	NEG	NEG	NEG	POS	NEG	NEG	NEG	POS	AMB	POS	POS	NEG	POS	NEG	POS	AMB	POS	NEG	POS	POS	POS	AMB	NEG	POS	POS	POS	POS	NEG	POS	NEG	AMB	NEG	NEG	POS	NEG
Iasi-40-380	BSI	POS	POS	POS	POS	POS	NEG	POS	AMB	POS	NEG	NEG	NEG	NEG	POS	NEG	NEG	NEG	POS	AMB	POS	POS	NEG	POS	NEG	POS	AMB	POS	NEG	POS	POS	AMB	POS	AMB	POS	POS	POS	POS	AMB	POS	AMB	AMB	NEG	NEG	POS	NEG
Iasi-46-406	BSI	POS	POS	POS	POS	POS	NEG	POS	NEG	POS	NEG	NEG	NEG	NEG	POS	NEG	NEG	NEG	POS	AMB	POS	POS	NEG	POS	NEG	POS	AMB	POS	NEG	POS	POS	POS	AMB	AMB	POS	POS	POS	POS	NEG	POS	AMB	AMB	NEG	NEG	POS	NEG
Iasi-02-6	SSTI	POS	POS	POS	POS	POS	NEG	POS	NEG	POS	NEG	NEG	NEG	NEG	POS	NEG	NEG	NEG	POS	NEG	POS	POS	NEG	POS	NEG	POS	AMB	POS	NEG	POS	POS	POS	AMB	NEG	POS	POS	POS	POS	NEG	POS	NEG	AMB	NEG	NEG	POS	NEG
Iasi-09-97	SSTI	POS	POS	POS	POS	POS	NEG	POS	NEG	POS	NEG	NEG	NEG	NEG	POS	NEG	NEG	NEG	POS	AMB	POS	POS	NEG	POS	NEG	POS	AMB	POS	NEG	POS	POS	POS	AMB	NEG	POS	POS	POS	POS	NEG	POS	NEG	AMB	NEG	NEG	POS	NEG
Iasi-19-182	SSTI	POS	POS	POS	POS	POS	NEG	POS	NEG	POS	NEG	NEG	NEG	NEG	POS	NEG	NEG	NEG	POS	NEG	POS	POS	NEG	POS	NEG	POS	AMB	POS	NEG	POS	POS	POS	AMB	NEG	POS	POS	POS	POS	NEG	POS	NEG	AMB	NEG	NEG	POS	NEG
Iasi-31-314	SSTI	POS	POS	POS	POS	POS	NEG	POS	NEG	POS	NEG	NEG	NEG	NEG	POS	NEG	NEG	NEG	POS	AMB	POS	POS	NEG	POS	NEG	POS	AMB	POS	NEG	POS	POS	POS	AMB	NEG	POS	POS	POS	POS	NEG	POS	NEG	AMB	NEG	NEG	POS	NEG
Iasi-33-367	SSTI	POS	POS	POS	POS	POS	NEG	POS	NEG	POS	NEG	NEG	NEG	NEG	POS	NEG	NEG	NEG	POS	AMB	POS	POS	NEG	POS	NEG	POS	AMB	POS	NEG	POS	POS	POS	AMB	NEG	POS	POS	POS	POS	NEG	POS	NEG	AMB	NEG	NEG	POS	NEG
Iasi-39-378	SSTI	POS	POS	POS	POS	POS	NEG	POS	NEG	POS	NEG	NEG	NEG	NEG	POS	NEG	NEG	NEG	POS	AMB	POS	POS	NEG	POS	NEG	POS	AMB	POS	NEG	POS	POS	POS	AMB	AMB	POS	POS	POS	POS	NEG	POS	AMB	AMB	NEG	NEG	POS	NEG
Iasi-42-394	SSTI	POS	POS	POS	POS	POS	NEG	POS	NEG	POS	NEG	NEG	NEG	NEG	POS	NEG	NEG	NEG	POS	AMB	POS	POS	NEG	POS	NEG	POS	AMB	POS	NEG	POS	POS	POS	AMB	NEG	POS	POS	POS	POS	NEG	POS	NEG	AMB	NEG	NEG	POS	NEG
Iasi-44-409	SSTI	POS	POS	POS	POS	POS	NEG	POS	NEG	POS	NEG	NEG	NEG	NEG	POS	NEG	NEG	NEG	POS	AMB	POS	POS	NEG	POS	NEG	POS	AMB	POS	NEG	POS	POS	POS	AMB	AMB	POS	POS	POS	POS	NEG	POS	NEG	AMB	NEG	NEG	POS	NEG
Iasi-45-405	SSTI	POS	POS	POS	POS	POS	NEG	POS	NEG	POS	NEG	NEG	NEG	NEG	POS	NEG	NEG	NEG	POS	AMB	POS	POS	NEG	POS	NEG	POS	AMB	POS	NEG	POS	POS	POS	AMB	AMB	POS	POS	POS	POS	NEG	POS	AMB	AMB	NEG	NEG	POS	NEG
Iasi-50-425	SSTI	POS	POS	POS	POS	POS	NEG	POS	NEG	POS	NEG	NEG	NEG	NEG	POS	NEG	NEG	NEG	POS	AMB	POS	POS	NEG	POS	NEG	POS	AMB	POS	NEG	POS	POS	POS	AMB	NEG	POS	POS	POS	POS	NEG	POS	AMB	AMB	NEG	NEG	POS	NEG
Iasi-50-428	SSTI	POS	POS	POS	POS	POS	NEG	POS	NEG	POS	NEG	NEG	NEG	NEG	POS	NEG	NEG	NEG	POS	AMB	POS	POS	NEG	POS	NEG	POS	AMB	POS	NEG	POS	POS	POS	AMB	AMB	POS	POS	POS	POS	NEG	POS	AMB	AMB	NEG	NEG	POS	NEG
Iasi-51-37	SSTI	POS	POS	POS	POS	POS	NEG	POS	NEG	POS	NEG	NEG	NEG	NEG	POS	NEG	NEG	NEG	POS	AMB	POS	POS	NEG	POS	NEG	POS	AMB	POS	NEG	POS	POS	POS	AMB	AMB	POS	POS	POS	POS	NEG	POS	AMB	AMB	NEG	NEG	POS	NEG
Iasi-01-S8	Carrier	POS	POS	POS	POS	POS	NEG	POS	NEG	POS	NEG	NEG	NEG	NEG	POS	NEG	NEG	NEG	POS	AMB	POS	POS	NEG	POS	NEG	POS	AMB	POS	NEG	POS	POS	AMB	POS	AMB	POS	POS	POS	POS	NEG	POS	AMB	AMB	NEG	NEG	POS	NEG
Iasi-08-S17	Carrier	POS	POS	POS	POS	POS	NEG	POS	NEG	POS	NEG	NEG	NEG	NEG	POS	NEG	NEG	NEG	POS	AMB	POS	POS	NEG	POS	NEG	POS	AMB	POS	NEG	POS	POS	POS	AMB	AMB	POS	POS	POS	POS	NEG	POS	AMB	AMB	NEG	NEG	POS	NEG
Iasi-10-S23	Carrier	POS	POS	POS	POS	POS	NEG	POS	NEG	POS	NEG	NEG	NEG	NEG	POS	NEG	NEG	NEG	POS	AMB	POS	POS	NEG	POS	NEG	POS	AMB	POS	NEG	POS	POS	AMB	POS	AMB	POS	POS	POS	POS	NEG	POS	AMB	AMB	NEG	NEG	POS	NEG
Iasi-13-S27	Carrier	POS	POS	POS	POS	POS	NEG	POS	NEG	POS	NEG	NEG	NEG	NEG	POS	NEG	NEG	NEG	POS	AMB	POS	POS	NEG	POS	NEG	POS	AMB	POS	NEG	POS	POS	AMB	POS	AMB	POS	POS	POS	POS	NEG	POS	AMB	AMB	NEG	NEG	POS	NEG
Iasi-14-S28	Carrier	POS	POS	POS	POS	POS	NEG	POS	NEG	POS	NEG	NEG	NEG	NEG	POS	NEG	NEG	NEG	POS	AMB	POS	POS	NEG	POS	NEG	POS	AMB	POS	NEG	POS	POS	AMB	POS	AMB	POS	POS	POS	POS	NEG	POS	AMB	AMB	NEG	NEG	POS	NEG
Iasi-15-S29	Carrier	POS	POS	POS	POS	POS	AMB	POS	NEG	POS	NEG	NEG	NEG	NEG	POS	NEG	NEG	NEG	POS	AMB	POS	POS	NEG	POS	NEG	POS	AMB	POS	NEG	POS	POS	AMB	POS	AMB	POS	POS	POS	POS	AMB	AMB	AMB	POS	NEG	NEG	POS	NEG
Iasi-25-P10	Carrier	POS	POS	POS	POS	POS	NEG	POS	NEG	POS	NEG	NEG	NEG	NEG	POS	NEG	NEG	NEG	POS	AMB	POS	POS	NEG	POS	AMB	POS	POS	AMB	NEG	POS	POS	AMB	POS	AMB	POS	POS	POS	POS	NEG	AMB	AMB	POS	NEG	NEG	POS	NEG
Iasi-26-P11	Carrier	POS	POS	POS	POS	POS	NEG	POS	NEG	POS	NEG	NEG	NEG	NEG	POS	NEG	NEG	NEG	POS	AMB	POS	POS	NEG	POS	NEG	POS	AMB	POS	NEG	POS	POS	AMB	POS	AMB	POS	POS	POS	POS	NEG	POS	AMB	AMB	NEG	NEG	POS	NEG
Iasi-32-P19	Carrier	POS	POS	POS	POS	POS	NEG	POS	NEG	POS	NEG	NEG	NEG	NEG	POS	NEG	NEG	NEG	POS	AMB	POS	POS	NEG	POS	NEG	POS	AMB	POS	NEG	POS	POS	AMB	POS	AMB	POS	POS	POS	POS	NEG	POS	AMB	AMB	NEG	NEG	POS	NEG
Iasi-33-P22	Carrier	POS	POS	POS	POS	POS	NEG	POS	NEG	POS	NEG	NEG	NEG	NEG	POS	NEG	NEG	NEG	POS	AMB	POS	POS	NEG	POS	NEG	POS	AMB	POS	NEG	POS	POS	AMB	POS	AMB	POS	POS	POS	POS	NEG	POS	AMB	AMB	NEG	NEG	POS	NEG
Iasi-41-P44	Carrier	POS	POS	POS	POS	POS	NEG	POS	NEG	POS	NEG	NEG	NEG	NEG	POS	NEG	NEG	NEG	POS	AMB	POS	POS	NEG	POS	NEG	POS	AMB	POS	NEG	POS	POS	AMB	POS	AMB	POS	POS	POS	POS	NEG	POS	AMB	AMB	NEG	NEG	POS	NEG
Iasi-45-G515	Carrier	POS	POS	POS	POS	POS	NEG	POS	NEG	POS	NEG	NEG	NEG	NEG	POS	NEG	NEG	NEG	POS	AMB	POS	POS	NEG	POS	NEG	POS	AMB	POS	NEG	POS	POS	POS	AMB	AMB	POS	POS	POS	POS	NEG	POS	AMB	AMB	NEG	NEG	POS	NEG
Iasi-02-39	misc. MRSA	POS	POS	POS	POS	POS	NEG	POS	NEG	POS	NEG	NEG	NEG	NEG	POS	NEG	NEG	NEG	POS	NEG	POS	POS	NEG	POS	NEG	POS	AMB	POS	NEG	POS	POS	POS	AMB	NEG	POS	POS	POS	POS	NEG	POS	NEG	AMB	NEG	NEG	POS	NEG
Iasi-24-263	misc. MRSA	POS	POS	POS	POS	POS	NEG	POS	NEG	POS	NEG	NEG	NEG	NEG	POS	NEG	NEG	NEG	POS	AMB	POS	POS	NEG	POS	NEG	POS	AMB	POS	NEG	POS	POS	POS	AMB	AMB	POS	POS	POS	POS	AMB	POS	AMB	AMB	NEG	NEG	POS	NEG
Iasi-26-285	misc. MRSA	POS	POS	POS	POS	POS	NEG	POS	NEG	POS	NEG	NEG	NEG	NEG	POS	NEG	NEG	NEG	POS	AMB	POS	POS	NEG	POS	NEG	POS	AMB	POS	NEG	POS	POS	POS	AMB	AMB	POS	POS	POS	POS	NEG	POS	AMB	AMB	NEG	NEG	POS	NEG
Iasi-30-306	misc. MRSA	POS	POS	POS	POS	POS	NEG	POS	AMB	POS	NEG	NEG	NEG	NEG	POS	NEG	NEG	NEG	POS	AMB	POS	POS	NEG	POS	NEG	POS	AMB	POS	NEG	POS	POS	AMB	POS	AMB	POS	POS	POS	POS	AMB	POS	AMB	AMB	NEG	NEG	POS	NEG
Iasi-41-390	misc. MRSA	POS	POS	POS	POS	POS	NEG	POS	NEG	POS	NEG	NEG	NEG	NEG	POS	NEG	NEG	NEG	POS	AMB	POS	POS	NEG	POS	NEG	POS	AMB	POS	NEG	POS	POS	POS	AMB	AMB	POS	POS	POS	POS	NEG	POS	AMB	AMB	NEG	NEG	POS	NEG
Iasi-47-407	misc. MRSA	POS	POS	POS	POS	POS	NEG	POS	AMB	POS	NEG	NEG	NEG	NEG	POS	NEG	NEG	NEG	POS	AMB	POS	POS	NEG	POS	NEG	POS	POS	AMB	NEG	POS	POS	AMB	POS	AMB	POS	POS	POS	POS	AMB	AMB	AMB	POS	NEG	NEG	POS	NEG
Iasi-55-408CP	misc. MRSA	POS	POS	POS	POS	POS	NEG	POS	NEG	POS	NEG	NEG	NEG	NEG	POS	NEG	NEG	NEG	POS	AMB	POS	POS	NEG	POS	NEG	POS	AMB	POS	NEG	POS	POS	POS	AMB	NEG	POS	POS	POS	POS	NEG	POS	NEG	AMB	NEG	NEG	POS	NEG
Iasi-15-151	BSI	POS	POS	POS	POS	POS	POS	AMB	POS	AMB	POS	NEG	POS	AMB	NEG	NEG	NEG	NEG	POS	AMB	POS	POS	NEG	POS	NEG	POS	AMB	POS	NEG	NEG	NEG	POS	AMB	AMB	POS	POS	POS	POS	AMB	POS	AMB	AMB	NEG	POS	NEG	NEG
Iasi-46-386	BSI	POS	POS	POS	POS	POS	POS	POS	POS	AMB	POS	NEG	POS	AMB	NEG	POS	NEG	NEG	POS	AMB	POS	POS	NEG	POS	AMB	POS	AMB	POS	NEG	NEG	AMB	AMB	POS	AMB	POS	POS	POS	POS	AMB	POS	AMB	AMB	NEG	POS	NEG	NEG
Iasi-21-195	SSTI	POS	POS	POS	POS	POS	POS	NEG	POS	NEG	POS	NEG	NEG	POS	NEG	NEG	NEG	NEG	POS	NEG	POS	POS	NEG	POS	NEG	POS	#ZAHL!	#ZAHL!	NEG	NEG	NEG	POS	AMB	NEG	POS	POS	POS	POS	NEG	POS	AMB	AMB	NEG	POS	NEG	NEG
Iasi-37-375	SSTI	POS	POS	POS	POS	POS	POS	NEG	POS	NEG	POS	NEG	NEG	POS	NEG	NEG	NEG	NEG	POS	AMB	POS	POS	NEG	POS	NEG	POS	AMB	POS	NEG	NEG	NEG	POS	AMB	NEG	POS	POS	POS	POS	NEG	POS	NEG	AMB	NEG	POS	NEG	NEG
Iasi-45-415	SSTI	POS	POS	POS	POS	POS	POS	NEG	POS	NEG	POS	NEG	NEG	POS	NEG	NEG	NEG	NEG	POS	AMB	POS	POS	NEG	POS	NEG	POS	AMB	POS	NEG	NEG	NEG	POS	AMB	NEG	POS	POS	POS	POS	NEG	POS	AMB	AMB	NEG	POS	NEG	NEG
Iasi-09-S19	Carrier	POS	POS	POS	POS	POS	POS	POS	POS	POS	POS	NEG	POS	AMB	POS	POS	NEG	NEG	POS	AMB	POS	POS	NEG	POS	NEG	POS	AMB	POS	NEG	NEG	NEG	AMB	POS	AMB	POS	POS	POS	POS	NEG	POS	AMB	AMB	NEG	POS	NEG	NEG
Iasi-53-434	BSI	POS	POS	POS	POS	POS	POS	POS	POS	POS	POS	NEG	POS	AMB	POS	POS	NEG	NEG	POS	AMB	POS	POS	NEG	POS	AMB	POS	POS	AMB	NEG	NEG	AMB	AMB	POS	AMB	POS	POS	POS	POS	AMB	POS	AMB	AMB	NEG	POS	NEG	NEG
Iasi-03-40	misc. MRSA	POS	POS	POS	POS	POS	POS	AMB	POS	AMB	POS	NEG	NEG	POS	NEG	NEG	NEG	NEG	POS	AMB	POS	POS	NEG	POS	NEG	POS	AMB	POS	NEG	NEG	NEG	POS	AMB	AMB	POS	POS	POS	POS	AMB	POS	AMB	AMB	NEG	POS	NEG	NEG
Iasi-04-43	misc. MRSA	POS	POS	POS	POS	POS	POS	NEG	POS	NEG	POS	NEG	NEG	POS	NEG	NEG	NEG	NEG	POS	AMB	POS	POS	NEG	POS	NEG	POS	AMB	POS	NEG	NEG	NEG	POS	AMB	NEG	POS	POS	POS	POS	NEG	POS	NEG	AMB	NEG	POS	NEG	NEG
Iasi-01-20	misc. MRSA	POS	POS	POS	POS	POS	POS	AMB	POS	AMB	POS	NEG	POS	AMB	NEG	NEG	NEG	NEG	POS	AMB	POS	POS	NEG	POS	NEG	POS	AMB	POS	NEG	NEG	NEG	AMB	POS	AMB	POS	POS	POS	POS	NEG	POS	AMB	AMB	NEG	POS	NEG	NEG
Iasi-35-228	BSI	POS	POS	POS	POS	POS	NEG	NEG	POS	NEG	POS	POS	NEG	NEG	NEG	NEG	NEG	POS	POS	AMB	POS	POS	NEG	POS	NEG	POS	AMB	POS	AMB	NEG	NEG	POS	NEG	NEG	POS	POS	POS	POS	NEG	POS	AMB	AMB	NEG	POS	NEG	NEG
Iasi-38-293	BSI	POS	POS	POS	POS	POS	POS	NEG	AMB	POS	NEG	NEG	NEG	NEG	NEG	NEG	NEG	POS	POS	AMB	POS	POS	NEG	POS	AMB	POS	AMB	POS	NEG	POS	POS	AMB	POS	AMB	POS	POS	POS	POS	NEG	POS	AMB	AMB	NEG	NEG	NEG	NEG
Iasi-31-P18	Carrier	POS	POS	POS	POS	POS	POS	NEG	NEG	POS	NEG	NEG	NEG	NEG	NEG	NEG	NEG	POS	POS	AMB	POS	POS	NEG	POS	NEG	POS	NEG	POS	NEG	POS	POS	AMB	POS	AMB	POS	POS	POS	POS	NEG	POS	AMB	AMB	NEG	NEG	NEG	NEG
Iasi-40-P42	Carrier	POS	POS	POS	POS	POS	POS	NEG	NEG	POS	NEG	NEG	NEG	NEG	NEG	NEG	NEG	POS	POS	AMB	POS	POS	NEG	POS	AMB	POS	NEG	POS	NEG	POS	POS	AMB	POS	AMB	POS	POS	POS	POS	NEG	POS	AMB	AMB	NEG	NEG	NEG	NEG
Iasi-40-303	BSI	POS	POS	POS	POS	POS	POS	NEG	POS	NEG	POS	NEG	POS	AMB	NEG	NEG	NEG	NEG	POS	AMB	POS	POS	NEG	POS	NEG	POS	AMB	POS	NEG	POS	POS	AMB	POS	AMB	POS	POS	POS	POS	NEG	POS	AMB	AMB	POS	NEG	NEG	NEG
Iasi-48-422	SSTI	POS	POS	POS	POS	POS	POS	NEG	POS	NEG	POS	NEG	POS	AMB	NEG	NEG	NEG	NEG	POS	AMB	POS	POS	NEG	POS	NEG	POS	AMB	POS	NEG	POS	POS	POS	AMB	NEG	POS	POS	POS	POS	NEG	POS	NEG	AMB	POS	NEG	NEG	NEG
Iasi-54-435	BSI	POS	POS	POS	POS	POS	POS	AMB	POS	AMB	POS	NEG	NEG	POS	NEG	NEG	NEG	NEG	POS	AMB	POS	POS	NEG	POS	AMB	POS	POS	AMB	NEG	POS	POS	AMB	POS	AMB	POS	POS	POS	POS	POS	AMB	AMB	POS	POS	NEG	NEG	NEG
Iasi-16-161	SSTI	POS	POS	POS	POS	POS	POS	NEG	POS	NEG	POS	NEG	POS	AMB	NEG	NEG	NEG	NEG	POS	AMB	POS	POS	NEG	POS	NEG	POS	AMB	POS	NEG	POS	POS	POS	AMB	AMB	POS	POS	POS	POS	NEG	POS	AMB	AMB	POS	NEG	NEG	NEG
Iasi-27-221	SSTI	POS	POS	POS	POS	POS	POS	NEG	POS	NEG	POS	NEG	POS	AMB	NEG	NEG	NEG	NEG	POS	NEG	POS	POS	NEG	POS	NEG	POS	AMB	POS	NEG	POS	POS	POS	AMB	NEG	POS	POS	POS	POS	NEG	POS	NEG	AMB	POS	NEG	NEG	NEG
Iasi-09-85	BSI	POS	POS	POS	POS	POS	POS	NEG	NEG	NEG	POS	NEG	POS	AMB	NEG	NEG	NEG	NEG	POS	NEG	POS	POS	NEG	POS	NEG	POS	AMB	POS	NEG	POS	POS	POS	AMB	NEG	POS	POS	POS	POS	NEG	POS	NEG	AMB	POS	NEG	NEG	NEG
Iasi-17-189	BSI	POS	POS	POS	POS	POS	POS	NEG	AMB	NEG	POS	NEG	POS	AMB	NEG	NEG	NEG	NEG	POS	NEG	POS	POS	NEG	POS	NEG	POS	AMB	POS	NEG	POS	POS	POS	AMB	NEG	POS	POS	POS	POS	NEG	POS	NEG	AMB	POS	NEG	NEG	NEG
Iasi-20-200	BSI	POS	POS	POS	POS	POS	POS	NEG	AMB	NEG	POS	NEG	POS	AMB	NEG	NEG	NEG	NEG	POS	NEG	POS	POS	NEG	POS	NEG	POS	AMB	POS	NEG	POS	POS	POS	AMB	NEG	POS	POS	POS	POS	NEG	POS	NEG	AMB	POS	NEG	NEG	NEG
Iasi-34-321	BSI	POS	POS	POS	POS	POS	POS	NEG	POS	NEG	POS	NEG	POS	AMB	NEG	NEG	NEG	NEG	POS	NEG	POS	POS	NEG	POS	NEG	POS	AMB	POS	NEG	POS	POS	POS	AMB	NEG	POS	POS	POS	POS	NEG	POS	NEG	AMB	POS	NEG	NEG	NEG
Iasi-04-57	SSTI	POS	POS	POS	POS	POS	POS	NEG	AMB	NEG	POS	NEG	POS	AMB	NEG	NEG	NEG	NEG	POS	NEG	POS	POS	NEG	POS	NEG	POS	AMB	POS	NEG	POS	POS	POS	AMB	NEG	POS	POS	POS	POS	NEG	POS	NEG	AMB	POS	NEG	NEG	NEG
Iasi-05-59	SSTI	POS	POS	POS	POS	POS	POS	NEG	POS	NEG	POS	NEG	POS	AMB	NEG	NEG	NEG	NEG	POS	NEG	POS	POS	NEG	POS	NEG	POS	AMB	POS	NEG	POS	POS	POS	AMB	NEG	POS	POS	POS	POS	NEG	POS	NEG	AMB	POS	NEG	NEG	NEG
Iasi-06-64	SSTI	POS	POS	POS	POS	POS	POS	NEG	POS	NEG	POS	NEG	POS	AMB	NEG	NEG	NEG	NEG	POS	AMB	POS	POS	NEG	POS	NEG	POS	AMB	POS	NEG	POS	POS	POS	AMB	NEG	POS	POS	POS	POS	NEG	POS	NEG	AMB	POS	NEG	NEG	NEG
Iasi-10-108	SSTI	POS	POS	POS	POS	POS	POS	NEG	AMB	NEG	POS	NEG	POS	AMB	NEG	NEG	NEG	NEG	POS	NEG	POS	POS	NEG	POS	NEG	POS	AMB	POS	NEG	POS	POS	POS	AMB	NEG	POS	POS	POS	POS	NEG	POS	NEG	AMB	POS	NEG	NEG	NEG
Iasi-12-117	SSTI	POS	POS	POS	POS	POS	POS	NEG	POS	NEG	POS	NEG	POS	AMB	NEG	NEG	NEG	NEG	POS	AMB	POS	POS	NEG	POS	NEG	POS	AMB	POS	NEG	POS	POS	POS	AMB	AMB	POS	POS	POS	POS	NEG	POS	NEG	AMB	POS	NEG	NEG	NEG
Iasi-12-138	SSTI	POS	POS	POS	POS	POS	POS	NEG	AMB	NEG	POS	NEG	POS	AMB	NEG	NEG	NEG	NEG	POS	AMB	POS	POS	NEG	POS	NEG	POS	AMB	POS	NEG	POS	POS	POS	AMB	NEG	POS	POS	POS	POS	NEG	POS	NEG	AMB	POS	NEG	NEG	NEG
Iasi-13-140	SSTI	POS	POS	POS	POS	POS	POS	NEG	AMB	NEG	POS	NEG	POS	AMB	NEG	NEG	NEG	NEG	POS	NEG	POS	POS	NEG	POS	NEG	POS	AMB	POS	NEG	POS	POS	POS	AMB	NEG	POS	POS	POS	POS	NEG	POS	NEG	AMB	POS	NEG	NEG	NEG
Iasi-15-152	SSTI	POS	POS	POS	POS	POS	POS	NEG	POS	NEG	POS	NEG	POS	AMB	NEG	NEG	NEG	NEG	POS	NEG	POS	POS	NEG	POS	NEG	POS	AMB	POS	NEG	POS	POS	POS	AMB	NEG	POS	POS	POS	POS	NEG	POS	NEG	AMB	POS	NEG	NEG	NEG
Iasi-17-168	SSTI	POS	POS	POS	POS	POS	POS	NEG	POS	NEG	POS	NEG	POS	AMB	NEG	NEG	NEG	NEG	POS	AMB	POS	POS	NEG	POS	NEG	POS	AMB	POS	NEG	POS	POS	POS	AMB	AMB	POS	POS	POS	POS	NEG	POS	AMB	AMB	POS	NEG	NEG	NEG
Iasi-20-193	SSTI	POS	POS	POS	POS	POS	POS	NEG	POS	NEG	POS	NEG	POS	AMB	NEG	NEG	NEG	NEG	POS	AMB	POS	POS	NEG	POS	NEG	POS	AMB	POS	NEG	POS	POS	POS	AMB	NEG	POS	POS	POS	POS	NEG	POS	NEG	AMB	POS	NEG	NEG	NEG
Iasi-26-218	SSTI	POS	POS	POS	POS	POS	POS	NEG	POS	NEG	POS	NEG	POS	AMB	NEG	NEG	NEG	NEG	POS	AMB	POS	POS	NEG	POS	NEG	POS	AMB	POS	NEG	POS	POS	POS	AMB	NEG	POS	POS	POS	POS	NEG	POS	NEG	AMB	POS	NEG	NEG	NEG
Iasi-28-335	SSTI	POS	POS	POS	POS	POS	POS	NEG	NEG	NEG	POS	NEG	POS	AMB	NEG	NEG	NEG	NEG	POS	NEG	POS	POS	NEG	POS	NEG	POS	NEG	POS	NEG	POS	POS	POS	AMB	NEG	POS	POS	POS	POS	NEG	POS	NEG	AMB	POS	NEG	NEG	NEG
Iasi-29-299b	SSTI	POS	POS	POS	POS	POS	POS	NEG	POS	NEG	POS	NEG	POS	AMB	NEG	NEG	NEG	NEG	POS	AMB	POS	POS	NEG	POS	NEG	POS	AMB	POS	NEG	POS	POS	POS	AMB	AMB	POS	POS	POS	POS	NEG	POS	AMB	AMB	POS	NEG	NEG	NEG
Iasi-32-364	SSTI	POS	POS	POS	POS	POS	POS	NEG	POS	NEG	POS	NEG	POS	AMB	NEG	NEG	NEG	NEG	POS	AMB	POS	POS	NEG	POS	NEG	POS	AMB	POS	NEG	POS	POS	POS	AMB	AMB	POS	POS	POS	POS	NEG	POS	NEG	AMB	POS	NEG	NEG	NEG
Iasi-369	SSTI	POS	POS	POS	POS	POS	POS	NEG	POS	NEG	POS	NEG	POS	AMB	NEG	NEG	NEG	NEG	POS	AMB	POS	POS	NEG	POS	NEG	POS	AMB	POS	NEG	POS	POS	POS	AMB	NEG	POS	POS	POS	POS	NEG	POS	AMB	AMB	POS	NEG	NEG	NEG
Iasi-03-S11	Carrier	POS	POS	POS	POS	POS	POS	NEG	POS	NEG	POS	NEG	POS	AMB	NEG	NEG	NEG	NEG	POS	AMB	POS	POS	NEG	POS	NEG	POS	AMB	POS	NEG	POS	POS	POS	AMB	AMB	POS	POS	POS	POS	NEG	POS	AMB	AMB	POS	NEG	NEG	NEG
Iasi-19-S35	Carrier	POS	POS	POS	POS	POS	POS	NEG	POS	AMB	POS	NEG	POS	AMB	NEG	NEG	NEG	NEG	POS	AMB	POS	POS	NEG	POS	NEG	POS	AMB	POS	NEG	POS	POS	AMB	POS	AMB	POS	POS	POS	POS	NEG	POS	AMB	AMB	POS	NEG	NEG	NEG
Iasi-20-P1	Carrier	POS	POS	POS	POS	POS	POS	NEG	POS	NEG	POS	NEG	POS	AMB	NEG	NEG	NEG	NEG	POS	AMB	POS	POS	NEG	POS	NEG	POS	AMB	POS	NEG	POS	POS	AMB	POS	AMB	POS	POS	POS	POS	NEG	POS	AMB	AMB	POS	NEG	NEG	NEG
Iasi-35-P24	Carrier	POS	POS	POS	POS	POS	POS	NEG	POS	NEG	POS	NEG	POS	AMB	NEG	NEG	NEG	NEG	POS	AMB	POS	POS	NEG	POS	NEG	POS	AMB	POS	NEG	NEG	NEG	AMB	POS	AMB	POS	POS	POS	POS	NEG	POS	NEG	AMB	POS	NEG	NEG	NEG
Iasi-36-P25	Carrier	POS	POS	POS	POS	POS	POS	NEG	POS	NEG	POS	NEG	POS	AMB	NEG	NEG	NEG	NEG	POS	AMB	POS	POS	NEG	POS	NEG	POS	AMB	POS	NEG	POS	POS	AMB	POS	AMB	POS	POS	POS	POS	NEG	POS	AMB	AMB	POS	NEG	NEG	NEG
Iasi-14-150	SSTI	POS	POS	POS	POS	POS	POS	NEG	POS	NEG	POS	NEG	POS	AMB	NEG	NEG	NEG	NEG	POS	AMB	POS	POS	NEG	POS	NEG	POS	AMB	POS	NEG	POS	POS	POS	AMB	NEG	POS	POS	POS	POS	NEG	POS	NEG	AMB	POS	NEG	NEG	NEG
Iasi-07-54	BSI	POS	POS	POS	POS	POS	POS	NEG	POS	NEG	POS	NEG	POS	AMB	NEG	NEG	NEG	NEG	POS	AMB	POS	POS	NEG	POS	NEG	POS	AMB	POS	NEG	POS	POS	AMB	POS	AMB	POS	POS	POS	POS	NEG	POS	AMB	AMB	POS	NEG	NEG	NEG
Iasi-16-188	BSI	POS	POS	POS	POS	POS	POS	NEG	POS	NEG	POS	NEG	POS	AMB	NEG	NEG	NEG	NEG	POS	AMB	POS	POS	NEG	POS	NEG	POS	AMB	POS	NEG	POS	POS	POS	AMB	AMB	POS	POS	POS	POS	NEG	POS	AMB	AMB	POS	NEG	NEG	NEG
Iasi-32-318	BSI	POS	POS	POS	POS	POS	POS	NEG	AMB	NEG	POS	NEG	POS	AMB	NEG	NEG	NEG	NEG	POS	NEG	POS	POS	NEG	POS	NEG	POS	AMB	POS	NEG	POS	POS	POS	AMB	NEG	POS	POS	POS	POS	NEG	POS	NEG	AMB	POS	NEG	NEG	NEG
Iasi-42-397	BSI	POS	POS	POS	POS	POS	POS	NEG	AMB	NEG	POS	NEG	POS	AMB	NEG	NEG	NEG	NEG	POS	NEG	POS	POS	NEG	POS	NEG	POS	AMB	POS	NEG	POS	POS	POS	AMB	NEG	POS	POS	POS	POS	NEG	POS	NEG	AMB	POS	NEG	NEG	NEG
Iasi-48-408	BSI	POS	POS	POS	POS	POS	POS	NEG	AMB	NEG	POS	NEG	POS	AMB	NEG	NEG	NEG	NEG	POS	AMB	POS	POS	NEG	POS	NEG	POS	AMB	POS	NEG	POS	POS	POS	AMB	AMB	POS	POS	POS	POS	NEG	POS	NEG	AMB	POS	NEG	NEG	NEG
Iasi-49-430	BSI	POS	POS	POS	POS	POS	POS	NEG	POS	NEG	POS	NEG	POS	AMB	NEG	NEG	NEG	NEG	POS	AMB	POS	POS	NEG	POS	NEG	POS	POS	AMB	NEG	POS	POS	AMB	POS	AMB	POS	POS	POS	POS	NEG	POS	AMB	AMB	POS	NEG	NEG	NEG
Iasi-52-52	BSI	POS	POS	POS	POS	POS	POS	NEG	AMB	NEG	POS	NEG	POS	AMB	NEG	NEG	NEG	NEG	POS	AMB	POS	POS	NEG	POS	NEG	POS	AMB	POS	NEG	POS	POS	POS	AMB	NEG	POS	POS	POS	POS	NEG	POS	NEG	AMB	POS	NEG	NEG	NEG
Iasi-01-2	SSTI	POS	POS	POS	POS	POS	POS	NEG	POS	NEG	POS	NEG	POS	AMB	NEG	NEG	NEG	NEG	POS	AMB	POS	POS	NEG	POS	NEG	POS	AMB	POS	NEG	POS	POS	POS	AMB	AMB	POS	POS	POS	POS	NEG	POS	AMB	AMB	POS	NEG	NEG	NEG
Iasi-10-101	SSTI	POS	POS	POS	POS	POS	POS	NEG	AMB	NEG	POS	NEG	POS	AMB	NEG	NEG	NEG	NEG	POS	AMB	POS	POS	NEG	POS	NEG	POS	AMB	POS	NEG	POS	POS	POS	AMB	NEG	POS	POS	POS	POS	NEG	POS	NEG	AMB	POS	NEG	NEG	NEG
Iasi-11-106	SSTI	POS	POS	POS	POS	POS	POS	NEG	POS	NEG	POS	NEG	POS	AMB	NEG	NEG	NEG	NEG	POS	NEG	POS	POS	NEG	POS	NEG	POS	AMB	POS	NEG	POS	POS	POS	AMB	NEG	POS	POS	POS	POS	NEG	POS	NEG	AMB	POS	NEG	NEG	NEG
Iasi-49-414	SSTI	POS	POS	POS	POS	POS	POS	NEG	POS	NEG	POS	NEG	POS	AMB	NEG	NEG	NEG	NEG	POS	AMB	POS	POS	NEG	POS	NEG	POS	AMB	POS	NEG	POS	POS	AMB	POS	AMB	POS	POS	POS	POS	NEG	POS	AMB	AMB	POS	NEG	NEG	NEG

Isolate	Group	VIRULENCE : PROTEASES				VIRULENCE : STAPHYLOCOCCAL SUPERANTIGEN/ENTEROTOXIN-LIKE GENES (SET/SSL)																																								
		glutamylendopeptidase	staphopain B, protease	staphopain A (staphylopain A), protease		staphyl. exotoxin-like protein	staphylococcal superantigen-like protein 1												staphylococcal superantigen-like protein 2		staphylococcal superantigen-like protein 3			staphylococcal superantigen-like protein 4		staphylococcal superantigen-like protein 5				staphylococcal superantigen-like protein 6		staphylococcal superantigen-like protein 7			staphylococcal superantigen-like protein 8		staphylococcal superantigen-like protein 9			staphylococcal superantigen-like protein 10			staphylococcal superantigene-like protein 11			
		sspA	sspB	sspP (consensus)	sspP (other than ST93)	setC	ssl01/set6_probe1_11	ssl01/set6_probe2_11	ssl01/set6_probe1_12	ssl01/set6_probe2_12	ssl01/set6_probe4_11	ssl01/set6_probeRF122	ssl01/set6 (COL)	ssl01/set6 (Mu50+N315)	ssl01/set6 (MW2+MSSA476)	ssl01/set6 (MRSA252)	ssl01/set6 (RF122)	ssl01/set6 (other alleles)	ssl02/set7	ssl02/set7 (MRSA252)	ssl03/set8_probe 1	ssl03/set8_probe 2	ssl03/set8 (MRSA252, SAR0424)	ssl04/set9	ssl04/set9 (MRSA252, SAR0425)	ssl05/set3_probe 1	ssl05/set3 (RF122, probe-611)	ssl05/set3_probe 2 (612)	ssl05/set3 (MRSA252)	ssl06/set21	ssl06 (NCTC8325+MW2)	ssl07/set1	ssl07/set1 (MRSA252)	ssl07/set1 (AF188836)	ssl08/set12_probe 1	ssl08/set12_probe 2	ssl09/set5_probe 1	ssl09/set5_probe 2	ssl09/set5 (MRSA252)	ssl10/set4	ssl10 (RF122)	ssl10/set4 (MRSA252)	ssl11/set2 (COL)	ssl11+set2(Mu50+N315)	ssl11+set2(MW2+MSSA476)	ssl11/set2 (MRSA252)
Iasi-05-46	misc. MRSA	POS	POS	POS	POS	POS	POS	NEG	AMB	NEG	POS	NEG	POS	AMB	NEG	NEG	NEG	NEG	POS	AMB	POS	POS	NEG	POS	NEG	POS	AMB	POS	NEG	POS	POS	POS	AMB	NEG	POS	POS	POS	POS	NEG	POS	NEG	AMB	POS	NEG	NEG	NEG
Iasi-06-47	misc. MRSA	POS	POS	POS	POS	POS	POS	NEG	POS	NEG	POS	NEG	POS	AMB	NEG	NEG	NEG	NEG	POS	AMB	POS	POS	NEG	POS	NEG	POS	AMB	POS	NEG	POS	POS	POS	AMB	AMB	POS	POS	POS	POS	NEG	POS	AMB	AMB	POS	NEG	NEG	NEG
Iasi-08-73	misc. MRSA	POS	POS	POS	POS	POS	POS	NEG	POS	NEG	POS	NEG	POS	AMB	NEG	NEG	NEG	NEG	POS	AMB	POS	POS	NEG	POS	NEG	POS	AMB	POS	NEG	POS	POS	POS	AMB	AMB	POS	POS	POS	POS	NEG	POS	AMB	AMB	POS	NEG	NEG	NEG
Iasi-19-194	misc. MRSA	POS	POS	POS	POS	POS	POS	NEG	AMB	NEG	POS	NEG	POS	AMB	NEG	NEG	NEG	NEG	POS	AMB	POS	POS	NEG	POS	NEG	POS	AMB	POS	NEG	POS	POS	POS	AMB	NEG	POS	POS	POS	POS	NEG	POS	NEG	AMB	POS	NEG	NEG	NEG
Iasi-21-219	misc. MRSA	POS	POS	POS	POS	POS	POS	NEG	POS	NEG	POS	NEG	POS	AMB	NEG	NEG	NEG	NEG	POS	AMB	POS	POS	NEG	POS	NEG	POS	AMB	POS	NEG	POS	POS	POS	AMB	AMB	POS	POS	POS	POS	NEG	POS	NEG	AMB	POS	NEG	NEG	NEG
Iasi-25-284	misc. MRSA	POS	POS	POS	POS	POS	POS	NEG	POS	NEG	POS	NEG	POS	AMB	NEG	NEG	NEG	NEG	POS	AMB	POS	POS	NEG	POS	NEG	POS	AMB	POS	NEG	POS	POS	POS	AMB	NEG	POS	POS	POS	POS	NEG	POS	NEG	AMB	POS	NEG	NEG	NEG
Iasi-284	misc. MRSA	POS	POS	POS	POS	POS	POS	NEG	AMB	NEG	POS	NEG	POS	AMB	NEG	NEG	NEG	NEG	POS	AMB	POS	POS	NEG	POS	NEG	POS	AMB	POS	NEG	POS	POS	POS	AMB	NEG	POS	POS	POS	POS	NEG	POS	AMB	AMB	POS	NEG	NEG	NEG
Iasi-36-336	misc. MRSA	POS	POS	POS	POS	POS	POS	NEG	AMB	NEG	POS	NEG	POS	AMB	NEG	NEG	NEG	NEG	POS	NEG	POS	POS	NEG	POS	NEG	POS	AMB	POS	NEG	POS	POS	POS	AMB	NEG	POS	POS	POS	POS	NEG	POS	NEG	AMB	POS	NEG	NEG	NEG
Iasi-43-402	misc. MRSA	POS	POS	POS	POS	POS	POS	NEG	POS	NEG	POS	NEG	POS	AMB	NEG	NEG	NEG	NEG	POS	AMB	POS	POS	NEG	POS	NEG	POS	AMB	POS	NEG	POS	POS	POS	AMB	AMB	POS	POS	POS	POS	NEG	POS	AMB	AMB	POS	NEG	NEG	NEG
Iasi-44-404	misc. MRSA	POS	POS	POS	POS	POS	POS	NEG	AMB	NEG	POS	NEG	POS	AMB	NEG	NEG	NEG	NEG	POS	NEG	POS	POS	NEG	POS	NEG	POS	AMB	POS	NEG	POS	POS	POS	AMB	NEG	POS	POS	POS	POS	NEG	POS	NEG	AMB	POS	NEG	NEG	NEG
Iasi-54-430	misc. MRSA	POS	POS	POS	POS	POS	POS	NEG	POS	NEG	POS	NEG	POS	AMB	NEG	NEG	NEG	NEG	POS	AMB	POS	POS	NEG	POS	NEG	POS	AMB	POS	NEG	POS	POS	AMB	POS	AMB	POS	POS	POS	POS	NEG	POS	AMB	AMB	POS	NEG	NEG	NEG
Iasi-24-216	SSTI	POS	POS	POS	POS	POS	POS	NEG	POS	NEG	POS	NEG	NEG	POS	NEG	NEG	NEG	NEG	AMB	POS	NEG	NEG	NEG	NEG	AMB	NEG	NEG	NEG	POS	NEG	NEG	NEG	POS	AMB	NEG	NEG	NEG	NEG	POS	POS	NEG	AMB	NEG	NEG	NEG	NEG
Iasi-05-S13	Carrier	POS	POS	POS	POS	POS	POS	NEG	POS	NEG	POS	NEG	POS	AMB	NEG	NEG	NEG	NEG	AMB	POS	NEG	NEG	NEG	NEG	POS	NEG	NEG	NEG	POS	NEG	AMB	AMB	POS	AMB	NEG	NEG	NEG	NEG	POS	POS	NEG	AMB	NEG	NEG	NEG	NEG
Iasi-45-362	BSI	POS	POS	POS	POS	POS	NEG	AMB	NEG	NEG	POS	NEG	NEG	NEG	NEG	NEG	NEG	POS	POS	AMB	POS	POS	NEG	POS	NEG	POS	AMB	POS	NEG	POS	POS	POS	AMB	NEG	POS	POS	POS	POS	NEG	POS	AMB	NEG	NEG	NEG	NEG	POS
Iasi-22-199	SSTI	POS	POS	POS	POS	POS	NEG	AMB	NEG	NEG	POS	NEG	NEG	NEG	NEG	NEG	NEG	POS	POS	AMB	POS	POS	NEG	POS	NEG	POS	POS	AMB	NEG	POS	POS	POS	AMB	NEG	POS	POS	POS	POS	NEG	POS	AMB	NEG	NEG	NEG	NEG	POS
Iasi-12-S26	Carrier	POS	POS	POS	POS	POS	NEG	POS	NEG	NEG	POS	NEG	NEG	NEG	NEG	NEG	NEG	POS	POS	AMB	POS	POS	NEG	POS	AMB	POS	AMB	POS	NEG	POS	POS	AMB	POS	AMB	POS	POS	POS	POS	NEG	POS	AMB	AMB	NEG	NEG	NEG	POS
Iasi-21-P2	Carrier	POS	POS	POS	POS	POS	NEG	POS	NEG	NEG	POS	NEG	NEG	NEG	NEG	NEG	NEG	POS	POS	AMB	POS	POS	NEG	POS	AMB	POS	AMB	POS	NEG	POS	POS	POS	AMB	AMB	POS	POS	POS	POS	NEG	AMB	POS	AMB	NEG	NEG	NEG	POS
Iasi-06-60	BSI	POS	POS	POS	POS	POS	NEG	POS	POS	NEG	NEG	NEG	NEG	NEG	NEG	POS	NEG	NEG	AMB	POS	NEG	NEG	AMB	NEG	POS	POS	POS	NEG	POS	NEG	AMB	AMB	POS	AMB	NEG	NEG	POS	POS	NEG	POS	NEG	AMB	NEG	NEG	NEG	NEG
Iasi-12-79	BSI	POS	POS	POS	POS	POS	NEG	POS	POS	NEG	NEG	NEG	NEG	NEG	NEG	POS	NEG	NEG	AMB	POS	NEG	NEG	NEG	NEG	AMB	POS	NEG	NEG	POS	NEG	NEG	AMB	POS	AMB	NEG	NEG	POS	POS	NEG	POS	NEG	NEG	NEG	NEG	NEG	NEG
Iasi-17-90	BSI	POS	POS	POS	POS	POS	NEG	POS	POS	NEG	NEG	NEG	NEG	NEG	NEG	POS	NEG	NEG	AMB	POS	NEG	NEG	NEG	NEG	AMB	POS	POS	NEG	POS	NEG	AMB	AMB	POS	AMB	NEG	NEG	POS	POS	NEG	POS	NEG	NEG	NEG	NEG	NEG	NEG
Iasi-21-105	BSI	POS	POS	POS	POS	POS	NEG	POS	POS	NEG	NEG	NEG	NEG	NEG	NEG	POS	NEG	NEG	POS	AMB	NEG	NEG	NEG	NEG	AMB	POS	NEG	NEG	POS	NEG	NEG	AMB	POS	AMB	NEG	NEG	POS	POS	NEG	POS	NEG	NEG	NEG	NEG	NEG	NEG
Iasi-22-109	BSI	POS	POS	POS	POS	POS	NEG	POS	POS	NEG	NEG	NEG	NEG	NEG	NEG	POS	NEG	NEG	AMB	POS	AMB	NEG	POS	NEG	POS	POS	POS	NEG	POS	NEG	POS	POS	AMB	AMB	NEG	NEG	POS	POS	AMB	POS	NEG	AMB	NEG	NEG	NEG	NEG
Iasi-23-110	BSI	POS	POS	POS	POS	POS	NEG	POS	POS	NEG	NEG	NEG	NEG	NEG	NEG	POS	NEG	NEG	POS	AMB	NEG	NEG	AMB	NEG	POS	POS	POS	NEG	POS	NEG	AMB	AMB	POS	AMB	NEG	NEG	POS	POS	NEG	POS	NEG	AMB	NEG	NEG	NEG	NEG
Iasi-24-136	BSI	POS	POS	POS	POS	POS	NEG	POS	POS	NEG	NEG	NEG	NEG	NEG	NEG	POS	NEG	NEG	AMB	POS	NEG	NEG	AMB	NEG	POS	POS	POS	NEG	POS	NEG	AMB	AMB	POS	AMB	NEG	NEG	POS	POS	NEG	POS	NEG	AMB	NEG	NEG	NEG	NEG
Iasi-34-224	BSI	POS	POS	POS	POS	POS	NEG	POS	POS	NEG	NEG	NEG	NEG	NEG	NEG	POS	NEG	NEG	AMB	POS	AMB	NEG	POS	NEG	POS	POS	POS	NEG	POS	NEG	AMB	AMB	POS	AMB	NEG	NEG	POS	POS	NEG	POS	NEG	NEG	NEG	NEG	NEG	NEG
Iasi-39-297	BSI	POS	POS	POS	POS	POS	NEG	POS	POS	NEG	NEG	NEG	NEG	NEG	NEG	POS	NEG	NEG	POS	AMB	AMB	NEG	POS	NEG	POS	POS	POS	NEG	POS	NEG	POS	AMB	POS	AMB	NEG	NEG	POS	POS	NEG	POS	NEG	AMB	NEG	NEG	NEG	NEG
Iasi-50-431	BSI	POS	POS	POS	POS	POS	NEG	POS	POS	NEG	NEG	NEG	NEG	NEG	NEG	POS	NEG	NEG	AMB	POS	NEG	NEG	AMB	NEG	POS	POS	POS	NEG	POS	NEG	AMB	AMB	POS	AMB	NEG	NEG	POS	POS	NEG	POS	NEG	AMB	NEG	NEG	NEG	NEG
Iasi-51-432	BSI	POS	POS	POS	POS	POS	NEG	POS	POS	NEG	NEG	NEG	NEG	NEG	NEG	POS	NEG	NEG	AMB	POS	AMB	NEG	POS	NEG	POS	POS	POS	NEG	POS	NEG	POS	AMB	POS	AMB	NEG	NEG	POS	POS	NEG	POS	NEG	AMB	NEG	NEG	NEG	NEG
Iasi-08-76	SSTI	POS	POS	POS	POS	POS	NEG	POS	POS	NEG	NEG	NEG	NEG	NEG	NEG	POS	NEG	NEG	AMB	POS	NEG	NEG	AMB	NEG	AMB	POS	NEG	NEG	POS	NEG	NEG	AMB	AMB	POS	NEG	NEG	POS	POS	NEG	POS	NEG	NEG	NEG	NEG	NEG	NEG
Iasi-30-350	SSTI	POS	POS	POS	POS	POS	NEG	POS	POS	NEG	NEG	NEG	NEG	NEG	NEG	POS	NEG	NEG	AMB	POS	NEG	NEG	AMB	NEG	POS	POS	NEG	NEG	POS	NEG	AMB	AMB	AMB	POS	NEG	NEG	POS	POS	NEG	POS	NEG	AMB	NEG	NEG	NEG	NEG
Iasi-33-319	SSTI	POS	POS	POS	POS	POS	NEG	POS	AMB	NEG	NEG	NEG	NEG	NEG	NEG	NEG	NEG	POS	AMB	POS	NEG	NEG	NEG	NEG	AMB	POS	NEG	NEG	POS	NEG	NEG	AMB	AMB	POS	NEG	NEG	AMB	AMB	NEG	NEG	NEG	NEG	NEG	NEG	NEG	NEG
Iasi-40-383	SSTI	POS	POS	POS	POS	POS	NEG	POS	POS	NEG	NEG	NEG	NEG	NEG	NEG	POS	NEG	NEG	AMB	POS	NEG	NEG	POS	NEG	POS	POS	NEG	NEG	POS	NEG	NEG	AMB	POS	AMB	NEG	NEG	POS	POS	NEG	POS	NEG	AMB	NEG	NEG	NEG	NEG
Iasi-49-423	SSTI	POS	POS	POS	POS	POS	NEG	POS	POS	NEG	NEG	NEG	NEG	NEG	NEG	POS	NEG	NEG	AMB	POS	NEG	NEG	POS	NEG	POS	POS	NEG	NEG	POS	NEG	NEG	AMB	AMB	POS	NEG	NEG	POS	POS	NEG	POS	NEG	AMB	NEG	NEG	NEG	NEG
Iasi-16-S31	Carrier	POS	POS	POS	POS	POS	NEG	POS	POS	NEG	NEG	NEG	NEG	NEG	NEG	POS	NEG	NEG	AMB	POS	NEG	NEG	POS	NEG	POS	POS	POS	NEG	POS	NEG	AMB	AMB	POS	AMB	NEG	NEG	POS	POS	NEG	POS	NEG	AMB	NEG	NEG	NEG	NEG
Iasi-24-P9	Carrier	POS	POS	POS	POS	POS	NEG	POS	POS	NEG	NEG	NEG	NEG	NEG	NEG	POS	NEG	NEG	AMB	POS	NEG	NEG	POS	NEG	POS	POS	POS	NEG	POS	NEG	AMB	AMB	POS	AMB	NEG	NEG	POS	POS	NEG	POS	NEG	AMB	NEG	NEG	NEG	NEG
Iasi-27-P12	Carrier	POS	POS	POS	POS	POS	NEG	POS	POS	NEG	NEG	NEG	NEG	NEG	NEG	POS	NEG	NEG	AMB	POS	NEG	NEG	POS	NEG	POS	POS	NEG	NEG	POS	NEG	NEG	AMB	POS	AMB	NEG	NEG	POS	POS	NEG	POS	NEG	AMB	NEG	NEG	NEG	NEG
Iasi-38-P27	Carrier	POS	POS	POS	POS	POS	NEG	POS	POS	NEG	NEG	NEG	NEG	NEG	NEG	POS	NEG	NEG	POS	AMB	NEG	NEG	POS	NEG	POS	POS	POS	NEG	POS	NEG	AMB	AMB	POS	AMB	NEG	NEG	POS	POS	NEG	POS	NEG	AMB	NEG	NEG	NEG	NEG
Iasi-42-G511	Carrier	POS	POS	POS	POS	POS	NEG	POS	POS	NEG	NEG	NEG	NEG	NEG	NEG	POS	NEG	NEG	AMB	POS	NEG	NEG	POS	NEG	POS	POS	POS	NEG	POS	NEG	NEG	AMB	POS	AMB	NEG	NEG	POS	POS	NEG	POS	NEG	AMB	NEG	NEG	NEG	NEG
Iasi-46-3N	Carrier	POS	POS	POS	POS	POS	NEG	POS	POS	NEG	NEG	NEG	NEG	NEG	NEG	POS	NEG	NEG	AMB	POS	AMB	NEG	POS	NEG	POS	POS	POS	NEG	POS	NEG	AMB	AMB	POS	AMB	NEG	NEG	POS	POS	NEG	POS	NEG	AMB	NEG	NEG	NEG	NEG
Iasi-47-42O	Carrier	POS	POS	POS	POS	POS	NEG	POS	POS	NEG	NEG	NEG	NEG	NEG	NEG	POS	NEG	NEG	AMB	POS	NEG	NEG	POS	NEG	POS	POS	POS	NEG	POS	NEG	AMB	AMB	POS	AMB	NEG	NEG	POS	POS	NEG	POS	NEG	AMB	NEG	NEG	NEG	NEG
Iasi-48-36N	Carrier	POS	POS	POS	POS	POS	NEG	POS	POS	NEG	NEG	NEG	NEG	NEG	NEG	POS	NEG	NEG	POS	AMB	NEG	NEG	AMB	NEG	POS	POS	POS	NEG	POS	NEG	AMB	AMB	POS	AMB	NEG	NEG	POS	POS	NEG	POS	NEG	AMB	NEG	NEG	NEG	NEG
Iasi-49-T3	Carrier	POS	POS	POS	POS	POS	NEG	POS	POS	NEG	NEG	NEG	NEG	NEG	NEG	POS	NEG	NEG	POS	AMB	NEG	NEG	AMB	NEG	POS	POS	POS	NEG	POS	NEG	NEG	AMB	POS	AMB	NEG	NEG	POS	POS	NEG	POS	NEG	AMB	NEG	NEG	NEG	NEG
Iasi-50-T2	Carrier	POS	POS	POS	POS	POS	NEG	POS	POS	NEG	NEG	NEG	NEG	NEG	NEG	POS	NEG	NEG	POS	AMB	NEG	NEG	POS	NEG	POS	POS	POS	NEG	POS	NEG	AMB	AMB	POS	AMB	NEG	NEG	POS	POS	NEG	POS	NEG	AMB	NEG	NEG	NEG	NEG
Iasi-22-251	BSI	POS	POS	POS	POS	POS	NEG	POS	POS	NEG	NEG	NEG	NEG	NEG	NEG	POS	NEG	NEG	POS	AMB	NEG	NEG	AMB	NEG	POS	POS	POS	NEG	POS	NEG	AMB	AMB	POS	AMB	NEG	NEG	POS	POS	NEG	POS	NEG	AMB	NEG	NEG	NEG	NEG
Iasi-27-158	BSI	POS	POS	POS	POS	POS	NEG	POS	POS	NEG	POS	POS	NEG	NEG	NEG	POS	NEG	NEG	POS	AMB	POS	POS	NEG	POS	POS	POS	AMB	POS	NEG	NEG	AMB	POS	AMB	AMB	POS	POS	POS	POS	POS	POS	AMB	AMB	NEG	NEG	NEG	NEG
Iasi-08-70	BSI	POS	POS	POS	POS	NEG	NEG	POS	POS	NEG	POS	NEG	NEG	NEG	NEG	POS	NEG	NEG	AMB	POS	NEG	AMB	POS	NEG	POS	NEG	POS	NEG	POS	NEG	POS	AMB	POS	AMB	NEG	NEG	NEG	NEG	POS	POS	AMB	AMB	NEG	NEG	NEG	POS
Iasi-18-93	BSI	POS	POS	POS	POS	NEG	NEG	POS	POS	NEG	POS	NEG	NEG	NEG	NEG	POS	NEG	NEG	AMB	POS	NEG	AMB	POS	NEG	POS	NEG	POS	NEG	POS	NEG	POS	AMB	POS	AMB	NEG	NEG	NEG	NEG	POS	POS	NEG	AMB	NEG	NEG	NEG	POS
Iasi-28-P14	Carrier	POS	POS	POS	POS	NEG	NEG	POS	POS	NEG	POS	NEG	NEG	NEG	NEG	POS	NEG	NEG	NEG	POS	NEG	NEG	POS	NEG	POS	NEG	NEG	NEG	POS	NEG	NEG	NEG	POS	NEG	NEG	NEG	NEG	NEG	POS	AMB	NEG	POS	NEG	NEG	NEG	POS
Iasi-29-P15	Carrier	POS	POS	POS	POS	NEG	NEG	POS	POS	AMB	POS	NEG	NEG	NEG	NEG	POS	NEG	NEG	AMB	POS	AMB	POS	POS	AMB	POS	NEG	POS	NEG	POS	NEG	POS	AMB	POS	AMB	NEG	NEG	NEG	NEG	POS	POS	NEG	AMB	NEG	NEG	NEG	POS
Iasi-03-19	BSI	POS	POS	POS	POS	AMB	NEG	POS	NEG	NEG	AMB	NEG	NEG	NEG	NEG	NEG	NEG	POS	NEG	POS	NEG	NEG	NEG	NEG	AMB	NEG	NEG	NEG	POS	NEG	AMB	NEG	NEG	POS	NEG	NEG	NEG	NEG	POS	POS	NEG	AMB	NEG	NEG	NEG	NEG
Iasi-11-77	BSI	POS	POS	POS	POS	AMB	NEG	POS	AMB	NEG	AMB	NEG	NEG	NEG	NEG	NEG	NEG	POS	NEG	POS	NEG	NEG	NEG	NEG	AMB	NEG	NEG	NEG	POS	NEG	AMB	NEG	NEG	POS	NEG	NEG	NEG	NEG	POS	POS	NEG	AMB	NEG	NEG	NEG	NEG
Iasi-13-82	BSI	POS	POS	POS	POS	AMB	NEG	POS	AMB	NEG	AMB	NEG	NEG	NEG	NEG	NEG	NEG	POS	NEG	POS	NEG	NEG	NEG	NEG	AMB	NEG	NEG	NEG	POS	NEG	AMB	NEG	NEG	POS	NEG	NEG	NEG	NEG	POS	POS	NEG	AMB	NEG	NEG	NEG	NEG
Iasi-15-86	BSI	POS	POS	POS	POS	POS	NEG	POS	POS	NEG	POS	NEG	NEG	NEG	NEG	POS	NEG	NEG	AMB	POS	NEG	NEG	NEG	NEG	POS	NEG	NEG	NEG	POS	NEG	POS	NEG	AMB	POS	NEG	NEG	NEG	NEG	POS	POS	AMB	AMB	NEG	NEG	NEG	NEG
Iasi-20-103	BSI	POS	POS	POS	POS	AMB	NEG	POS	AMB	NEG	POS	NEG	NEG	NEG	NEG	NEG	NEG	POS	NEG	POS	NEG	NEG	NEG	NEG	AMB	NEG	NEG	NEG	POS	NEG	AMB	NEG	NEG	POS	NEG	NEG	NEG	NEG	POS	POS	NEG	AMB	NEG	NEG	NEG	NEG
Iasi-33-222	BSI	POS	POS	POS	POS	POS	NEG	POS	POS	NEG	POS	NEG	NEG	NEG	NEG	POS	NEG	NEG	AMB	POS	NEG	NEG	NEG	NEG	POS	NEG	POS	NEG	POS	NEG	POS	NEG	AMB	POS	NEG	NEG	NEG	NEG	POS	POS	NEG	AMB	NEG	NEG	NEG	NEG
Iasi-44-360	BSI	POS	POS	POS	POS	POS	NEG	POS	NEG	NEG	POS	NEG	NEG	NEG	NEG	NEG	NEG	POS	NEG	POS	NEG	NEG	NEG	NEG	POS	NEG	NEG	NEG	POS	NEG	AMB	NEG	NEG	POS	NEG	NEG	NEG	NEG	POS	POS	NEG	AMB	NEG	NEG	NEG	NEG
Iasi-47-399	BSI	POS	POS	POS	POS	POS	NEG	POS	POS	NEG	POS	NEG	NEG	NEG	NEG	POS	NEG	NEG	AMB	POS	NEG	NEG	NEG	NEG	POS	NEG	NEG	NEG	POS	NEG	POS	NEG	AMB	POS	NEG	NEG	NEG	NEG	POS	POS	AMB	AMB	NEG	NEG	NEG	NEG
Iasi-34-368	SSTI	POS	POS	POS	POS	POS	NEG	POS	POS	NEG	POS	NEG	NEG	NEG	NEG	POS	NEG	NEG	NEG	POS	NEG	NEG	NEG	NEG	POS	NEG	NEG	NEG	POS	NEG	AMB	NEG	NEG	POS	NEG	NEG	NEG	NEG	POS	POS	NEG	AMB	NEG	NEG	NEG	NEG
Iasi-36-371	SSTI	POS	POS	POS	POS	POS	NEG	POS	AMB	NEG	POS	NEG	NEG	NEG	NEG	NEG	NEG	POS	NEG	POS	NEG	NEG	NEG	NEG	POS	NEG	NEG	NEG	POS	NEG	AMB	NEG	NEG	POS	NEG	NEG	NEG	NEG	POS	POS	NEG	AMB	NEG	NEG	NEG	NEG
Iasi-04-S12	Carrier	POS	POS	POS	POS	POS	NEG	POS	POS	NEG	POS	NEG	NEG	NEG	NEG	POS	NEG	NEG	AMB	POS	NEG	NEG	NEG	NEG	POS	NEG	NEG	NEG	POS	NEG	POS	NEG	AMB	POS	NEG	NEG	NEG	NEG	POS	POS	NEG	AMB	NEG	NEG	NEG	NEG
Iasi-06-S15	Carrier	POS	POS	POS	POS	POS	NEG	POS	POS	NEG	POS	NEG	NEG	NEG	NEG	POS	NEG	NEG	NEG	POS	NEG	NEG	NEG	NEG	POS	NEG	NEG	NEG	POS	NEG	POS	NEG	NEG	POS	NEG	NEG	NEG	NEG	POS	POS	NEG	AMB	NEG	NEG	NEG	NEG
Iasi-07-S16	Carrier	POS	POS	POS	POS	POS	NEG	POS	POS	NEG	POS	NEG	NEG	NEG	NEG	POS	NEG	NEG	AMB	POS	NEG	NEG	NEG	NEG	POS	NEG	NEG	NEG	POS	NEG	AMB	NEG	NEG	POS	NEG	NEG	NEG	NEG	POS	POS	NEG	AMB	NEG	NEG	NEG	NEG
Iasi-11-S24	Carrier	POS	POS	POS	POS	POS	NEG	POS	POS	NEG	POS	NEG	NEG	NEG	NEG	POS	NEG	NEG	AMB	POS	NEG	NEG	NEG	NEG	POS	NEG	NEG	NEG	POS	NEG	POS	NEG	AMB	POS	NEG	NEG	NEG	NEG	POS	POS	NEG	AMB	NEG	NEG	NEG	NEG
Iasi-22-P4	Carrier	POS	POS	POS	POS	POS	NEG	POS	POS	NEG	POS	NEG	NEG	NEG	NEG	POS	NEG	NEG	AMB	POS	NEG	NEG	NEG	NEG	POS	NEG	NEG	NEG	POS	NEG	POS	NEG	NEG	POS	NEG	NEG	NEG	NEG	POS	POS	NEG	AMB	NEG	NEG	NEG	NEG
Iasi-34-P23	Carrier	POS	POS	POS	POS	POS	NEG	POS	POS	NEG	POS	NEG	NEG	NEG	NEG	POS	NEG	NEG	AMB	POS	NEG	NEG	NEG	NEG	POS	NEG	NEG	NEG	POS	NEG	POS	NEG	AMB	POS	NEG	NEG	NEG	NEG	POS	POS	NEG	AMB	NEG	NEG	NEG	NEG
Iasi-37-P26	Carrier	POS	POS	POS	POS	POS	NEG	POS	POS	NEG	POS	NEG	NEG	NEG	NEG	POS	NEG	NEG	AMB	POS	AMB	NEG	NEG	NEG	POS	NEG	NEG	NEG	POS	NEG	POS	NEG	AMB	POS	NEG	NEG	NEG	NEG	POS	AMB	NEG	POS	NEG	NEG	NEG	NEG
Iasi-30-196	BSI	POS	POS	POS	POS	POS	NEG	POS	POS	NEG	POS	NEG	NEG	NEG	NEG	POS	NEG	NEG	AMB	POS	AMB	NEG	NEG	NEG	POS	NEG	NEG	NEG	POS	NEG	POS	NEG	AMB	POS	AMB	NEG	NEG	NEG	POS	POS	NEG	AMB	NEG	NEG	NEG	NEG
Iasi-27-298	BSI	POS	POS	POS	POS	POS	NEG	NEG	NEG	NEG	POS	POS	NEG	NEG	NEG	NEG	NEG	POS	POS	AMB	POS	POS	NEG	POS	NEG	POS	POS	AMB	NEG	NEG	NEG	POS	AMB	NEG	POS	POS	POS	POS	NEG	POS	NEG	AMB	NEG	NEG	NEG	NEG
Iasi-37-363	BSI	POS	POS	POS	POS	POS	NEG	NEG	AMB	NEG	POS	POS	NEG	NEG	NEG	NEG	NEG	POS	POS	AMB	POS	POS	NEG	POS	NEG	POS	AMB	POS	NEG	NEG	NEG	POS	AMB	NEG	POS	POS	POS	POS	NEG	POS	NEG	AMB	NEG	NEG	NEG	NEG
Iasi-38-372	BSI	POS	POS	POS	POS	POS	NEG	NEG	AMB	NEG	POS	POS	NEG	NEG	NEG	NEG	NEG	POS	POS	AMB	POS	POS	NEG	POS	NEG	POS	AMB	POS	NEG	NEG	NEG	POS	AMB	NEG	POS	POS	POS	POS	NEG	POS	AMB	AMB	NEG	NEG	NEG	NEG
Iasi-39-374	BSI	POS	POS	POS	POS	POS	NEG	NEG	POS	NEG	POS	POS	NEG	NEG	NEG	NEG	NEG	POS	POS	AMB	POS	POS	NEG	POS	AMB	POS	AMB	POS	NEG	NEG	NEG	POS	AMB	AMB	POS	POS	POS	POS	AMB	POS	AMB	AMB	NEG	NEG	NEG	NEG
Iasi-28-299	SSTI	POS	POS	POS	POS	POS	NEG	NEG	AMB	NEG	POS	POS	NEG	NEG	NEG	NEG	NEG	POS	POS	AMB	POS	POS	NEG	POS	NEG	POS	AMB	POS	NEG	NEG	NEG	POS	AMB	NEG	POS	POS	POS	POS	NEG	POS	NEG	AMB	NEG	NEG	NEG	NEG
Iasi-07-65	BSI	POS	POS	POS	POS	POS	NEG	POS	POS	NEG	POS	NEG	NEG	NEG	NEG	POS	NEG	NEG	POS	AMB	POS	POS	NEG	POS	NEG	NEG	AMB	POS	NEG	POS	POS	AMB	POS	AMB	NEG	NEG	NEG	NEG	NEG	POS	AMB	AMB	NEG	NEG	NEG	AMB
Iasi-19-99	BSI	POS	POS	POS	POS	POS	NEG	NEG	AMB	NEG	NEG	POS	NEG	NEG	NEG	NEG	POS	NEG	POS	AMB	AMB	AMB	NEG	POS	NEG	POS	AMB	POS	NEG	NEG	AMB	POS	AMB	AMB	POS	POS	POS	POS	NEG	POS	NEG	AMB	NEG	NEG	NEG	NEG
Iasi-25-217	SSTI	POS	POS	POS	POS	POS	NEG	NEG	AMB	NEG	NEG	POS	NEG	NEG	NEG	NEG	NEG	POS	POS	AMB	POS	POS	NEG	POS	AMB	POS	AMB	POS	NEG	NEG	NEG	AMB	POS	AMB	POS	POS	POS	POS	NEG	POS	AMB	AMB	NEG	NEG	NEG	NEG
Iasi-23-P5	Carrier	POS	POS	POS	POS	POS	NEG	NEG	NEG	NEG	NEG	POS	NEG	NEG	NEG	POS	NEG	NEG	POS	AMB	POS	POS	NEG	POS	NEG	POS	AMB	POS	NEG	NEG	AMB	POS	AMB	NEG	POS	POS	POS	POS	NEG	POS	AMB	AMB	NEG	NEG	NEG	NEG
Iasi-43-G513	Carrier	POS	POS	POS	POS	POS	NEG	NEG	NEG	NEG	NEG	POS	NEG	NEG	NEG	POS	NEG	NEG	POS	AMB	POS	POS	NEG	POS	NEG	POS	AMB	POS	NEG	NEG	NEG	POS	AMB	AMB	POS	POS	POS	POS	NEG	POS	AMB	AMB	NEG	NEG	NEG	NEG
Iasi-44-G514	Carrier	POS	POS	POS	POS	POS	NEG	NEG	NEG	NEG	NEG	POS	NEG	NEG	NEG	POS	NEG	NEG	POS	AMB	POS	POS	NEG	POS	AMB	POS	AMB	POS	NEG	NEG	AMB	POS	AMB	AMB	POS	POS	POS	POS	NEG	POS	AMB	AMB	NEG	NEG	NEG	NEG
Iasi-28-172	BSI	POS	POS	POS	POS	POS	POS	AMB	POS	AMB	POS	NEG	POS	AMB	NEG	NEG	NEG	NEG	POS	NEG	POS	POS	NEG	POS	AMB	NEG	AMB	POS	NEG	POS	POS	POS	AMB	NEG	POS	POS	POS	POS	NEG	POS	AMB	AMB	NEG	NEG	NEG	NEG
Iasi-42-358	BSI	POS	POS	POS	POS	POS	POS	AMB	POS	AMB	POS	NEG	POS	AMB	NEG	NEG	NEG	NEG	POS	NEG	POS	POS	NEG	POS	AMB	NEG	AMB	POS	AMB	POS	POS	POS	AMB	NEG	POS	POS	POS	POS	NEG	POS	AMB	AMB	NEG	NEG	NEG	NEG
Iasi-48-403	BSI	POS	POS	POS	POS	POS	POS	AMB	POS	NEG	POS	NEG	POS	AMB	NEG	NEG	NEG	NEG	POS	NEG	POS	POS	NEG	POS	NEG	NEG	AMB	POS	NEG	POS	POS	POS	AMB	NEG	POS	POS	POS	POS	NEG	POS	AMB	AMB	NEG	NEG	NEG	NEG
Iasi-26-153	BSI	POS	POS	POS	POS	POS	POS	NEG	POS	NEG	POS	NEG	NEG	POS	NEG	NEG	NEG	NEG	POS	NEG	POS	POS	NEG	POS	NEG	NEG	AMB	POS	NEG	POS	POS	POS	AMB	NEG	POS	POS	POS	POS	NEG	POS	AMB	AMB	NEG	NEG	NEG	NEG
Iasi-29-190	BSI	POS	POS	POS	POS	POS	POS	NEG	POS	NEG	POS	NEG	POS	AMB	NEG	NEG	NEG	NEG	POS	NEG	POS	POS	NEG	POS	NEG	NEG	AMB	POS	NEG	POS	POS	POS	AMB	NEG	POS	POS	POS	POS	NEG	POS	AMB	AMB	NEG	NEG	NEG	NEG
Iasi-31-201	BSI	POS	POS	POS	POS	POS	POS	NEG	POS	NEG	POS	NEG	NEG	POS	NEG	NEG	NEG	NEG	POS	NEG	POS	POS	NEG	POS	NEG	NEG	AMB	POS	NEG	POS	POS	POS	AMB	NEG	POS	POS	POS	POS	NEG	POS	AMB	AMB	NEG	NEG	NEG	NEG
Iasi-37-289	BSI	POS	POS	POS	POS	POS	POS	AMB	POS	NEG	POS	NEG	POS	AMB	NEG	NEG	NEG	NEG	POS	NEG	POS	POS	NEG	POS	AMB	NEG	AMB	POS	NEG	POS	POS	POS	AMB	NEG	POS	POS	POS	POS	NEG	POS	AMB	AMB	NEG	NEG	NEG	NEG
Iasi-41-334	BSI	POS	POS	POS	POS	POS	POS	POS	POS	AMB	POS	NEG	POS	AMB	NEG	POS	NEG	NEG	POS	NEG	POS	POS	NEG	POS	AMB	NEG	AMB	POS	AMB	POS	POS	POS	AMB	NEG	POS	POS	POS	POS	NEG	POS	AMB	AMB	NEG	NEG	NEG	NEG
Iasi-52-433	BSI	POS	POS	POS	POS	POS	POS	NEG	POS	NEG	POS	NEG	NEG	POS	NEG	NEG	NEG	NEG	POS	NEG	POS	POS	NEG	POS	NEG	NEG	POS	AMB	NEG	POS	POS	POS	NEG	NEG	POS	POS	POS	POS	NEG	POS	AMB	NEG	NEG	NEG	NEG	NEG
Iasi-07-66	SSTI	POS	POS	POS	POS	POS	POS	NEG	POS	NEG	POS	NEG	NEG	POS	NEG	NEG	NEG	NEG	POS	NEG	POS	POS	NEG	POS	NEG	NEG	POS	AMB	NEG	POS	POS	POS	NEG	NEG	POS	POS	POS	POS	NEG	POS	AMB	NEG	NEG	NEG	NEG	NEG
Iasi-23-211	SSTI	POS	POS	POS	POS	POS	POS	NEG	POS	NEG	POS	NEG	NEG	POS	NEG	NEG	NEG	NEG	POS	NEG	POS	POS	NEG	POS	NEG	NEG	AMB	POS	NEG	POS	POS	POS	AMB	NEG	POS	POS	POS	POS	NEG	POS	AMB	NEG	NEG	NEG	NEG	NEG
Iasi-43-401	SSTI	POS	POS	POS	POS	POS	POS	NEG	POS	NEG	POS	NEG	NEG	POS	NEG	NEG	NEG	NEG	POS	NEG	POS	POS	NEG	POS	NEG	NEG	POS	AMB	NEG	POS	POS	POS	AMB	NEG	POS	POS	POS	POS	NEG	POS	AMB	NEG	NEG	NEG	NEG	NEG
Iasi-47-418	SSTI	POS	POS	POS	POS	POS	POS	NEG	POS	NEG	POS	NEG	NEG	POS	NEG	NEG	NEG	NEG	POS	NEG	POS	POS	NEG	POS	NEG	NEG	POS	AMB	NEG	POS	POS	POS	AMB	NEG	POS	POS	POS	POS	NEG	POS	AMB	NEG	NEG	NEG	NEG	NEG
Iasi-18-169	SSTI	POS	POS	POS	POS	POS	NEG	POS	NEG	POS	NEG	NEG	NEG	NEG	POS	NEG	NEG	NEG	POS	AMB	POS	POS	NEG	POS	NEG	NEG	AMB	POS	AMB	POS	POS	POS	AMB	NEG	POS	POS	POS	POS	NEG	POS	AMB	AMB	NEG	NEG	NEG	NEG
Iasi-18-S34	Carrier	POS	POS	POS	POS	POS	NEG	POS	NEG	POS	NEG	NEG	NEG	NEG	POS	NEG	NEG	NEG	POS	AMB	POS	POS	NEG	POS	AMB	POS	AMB	POS	NEG	POS	POS	AMB	POS	AMB	POS	POS	POS	POS	AMB	POS	AMB	AMB	NEG	NEG	POS	NEG
Iasi-36-276	BSI	POS	POS	POS	POS	POS	NEG	POS	POS	NEG	POS	NEG	NEG	NEG	NEG	POS	NEG	NEG	AMB	POS	NEG	NEG	NEG	NEG	POS	NEG	NEG	NEG	POS	NEG	POS	NEG	POS	AMB	NEG	NEG	NEG	NEG	POS	POS	NEG	AMB	NEG	NEG	NEG	NEG
Iasi-151	BSI	POS	POS	POS	POS	POS	POS	NEG	POS	NEG	POS	NEG	NEG	POS	NEG	NEG	NEG	NEG	AMB	POS	NEG	NEG	NEG	NEG	POS	NEG	POS	NEG	POS	NEG	POS	AMB	POS	AMB	NEG	NEG	NEG	NEG	POS	AMB	NEG	POS	NEG	NEG	NEG	POS
Iasi-02-11	BSI	POS	POS	POS	POS	POS	NEG	POS	NEG	POS	NEG	NEG	NEG	NEG	POS	NEG	NEG	NEG	POS	AMB	POS	POS	NEG	POS	NEG	NEG	AMB	POS	NEG	NEG	NEG	AMB	POS	AMB	POS	POS	POS	POS	NEG	POS	AMB	NEG	NEG	NEG	NEG	NEG

Isolate	Group	VIRULENCE : STAPHYLOCOCCAL SUPERANTIGEN/ENTEROTOXIN-LIKE GENES (SET/SSL)					CAPSULE- AND BIOFILM-ASSOCIATED GENES																	ADHAESION FACTORS / GENES ENCODING MICROBIAL SURFACE COMPONENTS RECOGNIZING ADHESIVE MATRIX MOLECULES (MSCRAMM GENES)																							
		staphylococcal exotoxin-like protein, second locus					Capsule type 1	capsular polysaccharide synthesis enzyme	O-antigen polymerase	capsular polysaccharide biosynthesis protein	Capsule type 5	capsular polysaccharide synthesis enzyme	O-antigen polymerase	capsular polysaccharide biosynthesis protein	Capsule type 8	capsular polysaccharide synthesis enzyme	capsular polysaccharide biosynthesis protein	O-antigen polymerase	capsular polysaccharide biosynthesis protein	intercellular adhesion protein A	intercellular adhesion protein C	biofilm PIA synthesis protein D	surface protein involved in biofilm formation	bone sialoprotein-binding protein							clumping factor A					clumping factor B					collagen-binding adhesin	cell wall associated fibronectin-binding protein	cell surface elastin binding protein				
		setB3	setB3 (MRSA252)	setB2	setB2 (MRSA252)	setB1	cap 1 (total)	capH1	capJ1	capK1	cap 5 (total)	capH5	capJ5	capK5	cap 8 (total)	capH8	capI8	capJ8	capK8	icaA	icaC	icaD	bap	bbp (total)	bbp (consensus)	bbp (COL+MW2)	bbp (MRSA252)	bbp (Mu50)	bbp (RF122)	bbp (ST45)	clfA (total)	clfA (consensus)	clfA (COL+RF122)	clfA (MRSA252)	clfA (Mu50+MW2)	clfB (total)	clfB (consensus)	clfB (COL+Mu50)	clfB (MW2)	clfB (RF122)	cna	ebh (consensus)	ebpS (total)	ebpS_probe 612	ebpS_probe 614	ebpS (01-1111)	ebpS (COL)
Iasi-01-4	BSI	POS	NEG	POS	NEG	POS	NEG	NEG	NEG	NEG	NEG	NEG	NEG	NEG	POS	POS	POS	POS	POS	POS	POS	POS	NEG	POS	POS	POS	NEG	NEG	NEG	NEG	POS	POS	NEG	NEG	POS	POS	POS	NEG	POS	AMB	POS	POS	POS	POS	POS	NEG	NEG
Iasi-05-32	BSI	POS	NEG	POS	NEG	POS	NEG	AMB	NEG	NEG	NEG	NEG	NEG	NEG	POS	POS	POS	POS	POS	POS	POS	POS	NEG	POS	POS	POS	NEG	AMB	NEG	NEG	POS	POS	NEG	NEG	POS	POS	POS	NEG	POS	AMB	POS	POS	POS	POS	POS	NEG	NEG
Iasi-16-88	BSI	POS	NEG	POS	NEG	POS	NEG	AMB	NEG	NEG	NEG	NEG	NEG	NEG	POS	POS	POS	POS	POS	POS	POS	POS	NEG	POS	POS	POS	NEG	AMB	NEG	NEG	POS	POS	NEG	NEG	POS	POS	POS	NEG	AMB	POS	POS	POS	POS	POS	POS	NEG	NEG
Iasi-25-141	BSI	POS	NEG	POS	NEG	POS	NEG	NEG	NEG	AMB	NEG	NEG	NEG	NEG	POS	POS	POS	POS	POS	POS	POS	POS	NEG	POS	POS	POS	NEG	AMB	NEG	NEG	POS	POS	NEG	NEG	POS	POS	POS	AMB	AMB	POS	POS	POS	POS	POS	POS	NEG	NEG
Iasi-32-215	BSI	POS	NEG	POS	NEG	POS	AMB	AMB	NEG	AMB	NEG	NEG	NEG	AMB	POS	POS	POS	POS	POS	POS	POS	POS	NEG	POS	POS	POS	NEG	AMB	NEG	NEG	POS	POS	NEG	NEG	POS	POS	POS	AMB	POS	AMB	POS	POS	POS	POS	POS	NEG	AMB
Iasi-43-359	BSI	POS	NEG	POS	NEG	POS	NEG	NEG	NEG	NEG	NEG	NEG	NEG	NEG	POS	POS	POS	POS	POS	POS	POS	POS	NEG	POS	POS	POS	NEG	AMB	NEG	NEG	POS	POS	NEG	NEG	POS	POS	POS	NEG	POS	AMB	POS	POS	POS	POS	POS	NEG	NEG
Iasi-03-27	SSTI	POS	NEG	POS	NEG	POS	NEG	NEG	NEG	NEG	NEG	NEG	NEG	NEG	POS	POS	POS	POS	POS	POS	POS	POS	NEG	POS	POS	POS	NEG	AMB	NEG	NEG	POS	POS	NEG	NEG	POS	POS	POS	NEG	POS	AMB	POS	POS	POS	POS	POS	NEG	NEG
Iasi-13-149	SSTI	POS	NEG	POS	NEG	POS	NEG	NEG	NEG	NEG	NEG	NEG	NEG	NEG	POS	POS	POS	POS	POS	POS	POS	POS	NEG	POS	POS	POS	NEG	AMB	NEG	NEG	POS	POS	NEG	NEG	POS	POS	POS	NEG	POS	AMB	POS	POS	POS	POS	POS	NEG	NEG
Iasi-29-337	SSTI	POS	NEG	POS	NEG	POS	NEG	NEG	NEG	NEG	NEG	NEG	NEG	NEG	POS	POS	POS	POS	POS	POS	POS	POS	NEG	POS	POS	POS	NEG	NEG	NEG	NEG	POS	POS	NEG	NEG	POS	POS	POS	NEG	POS	AMB	POS	POS	POS	POS	POS	NEG	NEG
Iasi-31-355	SSTI	POS	NEG	POS	NEG	POS	NEG	NEG	NEG	NEG	NEG	NEG	NEG	NEG	POS	POS	POS	POS	POS	POS	POS	POS	NEG	POS	POS	POS	NEG	AMB	NEG	NEG	POS	POS	NEG	NEG	POS	POS	POS	NEG	POS	AMB	POS	POS	POS	POS	POS	NEG	NEG
Iasi-38-377	SSTI	POS	NEG	POS	NEG	POS	NEG	NEG	NEG	NEG	NEG	NEG	NEG	NEG	POS	POS	POS	POS	POS	POS	POS	POS	NEG	POS	POS	POS	NEG	NEG	NEG	NEG	POS	POS	NEG	NEG	POS	POS	POS	NEG	POS	AMB	POS	POS	POS	POS	POS	NEG	NEG
Iasi-41-388	SSTI	POS	NEG	POS	NEG	POS	NEG	NEG	NEG	NEG	NEG	NEG	NEG	NEG	POS	POS	POS	POS	POS	POS	POS	POS	NEG	POS	POS	POS	NEG	AMB	NEG	NEG	POS	POS	NEG	NEG	POS	POS	POS	NEG	POS	AMB	POS	POS	POS	POS	POS	NEG	NEG
Iasi-46-417	SSTI	POS	NEG	POS	NEG	POS	NEG	NEG	NEG	NEG	NEG	NEG	NEG	NEG	POS	POS	POS	POS	POS	POS	POS	POS	NEG	POS	POS	POS	NEG	AMB	NEG	NEG	POS	POS	NEG	NEG	POS	POS	POS	NEG	POS	AMB	POS	POS	POS	POS	POS	NEG	NEG
Iasi-02-S9	Carrier	POS	NEG	POS	NEG	POS	NEG	NEG	NEG	NEG	NEG	NEG	NEG	NEG	POS	POS	POS	POS	POS	POS	POS	POS	NEG	POS	POS	POS	NEG	AMB	NEG	AMB	POS	POS	NEG	NEG	POS	POS	POS	AMB	AMB	POS	POS	POS	POS	POS	POS	NEG	NEG
Iasi-17-S33	Carrier	POS	NEG	POS	NEG	POS	NEG	NEG	NEG	NEG	NEG	NEG	NEG	NEG	POS	POS	POS	POS	POS	POS	POS	POS	NEG	POS	POS	POS	NEG	AMB	NEG	NEG	POS	POS	NEG	NEG	POS	POS	POS	NEG	AMB	POS	POS	POS	POS	POS	POS	NEG	NEG
Iasi-30-P17	Carrier	POS	NEG	POS	NEG	POS	NEG	POS	NEG	NEG	NEG	NEG	NEG	NEG	POS	POS	POS	POS	POS	POS	POS	POS	NEG	POS	POS	POS	NEG	AMB	NEG	NEG	POS	POS	NEG	NEG	POS	POS	POS	NEG	POS	AMB	POS	POS	POS	POS	POS	NEG	NEG
Iasi-39-P28	Carrier	POS	NEG	POS	NEG	POS	NEG	NEG	NEG	NEG	NEG	NEG	NEG	NEG	POS	POS	POS	POS	POS	POS	POS	POS	NEG	POS	POS	POS	NEG	AMB	NEG	NEG	POS	POS	NEG	NEG	POS	POS	POS	NEG	AMB	POS	POS	POS	POS	POS	POS	NEG	NEG
Iasi-04-26	BSI	POS	NEG	POS	NEG	POS	NEG	NEG	NEG	NEG	NEG	NEG	NEG	NEG	POS	POS	POS	POS	POS	POS	POS	POS	NEG	POS	POS	POS	NEG	AMB	NEG	NEG	POS	POS	NEG	NEG	POS	POS	POS	NEG	POS	AMB	POS	POS	POS	POS	POS	NEG	NEG
Iasi-09-72	BSI	POS	NEG	POS	NEG	POS	NEG	NEG	NEG	NEG	NEG	NEG	NEG	NEG	POS	POS	POS	POS	POS	POS	POS	POS	NEG	POS	POS	POS	NEG	NEG	NEG	NEG	POS	POS	NEG	NEG	POS	POS	POS	NEG	POS	AMB	POS	POS	POS	POS	POS	NEG	NEG
Iasi-11-131	BSI	POS	NEG	POS	NEG	POS	NEG	NEG	NEG	NEG	NEG	NEG	NEG	NEG	POS	POS	POS	POS	POS	POS	POS	POS	NEG	POS	POS	POS	NEG	NEG	NEG	NEG	POS	POS	NEG	NEG	POS	POS	POS	NEG	POS	AMB	POS	POS	POS	POS	POS	NEG	NEG
Iasi-14-143	BSI	POS	NEG	POS	NEG	POS	NEG	NEG	NEG	NEG	NEG	NEG	NEG	NEG	POS	POS	POS	POS	POS	POS	POS	POS	NEG	POS	POS	POS	NEG	NEG	NEG	NEG	POS	POS	NEG	NEG	POS	POS	POS	NEG	POS	AMB	POS	POS	POS	POS	POS	NEG	NEG
Iasi-18-192	BSI	POS	NEG	POS	NEG	POS	NEG	NEG	NEG	NEG	NEG	NEG	NEG	NEG	POS	POS	POS	POS	POS	POS	POS	POS	NEG	POS	POS	POS	NEG	NEG	NEG	NEG	POS	POS	NEG	NEG	POS	POS	POS	NEG	POS	AMB	POS	POS	POS	POS	POS	NEG	NEG
Iasi-23-253	BSI	POS	NEG	POS	NEG	POS	NEG	NEG	NEG	AMB	NEG	NEG	NEG	NEG	POS	POS	POS	POS	POS	POS	POS	POS	NEG	POS	POS	POS	NEG	AMB	NEG	NEG	POS	POS	NEG	NEG	POS	POS	POS	AMB	POS	AMB	POS	POS	POS	POS	POS	NEG	NEG
Iasi-35-322	BSI	POS	NEG	POS	NEG	POS	NEG	NEG	NEG	NEG	NEG	NEG	NEG	NEG	POS	POS	POS	POS	POS	POS	POS	POS	NEG	POS	POS	POS	NEG	NEG	NEG	NEG	POS	POS	NEG	NEG	POS	POS	POS	NEG	POS	AMB	POS	POS	POS	POS	POS	NEG	NEG
Iasi-40-380	BSI	POS	NEG	POS	NEG	POS	NEG	NEG	NEG	AMB	NEG	NEG	NEG	NEG	POS	POS	POS	POS	POS	POS	POS	POS	NEG	POS	POS	POS	NEG	AMB	NEG	NEG	POS	POS	NEG	NEG	POS	POS	POS	NEG	POS	AMB	POS	POS	POS	POS	POS	NEG	NEG
Iasi-46-406	BSI	POS	NEG	POS	NEG	POS	NEG	NEG	NEG	NEG	NEG	NEG	NEG	NEG	POS	POS	POS	POS	POS	POS	POS	POS	NEG	POS	POS	POS	NEG	NEG	NEG	NEG	POS	POS	NEG	NEG	POS	POS	POS	NEG	POS	AMB	POS	POS	POS	POS	POS	NEG	NEG
Iasi-02-6	SSTI	POS	NEG	POS	NEG	POS	NEG	NEG	NEG	NEG	NEG	NEG	NEG	NEG	POS	POS	POS	POS	POS	POS	POS	POS	NEG	POS	POS	POS	NEG	NEG	NEG	NEG	POS	POS	NEG	NEG	POS	POS	POS	NEG	POS	AMB	POS	POS	POS	POS	POS	NEG	NEG
Iasi-09-97	SSTI	POS	NEG	POS	NEG	POS	NEG	NEG	NEG	NEG	NEG	NEG	NEG	NEG	POS	POS	POS	POS	POS	POS	POS	POS	NEG	POS	POS	POS	NEG	NEG	NEG	NEG	POS	POS	NEG	NEG	POS	POS	POS	NEG	POS	AMB	POS	POS	POS	POS	POS	NEG	NEG
Iasi-19-182	SSTI	POS	NEG	POS	NEG	POS	NEG	NEG	NEG	NEG	NEG	NEG	NEG	NEG	POS	POS	POS	POS	POS	POS	POS	POS	NEG	POS	POS	POS	NEG	NEG	NEG	NEG	POS	POS	NEG	NEG	POS	POS	POS	NEG	POS	AMB	POS	POS	POS	POS	POS	NEG	NEG
Iasi-31-314	SSTI	POS	NEG	POS	NEG	POS	NEG	NEG	NEG	NEG	NEG	NEG	NEG	NEG	POS	POS	POS	POS	POS	POS	POS	POS	NEG	POS	POS	POS	NEG	NEG	NEG	NEG	POS	POS	NEG	NEG	POS	POS	POS	NEG	POS	AMB	POS	POS	POS	POS	POS	NEG	NEG
Iasi-33-367	SSTI	POS	NEG	POS	NEG	POS	NEG	NEG	NEG	NEG	NEG	NEG	NEG	NEG	POS	POS	POS	POS	POS	POS	POS	POS	NEG	POS	POS	POS	NEG	NEG	NEG	NEG	POS	POS	NEG	NEG	POS	POS	POS	NEG	POS	AMB	POS	POS	POS	POS	POS	NEG	NEG
Iasi-39-378	SSTI	POS	NEG	POS	NEG	POS	NEG	NEG	NEG	NEG	NEG	NEG	NEG	NEG	POS	POS	POS	POS	POS	POS	POS	POS	NEG	POS	POS	POS	NEG	AMB	NEG	NEG	POS	POS	NEG	NEG	POS	POS	POS	NEG	POS	AMB	POS	POS	POS	POS	POS	NEG	NEG
Iasi-42-394	SSTI	POS	NEG	POS	NEG	POS	NEG	NEG	NEG	NEG	NEG	NEG	NEG	NEG	POS	POS	POS	POS	POS	POS	POS	POS	NEG	POS	POS	POS	NEG	NEG	NEG	NEG	POS	POS	NEG	NEG	POS	POS	POS	NEG	POS	AMB	POS	POS	POS	POS	POS	NEG	NEG
Iasi-44-409	SSTI	POS	NEG	POS	NEG	POS	NEG	NEG	NEG	NEG	NEG	NEG	NEG	NEG	POS	POS	POS	POS	POS	POS	POS	POS	NEG	POS	POS	POS	NEG	NEG	NEG	NEG	POS	POS	NEG	NEG	POS	POS	POS	NEG	POS	AMB	POS	POS	POS	POS	POS	NEG	NEG
Iasi-45-405	SSTI	POS	NEG	POS	NEG	POS	NEG	NEG	NEG	NEG	NEG	NEG	NEG	NEG	POS	POS	POS	POS	POS	POS	POS	POS	NEG	POS	POS	POS	NEG	NEG	NEG	NEG	POS	POS	NEG	NEG	POS	POS	POS	NEG	POS	AMB	POS	POS	POS	POS	POS	NEG	NEG
Iasi-50-425	SSTI	POS	NEG	POS	NEG	POS	NEG	NEG	NEG	NEG	NEG	NEG	NEG	NEG	POS	POS	POS	POS	POS	POS	POS	POS	NEG	POS	POS	POS	NEG	NEG	NEG	NEG	POS	POS	NEG	NEG	POS	POS	POS	NEG	POS	AMB	POS	POS	POS	POS	POS	NEG	NEG
Iasi-50-428	SSTI	POS	NEG	POS	NEG	POS	NEG	NEG	NEG	NEG	NEG	NEG	NEG	NEG	POS	POS	POS	POS	POS	POS	POS	POS	NEG	POS	POS	POS	NEG	AMB	NEG	NEG	POS	POS	NEG	NEG	POS	POS	POS	NEG	POS	AMB	POS	POS	POS	POS	POS	NEG	NEG
Iasi-51-37	SSTI	POS	NEG	POS	NEG	POS	NEG	NEG	NEG	NEG	NEG	NEG	NEG	NEG	POS	POS	POS	POS	POS	POS	POS	POS	NEG	POS	POS	POS	NEG	NEG	NEG	NEG	POS	POS	NEG	NEG	POS	POS	POS	NEG	AMB	POS	POS	POS	POS	POS	POS	NEG	NEG
Iasi-01-S8	Carrier	POS	NEG	POS	NEG	POS	NEG	NEG	NEG	NEG	NEG	NEG	NEG	NEG	POS	POS	POS	POS	POS	POS	POS	POS	NEG	POS	POS	POS	NEG	AMB	NEG	NEG	POS	POS	NEG	NEG	POS	POS	POS	NEG	POS	AMB	POS	POS	POS	POS	POS	NEG	NEG
Iasi-08-S17	Carrier	POS	NEG	POS	NEG	POS	NEG	NEG	NEG	NEG	NEG	NEG	NEG	NEG	POS	POS	POS	POS	POS	POS	POS	POS	NEG	POS	POS	POS	NEG	AMB	NEG	NEG	POS	POS	NEG	NEG	POS	POS	POS	NEG	AMB	POS	POS	POS	POS	POS	POS	NEG	NEG
Iasi-10-S23	Carrier	POS	NEG	POS	NEG	POS	NEG	NEG	NEG	NEG	NEG	NEG	NEG	NEG	POS	POS	POS	POS	POS	POS	POS	POS	NEG	POS	POS	POS	NEG	AMB	NEG	NEG	POS	POS	NEG	NEG	POS	POS	POS	NEG	AMB	POS	POS	POS	POS	POS	POS	NEG	NEG
Iasi-13-S27	Carrier	POS	NEG	POS	NEG	POS	NEG	NEG	NEG	NEG	NEG	NEG	NEG	NEG	POS	POS	POS	POS	POS	POS	POS	POS	NEG	POS	POS	POS	NEG	AMB	NEG	NEG	POS	POS	NEG	NEG	POS	POS	POS	NEG	POS	AMB	POS	POS	POS	POS	POS	NEG	NEG
Iasi-14-S28	Carrier	POS	NEG	POS	NEG	POS	NEG	NEG	NEG	NEG	NEG	NEG	NEG	NEG	POS	POS	POS	POS	POS	POS	POS	POS	NEG	POS	POS	POS	NEG	AMB	NEG	NEG	POS	POS	NEG	NEG	POS	POS	POS	NEG	POS	AMB	POS	POS	POS	POS	POS	NEG	NEG
Iasi-15-S29	Carrier	POS	NEG	POS	NEG	POS	AMB	AMB	NEG	AMB	NEG	NEG	NEG	NEG	POS	POS	POS	POS	POS	POS	POS	POS	NEG	POS	POS	POS	NEG	AMB	NEG	NEG	POS	POS	NEG	NEG	POS	POS	POS	AMB	POS	AMB	POS	POS	POS	POS	POS	NEG	NEG
Iasi-25-P10	Carrier	POS	NEG	POS	NEG	POS	NEG	AMB	NEG	AMB	NEG	NEG	NEG	NEG	POS	POS	POS	POS	POS	POS	POS	POS	NEG	POS	POS	POS	NEG	AMB	NEG	NEG	POS	POS	NEG	NEG	POS	POS	POS	NEG	POS	AMB	POS	POS	POS	POS	POS	NEG	NEG
Iasi-26-P11	Carrier	POS	NEG	POS	NEG	POS	NEG	NEG	NEG	NEG	NEG	NEG	NEG	NEG	POS	POS	POS	POS	POS	POS	POS	POS	NEG	POS	POS	POS	NEG	AMB	NEG	NEG	POS	POS	NEG	NEG	POS	POS	POS	NEG	POS	AMB	POS	POS	POS	POS	POS	NEG	NEG
Iasi-32-P19	Carrier	POS	NEG	POS	NEG	POS	NEG	NEG	NEG	NEG	NEG	NEG	NEG	NEG	POS	POS	POS	POS	POS	POS	POS	POS	NEG	POS	POS	POS	NEG	AMB	NEG	NEG	POS	POS	NEG	NEG	POS	POS	POS	NEG	POS	AMB	POS	POS	POS	POS	POS	NEG	NEG
Iasi-33-P22	Carrier	POS	NEG	POS	NEG	POS	NEG	NEG	NEG	NEG	NEG	NEG	NEG	NEG	POS	POS	POS	POS	POS	POS	POS	POS	NEG	POS	POS	POS	NEG	AMB	NEG	NEG	POS	POS	NEG	NEG	POS	POS	POS	NEG	AMB	POS	POS	POS	POS	POS	POS	NEG	NEG
Iasi-41-P44	Carrier	POS	NEG	POS	NEG	POS	NEG	NEG	NEG	NEG	NEG	NEG	NEG	NEG	POS	POS	POS	POS	POS	POS	POS	POS	NEG	POS	POS	POS	NEG	AMB	NEG	NEG	POS	POS	NEG	NEG	POS	POS	POS	NEG	AMB	POS	POS	POS	POS	POS	POS	NEG	NEG
Iasi-45-G515	Carrier	POS	NEG	POS	NEG	POS	NEG	NEG	NEG	NEG	NEG	NEG	NEG	NEG	POS	POS	POS	POS	POS	POS	POS	POS	NEG	POS	POS	POS	NEG	AMB	NEG	NEG	POS	POS	NEG	NEG	POS	POS	POS	NEG	AMB	POS	POS	POS	POS	POS	POS	NEG	NEG
Iasi-02-39	misc. MRSA	POS	NEG	POS	NEG	POS	NEG	NEG	NEG	NEG	NEG	NEG	NEG	NEG	POS	POS	POS	POS	POS	POS	POS	POS	NEG	POS	POS	POS	NEG	NEG	NEG	NEG	POS	POS	NEG	NEG	POS	POS	POS	NEG	POS	AMB	POS	POS	POS	POS	POS	NEG	NEG
Iasi-24-263	misc. MRSA	POS	NEG	POS	NEG	POS	NEG	NEG	NEG	NEG	NEG	NEG	NEG	NEG	POS	POS	POS	POS	POS	POS	POS	POS	NEG	POS	POS	POS	NEG	AMB	NEG	NEG	POS	POS	NEG	NEG	POS	POS	POS	NEG	POS	AMB	POS	POS	POS	POS	POS	NEG	NEG
Iasi-26-285	misc. MRSA	POS	NEG	POS	NEG	POS	NEG	NEG	NEG	NEG	NEG	NEG	NEG	NEG	POS	POS	POS	POS	POS	POS	POS	POS	NEG	POS	POS	POS	NEG	AMB	NEG	NEG	POS	POS	NEG	NEG	POS	POS	POS	NEG	POS	AMB	POS	POS	POS	POS	POS	NEG	NEG
Iasi-30-306	misc. MRSA	POS	NEG	POS	NEG	POS	AMB	AMB	NEG	AMB	NEG	NEG	NEG	NEG	POS	POS	POS	POS	POS	POS	POS	POS	NEG	POS	POS	POS	NEG	AMB	NEG	AMB	POS	POS	NEG	NEG	POS	POS	POS	AMB	POS	AMB	POS	POS	POS	POS	POS	NEG	NEG
Iasi-41-390	misc. MRSA	POS	NEG	POS	NEG	POS	NEG	NEG	NEG	NEG	NEG	NEG	NEG	NEG	POS	POS	POS	POS	POS	POS	POS	POS	NEG	POS	POS	POS	NEG	AMB	NEG	NEG	POS	POS	NEG	NEG	POS	POS	POS	NEG	POS	AMB	POS	POS	POS	POS	POS	NEG	NEG
Iasi-47-407	misc. MRSA	POS	NEG	POS	NEG	POS	NEG	NEG	NEG	AMB	NEG	NEG	NEG	NEG	POS	POS	POS	POS	POS	POS	POS	POS	NEG	POS	POS	POS	NEG	AMB	NEG	AMB	POS	POS	NEG	NEG	POS	POS	POS	NEG	POS	AMB	POS	POS	POS	POS	POS	NEG	NEG
Iasi-55-408CP	misc. MRSA	POS	NEG	POS	NEG	POS	NEG	NEG	NEG	NEG	NEG	NEG	NEG	NEG	POS	POS	POS	POS	POS	POS	POS	POS	NEG	POS	POS	POS	NEG	NEG	NEG	NEG	POS	POS	NEG	NEG	POS	POS	POS	NEG	POS	AMB	POS	POS	POS	POS	POS	NEG	NEG
Iasi-15-151	BSI	POS	NEG	POS	NEG	POS	AMB	AMB	AMB	NEG	POS	POS	POS	POS	NEG	NEG	NEG	NEG	NEG	POS	POS	POS	NEG	POS	POS	AMB	NEG	POS	NEG	AMB	POS	POS	NEG	NEG	POS	POS	POS	POS	NEG	NEG	NEG	POS	POS	POS	POS	NEG	NEG
Iasi-46-386	BSI	POS	NEG	POS	NEG	POS	NEG	NEG	POS	NEG	POS	POS	POS	POS	NEG	NEG	NEG	NEG	NEG	POS	POS	POS	NEG	POS	POS	AMB	NEG	POS	NEG	NEG	POS	POS	AMB	NEG	POS	POS	POS	POS	AMB	NEG	NEG	POS	POS	POS	POS	NEG	NEG
Iasi-21-195	SSTI	POS	NEG	POS	NEG	POS	NEG	NEG	NEG	NEG	POS	POS	POS	POS	NEG	NEG	NEG	NEG	NEG	POS	POS	POS	NEG	POS	POS	NEG	NEG	POS	NEG	NEG	POS	POS	NEG	NEG	POS	POS	POS	POS	NEG	NEG	NEG	POS	POS	POS	POS	NEG	NEG
Iasi-37-375	SSTI	POS	NEG	POS	NEG	POS	NEG	NEG	NEG	NEG	POS	POS	POS	POS	NEG	NEG	NEG	NEG	NEG	POS	POS	POS	NEG	POS	POS	NEG	NEG	POS	NEG	NEG	POS	POS	NEG	NEG	POS	POS	POS	POS	NEG	NEG	NEG	POS	POS	POS	POS	NEG	NEG
Iasi-45-415	SSTI	POS	NEG	POS	NEG	POS	NEG	NEG	NEG	NEG	POS	POS	POS	POS	NEG	NEG	NEG	NEG	NEG	POS	POS	POS	NEG	POS	POS	NEG	NEG	POS	NEG	NEG	POS	POS	NEG	NEG	POS	POS	POS	POS	NEG	NEG	NEG	POS	POS	POS	POS	NEG	NEG
Iasi-09-S19	Carrier	POS	NEG	POS	NEG	POS	NEG	NEG	AMB	NEG	POS	POS	POS	POS	NEG	NEG	NEG	NEG	NEG	POS	POS	POS	NEG	POS	POS	AMB	NEG	POS	NEG	AMB	POS	POS	NEG	NEG	POS	POS	POS	POS	AMB	NEG	NEG	POS	POS	POS	POS	NEG	NEG
Iasi-53-434	BSI	POS	NEG	POS	NEG	POS	NEG	NEG	AMB	NEG	POS	POS	POS	POS	NEG	NEG	NEG	NEG	NEG	POS	POS	POS	NEG	POS	POS	AMB	NEG	POS	NEG	NEG	POS	POS	NEG	NEG	POS	POS	POS	POS	AMB	NEG	NEG	POS	POS	POS	POS	NEG	NEG
Iasi-03-40	misc. MRSA	POS	NEG	POS	NEG	POS	AMB	AMB	AMB	NEG	POS	POS	POS	POS	NEG	NEG	NEG	NEG	NEG	POS	POS	POS	NEG	POS	POS	AMB	NEG	POS	NEG	AMB	POS	POS	NEG	NEG	POS	POS	POS	POS	NEG	NEG	NEG	POS	POS	POS	POS	NEG	NEG
Iasi-04-43	misc. MRSA	POS	NEG	POS	NEG	POS	NEG	NEG	NEG	NEG	POS	POS	POS	POS	NEG	NEG	NEG	NEG	NEG	POS	POS	POS	NEG	POS	POS	NEG	NEG	POS	NEG	NEG	POS	POS	NEG	NEG	POS	POS	POS	POS	NEG	NEG	NEG	POS	POS	POS	POS	NEG	NEG
Iasi-01-20	misc. MRSA	POS	NEG	POS	NEG	POS	NEG	NEG	NEG	NEG	POS	POS	POS	POS	NEG	NEG	NEG	NEG	NEG	POS	POS	POS	NEG	POS	POS	AMB	NEG	POS	NEG	NEG	POS	POS	NEG	NEG	POS	POS	POS	POS	NEG	NEG	NEG	POS	POS	POS	POS	NEG	NEG
Iasi-35-228	BSI	POS	NEG	POS	NEG	POS	NEG	AMB	NEG	NEG	NEG	NEG	NEG	NEG	POS	POS	POS	POS	POS	POS	POS	POS	NEG	POS	POS	POS	NEG	AMB	NEG	NEG	POS	POS	NEG	NEG	POS	POS	POS	NEG	NEG	POS	POS	POS	POS	POS	POS	NEG	NEG
Iasi-38-293	BSI	POS	NEG	POS	NEG	POS	AMB	POS	NEG	AMB	NEG	NEG	NEG	AMB	POS	POS	POS	POS	POS	POS	POS	POS	NEG	POS	POS	POS	AMB	NEG	NEG	NEG	POS	POS	AMB	POS	AMB	POS	POS	AMB	NEG	POS	NEG	POS	POS	POS	POS	AMB	POS
Iasi-31-P18	Carrier	POS	NEG	POS	NEG	POS	NEG	POS	NEG	NEG	NEG	NEG	NEG	NEG	POS	POS	POS	POS	POS	POS	POS	POS	NEG	POS	POS	POS	AMB	NEG	NEG	AMB	POS	POS	POS	AMB	AMB	POS	POS	NEG	NEG	POS	NEG	POS	POS	POS	POS	NEG	POS
Iasi-40-P42	Carrier	POS	NEG	POS	NEG	POS	AMB	POS	NEG	AMB	NEG	NEG	NEG	NEG	POS	POS	POS	POS	POS	POS	POS	POS	NEG	POS	POS	POS	AMB	NEG	NEG	AMB	POS	POS	AMB	POS	AMB	POS	POS	NEG	NEG	POS	NEG	POS	POS	POS	POS	NEG	POS
Iasi-40-303	BSI	POS	NEG	POS	NEG	POS	NEG	NEG	NEG	NEG	POS	POS	POS	POS	NEG	NEG	NEG	NEG	NEG	POS	POS	POS	NEG	NEG	NEG	NEG	NEG	NEG	NEG	NEG	POS	POS	AMB	AMB	POS	POS	POS	POS	NEG	NEG	NEG	POS	POS	POS	POS	NEG	POS
Iasi-48-422	SSTI	POS	NEG	POS	NEG	POS	NEG	NEG	NEG	NEG	POS	POS	POS	POS	NEG	NEG	NEG	NEG	NEG	POS	POS	POS	NEG	POS	POS	POS	NEG	NEG	NEG	NEG	POS	POS	POS	AMB	AMB	POS	POS	POS	NEG	NEG	NEG	POS	POS	POS	POS	NEG	POS
Iasi-54-435	BSI	POS	NEG	POS	NEG	POS	AMB	AMB	POS	NEG	POS	POS	POS	POS	NEG	NEG	NEG	NEG	NEG	POS	POS	POS	NEG	POS	POS	POS	NEG	AMB	NEG	NEG	POS	POS	AMB	POS	AMB	POS	POS	POS	AMB	NEG	NEG	POS	POS	POS	POS	AMB	POS
Iasi-16-161	SSTI	POS	NEG	POS	NEG	POS	NEG	NEG	NEG	NEG	POS	POS	POS	POS	NEG	NEG	NEG	NEG	NEG	POS	POS	POS	NEG	POS	POS	POS	NEG	AMB	NEG	NEG	POS	POS	AMB	AMB	POS	POS	POS	POS	NEG	NEG	NEG	POS	POS	POS	POS	NEG	POS
Iasi-27-221	SSTI	POS	NEG	POS	NEG	POS	NEG	NEG	NEG	NEG	POS	POS	POS	POS	NEG	NEG	NEG	NEG	NEG	POS	POS	POS	NEG	POS	POS	POS	NEG	NEG	NEG	NEG	POS	POS	AMB	AMB	POS	POS	POS	POS	NEG	NEG	NEG	POS	POS	POS	POS	NEG	POS
Iasi-09-85	BSI	POS	NEG	POS	NEG	POS	NEG	NEG	NEG	NEG	POS	POS	POS	POS	NEG	NEG	NEG	NEG	NEG	POS	POS	POS	NEG	POS	POS	POS	NEG	NEG	NEG	NEG	POS	POS	POS	AMB	AMB	POS	POS	POS	NEG	NEG	NEG	POS	POS	POS	POS	NEG	POS
Iasi-17-189	BSI	POS	NEG	POS	NEG	POS	NEG	NEG	NEG	NEG	POS	POS	POS	POS	NEG	NEG	NEG	NEG	NEG	POS	POS	POS	NEG	POS	POS	POS	NEG	NEG	NEG	NEG	POS	POS	POS	AMB	AMB	POS	POS	POS	NEG	NEG	NEG	POS	POS	POS	POS	NEG	POS
Iasi-20-200	BSI	POS	NEG	POS	NEG	POS	NEG	NEG	NEG	NEG	POS	POS	POS	POS	NEG	NEG	NEG	NEG	NEG	POS	POS	POS	NEG	POS	POS	POS	NEG	NEG	NEG	NEG	POS	POS	POS	AMB	AMB	POS	POS	POS	NEG	NEG	NEG	POS	POS	POS	POS	NEG	POS
Iasi-34-321	BSI	POS	NEG	POS	NEG	POS	NEG	NEG	NEG	NEG	POS	POS	POS	POS	NEG	NEG	NEG	NEG	NEG	POS	POS	POS	NEG	POS	POS	POS	NEG	NEG	NEG	NEG	POS	POS	POS	AMB	AMB	POS	POS	POS	NEG	NEG	NEG	POS	POS	POS	POS	NEG	POS
Iasi-04-57	SSTI	POS	NEG	POS	NEG	POS	NEG	NEG	NEG	NEG	POS	POS	POS	POS	NEG	NEG	NEG	NEG	NEG	POS	POS	POS	NEG	POS	POS	POS	NEG	NEG	NEG	NEG	POS	POS	POS	AMB	AMB	POS	POS	POS	NEG	NEG	NEG	POS	POS	POS	POS	NEG	POS
Iasi-05-59	SSTI	POS	NEG	POS	NEG	POS	NEG	NEG	NEG	NEG	POS	POS	POS	POS	NEG	NEG	NEG	NEG	NEG	POS	POS	POS	NEG	POS	POS	POS	NEG	NEG	NEG	NEG	POS	POS	POS	AMB	AMB	POS	POS	POS	NEG	NEG	NEG	POS	POS	POS	POS	NEG	POS
Iasi-06-64	SSTI	POS	NEG	POS	NEG	POS	NEG	NEG	NEG	NEG	POS	POS	POS	POS	NEG	NEG	NEG	NEG	NEG	POS	POS	POS	NEG	POS	POS	POS	NEG	NEG	NEG	NEG	POS	POS	POS	AMB	AMB	POS	POS	POS	NEG	NEG	NEG	POS	POS	POS	POS	NEG	POS
Iasi-10-108	SSTI	POS	NEG	POS	NEG	POS	NEG	NEG	NEG	NEG	POS	POS	POS	POS	NEG	NEG	NEG	NEG	NEG	POS	POS	POS	NEG	POS	POS	POS	NEG	NEG	NEG	NEG	POS	POS	POS	AMB	AMB	POS	POS	POS	NEG	NEG	NEG	POS	POS	POS	POS	NEG	POS
Iasi-12-117	SSTI	POS	NEG	POS	NEG	POS	NEG	NEG	NEG	NEG	POS	POS	POS	POS	NEG	NEG	NEG	NEG	NEG	POS	POS	POS	NEG	POS	POS	POS	NEG	AMB	NEG	NEG	POS	POS	AMB	AMB	POS	POS	POS	POS	NEG	NEG	NEG	POS	POS	POS	POS	NEG	POS
Iasi-12-138	SSTI	POS	NEG	POS	NEG	POS	NEG	NEG	NEG	NEG	POS	POS	POS	POS	NEG	NEG	NEG	NEG	NEG	POS	POS	POS	NEG	POS	POS	POS	NEG	NEG	NEG	NEG	POS	POS	POS	AMB	AMB	POS	POS	POS	NEG	NEG	NEG	POS	POS	POS	POS	NEG	POS
Iasi-13-140	SSTI	POS	NEG	POS	NEG	POS	NEG	NEG	NEG	NEG	POS	POS	POS	POS	NEG	NEG	NEG	NEG	NEG	POS	POS	POS	NEG	POS	POS	POS	NEG	NEG	NEG	NEG	POS	POS	POS	AMB	AMB	POS	POS	POS	NEG	NEG	NEG	POS	POS	POS	POS	NEG	POS
Iasi-15-152	SSTI	POS	NEG	POS	NEG	POS	NEG	NEG	NEG	NEG	POS	POS	POS	POS	NEG	NEG	NEG	NEG	NEG	POS	POS	POS	NEG	POS	POS	POS	NEG	AMB	NEG	NEG	POS	POS	POS	AMB	AMB	POS	POS	POS	NEG	NEG	NEG	POS	POS	POS	POS	NEG	POS
Iasi-17-168	SSTI	POS	NEG	POS	NEG	POS	NEG	NEG	NEG	NEG	POS	POS	POS	POS	NEG	NEG	NEG	NEG	NEG	POS	POS	POS	NEG	POS	POS	POS	NEG	AMB	NEG	NEG	POS	POS	POS	AMB	AMB	POS	POS	POS	NEG	NEG	NEG	POS	POS	POS	POS	NEG	POS
Iasi-20-193	SSTI	POS	NEG	POS	NEG	POS	NEG	NEG	NEG	NEG	POS	POS	POS	POS	NEG	NEG	NEG	NEG	NEG	POS	POS	POS	NEG	POS	POS	POS	NEG	NEG	NEG	NEG	POS	POS	AMB	AMB	POS	POS	POS	POS	NEG	NEG	NEG	POS	POS	POS	POS	NEG	POS
Iasi-26-218	SSTI	POS	NEG	POS	NEG	POS	NEG	NEG	NEG	NEG	POS	POS	POS	POS	NEG	NEG	NEG	NEG	NEG	POS	POS	POS	NEG	POS	POS	POS	NEG	NEG	NEG	NEG	POS	POS	AMB	AMB	POS	POS	POS	POS	NEG	NEG	NEG	POS	POS	POS	POS	NEG	POS
Iasi-28-335	SSTI	POS	NEG	POS	NEG	POS	NEG	NEG	NEG	NEG	POS	POS	POS	POS	NEG	NEG	NEG	NEG	NEG	POS	POS	POS	NEG	POS	POS	POS	NEG	NEG	NEG	NEG	POS	POS	POS	AMB	AMB	POS	POS	POS	NEG	NEG	NEG	POS	POS	POS	POS	NEG	POS
Iasi-29-299b	SSTI	POS	NEG	POS	NEG	POS	NEG	AMB	NEG	NEG	POS	POS	POS	POS	NEG	NEG	NEG	NEG	NEG	POS	POS	POS	NEG	POS	POS	POS	NEG	AMB	NEG	NEG	POS	POS	AMB	AMB	POS	POS	POS	POS	NEG	NEG	NEG	POS	POS	POS	POS	NEG	POS
Iasi-32-364	SSTI	POS	NEG	POS	NEG	POS	NEG	NEG	NEG	NEG	POS	POS	POS	POS	NEG	NEG	NEG	NEG	NEG	POS	POS	POS	NEG	POS	POS	POS	NEG	NEG	NEG	NEG	POS	POS	POS	AMB	AMB	POS	POS	POS	NEG	NEG	NEG	POS	POS	POS	POS	NEG	POS
Iasi-369	SSTI	POS	NEG	POS	NEG	POS	NEG	NEG	NEG	NEG	POS	POS	POS	POS	NEG	NEG	NEG	NEG	NEG	POS	POS	POS	NEG	POS	POS	POS	NEG	NEG	NEG	NEG	POS	POS	AMB	AMB	POS	POS	POS	POS	NEG	NEG	NEG	POS	POS	POS	POS	NEG	POS
Iasi-03-S11	Carrier	POS	NEG	POS	NEG	POS	NEG	AMB	NEG	NEG	POS	POS	POS	POS	NEG	NEG	NEG	NEG	NEG	POS	POS	POS	NEG	POS	POS	POS	NEG	AMB	NEG	NEG	POS	POS	AMB	AMB	POS	POS	POS	POS	NEG	NEG	NEG	POS	POS	POS	POS	NEG	POS
Iasi-19-S35	Carrier	POS	NEG	POS	NEG	POS	AMB	AMB	AMB	NEG	POS	POS	POS	POS	NEG	NEG	NEG	NEG	NEG	POS	POS	POS	NEG	POS	POS	POS	NEG	AMB	NEG	AMB	POS	POS	AMB	POS	AMB	POS	POS	POS	NEG	NEG	NEG	POS	POS	POS	POS	NEG	POS
Iasi-20-P1	Carrier	POS	NEG	POS	NEG	POS	NEG	AMB	NEG	NEG	POS	POS	POS	POS	NEG	NEG	NEG	NEG	NEG	POS	POS	POS	NEG	POS	POS	POS	NEG	AMB	NEG	NEG	POS	POS	AMB	AMB	POS	POS	POS	POS	NEG	NEG	NEG	POS	POS	POS	POS	NEG	POS
Iasi-35-P24	Carrier	POS	NEG	POS	NEG	POS	NEG	AMB	NEG	NEG	POS	POS	POS	POS	NEG	NEG	NEG	NEG	NEG	POS	POS	POS	NEG	POS	POS	POS	NEG	NEG	NEG	NEG	POS	POS	AMB	AMB	POS	POS	POS	POS	NEG	NEG	NEG	POS	POS	POS	POS	NEG	POS
Iasi-36-P25	Carrier	POS	NEG	POS	NEG	POS	AMB	AMB	AMB	NEG	POS	POS	POS	POS	NEG	NEG	NEG	NEG	NEG	POS	POS	POS	NEG	POS	POS	POS	NEG	NEG	NEG	NEG	POS	POS	AMB	AMB	POS	POS	POS	POS	NEG	NEG	NEG	POS	POS	POS	POS	NEG	POS
Iasi-14-150	SSTI	POS	NEG	POS	NEG	POS	NEG	NEG	NEG	NEG	POS	POS	POS	POS	NEG	NEG	NEG	NEG	NEG	POS	POS	POS	NEG	POS	POS	POS	NEG	AMB	NEG	NEG	POS	POS	POS	AMB	AMB	POS	POS	POS	NEG	NEG	NEG	POS	POS	POS	POS	NEG	POS
Iasi-07-54	BSI	POS	NEG	POS	NEG	POS	NEG	NEG	NEG	NEG	NEG	NEG	NEG	NEG	POS	POS	POS	POS	POS	POS	POS	POS	NEG	POS	POS	POS	NEG	NEG	NEG	AMB	POS	POS	POS	AMB	AMB	POS	POS	NEG	NEG	POS	POS	POS	POS	POS	POS	NEG	POS
Iasi-16-188	BSI	POS	NEG	POS	NEG	POS	NEG	NEG	NEG	NEG	NEG	NEG	NEG	NEG	POS	POS	POS	POS	POS	POS	POS	POS	NEG	POS	POS	POS	NEG	NEG	NEG	NEG	POS	POS	POS	AMB	AMB	POS	POS	NEG	NEG	POS	POS	POS	POS	POS	POS	NEG	POS
Iasi-32-318	BSI	POS	NEG	POS	NEG	POS	NEG	NEG	NEG	NEG	NEG	NEG	NEG	NEG	POS	POS	POS	POS	POS	POS	POS	POS	NEG	POS	POS	POS	NEG	NEG	NEG	NEG	POS	POS	POS	AMB	AMB	POS	POS	NEG	NEG	NEG	POS	POS	POS	POS	POS	NEG	POS
Iasi-42-397	BSI	POS	NEG	POS	NEG	POS	NEG	NEG	NEG	NEG	NEG	NEG	NEG	NEG	POS	POS	POS	POS	POS	POS	POS	POS	NEG	POS	POS	POS	NEG	NEG	NEG	NEG	POS	POS	POS	AMB	AMB	POS	POS	NEG	NEG	NEG	POS	POS	POS	POS	POS	NEG	POS
Iasi-48-408	BSI	POS	NEG	POS	NEG	POS	NEG	NEG	NEG	NEG	NEG	NEG	NEG	NEG	POS	POS	POS	POS	POS	POS	POS	POS	NEG	POS	POS	POS	NEG	NEG	NEG	NEG	POS	POS	POS	AMB	AMB	POS	POS	NEG	NEG	POS	POS	POS	POS	POS	POS	NEG	POS
Iasi-49-430	BSI	POS	NEG	POS	NEG	POS	NEG	NEG	NEG	NEG	NEG	NEG	NEG	NEG	POS	POS	POS	POS	POS	POS	POS	POS	NEG	POS	POS	POS	NEG	NEG	NEG	NEG	POS	POS	AMB	AMB	POS	POS	POS	NEG	NEG	POS	POS	POS	POS	POS	POS	NEG	POS
Iasi-52-52	BSI	POS	NEG	POS	NEG	POS	NEG	NEG	NEG	NEG	NEG	NEG	NEG	NEG	POS	POS	POS	POS	POS	POS	POS	POS	NEG	POS	POS	POS	NEG	NEG	NEG	NEG	POS	POS	POS	AMB	AMB	POS	POS	NEG	NEG	POS	POS	POS	POS	POS	POS	NEG	POS
Iasi-01-2	SSTI	POS	NEG	POS	NEG	POS	NEG	NEG	NEG	NEG	NEG	NEG	NEG	NEG	POS	POS	POS	POS	POS	POS	POS	POS	NEG	POS	POS	POS	NEG	AMB	NEG	NEG	POS	POS	POS	AMB	AMB	POS	POS	NEG	NEG	POS	POS	POS	POS	POS	POS	NEG	POS
Iasi-10-101	SSTI	POS	NEG	POS	NEG	POS	NEG	NEG	NEG	NEG	NEG	NEG	NEG	NEG	POS	POS	POS	POS	POS	POS	POS	POS	NEG	POS	POS	POS	NEG	NEG	NEG	NEG	POS	POS	POS	AMB	AMB	POS	POS	NEG	NEG	NEG	POS	POS	POS	POS	POS	NEG	POS
Iasi-11-106	SSTI	POS	NEG	POS	NEG	POS	NEG	NEG	NEG	NEG	NEG	NEG	NEG	NEG	POS	POS	POS	POS	POS	POS	POS	POS	NEG	POS	POS	POS	NEG	AMB	NEG	NEG	POS	POS	AMB	AMB	POS	POS	POS	NEG	NEG	POS	POS	POS	POS	POS	POS	NEG	POS
Iasi-49-414	SSTI	POS	NEG	POS	NEG	POS	NEG	NEG	NEG	NEG	NEG	NEG	NEG	NEG	POS	POS	POS	POS	POS	POS	POS	POS	NEG	POS	POS	POS	NEG	NEG	NEG	AMB	POS	POS	AMB	AMB	POS	POS	POS	NEG	NEG	POS	POS	POS	POS	POS	POS	NEG	POS

Isolate	Group	VIRULENCE : STAPHYLOCOCCAL SUPERANTIGEN/ENTEROTOXIN-LIKE GENES (SET/SSL)					CAPSULE- AND BIOFILM-ASSOCIATED GENES							
		staphylococcal exotoxin-like protein, second locus					Capsule type 1	capsular poly-saccharide synthesis enzyme	O-antigen poly-merase	capsular poly-saccharide biosyn-thesis protein	Capsule type 5	capsular poly-saccharide synthesis enzyme	O-antigen poly-merase	capsular poly-saccharide biosyn-thesis protein
		setB3	setB3 (MRSA252)	setB2	setB2 (MRSA252)	setB1	cap 1 (total)	capH1	capJ1	capK1	cap 5 (total)	capH5	capJ5	capK5
Iasi-05-46	misc. MRSA	POS	NEG	POS	NEG	POS	NEG	NEG	NEG	NEG	NEG	NEG	NEG	NEG
Iasi-06-47	misc. MRSA	POS	NEG	POS	NEG	POS	NEG	NEG	NEG	NEG	NEG	NEG	NEG	NEG
Iasi-08-73	misc. MRSA	POS	NEG	POS	NEG	POS	NEG	NEG	NEG	NEG	NEG	NEG	NEG	NEG
Iasi-19-194	misc. MRSA	POS	NEG	POS	NEG	POS	NEG	NEG	NEG	NEG	NEG	NEG	NEG	NEG
Iasi-21-219	misc. MRSA	POS	NEG	POS	NEG	POS	NEG	NEG	NEG	NEG	NEG	NEG	NEG	NEG
Iasi-25-284	misc. MRSA	POS	NEG	POS	NEG	POS	NEG	NEG	NEG	NEG	NEG	NEG	NEG	NEG
Iasi-284	misc. MRSA	POS	NEG	POS	NEG	POS	NEG	NEG	NEG	NEG	NEG	NEG	NEG	NEG
Iasi-36-336	misc. MRSA	POS	NEG	POS	NEG	POS	NEG	NEG	NEG	NEG	NEG	NEG	NEG	NEG
Iasi-43-402	misc. MRSA	POS	NEG	POS	NEG	POS	NEG	NEG	NEG	NEG	NEG	NEG	NEG	NEG
Iasi-44-404	misc. MRSA	POS	NEG	POS	NEG	POS	NEG	NEG	NEG	NEG	NEG	NEG	NEG	NEG
Iasi-54-430	misc. MRSA	POS	NEG	POS	NEG	POS	NEG	NEG	NEG	AMB	NEG	NEG	NEG	NEG
Iasi-24-216	SSTI	NEG	POS	NEG	POS	AMB	NEG	NEG	NEG	NEG	POS	POS	POS	POS
Iasi-05-S13	Carrier	NEG	POS	NEG	POS	POS	NEG	NEG	NEG	NEG	POS	POS	POS	POS
Iasi-45-362	BSI	POS	NEG	POS	NEG	POS	NEG	NEG	NEG	NEG	NEG	NEG	NEG	NEG
Iasi-22-199	SSTI	POS	NEG	POS	NEG	POS	NEG	NEG	NEG	NEG	NEG	NEG	NEG	NEG
Iasi-12-S26	Carrier	POS	NEG	POS	NEG	POS	NEG	NEG	NEG	NEG	NEG	NEG	NEG	NEG
Iasi-21-P2	Carrier	POS	NEG	POS	NEG	POS	NEG	POS	NEG	NEG	NEG	NEG	NEG	NEG
Iasi-06-60	BSI	NEG	NEG	NEG	NEG	AMB	NEG	NEG	AMB	NEG	POS	POS	POS	POS
Iasi-12-79	BSI	NEG	NEG	NEG	NEG	NEG	NEG	NEG	NEG	NEG	POS	POS	POS	POS
Iasi-17-90	BSI	NEG	NEG	NEG	NEG	NEG	NEG	NEG	NEG	NEG	POS	POS	POS	POS
Iasi-21-105	BSI	NEG	NEG	NEG	NEG	NEG	NEG	NEG	NEG	NEG	POS	POS	POS	POS
Iasi-22-109	BSI	NEG	NEG	NEG	NEG	POS	NEG	NEG	POS	NEG	POS	POS	POS	POS
Iasi-23-110	BSI	NEG	NEG	NEG	NEG	AMB	NEG	NEG	AMB	NEG	POS	POS	POS	POS
Iasi-24-136	BSI	NEG	NEG	NEG	NEG	NEG	NEG	NEG	NEG	NEG	POS	POS	POS	POS
Iasi-34-224	BSI	NEG	NEG	NEG	NEG	NEG	NEG	NEG	AMB	NEG	POS	POS	POS	POS
Iasi-39-297	BSI	NEG	NEG	NEG	NEG	POS	NEG	NEG	AMB	NEG	POS	POS	POS	POS
Iasi-50-431	BSI	NEG	NEG	NEG	NEG	NEG	NEG	NEG	NEG	NEG	POS	POS	POS	POS
Iasi-51-432	BSI	NEG	NEG	NEG	NEG	AMB	NEG	NEG	AMB	NEG	POS	POS	POS	POS
Iasi-08-76	SSTI	NEG	NEG	NEG	NEG	NEG	NEG	NEG	NEG	NEG	POS	POS	POS	POS
Iasi-30-350	SSTI	NEG	NEG	NEG	NEG	AMB	NEG	NEG	NEG	NEG	POS	POS	POS	POS
Iasi-33-319	SSTI	NEG	NEG	NEG	NEG	NEG	NEG	NEG	NEG	NEG	POS	POS	POS	POS
Iasi-40-383	SSTI	NEG	NEG	NEG	NEG	NEG	NEG	NEG	NEG	NEG	POS	POS	POS	POS
Iasi-49-423	SSTI	NEG	NEG	NEG	NEG	NEG	NEG	NEG	NEG	NEG	POS	POS	POS	POS
Iasi-16-S31	Carrier	NEG	NEG	NEG	NEG	AMB	NEG	NEG	NEG	NEG	POS	POS	POS	POS
Iasi-24-P9	Carrier	NEG	NEG	NEG	NEG	POS	NEG	NEG	AMB	NEG	POS	POS	POS	POS
Iasi-27-P12	Carrier	NEG	NEG	NEG	NEG	NEG	NEG	NEG	NEG	NEG	POS	POS	POS	POS
Iasi-38-P27	Carrier	NEG	NEG	NEG	NEG	POS	NEG	NEG	AMB	NEG	POS	POS	POS	POS
Iasi-42-G511	Carrier	NEG	NEG	NEG	NEG	NEG	NEG	NEG	NEG	NEG	POS	POS	POS	POS
Iasi-46-3N	Carrier	NEG	NEG	NEG	NEG	POS	NEG	NEG	AMB	NEG	POS	POS	POS	POS
Iasi-47-42O	Carrier	NEG	NEG	NEG	NEG	AMB	NEG	NEG	AMB	NEG	POS	POS	POS	POS
Iasi-48-36N	Carrier	NEG	NEG	NEG	NEG	AMB	NEG	NEG	AMB	NEG	POS	POS	POS	POS
Iasi-49-T3	Carrier	NEG	NEG	NEG	NEG	NEG	NEG	NEG	NEG	NEG	POS	POS	POS	POS
Iasi-50-T2	Carrier	NEG	NEG	NEG	NEG	AMB	NEG	NEG	AMB	NEG	POS	POS	POS	POS
Iasi-22-251	BSI	NEG	NEG	NEG	NEG	NEG	NEG	NEG	NEG	NEG	POS	POS	POS	POS
Iasi-27-158	BSI	POS	NEG	POS	NEG	POS	AMB	AMB	AMB	NEG	POS	POS	POS	POS
Iasi-08-70	BSI	NEG	POS	NEG	POS	POS	NEG	NEG	NEG	NEG	NEG	NEG	NEG	NEG
Iasi-18-93	BSI	NEG	POS	NEG	POS	POS	NEG	NEG	NEG	NEG	NEG	NEG	NEG	NEG
Iasi-28-P14	Carrier	NEG	POS	NEG	POS	POS	NEG	NEG	NEG	NEG	NEG	NEG	NEG	NEG
Iasi-29-P15	Carrier	NEG	POS	NEG	POS	POS	NEG	AMB	NEG	AMB	NEG	NEG	NEG	NEG
Iasi-03-19	BSI	NEG	POS	NEG	NEG	NEG	NEG	NEG	NEG	NEG	NEG	NEG	NEG	NEG
Iasi-11-77	BSI	NEG	POS	NEG	NEG	NEG	NEG	NEG	NEG	NEG	NEG	NEG	NEG	NEG
Iasi-13-82	BSI	NEG	POS	NEG	NEG	NEG	NEG	NEG	NEG	NEG	NEG	NEG	NEG	NEG
Iasi-15-86	BSI	NEG	POS	NEG	AMB	NEG	NEG	NEG	NEG	NEG	NEG	NEG	NEG	NEG
Iasi-20-103	BSI	NEG	POS	NEG	AMB	NEG	NEG	NEG	NEG	NEG	NEG	NEG	NEG	NEG
Iasi-33-222	BSI	NEG	POS	NEG	AMB	NEG	NEG	AMB	NEG	NEG	NEG	NEG	NEG	NEG
Iasi-44-360	BSI	NEG	POS	NEG	POS	NEG	NEG	NEG	NEG	NEG	NEG	NEG	NEG	NEG
Iasi-47-399	BSI	NEG	POS	NEG	AMB	NEG	NEG	NEG	NEG	AMB	NEG	NEG	NEG	NEG
Iasi-34-368	SSTI	NEG	POS	NEG	POS	NEG	NEG	NEG	NEG	NEG	NEG	NEG	NEG	NEG
Iasi-36-371	SSTI	NEG	POS	NEG	AMB	AMB	NEG	NEG	NEG	NEG	NEG	NEG	NEG	NEG
Iasi-04-S12	Carrier	NEG	POS	NEG	POS	POS	NEG	AMB	NEG	NEG	NEG	NEG	NEG	NEG
Iasi-06-S15	Carrier	NEG	POS	NEG	POS	POS	NEG	NEG	NEG	NEG	NEG	NEG	NEG	NEG
Iasi-07-S16	Carrier	NEG	POS	NEG	POS	POS	NEG	NEG	NEG	NEG	NEG	NEG	NEG	NEG
Iasi-11-S24	Carrier	NEG	POS	NEG	POS	AMB	AMB	POS	NEG	AMB	NEG	NEG	NEG	NEG
Iasi-22-P4	Carrier	NEG	POS	NEG	POS	AMB	NEG	NEG	NEG	NEG	NEG	NEG	NEG	NEG
Iasi-34-P23	Carrier	NEG	POS	NEG	POS	AMB	NEG	NEG	NEG	NEG	NEG	NEG	NEG	NEG
Iasi-37-P26	Carrier	NEG	POS	NEG	POS	AMB	NEG	NEG	NEG	AMB	NEG	NEG	NEG	NEG
Iasi-30-196	BSI	NEG	POS	NEG	POS	POS	AMB	AMB	NEG	POS	NEG	NEG	NEG	AMB
Iasi-27-298	BSI	POS	NEG	POS	NEG	POS	NEG	NEG	NEG	NEG	NEG	NEG	NEG	NEG
Iasi-37-363	BSI	POS	NEG	POS	NEG	POS	NEG	NEG	NEG	NEG	NEG	NEG	NEG	NEG
Iasi-38-372	BSI	POS	NEG	POS	NEG	POS	NEG	NEG	NEG	NEG	NEG	NEG	NEG	NEG
Iasi-39-374	BSI	POS	NEG	POS	NEG	POS	NEG	NEG	NEG	NEG	NEG	NEG	NEG	NEG
Iasi-28-299	SSTI	POS	NEG	POS	NEG	POS	NEG	NEG	NEG	NEG	NEG	NEG	NEG	NEG
Iasi-07-65	BSI	POS	NEG	POS	NEG	POS	NEG	NEG	NEG	NEG	NEG	NEG	NEG	NEG
Iasi-19-99	BSI	POS	NEG	POS	NEG	POS	NEG	NEG	NEG	NEG	POS	POS	POS	POS
Iasi-25-217	SSTI	POS	NEG	POS	NEG	POS	NEG	NEG	NEG	NEG	POS	POS	POS	POS
Iasi-23-P5	Carrier	POS	NEG	POS	NEG	POS	NEG	AMB	NEG	NEG	POS	POS	POS	POS
Iasi-43-G513	Carrier	POS	NEG	POS	NEG	POS	NEG	NEG	NEG	NEG	POS	POS	POS	POS
Iasi-44-G514	Carrier	POS	NEG	POS	NEG	POS	AMB	AMB	AMB	NEG	POS	POS	POS	POS
Iasi-28-172	BSI	POS	NEG	POS	NEG	POS	AMB	AMB	NEG	AMB	NEG	NEG	NEG	AMB
Iasi-42-358	BSI	POS	NEG	POS	NEG	POS	AMB	AMB	NEG	AMB	NEG	NEG	NEG	NEG
Iasi-48-403	BSI	POS	NEG	POS	NEG	POS	NEG	NEG	NEG	AMB	NEG	NEG	NEG	NEG
Iasi-26-153	BSI	POS	NEG	POS	NEG	POS	NEG	NEG	NEG	NEG	NEG	NEG	NEG	NEG
Iasi-29-190	BSI	POS	NEG	POS	NEG	POS	NEG	NEG	NEG	AMB	NEG	NEG	NEG	NEG
Iasi-31-201	BSI	POS	NEG	POS	NEG	POS	NEG	NEG	NEG	NEG	NEG	NEG	NEG	NEG
Iasi-37-289	BSI	POS	NEG	POS	NEG	POS	NEG	NEG	NEG	AMB	NEG	NEG	NEG	NEG
Iasi-41-334	BSI	POS	NEG	POS	NEG	POS	AMB	AMB	NEG	AMB	NEG	NEG	NEG	NEG
Iasi-52-433	BSI	POS	NEG	POS	NEG	POS	NEG	NEG	NEG	NEG	NEG	NEG	NEG	NEG
Iasi-07-66	SSTI	POS	NEG	POS	NEG	POS	NEG	NEG	NEG	NEG	NEG	NEG	NEG	NEG
Iasi-23-211	SSTI	POS	NEG	POS	NEG	POS	NEG	NEG	NEG	NEG	NEG	NEG	NEG	NEG
Iasi-43-401	SSTI	POS	NEG	POS	NEG	POS	NEG	NEG	NEG	NEG	NEG	NEG	NEG	NEG
Iasi-47-418	SSTI	POS	NEG	POS	NEG	POS	NEG	NEG	NEG	NEG	NEG	NEG	NEG	NEG
Iasi-18-169	SSTI	POS	NEG	POS	NEG	POS	NEG	NEG	NEG	NEG	NEG	NEG	NEG	NEG
Iasi-18-S34	Carrier	POS	NEG	POS	NEG	POS	AMB	AMB	NEG	AMB	NEG	NEG	NEG	NEG
Iasi-36-276	BSI	NEG	POS	NEG	POS	POS	NEG	AMB	NEG	NEG	POS	POS	POS	POS
Iasi-151	BSI	NEG	POS	NEG	POS	POS	NEG	NEG	AMB	NEG	POS	POS	POS	POS
Iasi-02-11	BSI	POS	NEG	POS	NEG	POS	NEG	AMB	NEG	NEG	POS	AMB	POS	POS

Isolate	CAPSULE- AND BIOFILM-ASSOCIATED GENES									ADHAESION FACTORS / GENES ENCODING MICROBIAL SURFACE COMPONENTS RECOGNIZING ADHESIVE MATRIX MOLECULES (MSCRAMM GENES)						
	Capsule type 8	capsular poly-saccharide synthesis enzyme	capsular poly-saccharide biosyn-thesis protein	O-antigen poly-merase	capsular poly-saccharide biosyn-thesis protein	intercellular adhesion protein A	intercellular adhesion protein C	biofilm PIA synthesis protein D	surface protein involved in biofilm formation	bone sialoprotein-binding protein						
	cap 8 (total)	capH8	capI8	capJ8	capK8	icaA	icaC	icaD	bap	bbp (total)	bbp (consensus)	bbp (COL+MW2)	bbp (MRSA252)	bbp (Mu50)	bbp (RF122)	bbp (ST45)
Iasi-05-46	POS	POS	POS	POS	POS	POS	POS	POS	NEG	POS	POS	POS	NEG	NEG	NEG	NEG
Iasi-06-47	POS	POS	POS	POS	POS	POS	POS	POS	NEG	POS	POS	POS	NEG	NEG	NEG	NEG
Iasi-08-73	POS	POS	POS	POS	POS	POS	POS	POS	NEG	POS	POS	POS	NEG	NEG	NEG	NEG
Iasi-19-194	POS	POS	POS	POS	POS	POS	POS	POS	NEG	POS	POS	POS	NEG	NEG	NEG	NEG
Iasi-21-219	POS	POS	POS	POS	POS	POS	POS	POS	NEG	POS	POS	POS	NEG	NEG	NEG	NEG
Iasi-25-284	POS	POS	POS	POS	POS	POS	POS	POS	NEG	POS	POS	POS	NEG	NEG	NEG	NEG
Iasi-284	POS	POS	POS	POS	POS	POS	POS	POS	NEG	POS	POS	POS	NEG	NEG	NEG	NEG
Iasi-36-336	POS	POS	POS	POS	POS	POS	POS	POS	NEG	POS	POS	POS	NEG	NEG	NEG	NEG
Iasi-43-402	POS	POS	POS	POS	POS	POS	POS	POS	NEG	POS	POS	POS	NEG	NEG	NEG	NEG
Iasi-44-404	POS	POS	POS	POS	POS	POS	POS	POS	NEG	POS	POS	POS	NEG	NEG	NEG	NEG
Iasi-54-430	POS	POS	POS	POS	POS	POS	POS	POS	NEG	POS	POS	POS	NEG	AMB	NEG	NEG
Iasi-24-216	NEG	NEG	NEG	NEG	NEG	POS	POS	POS	NEG	NEG	NEG	NEG	NEG	NEG	NEG	NEG
Iasi-05-S13	NEG	NEG	NEG	NEG	NEG	POS	POS	POS	NEG	NEG	NEG	NEG	NEG	NEG	NEG	NEG
Iasi-45-362	POS	POS	POS	POS	POS	POS	POS	POS	NEG	POS	POS	NEG	NEG	POS	NEG	NEG
Iasi-22-199	POS	POS	POS	POS	POS	POS	POS	POS	NEG	POS	POS	NEG	NEG	POS	NEG	NEG
Iasi-12-S26	POS	POS	POS	POS	POS	POS	POS	POS	NEG	POS	POS	AMB	NEG	POS	NEG	NEG
Iasi-21-P2	POS	POS	POS	POS	POS	POS	POS	POS	NEG	POS	POS	AMB	NEG	POS	NEG	NEG
Iasi-06-60	NEG	NEG	NEG	NEG	NEG	POS	POS	POS	NEG	POS	POS	NEG	NEG	POS	NEG	NEG
Iasi-12-79	NEG	NEG	NEG	NEG	NEG	POS	POS	POS	NEG	POS	POS	NEG	NEG	POS	NEG	NEG
Iasi-17-90	NEG	NEG	NEG	NEG	NEG	POS	POS	POS	NEG	POS	POS	NEG	NEG	NEG	NEG	NEG
Iasi-21-105	NEG	NEG	NEG	NEG	NEG	POS	POS	POS	NEG	POS	POS	NEG	NEG	NEG	NEG	NEG
Iasi-22-109	NEG	NEG	NEG	NEG	NEG	POS	POS	POS	NEG	POS	POS	NEG	NEG	POS	NEG	AMB
Iasi-23-110	NEG	NEG	NEG	NEG	NEG	POS	POS	POS	NEG	POS	POS	NEG	NEG	POS	NEG	NEG
Iasi-24-136	NEG	NEG	NEG	NEG	NEG	POS	POS	POS	NEG	POS	POS	NEG	NEG	POS	NEG	NEG
Iasi-34-224	NEG	NEG	NEG	NEG	NEG	POS	POS	POS	NEG	POS	POS	NEG	NEG	POS	NEG	NEG
Iasi-39-297	NEG	NEG	NEG	NEG	NEG	POS	POS	POS	NEG	POS	POS	NEG	NEG	POS	NEG	NEG
Iasi-50-431	NEG	NEG	NEG	NEG	NEG	POS	POS	POS	NEG	POS	POS	NEG	NEG	NEG	NEG	NEG
Iasi-51-432	NEG	NEG	NEG	NEG	NEG	POS	POS	POS	NEG	POS	POS	NEG	NEG	POS	NEG	NEG
Iasi-08-76	NEG	NEG	NEG	NEG	NEG	POS	POS	POS	NEG	POS	POS	NEG	NEG	NEG	NEG	NEG
Iasi-30-350	NEG	NEG	NEG	NEG	NEG	POS	POS	POS	NEG	POS	POS	NEG	NEG	POS	NEG	NEG
Iasi-33-319	NEG	NEG	NEG	NEG	NEG	POS	AMB	POS	NEG	POS	POS	NEG	NEG	NEG	NEG	NEG
Iasi-40-383	NEG	NEG	NEG	NEG	NEG	POS	POS	POS	NEG	POS	POS	NEG	NEG	POS	NEG	NEG
Iasi-49-423	NEG	NEG	NEG	NEG	NEG	POS	POS	POS	NEG	POS	POS	NEG	NEG	NEG	NEG	NEG
Iasi-16-S31	NEG	NEG	NEG	NEG	NEG	POS	POS	POS	NEG	POS	POS	NEG	NEG	POS	NEG	AMB
Iasi-24-P9	NEG	NEG	NEG	NEG	NEG	POS	POS	POS	NEG	POS	POS	NEG	NEG	POS	NEG	NEG
Iasi-27-P12	NEG	NEG	NEG	NEG	NEG	POS	POS	POS	NEG	NEG	NEG	NEG	NEG	NEG	NEG	NEG
Iasi-38-P27	NEG	NEG	NEG	NEG	NEG	POS	POS	POS	NEG	POS	POS	NEG	NEG	POS	NEG	NEG
Iasi-42-G511	NEG	NEG	NEG	NEG	NEG	POS	POS	POS	NEG	POS	POS	NEG	NEG	POS	NEG	NEG
Iasi-46-3N	NEG	NEG	NEG	NEG	NEG	POS	POS	POS	NEG	POS	POS	NEG	NEG	POS	NEG	NEG
Iasi-47-42O	NEG	NEG	NEG	NEG	NEG	POS	POS	POS	NEG	POS	POS	NEG	NEG	POS	NEG	NEG
Iasi-48-36N	NEG	NEG	NEG	NEG	NEG	POS	POS	POS	NEG	POS	POS	NEG	NEG	POS	NEG	NEG
Iasi-49-T3	NEG	NEG	NEG	NEG	NEG	POS	POS	POS	NEG	POS	POS	NEG	NEG	POS	NEG	NEG
Iasi-50-T2	NEG	NEG	NEG	NEG	NEG	POS	POS	POS	NEG	POS	POS	NEG	NEG	POS	NEG	NEG
Iasi-22-251	NEG	NEG	NEG	NEG	NEG	POS	POS	POS	NEG	POS	POS	NEG	NEG	POS	NEG	NEG
Iasi-27-158	NEG	NEG	NEG	NEG	NEG	POS	POS	POS	NEG	POS	POS	POS	NEG	AMB	NEG	NEG
Iasi-08-70	POS	POS	POS	POS	POS	POS	POS	POS	NEG	POS	POS	AMB	POS	NEG	NEG	NEG
Iasi-18-93	POS	POS	POS	POS	POS	POS	POS	POS	NEG	POS	POS	AMB	POS	NEG	NEG	NEG
Iasi-28-P14	POS	POS	POS	POS	POS	POS	POS	POS	NEG	POS	POS	NEG	POS	NEG	NEG	NEG
Iasi-29-P15	POS	POS	POS	POS	POS	POS	POS	POS	NEG	POS	POS	AMB	POS	NEG	NEG	NEG
Iasi-03-19	POS	POS	POS	POS	POS	POS	POS	POS	NEG	POS	POS	NEG	NEG	NEG	NEG	POS
Iasi-11-77	POS	POS	POS	POS	POS	POS	POS	POS	NEG	POS	POS	NEG	NEG	NEG	NEG	POS
Iasi-13-82	POS	POS	POS	POS	POS	POS	POS	POS	NEG	POS	POS	NEG	NEG	NEG	NEG	POS
Iasi-15-86	POS	POS	POS	POS	POS	POS	POS	POS	NEG	POS	POS	AMB	NEG	AMB	NEG	POS
Iasi-20-103	POS	POS	POS	POS	POS	POS	POS	POS	NEG	POS	POS	NEG	NEG	NEG	NEG	POS
Iasi-33-222	POS	POS	POS	POS	POS	POS	POS	POS	NEG	POS	POS	AMB	NEG	NEG	NEG	POS
Iasi-44-360	POS	POS	POS	POS	POS	POS	POS	POS	NEG	POS	POS	NEG	NEG	AMB	NEG	POS
Iasi-47-399	POS	POS	POS	POS	POS	POS	POS	POS	NEG	POS	POS	AMB	NEG	NEG	NEG	POS
Iasi-34-368	POS	POS	POS	POS	POS	POS	POS	POS	NEG	POS	POS	NEG	NEG	AMB	NEG	POS
Iasi-36-371	POS	POS	POS	POS	POS	POS	POS	POS	NEG	NEG	NEG	NEG	NEG	NEG	NEG	NEG
Iasi-04-S12	POS	POS	POS	POS	POS	POS	POS	POS	NEG	POS	POS	AMB	NEG	AMB	NEG	POS
Iasi-06-S15	POS	POS	POS	POS	POS	POS	POS	POS	NEG	POS	POS	NEG	NEG	AMB	NEG	POS
Iasi-07-S16	POS	POS	POS	POS	POS	POS	POS	POS	NEG	POS	POS	NEG	NEG	AMB	NEG	POS
Iasi-11-S24	POS	POS	POS	POS	POS	POS	POS	POS	NEG	POS	POS	AMB	NEG	AMB	NEG	POS
Iasi-22-P4	POS	POS	POS	POS	POS	POS	POS	POS	NEG	POS	POS	NEG	NEG	AMB	NEG	POS
Iasi-34-P23	POS	POS	POS	POS	POS	POS	POS	POS	NEG	POS	POS	AMB	NEG	AMB	NEG	POS
Iasi-37-P26	POS	POS	POS	POS	POS	POS	POS	POS	NEG	POS	POS	AMB	NEG	AMB	NEG	POS
Iasi-30-196	POS	POS	POS	POS	POS	POS	POS	POS	NEG	POS	POS	AMB	NEG	AMB	AMB	POS
Iasi-27-298	POS	POS	POS	POS	POS	POS	POS	POS	NEG	POS	POS	NEG	NEG	POS	NEG	NEG
Iasi-37-363	POS	POS	POS	POS	POS	POS	POS	POS	NEG	POS	POS	NEG	NEG	POS	NEG	NEG
Iasi-38-372	POS	POS	POS	POS	POS	POS	POS	POS	NEG	POS	POS	NEG	NEG	POS	NEG	NEG
Iasi-39-374	POS	POS	POS	POS	POS	POS	POS	POS	NEG	POS	POS	AMB	NEG	POS	NEG	NEG
Iasi-28-299	POS	POS	POS	POS	POS	POS	POS	POS	NEG	POS	POS	NEG	NEG	POS	NEG	NEG
Iasi-07-65	POS	POS	POS	POS	POS	POS	POS	POS	NEG	POS	POS	NEG	NEG	POS	NEG	NEG
Iasi-19-99	NEG	NEG	NEG	NEG	NEG	POS	POS	POS	NEG	POS	POS	NEG	NEG	POS	NEG	NEG
Iasi-25-217	NEG	NEG	NEG	NEG	NEG	POS	POS	POS	NEG	NEG	NEG	NEG	NEG	NEG	NEG	NEG
Iasi-23-P5	NEG	NEG	NEG	NEG	NEG	POS	POS	POS	NEG	POS	POS	NEG	NEG	POS	NEG	NEG
Iasi-43-G513	NEG	NEG	NEG	NEG	NEG	POS	POS	POS	NEG	NEG	NEG	NEG	NEG	NEG	NEG	NEG
Iasi-44-G514	NEG	NEG	NEG	NEG	NEG	POS	POS	POS	NEG	NEG	NEG	NEG	NEG	NEG	NEG	NEG
Iasi-28-172	POS	POS	POS	POS	POS	POS	POS	POS	NEG	POS	POS	AMB	POS	NEG	NEG	NEG
Iasi-42-358	POS	POS	POS	POS	POS	POS	POS	POS	NEG	POS	POS	AMB	POS	NEG	NEG	NEG
Iasi-48-403	POS	POS	POS	POS	POS	POS	POS	POS	NEG	POS	POS	AMB	POS	NEG	NEG	NEG
Iasi-26-153	POS	POS	POS	POS	POS	POS	POS	POS	NEG	POS	POS	NEG	POS	NEG	NEG	NEG
Iasi-29-190	POS	POS	POS	POS	POS	POS	POS	POS	NEG	POS	POS	NEG	POS	NEG	NEG	NEG
Iasi-31-201	POS	POS	POS	POS	POS	POS	POS	POS	NEG	POS	POS	NEG	POS	NEG	NEG	NEG
Iasi-37-289	POS	POS	POS	POS	POS	POS	POS	POS	NEG	POS	POS	AMB	POS	NEG	NEG	NEG
Iasi-41-334	POS	POS	POS	POS	POS	POS	POS	POS	NEG	POS	POS	AMB	POS	NEG	NEG	AMB
Iasi-52-433	POS	POS	POS	POS	POS	POS	POS	POS	NEG	POS	POS	NEG	POS	NEG	NEG	NEG
Iasi-07-66	POS	POS	POS	POS	POS	POS	POS	POS	NEG	POS	POS	NEG	POS	NEG	NEG	NEG
Iasi-23-211	POS	POS	POS	POS	POS	POS	POS	POS	NEG	POS	POS	NEG	POS	NEG	NEG	NEG
Iasi-43-401	POS	POS	POS	POS	POS	POS	POS	POS	NEG	POS	POS	NEG	POS	NEG	NEG	NEG
Iasi-47-418	POS	POS	POS	POS	POS	POS	POS	POS	NEG	POS	POS	NEG	POS	NEG	NEG	NEG
Iasi-18-169	POS	POS	POS	POS	POS	POS	POS	POS	NEG	POS	POS	NEG	NEG	POS	NEG	NEG
Iasi-18-S34	POS	POS	POS	POS	POS	POS	POS	POS	NEG	POS	POS	POS	NEG	AMB	NEG	AMB
Iasi-36-276	NEG	NEG	NEG	NEG	NEG	POS	POS	POS	NEG	POS	POS	AMB	NEG	NEG	NEG	POS
Iasi-151	NEG	NEG	NEG	NEG	NEG	POS	POS	POS	NEG	POS	POS	AMB	NEG	AMB	NEG	POS
Iasi-02-11	NEG	NEG	NEG	NEG	NEG	POS	POS	POS	NEG	POS	POS	POS	NEG	NEG	NEG	NEG

Isolate	ADHAESION FACTORS / GENES ENCODING MICROBIAL SURFACE COMPONENTS RECOGNIZING ADHESIVE MATRIX MOLECULES (MSCRAMM GENES)																
	clumping factor A					clumping factor B					collagen-binding adhesin	cell wall associated fibronectin-binding protein	cell surface elastin binding protein				
	clfA (total)	clfA (consensus)	clfA (COL+RF122)	clfA (MRSA252)	clfA (Mu50+MW2)	clfB (total)	clfB (consensus)	clfB (COL+Mu50)	clfB (MW2)	clfB (RF122)	cna	ebh (consensus)	ebpS (total)	ebpS_probe 612	ebpS_probe 614	ebpS (01-1111)	ebpS (COL)
Iasi-05-46	POS	POS	POS	AMB	AMB	POS	POS	NEG	NEG	NEG	POS	POS	POS	POS	POS	NEG	POS
Iasi-06-47	POS	POS	POS	AMB	AMB	POS	POS	NEG	NEG	POS	POS	POS	POS	POS	POS	NEG	POS
Iasi-08-73	POS	POS	POS	AMB	AMB	POS	POS	NEG	NEG	POS	POS	POS	POS	POS	POS	NEG	POS
Iasi-19-194	POS	POS	AMB	AMB	POS	POS	POS	NEG	NEG	NEG	POS	POS	POS	POS	POS	NEG	POS
Iasi-21-219	POS	POS	POS	AMB	AMB	POS	POS	NEG	NEG	POS	POS	POS	POS	POS	POS	NEG	POS
Iasi-25-284	POS	POS	POS	AMB	POS	POS	POS	NEG	NEG	POS	POS	POS	POS	POS	POS	NEG	POS
Iasi-284	POS	POS	POS	AMB	AMB	POS	POS	NEG	NEG	POS	POS	POS	POS	POS	POS	NEG	POS
Iasi-36-336	POS	POS	POS	AMB	AMB	POS	POS	NEG	NEG	NEG	POS	POS	POS	POS	POS	NEG	POS
Iasi-43-402	POS	POS	POS	AMB	AMB	POS	POS	NEG	NEG	POS	POS	POS	POS	POS	POS	NEG	POS
Iasi-44-404	POS	POS	POS	AMB	AMB	POS	POS	NEG	NEG	NEG	POS	POS	POS	POS	POS	NEG	POS
Iasi-54-430	POS	POS	POS	AMB	AMB	POS	POS	NEG	NEG	POS	POS	POS	POS	POS	POS	NEG	POS
Iasi-24-216	POS	POS	POS	AMB	AMB	POS	POS	NEG	POS	AMB	NEG	POS	POS	NEG	POS	POS	NEG
Iasi-05-S13	POS	POS	POS	AMB	AMB	POS	POS	NEG	POS	AMB	NEG	POS	POS	NEG	POS	POS	NEG
Iasi-45-362	POS	POS	NEG	NEG	POS	POS	POS	NEG	POS	AMB	NEG	POS	POS	POS	POS	NEG	POS
Iasi-22-199	POS	POS	NEG	NEG	POS	POS	POS	NEG	POS	AMB	NEG	POS	POS	POS	POS	NEG	POS
Iasi-12-S26	POS	POS	NEG	NEG	POS	POS	POS	NEG	AMB	POS	NEG	POS	POS	POS	POS	NEG	POS
Iasi-21-P2	POS	POS	NEG	NEG	POS	POS	POS	NEG	AMB	POS	NEG	POS	POS	POS	POS	NEG	POS
Iasi-06-60	POS	POS	NEG	NEG	POS	POS	POS	AMB	NEG	POS	POS	NEG	POS	POS	POS	NEG	POS
Iasi-12-79	POS	POS	NEG	NEG	POS	POS	POS	NEG	NEG	POS	POS	NEG	POS	POS	POS	NEG	POS
Iasi-17-90	POS	POS	NEG	NEG	POS	POS	POS	NEG	NEG	POS	POS	NEG	POS	POS	POS	NEG	POS
Iasi-21-105	POS	POS	NEG	NEG	POS	POS	POS	NEG	NEG	POS	POS	NEG	POS	POS	POS	NEG	POS
Iasi-22-109	POS	POS	NEG	NEG	POS	POS	POS	NEG	NEG	POS	POS	NEG	POS	POS	POS	NEG	POS
Iasi-23-110	POS	POS	NEG	NEG	POS	POS	POS	AMB	NEG	POS	POS	NEG	POS	POS	POS	NEG	POS
Iasi-24-136	POS	POS	NEG	NEG	POS	POS	POS	NEG	NEG	POS	POS	NEG	POS	POS	POS	NEG	POS
Iasi-34-224	POS	POS	NEG	NEG	POS	POS	POS	NEG	NEG	POS	POS	NEG	POS	POS	POS	NEG	POS
Iasi-39-297	POS	POS	NEG	NEG	POS	POS	POS	AMB	NEG	POS	POS	NEG	POS	POS	POS	NEG	POS
Iasi-50-431	POS	POS	NEG	NEG	POS	POS	POS	NEG	NEG	POS	POS	NEG	POS	POS	POS	NEG	POS
Iasi-51-432	POS	POS	NEG	NEG	POS	POS	POS	AMB	NEG	POS	POS	NEG	POS	POS	POS	NEG	POS
Iasi-08-76	POS	POS	NEG	NEG	POS	POS	POS	NEG	NEG	NEG	POS	NEG	POS	POS	POS	NEG	POS
Iasi-30-350	POS	POS	NEG	NEG	POS	POS	POS	NEG	NEG	POS	POS	NEG	POS	POS	POS	NEG	POS
Iasi-33-319	POS	POS	NEG	NEG	POS	POS	POS	NEG	NEG	NEG	POS	NEG	POS	AMB	POS	NEG	POS
Iasi-40-383	POS	POS	NEG	NEG	POS	POS	POS	NEG	NEG	POS	POS	NEG	POS	POS	POS	NEG	POS
Iasi-49-423	POS	POS	NEG	NEG	POS	POS	POS	NEG	NEG	POS	POS	NEG	POS	POS	POS	NEG	POS
Iasi-16-S31	POS	POS	NEG	NEG	POS	POS	POS	NEG	NEG	POS	POS	NEG	POS	POS	POS	NEG	POS
Iasi-24-P9	POS	POS	NEG	NEG	POS	POS	POS	NEG	NEG	POS	POS	NEG	POS	POS	POS	NEG	POS
Iasi-27-P12	POS	POS	NEG	NEG	POS	POS	POS	NEG	NEG	POS	POS	NEG	POS	POS	POS	NEG	POS
Iasi-38-P27	POS	POS	NEG	NEG	POS	POS	POS	NEG	NEG	POS	POS	NEG	POS	POS	POS	NEG	POS
Iasi-42-G511	POS	POS	NEG	NEG	POS	POS	POS	NEG	NEG	POS	POS	NEG	POS	POS	POS	NEG	POS
Iasi-46-3N	POS	POS	NEG	NEG	POS	POS	POS	NEG	NEG	POS	POS	NEG	POS	POS	POS	NEG	POS
Iasi-47-42O	POS	POS	NEG	NEG	POS	POS	POS	NEG	NEG	POS	POS	NEG	POS	POS	POS	NEG	POS
Iasi-48-36N	POS	POS	NEG	NEG	POS	POS	POS	NEG	NEG	POS	POS	NEG	POS	POS	POS	NEG	POS
Iasi-49-T3	POS	POS	NEG	NEG	POS	POS	POS	NEG	NEG	POS	POS	NEG	POS	POS	POS	NEG	POS
Iasi-50-T2	POS	POS	NEG	NEG	POS	POS	POS	AMB	NEG	POS	POS	NEG	POS	POS	POS	NEG	POS
Iasi-22-251	POS	POS	NEG	NEG	POS	POS	POS	NEG	NEG	POS	POS	NEG	POS	POS	POS	NEG	POS
Iasi-27-158	POS	POS	NEG	NEG	POS	POS	POS	NEG	NEG	POS	NEG	POS	POS	POS	POS	NEG	POS
Iasi-08-70	POS	POS	POS	AMB	NEG	POS	POS	NEG	NEG	POS	POS	POS	POS	POS	POS	NEG	NEG
Iasi-18-93	POS	POS	POS	AMB	NEG	POS	POS	NEG	NEG	POS	POS	POS	POS	POS	POS	NEG	NEG
Iasi-28-P14	POS	POS	POS	AMB	NEG	POS	POS	NEG	NEG	NEG	POS	POS	POS	POS	POS	NEG	NEG
Iasi-29-P15	POS	POS	POS	AMB	AMB	POS	POS	AMB	NEG	POS	POS	POS	POS	POS	POS	NEG	NEG
Iasi-03-19	POS	POS	AMB	NEG	POS	POS	POS	NEG	POS	AMB	POS	POS	POS	NEG	POS	POS	NEG
Iasi-11-77	POS	POS	AMB	NEG	POS	POS	POS	NEG	POS	AMB	POS	POS	POS	NEG	POS	POS	NEG
Iasi-13-82	POS	POS	AMB	NEG	POS	POS	POS	NEG	POS	AMB	POS	POS	POS	NEG	POS	POS	NEG
Iasi-15-86	POS	POS	POS	NEG	AMB	POS	POS	NEG	AMB	POS	POS	POS	POS	NEG	POS	POS	NEG
Iasi-20-103	POS	POS	AMB	NEG	POS	POS	POS	NEG	POS	AMB	POS	POS	POS	NEG	POS	POS	NEG
Iasi-33-222	POS	POS	AMB	NEG	POS	POS	POS	NEG	POS	AMB	POS	POS	POS	NEG	POS	POS	NEG
Iasi-44-360	POS	POS	AMB	NEG	POS	POS	POS	NEG	POS	AMB	POS	POS	POS	NEG	POS	POS	NEG
Iasi-47-399	POS	POS	AMB	NEG	POS	POS	POS	AMB	POS	AMB	POS	POS	POS	NEG	POS	POS	NEG
Iasi-34-368	POS	POS	AMB	NEG	POS	POS	POS	NEG	POS	AMB	POS	POS	POS	NEG	POS	POS	NEG
Iasi-36-371	POS	POS	AMB	NEG	POS	POS	POS	NEG	POS	AMB	POS	POS	POS	NEG	POS	POS	NEG
Iasi-04-S12	POS	POS	AMB	NEG	POS	POS	POS	NEG	AMB	POS	POS	POS	POS	NEG	POS	POS	NEG
Iasi-06-S15	POS	POS	AMB	NEG	POS	POS	POS	NEG	AMB	POS	POS	POS	POS	NEG	POS	POS	NEG
Iasi-07-S16	POS	POS	AMB	NEG	POS	POS	POS	NEG	AMB	POS	POS	POS	POS	NEG	POS	POS	NEG
Iasi-11-S24	POS	POS	AMB	NEG	POS	POS	POS	AMB	AMB	POS	POS	POS	POS	NEG	POS	POS	NEG
Iasi-22-P4	POS	POS	AMB	NEG	POS	POS	POS	NEG	AMB	POS	POS	POS	POS	NEG	POS	POS	NEG
Iasi-34-P23	POS	POS	AMB	NEG	POS	POS	POS	NEG	AMB	POS	POS	POS	POS	NEG	POS	POS	NEG
Iasi-37-P26	POS	POS	AMB	NEG	POS	POS	POS	AMB	POS	AMB	POS	POS	POS	NEG	POS	POS	NEG
Iasi-30-196	POS	POS	POS	NEG	AMB	POS	POS	AMB	POS	AMB	POS	POS	POS	NEG	POS	POS	POS
Iasi-27-298	POS	POS	NEG	NEG	POS	POS	POS	POS	NEG	NEG	NEG	POS	POS	POS	POS	NEG	NEG
Iasi-37-363	POS	POS	NEG	NEG	POS	POS	POS	POS	NEG	NEG	NEG	POS	POS	POS	POS	NEG	NEG
Iasi-38-372	POS	POS	NEG	NEG	POS	POS	POS	POS	NEG	NEG	NEG	POS	POS	POS	POS	NEG	NEG
Iasi-39-374	POS	POS	NEG	NEG	POS	POS	POS	POS	NEG	NEG	NEG	POS	POS	POS	POS	NEG	NEG
Iasi-28-299	POS	POS	NEG	NEG	POS	POS	POS	POS	NEG	NEG	NEG	POS	POS	POS	POS	NEG	NEG
Iasi-07-65	POS	POS	NEG	NEG	POS	POS	POS	NEG	NEG	POS	NEG	POS	POS	POS	POS	NEG	POS
Iasi-19-99	POS	POS	AMB	AMB	POS	POS	POS	NEG	NEG	POS	NEG	POS	POS	POS	POS	NEG	NEG
Iasi-25-217	POS	POS	POS	AMB	AMB	POS	POS	NEG	NEG	POS	NEG	POS	POS	POS	POS	NEG	NEG
Iasi-23-P5	POS	POS	AMB	AMB	POS	POS	POS	NEG	NEG	POS	NEG	POS	POS	POS	POS	NEG	NEG
Iasi-43-G513	POS	POS	AMB	AMB	POS	POS	POS	NEG	NEG	POS	NEG	POS	POS	POS	POS	NEG	NEG
Iasi-44-G514	POS	POS	POS	AMB	AMB	POS	POS	NEG	NEG	POS	NEG	POS	POS	POS	POS	NEG	NEG
Iasi-28-172	POS	POS	NEG	NEG	POS	POS	POS	AMB	NEG	POS	POS	POS	POS	NEG	POS	POS	POS
Iasi-42-358	POS	POS	NEG	NEG	POS	POS	POS	AMB	NEG	POS	POS	POS	POS	NEG	POS	POS	POS
Iasi-48-403	POS	POS	NEG	NEG	POS	POS	POS	AMB	NEG	POS	POS	POS	POS	NEG	POS	POS	AMB
Iasi-26-153	POS	POS	NEG	NEG	POS	POS	POS	NEG	NEG	POS	POS	POS	POS	NEG	POS	POS	NEG
Iasi-29-190	POS	POS	NEG	NEG	POS	POS	POS	AMB	NEG	POS	POS	POS	POS	NEG	POS	POS	NEG
Iasi-31-201	POS	POS	NEG	NEG	POS	POS	POS	NEG	NEG	POS	POS	POS	POS	NEG	POS	POS	NEG
Iasi-37-289	POS	POS	NEG	NEG	POS	POS	POS	AMB	NEG	POS	POS	POS	POS	NEG	POS	POS	AMB
Iasi-41-334	POS	POS	AMB	NEG	POS	POS	POS	NEG	NEG	POS	POS	POS	POS	NEG	POS	POS	POS
Iasi-52-433	POS	POS	NEG	NEG	POS	POS	POS	NEG	NEG	NEG	POS	POS	POS	NEG	POS	POS	NEG
Iasi-07-66	POS	POS	NEG	NEG	POS	POS	POS	NEG	NEG	NEG	POS	POS	POS	NEG	POS	POS	NEG
Iasi-23-211	POS	POS	NEG	NEG	POS	POS	POS	NEG	NEG	POS	POS	POS	POS	NEG	POS	POS	NEG
Iasi-43-401	POS	POS	NEG	NEG	POS	POS	POS	NEG	NEG	POS	POS	POS	POS	NEG	POS	POS	NEG
Iasi-47-418	POS	POS	NEG	NEG	POS	POS	POS	NEG	NEG	POS	POS	POS	POS	NEG	POS	POS	NEG
Iasi-18-169	POS	POS	NEG	NEG	POS	POS	POS	NEG	NEG	POS	NEG	POS	POS	NEG	POS	POS	NEG
Iasi-18-S34	POS	POS	NEG	NEG	POS	POS	POS	NEG	NEG	POS	POS	POS	POS	POS	POS	NEG	POS
Iasi-36-276	POS	POS	AMB	AMB	AMB	POS	POS	NEG	POS	AMB	NEG	POS	POS	NEG	POS	POS	NEG
Iasi-151	POS	POS	POS	AMB	AMB	POS	POS	AMB	AMB	POS	POS	POS	POS	NEG	POS	POS	POS
Iasi-02-11	POS	POS	NEG	NEG	POS	POS	POS	NEG	NEG	POS	NEG	POS	POS	NEG	POS	POS	NEG

Isolate	Group	ADHAESION FACTORS / GENES ENCODING MICROBIAL SURFACE COMPONENTS RECOGNIZING ADHESIVE MATRIX MOLECULES (MSCRAMM GENES)																																									
		enolase	fibrinogen binding protein (19 kDa)		fibronectin-binding protein A						fibronectin-binding protein B							Major histocompatibility complex class II analog protein (=Extracellular adherence protein, eap)				Staphylococcus aureus surface protein G					Ser-Asp rich fibrinogen-/bone sialoprotein-binding protein C							Ser-Asp rich fibrinogen-/bone sialoprotein-binding protein D					van Willebrand factor binding protein					
		eno	fib	fib (MRSA252)	fnbA (total)	fnbA (consensus)	fnbA (COL)	fnbA (MRSA252)	fnbA (Mu50+MW2)	fnbA (RF122)	fnbB (total)	fnbB (COL)	fnbB (COL+Mu50+MW2)	fnbB (Mu50)	fnbB (MW2)	fnbB (ST15)	fnbB (ST45-2)	map (total)	map (COL)	map (MRSA252)	map (Mu50+MW2)	sasG (total)	sasG (COL+Mu50)	sasG (MW2)	sasG (other than MRSA252+RF122)	sasX	sdrC (total)	sdrC (consensus)	sdrC (B1)	sdrC (COL)	sdrC (Mu50)	sdrC (MW2+MRSA252+RF122)	sdrC (other than MRSA252+RF122)	sdrD (total)	sdrD (consensus)	sdrD (COL+MW2)	sdrD (Mu50)	sdrD (other)	vwb (total)	vwb (consensus)	vwb (COL+MW2)	vwb (MRSA252)	vwb (Mu50)	vwb (RF122)
Iasi-01-4	BSI	POS	POS	NEG	POS	POS	NEG	NEG	POS	NEG	POS	NEG	AMB	NEG	POS	NEG	NEG	NEG	NEG	NEG	NEG	POS	NEG	POS	POS		POS	POS	NEG	NEG	NEG	POS	POS	POS	POS	POS	NEG	NEG	POS	POS	POS	NEG	NEG	NEG
Iasi-05-32	BSI	POS	POS	NEG	POS	POS	NEG	NEG	POS	NEG	POS	NEG	AMB	NEG	POS	NEG	NEG	NEG	NEG	NEG	AMB	POS	NEG	POS	POS		POS	POS	NEG	NEG	NEG	POS	POS	POS	POS	POS	NEG	NEG	POS	POS	POS	NEG	NEG	NEG
Iasi-16-88	BSI	POS	POS	NEG	POS	POS	NEG	NEG	POS	NEG	POS	NEG	AMB	NEG	POS	NEG	NEG	NEG	NEG	NEG	AMB	POS	NEG	POS	POS		POS	POS	NEG	NEG	NEG	POS	POS	POS	POS	POS	NEG	NEG	POS	POS	POS	NEG	NEG	NEG
Iasi-25-141	BSI	POS	POS	AMB	POS	POS	NEG	NEG	POS	NEG	POS	NEG	AMB	AMB	POS	NEG	NEG	NEG	AMB	NEG	AMB	POS	NEG	POS	POS		POS	POS	NEG	NEG	NEG	POS	POS	POS	POS	POS	NEG	NEG	POS	POS	POS	NEG	NEG	NEG
Iasi-32-215	BSI	POS	POS	AMB	POS	POS	NEG	NEG	POS	NEG	POS	AMB	AMB	AMB	POS	NEG	NEG	POS	AMB	NEG	POS	POS	NEG	POS	POS		POS	POS	AMB	NEG	NEG	POS	POS	POS	POS	POS	NEG	AMB	POS	POS	POS	NEG	NEG	NEG
Iasi-43-359	BSI	POS	POS	NEG	POS	POS	NEG	NEG	POS	NEG	POS	NEG	AMB	NEG	POS	NEG	NEG	NEG	NEG	NEG	AMB	POS	NEG	POS	POS		POS	POS	NEG	NEG	NEG	POS	POS	POS	POS	POS	NEG	NEG	POS	POS	POS	NEG	NEG	NEG
Iasi-03-27	SSTI	POS	POS	NEG	POS	POS	NEG	NEG	POS	NEG	POS	NEG	POS	NEG	AMB	NEG	NEG	NEG	NEG	NEG	AMB	POS	NEG	POS	POS		POS	POS	NEG	NEG	NEG	POS	POS	POS	POS	POS	NEG	NEG	POS	POS	POS	NEG	NEG	NEG
Iasi-13-149	SSTI	POS	POS	NEG	POS	POS	NEG	NEG	POS	NEG	POS	NEG	POS	NEG	AMB	NEG	NEG	NEG	NEG	NEG	AMB	POS	NEG	POS	POS		POS	POS	NEG	NEG	NEG	POS	POS	POS	POS	POS	NEG	NEG	POS	POS	POS	NEG	NEG	NEG
Iasi-29-337	SSTI	POS	POS	NEG	POS	POS	NEG	NEG	POS	NEG	POS	NEG	AMB	NEG	POS	NEG	NEG	NEG	NEG	NEG	NEG	POS	NEG	POS	POS		POS	POS	NEG	NEG	NEG	POS	POS	POS	POS	POS	NEG	NEG	POS	POS	POS	NEG	NEG	NEG
Iasi-31-355	SSTI	POS	POS	NEG	POS	POS	NEG	NEG	POS	NEG	POS	NEG	AMB	NEG	POS	NEG	NEG	POS	AMB	NEG	POS	POS	NEG	POS	POS		POS	POS	NEG	NEG	NEG	POS	POS	POS	POS	POS	NEG	NEG	POS	POS	POS	NEG	NEG	NEG
Iasi-38-377	SSTI	POS	POS	NEG	POS	POS	NEG	NEG	POS	NEG	POS	NEG	POS	NEG	AMB	NEG	NEG	POS	AMB	NEG	POS	POS	NEG	POS	POS		POS	POS	NEG	NEG	NEG	POS	POS	POS	POS	POS	NEG	NEG	POS	POS	POS	NEG	NEG	NEG
Iasi-41-388	SSTI	POS	POS	NEG	POS	POS	NEG	NEG	POS	NEG	POS	NEG	AMB	NEG	POS	NEG	NEG	NEG	NEG	NEG	AMB	POS	NEG	POS	POS		POS	POS	NEG	NEG	NEG	POS	POS	POS	POS	POS	NEG	NEG	POS	POS	POS	NEG	NEG	NEG
Iasi-46-417	SSTI	POS	POS	NEG	POS	POS	NEG	NEG	POS	NEG	POS	NEG	POS	NEG	AMB	NEG	NEG	NEG	NEG	NEG	AMB	POS	NEG	POS	POS		POS	POS	NEG	NEG	NEG	POS	POS	POS	POS	POS	NEG	NEG	POS	POS	POS	NEG	NEG	NEG
Iasi-02-S9	Carrier	POS	POS	AMB	POS	POS	NEG	NEG	POS	NEG	POS	NEG	AMB	NEG	POS	NEG	NEG	POS	POS	NEG	POS	POS	NEG	POS	POS		POS	POS	NEG	NEG	NEG	POS	POS	POS	POS	POS	NEG	NEG	POS	POS	POS	NEG	NEG	NEG
Iasi-17-S33	Carrier	POS	POS	NEG	POS	POS	NEG	NEG	POS	NEG	POS	NEG	AMB	NEG	POS	NEG	NEG	NEG	NEG	NEG	AMB	POS	NEG	POS	POS		POS	POS	NEG	NEG	NEG	POS	POS	POS	POS	POS	NEG	NEG	POS	POS	POS	NEG	NEG	NEG
Iasi-30-P17	Carrier	POS	POS	AMB	POS	POS	NEG	NEG	POS	NEG	POS	NEG	AMB	NEG	POS	NEG	NEG	POS	AMB	NEG	POS	POS	NEG	POS	POS		POS	POS	NEG	NEG	NEG	POS	POS	POS	POS	POS	NEG	NEG	POS	POS	POS	NEG	NEG	NEG
Iasi-39-P28	Carrier	POS	POS	NEG	POS	POS	NEG	NEG	POS	NEG	POS	NEG	AMB	NEG	POS	NEG	NEG	POS	AMB	NEG	POS	POS	NEG	POS	POS		POS	POS	NEG	NEG	NEG	POS	POS	POS	POS	POS	NEG	NEG	POS	POS	POS	NEG	NEG	NEG
Iasi-04-26	BSI	POS	POS	NEG	POS	POS	NEG	NEG	POS	NEG	POS	NEG	AMB	NEG	POS	NEG	NEG	NEG	NEG	NEG	AMB	POS	NEG	POS	POS		POS	POS	NEG	NEG	NEG	POS	POS	POS	POS	POS	NEG	NEG	POS	POS	POS	NEG	NEG	NEG
Iasi-09-72	BSI	POS	POS	NEG	POS	POS	NEG	NEG	POS	NEG	POS	NEG	AMB	NEG	POS	NEG	NEG	NEG	NEG	NEG	NEG	POS	NEG	POS	POS		POS	POS	NEG	NEG	NEG	POS	POS	POS	POS	POS	NEG	NEG	POS	POS	POS	NEG	NEG	NEG
Iasi-11-131	BSI	POS	POS	NEG	POS	POS	NEG	NEG	POS	NEG	VAR	NEG	NEG	VAR	NEG	NEG	NEG	VAR	NEG	NEG	VAR	POS	NEG	POS	POS		POS	POS	NEG	NEG	NEG	POS	POS	POS	POS	POS	NEG	NEG	POS	POS	POS	NEG	NEG	NEG
Iasi-14-143	BSI	POS	POS	NEG	POS	POS	NEG	NEG	POS	NEG	POS	NEG	AMB	NEG	POS	NEG	NEG	NEG	NEG	NEG	NEG	POS	NEG	POS	POS		POS	POS	NEG	NEG	NEG	POS	POS	POS	POS	POS	NEG	NEG	POS	POS	POS	NEG	NEG	NEG
Iasi-18-192	BSI	POS	POS	NEG	POS	POS	NEG	NEG	POS	NEG	POS	NEG	AMB	NEG	POS	NEG	NEG	NEG	NEG	NEG	AMB	POS	NEG	POS	POS		POS	POS	NEG	NEG	NEG	POS	POS	POS	POS	POS	NEG	NEG	POS	POS	POS	NEG	NEG	NEG
Iasi-23-253	BSI	POS	POS	AMB	POS	POS	NEG	NEG	POS	NEG	POS	NEG	AMB	AMB	POS	NEG	NEG	POS	POS	NEG	POS	POS	NEG	POS	POS		POS	POS	NEG	NEG	NEG	POS	POS	POS	POS	POS	NEG	NEG	POS	POS	POS	NEG	NEG	NEG
Iasi-35-322	BSI	POS	POS	NEG	POS	POS	NEG	NEG	POS	NEG	POS	NEG	AMB	NEG	POS	NEG	NEG	NEG	NEG	NEG	AMB	POS	NEG	POS	POS		POS	POS	NEG	NEG	NEG	POS	POS	POS	POS	POS	NEG	NEG	POS	POS	POS	NEG	NEG	NEG
Iasi-40-380	BSI	POS	POS	NEG	POS	POS	NEG	NEG	POS	NEG	POS	NEG	AMB	NEG	POS	NEG	NEG	NEG	NEG	NEG	AMB	POS	NEG	POS	POS		POS	POS	NEG	NEG	NEG	POS	POS	POS	POS	POS	NEG	NEG	POS	POS	POS	NEG	NEG	NEG
Iasi-46-406	BSI	POS	POS	NEG	POS	POS	NEG	NEG	POS	NEG	POS	NEG	AMB	NEG	POS	NEG	NEG	NEG	NEG	NEG	AMB	POS	NEG	POS	POS		POS	POS	NEG	NEG	NEG	POS	POS	POS	POS	POS	NEG	NEG	POS	POS	POS	NEG	NEG	NEG
Iasi-02-6	SSTI	POS	POS	NEG	POS	POS	NEG	NEG	POS	NEG	POS	NEG	AMB	NEG	POS	NEG	NEG	NEG	NEG	NEG	NEG	POS	NEG	POS	POS		POS	POS	NEG	NEG	NEG	POS	POS	POS	POS	POS	NEG	NEG	POS	POS	POS	NEG	NEG	NEG
Iasi-09-97	SSTI	POS	POS	NEG	POS	POS	NEG	NEG	POS	NEG	POS	NEG	AMB	NEG	POS	NEG	NEG	NEG	NEG	NEG	NEG	POS	NEG	POS	POS		POS	POS	NEG	NEG	NEG	POS	POS	POS	POS	POS	NEG	NEG	POS	POS	POS	NEG	NEG	NEG
Iasi-19-182	SSTI	POS	POS	NEG	POS	POS	NEG	NEG	POS	NEG	POS	NEG	AMB	NEG	POS	NEG	NEG	NEG	NEG	NEG	AMB	POS	NEG	POS	POS		POS	POS	NEG	NEG	NEG	POS	POS	POS	POS	POS	NEG	NEG	POS	POS	POS	NEG	NEG	NEG
Iasi-31-314	SSTI	POS	POS	NEG	POS	POS	NEG	NEG	POS	NEG	POS	NEG	AMB	NEG	POS	NEG	NEG	NEG	NEG	NEG	NEG	POS	NEG	POS	POS		POS	POS	NEG	NEG	NEG	POS	POS	POS	POS	POS	NEG	NEG	POS	POS	POS	NEG	NEG	NEG
Iasi-33-367	SSTI	POS	POS	NEG	POS	POS	NEG	NEG	POS	NEG	POS	NEG	AMB	NEG	POS	NEG	NEG	POS	AMB	NEG	POS	POS	NEG	POS	POS		POS	POS	NEG	NEG	NEG	POS	POS	POS	POS	POS	NEG	NEG	POS	POS	POS	NEG	NEG	NEG
Iasi-39-378	SSTI	POS	POS	NEG	POS	POS	NEG	NEG	POS	NEG	POS	NEG	AMB	NEG	POS	NEG	NEG	NEG	NEG	NEG	AMB	POS	NEG	POS	POS		POS	POS	NEG	NEG	NEG	POS	POS	POS	POS	POS	NEG	NEG	POS	POS	POS	NEG	NEG	NEG
Iasi-42-394	SSTI	POS	POS	NEG	POS	POS	NEG	NEG	POS	NEG	POS	NEG	POS	NEG	AMB	NEG	NEG	NEG	NEG	NEG	AMB	POS	NEG	POS	POS		POS	POS	NEG	NEG	NEG	POS	POS	POS	POS	POS	NEG	NEG	POS	POS	POS	NEG	NEG	NEG
Iasi-44-409	SSTI	POS	POS	NEG	POS	POS	NEG	NEG	POS	NEG	POS	NEG	POS	NEG	AMB	NEG	NEG	NEG	NEG	NEG	AMB	POS	NEG	POS	POS		POS	POS	NEG	NEG	NEG	POS	POS	NEG	NEG	NEG	NEG	NEG	POS	POS	POS	NEG	NEG	NEG
Iasi-45-405	SSTI	POS	POS	NEG	POS	POS	NEG	NEG	POS	NEG	POS	NEG	AMB	NEG	POS	NEG	NEG	NEG	NEG	NEG	AMB	POS	NEG	POS	POS		POS	POS	NEG	NEG	NEG	POS	POS	POS	POS	POS	NEG	NEG	POS	POS	POS	NEG	NEG	NEG
Iasi-50-425	SSTI	POS	POS	NEG	POS	POS	NEG	NEG	POS	NEG	NEG	NEG	NEG	NEG	NEG	NEG	NEG	NEG	NEG	NEG	NEG	POS	NEG	POS	POS		POS	POS	NEG	NEG	NEG	POS	POS	POS	POS	POS	NEG	NEG	POS	POS	POS	NEG	NEG	NEG
Iasi-50-428	SSTI	POS	POS	NEG	POS	POS	NEG	NEG	POS	NEG	POS	NEG	AMB	NEG	POS	NEG	NEG	POS	NEG	NEG	POS	POS	NEG	POS	POS		POS	POS	NEG	NEG	NEG	POS	POS	POS	POS	POS	NEG	NEG	POS	POS	POS	NEG	NEG	NEG
Iasi-51-37	SSTI	POS	POS	NEG	POS	POS	NEG	NEG	POS	NEG	POS	NEG	AMB	NEG	POS	NEG	NEG	NEG	NEG	NEG	AMB	POS	NEG	POS	POS		POS	POS	NEG	NEG	NEG	POS	POS	POS	POS	POS	NEG	NEG	POS	POS	POS	NEG	NEG	NEG
Iasi-01-S8	Carrier	POS	POS	NEG	POS	POS	NEG	NEG	POS	NEG	POS	NEG	AMB	NEG	POS	NEG	NEG	POS	AMB	NEG	POS	POS	NEG	POS	POS		POS	POS	NEG	NEG	NEG	POS	POS	POS	POS	POS	NEG	NEG	POS	POS	POS	NEG	NEG	NEG
Iasi-08-S17	Carrier	POS	POS	NEG	POS	POS	NEG	NEG	POS	NEG	POS	NEG	AMB	NEG	POS	NEG	NEG	NEG	NEG	NEG	AMB	POS	NEG	POS	POS		POS	POS	NEG	NEG	NEG	POS	POS	POS	POS	POS	NEG	NEG	POS	POS	POS	NEG	NEG	NEG
Iasi-10-S23	Carrier	POS	POS	NEG	POS	POS	NEG	NEG	POS	NEG	POS	NEG	AMB	NEG	POS	NEG	NEG	POS	AMB	NEG	POS	POS	NEG	POS	POS		POS	POS	NEG	NEG	NEG	POS	POS	POS	POS	POS	NEG	NEG	POS	POS	POS	NEG	NEG	NEG
Iasi-13-S27	Carrier	POS	POS	AMB	POS	POS	NEG	NEG	POS	NEG	POS	NEG	AMB	NEG	POS	NEG	NEG	POS	AMB	NEG	POS	POS	NEG	POS	POS		POS	POS	NEG	NEG	NEG	POS	POS	POS	POS	POS	NEG	NEG	POS	POS	POS	NEG	NEG	NEG
Iasi-14-S28	Carrier	POS	POS	NEG	POS	POS	NEG	NEG	POS	NEG	POS	NEG	AMB	NEG	POS	NEG	NEG	POS	AMB	NEG	POS	POS	NEG	POS	POS		POS	POS	NEG	NEG	NEG	POS	POS	POS	POS	POS	NEG	NEG	POS	POS	POS	NEG	NEG	NEG
Iasi-15-S29	Carrier	POS	POS	AMB	POS	POS	NEG	NEG	POS	NEG	POS	NEG	AMB	NEG	POS	NEG	NEG	POS	AMB	NEG	POS	POS	NEG	POS	POS		POS	POS	NEG	NEG	NEG	POS	POS	POS	POS	POS	NEG	NEG	POS	POS	POS	NEG	NEG	NEG
Iasi-25-P10	Carrier	POS	POS	AMB	POS	POS	NEG	NEG	POS	NEG	POS	NEG	AMB	NEG	POS	NEG	NEG	POS	AMB	NEG	POS	POS	NEG	POS	POS		POS	POS	NEG	NEG	NEG	POS	POS	NEG	NEG	NEG	NEG	NEG	POS	POS	POS	NEG	NEG	NEG
Iasi-26-P11	Carrier	POS	POS	NEG	POS	POS	NEG	NEG	POS	NEG	POS	NEG	AMB	NEG	POS	NEG	NEG	NEG	NEG	NEG	AMB	POS	NEG	POS	POS		POS	POS	NEG	NEG	NEG	POS	POS	POS	POS	POS	NEG	NEG	POS	POS	POS	NEG	NEG	NEG
Iasi-32-P19	Carrier	POS	POS	NEG	POS	POS	NEG	NEG	POS	NEG	POS	NEG	AMB	NEG	POS	NEG	NEG	POS	AMB	NEG	POS	POS	NEG	POS	POS		POS	POS	NEG	NEG	NEG	POS	POS	POS	POS	POS	NEG	NEG	POS	POS	POS	NEG	NEG	NEG
Iasi-33-P22	Carrier	POS	POS	NEG	POS	POS	NEG	NEG	POS	NEG	POS	NEG	AMB	NEG	POS	NEG	NEG	POS	AMB	NEG	POS	POS	NEG	POS	POS		POS	POS	NEG	NEG	NEG	POS	POS	POS	POS	POS	NEG	NEG	POS	POS	POS	NEG	NEG	NEG
Iasi-41-P44	Carrier	POS	POS	NEG	POS	POS	NEG	NEG	POS	NEG	POS	NEG	AMB	NEG	POS	NEG	NEG	NEG	AMB	NEG	AMB	POS	NEG	POS	POS		POS	POS	NEG	NEG	NEG	POS	POS	POS	POS	POS	NEG	NEG	POS	POS	POS	NEG	NEG	NEG
Iasi-45-G515	Carrier	POS	POS	NEG	POS	POS	NEG	NEG	POS	NEG	POS	NEG	AMB	NEG	POS	NEG	NEG	NEG	NEG	NEG	AMB	POS	NEG	POS	POS		POS	POS	NEG	NEG	NEG	POS	POS	POS	POS	POS	NEG	NEG	POS	POS	POS	NEG	NEG	NEG
Iasi-02-39	misc. MRSA	POS	POS	NEG	POS	POS	NEG	NEG	POS	NEG	POS	NEG	AMB	NEG	POS	NEG	NEG	NEG	NEG	NEG	NEG	POS	NEG	POS	POS		POS	POS	NEG	NEG	NEG	POS	POS	POS	POS	POS	NEG	NEG	POS	POS	POS	NEG	NEG	NEG
Iasi-24-263	misc. MRSA	POS	POS	NEG	POS	POS	NEG	NEG	POS	NEG	POS	NEG	AMB	NEG	POS	NEG	NEG	POS	AMB	NEG	POS	POS	NEG	POS	POS		POS	POS	NEG	NEG	NEG	POS	POS	POS	POS	POS	NEG	NEG	POS	POS	POS	NEG	NEG	NEG
Iasi-26-285	misc. MRSA	POS	POS	NEG	POS	POS	NEG	NEG	POS	NEG	POS	NEG	AMB	NEG	POS	NEG	NEG	POS	NEG	NEG	AMB	POS	NEG	POS	POS		POS	POS	NEG	NEG	NEG	POS	POS	POS	POS	POS	NEG	NEG	POS	POS	POS	NEG	NEG	NEG
Iasi-30-306	misc. MRSA	POS	POS	AMB	POS	POS	NEG	NEG	POS	NEG	POS	NEG	AMB	AMB	POS	NEG	NEG	POS	POS	NEG	POS	POS	NEG	POS	POS		POS	POS	NEG	NEG	NEG	POS	POS	POS	POS	POS	NEG	NEG	POS	POS	POS	NEG	NEG	NEG
Iasi-41-390	misc. MRSA	POS	POS	NEG	POS	POS	NEG	NEG	POS	NEG	POS	NEG	AMB	NEG	POS	NEG	NEG	POS	AMB	NEG	POS	POS	NEG	POS	POS		POS	POS	NEG	NEG	NEG	POS	POS	POS	POS	POS	NEG	NEG	POS	POS	POS	NEG	NEG	NEG
Iasi-47-407	misc. MRSA	POS	POS	NEG	POS	POS	NEG	NEG	POS	NEG	POS	NEG	AMB	NEG	POS	NEG	NEG	NEG	AMB	NEG	AMB	POS	NEG	POS	POS		POS	POS	NEG	NEG	NEG	POS	POS	POS	POS	POS	NEG	NEG	POS	POS	POS	NEG	NEG	NEG
Iasi-55-408CP	misc. MRSA	POS	POS	NEG	POS	POS	NEG	NEG	POS	NEG	POS	NEG	AMB	NEG	POS	NEG	NEG	NEG	NEG	NEG	NEG	POS	NEG	POS	POS		POS	POS	NEG	NEG	NEG	POS	POS	POS	POS	POS	NEG	NEG	POS	POS	POS	NEG	NEG	NEG
Iasi-15-151	BSI	POS	POS	AMB	POS	POS	NEG	NEG	POS	NEG	POS	NEG	AMB	POS	NEG	AMB	NEG	POS	POS	NEG	POS	POS	POS	NEG	POS		POS	POS	AMB	NEG	POS	AMB	POS	POS	POS	NEG	POS	NEG	POS	POS	NEG	NEG	POS	NEG
Iasi-46-386	BSI	POS	POS	AMB	POS	POS	NEG	NEG	POS	NEG	POS	NEG	AMB	POS	NEG	AMB	NEG	POS	POS	NEG	POS	POS	POS	AMB	POS		POS	POS	POS	NEG	POS	AMB	POS	POS	POS	NEG	POS	NEG	POS	POS	NEG	NEG	POS	NEG
Iasi-21-195	SSTI	POS	POS	NEG	POS	POS	NEG	NEG	POS	NEG	POS	NEG	POS	AMB	NEG	NEG	NEG	POS	NEG	NEG	POS	POS	POS	NEG	POS		POS	POS	NEG	NEG	POS	NEG	POS	POS	POS	NEG	POS	NEG	POS	POS	NEG	NEG	POS	NEG
Iasi-37-375	SSTI	POS	POS	NEG	POS	POS	NEG	NEG	POS	NEG	POS	NEG	AMB	POS	NEG	AMB	NEG	POS	AMB	NEG	POS	POS	POS	NEG	POS		POS	POS	NEG	NEG	POS	NEG	POS	POS	POS	NEG	POS	NEG	POS	POS	NEG	NEG	POS	NEG
Iasi-45-415	SSTI	POS	POS	NEG	POS	POS	NEG	NEG	POS	NEG	POS	NEG	AMB	POS	NEG	AMB	NEG	POS	POS	NEG	POS	POS	POS	NEG	POS		POS	POS	NEG	NEG	POS	NEG	POS	POS	POS	NEG	POS	NEG	POS	POS	NEG	NEG	POS	NEG
Iasi-09-S19	Carrier	POS	POS	AMB	POS	POS	NEG	NEG	POS	NEG	POS	NEG	AMB	POS	NEG	AMB	NEG	POS	POS	AMB	POS	POS	POS	AMB	POS		POS	POS	AMB	NEG	POS	AMB	POS	POS	POS	NEG	POS	NEG	POS	POS	NEG	NEG	POS	NEG
Iasi-53-434	BSI	POS	POS	AMB	POS	POS	NEG	NEG	POS	NEG	POS	NEG	AMB	POS	NEG	AMB	NEG	POS	POS	NEG	POS	POS	POS	AMB	POS		POS	POS	POS	NEG	POS	AMB	POS	POS	POS	NEG	POS	NEG	POS	POS	NEG	NEG	POS	NEG
Iasi-03-40	misc. MRSA	POS	POS	AMB	POS	POS	NEG	NEG	POS	NEG	POS	NEG	AMB	POS	NEG	AMB	NEG	POS	POS	NEG	POS	POS	POS	NEG	POS		POS	POS	AMB	NEG	POS	NEG	POS	POS	POS	NEG	POS	NEG	POS	POS	NEG	NEG	POS	NEG
Iasi-04-43	misc. MRSA	POS	POS	NEG	POS	POS	NEG	NEG	POS	NEG	POS	NEG	AMB	POS	NEG	NEG	NEG	POS	AMB	NEG	POS	POS	POS	NEG	POS		POS	POS	NEG	NEG	POS	NEG	POS	POS	POS	NEG	POS	NEG	POS	POS	NEG	NEG	POS	NEG
Iasi-01-20	misc. MRSA	POS	POS	NEG	POS	POS	NEG	NEG	POS	NEG	POS	NEG	AMB	POS	NEG	AMB	NEG	POS	POS	NEG	POS	POS	POS	NEG	POS		POS	POS	AMB	NEG	POS	NEG	POS	POS	POS	NEG	POS	NEG	POS	POS	NEG	NEG	POS	NEG
Iasi-35-228	BSI	POS	POS	NEG	POS	POS	NEG	POS	NEG	NEG	POS	NEG	AMB	POS	NEG	NEG	NEG	POS	NEG	NEG	POS	POS	NEG	POS	POS		POS	POS	NEG	POS	NEG	NEG	POS	POS	POS	NEG	POS	NEG	POS	POS	NEG	POS	NEG	NEG
Iasi-38-293	BSI	POS	POS	POS	POS	POS	NEG	NEG	NEG	NEG	POS	NEG	AMB	AMB	NEG	NEG	POS	POS	AMB	NEG	POS	NEG	NEG	NEG	NEG		POS	POS	AMB	POS	NEG	AMB	POS	POS	POS	NEG	POS	NEG	POS	POS	NEG	NEG	POS	NEG
Iasi-31-P18	Carrier	POS	POS	AMB	POS	POS	NEG	NEG	NEG	NEG	POS	NEG	AMB	NEG	AMB	NEG	POS	POS	AMB	NEG	POS	NEG	NEG	NEG	NEG		POS	POS	NEG	POS	NEG	NEG	POS	POS	POS	NEG	POS	NEG	POS	POS	NEG	NEG	NEG	NEG
Iasi-40-P42	Carrier	POS	POS	AMB	POS	POS	NEG	NEG	NEG	NEG	POS	NEG	AMB	NEG	NEG	NEG	POS	POS	POS	AMB	POS	NEG	NEG	AMB	NEG		POS	POS	AMB	POS	NEG	AMB	POS	POS	POS	NEG	POS	NEG	POS	POS	NEG	NEG	POS	NEG
Iasi-40-303	BSI	POS	POS	NEG	POS	POS	POS	NEG	NEG	NEG	POS	POS	AMB	AMB	NEG	NEG	NEG	POS	POS	NEG	AMB	POS	POS	NEG	POS		POS	POS	NEG	POS	NEG	NEG	POS	POS	POS	POS	NEG	NEG	POS	POS	POS	NEG	NEG	NEG
Iasi-48-422	SSTI	POS	POS	NEG	POS	POS	POS	NEG	NEG	NEG	POS	POS	AMB	NEG	NEG	NEG	NEG	POS	POS	NEG	NEG	POS	POS	NEG	POS		POS	POS	NEG	POS	NEG	NEG	POS	POS	POS	POS	NEG	NEG	POS	POS	POS	NEG	NEG	NEG
Iasi-54-435	BSI	POS	POS	POS	POS	POS	POS	NEG	NEG	NEG	POS	AMB	AMB	POS	AMB	NEG	AMB	POS	POS	NEG	POS	POS	POS	AMB	POS		POS	POS	POS	POS	NEG	POS	POS	POS	POS	POS	NEG	NEG	POS	POS	POS	NEG	NEG	NEG
Iasi-16-161	SSTI	POS	POS	NEG	POS	POS	POS	NEG	NEG	NEG	POS	POS	AMB	AMB	NEG	NEG	NEG	POS	POS	NEG	NEG	POS	POS	NEG	POS		POS	POS	NEG	POS	NEG	NEG	POS	POS	POS	POS	NEG	NEG	POS	POS	POS	NEG	NEG	NEG
Iasi-27-221	SSTI	POS	POS	NEG	POS	POS	POS	NEG	NEG	NEG	POS	POS	AMB	AMB	NEG	NEG	NEG	POS	POS	NEG	NEG	POS	POS	NEG	POS		POS	POS	NEG	POS	NEG	NEG	POS	POS	POS	POS	NEG	NEG	POS	POS	POS	NEG	NEG	NEG
Iasi-09-85	BSI	POS	POS	NEG	POS	POS	POS	NEG	NEG	NEG	POS	POS	AMB	NEG	NEG	NEG	NEG	POS	POS	NEG	NEG	POS	POS	NEG	POS		POS	POS	NEG	POS	NEG	NEG	POS	POS	POS	POS	NEG	NEG	POS	POS	POS	NEG	NEG	NEG
Iasi-17-189	BSI	POS	POS	NEG	POS	POS	POS	NEG	NEG	NEG	POS	POS	AMB	AMB	NEG	NEG	NEG	POS	POS	NEG	NEG	POS	POS	NEG	POS		POS	POS	NEG	POS	NEG	NEG	POS	POS	POS	POS	NEG	NEG	POS	POS	POS	NEG	NEG	NEG
Iasi-20-200	BSI	POS	POS	NEG	POS	POS	POS	NEG	NEG	NEG	POS	POS	AMB	NEG	NEG	NEG	NEG	POS	POS	NEG	NEG	POS	POS	NEG	POS		POS	POS	NEG	POS	NEG	NEG	POS	POS	POS	POS	NEG	NEG	POS	POS	POS	NEG	NEG	NEG
Iasi-34-321	BSI	POS	POS	NEG	POS	POS	POS	NEG	NEG	NEG	POS	POS	AMB	AMB	NEG	NEG	NEG	POS	POS	NEG	NEG	POS	POS	NEG	POS		POS	POS	NEG	POS	NEG	NEG	POS	POS	POS	POS	NEG	NEG	POS	POS	POS	NEG	NEG	NEG
Iasi-04-57	SSTI	POS	POS	NEG	POS	POS	POS	NEG	NEG	NEG	POS	POS	AMB	NEG	NEG	NEG	NEG	POS	POS	NEG	NEG	POS	POS	NEG	POS		POS	POS	NEG	POS	NEG	NEG	POS	POS	POS	POS	NEG	NEG	POS	POS	POS	NEG	NEG	NEG
Iasi-05-59	SSTI	POS	POS	NEG	POS	POS	POS	NEG	NEG	NEG	POS	POS	AMB	AMB	NEG	NEG	NEG	POS	POS	NEG	NEG	POS	POS	NEG	POS		POS	POS	NEG	POS	NEG	NEG	POS	POS	POS	POS	NEG	NEG	POS	POS	POS	NEG	NEG	NEG
Iasi-06-64	SSTI	POS	POS	NEG	POS	POS	POS	NEG	NEG	NEG	POS	POS	AMB	NEG	NEG	NEG	NEG	POS	POS	NEG	NEG	POS	POS	NEG	POS		POS	POS	NEG	POS	NEG	NEG	POS	POS	POS	POS	NEG	NEG	POS	POS	POS	NEG	NEG	NEG
Iasi-10-108	SSTI	POS	POS	NEG	POS	POS	POS	NEG	NEG	NEG	POS	POS	AMB	NEG	NEG	NEG	NEG	POS	POS	NEG	NEG	POS	POS	NEG	POS		POS	POS	NEG	POS	NEG	NEG	POS	POS	POS	POS	NEG	NEG	POS	POS	POS	NEG	NEG	NEG
Iasi-12-117	SSTI	POS	POS	NEG	POS	POS	POS	NEG	NEG	NEG	POS	POS	AMB	AMB	NEG	NEG	NEG	POS	POS	NEG	AMB	POS	POS	NEG	POS		POS	POS	NEG	POS	NEG	NEG	POS	POS	POS	POS	NEG	NEG	POS	POS	POS	NEG	NEG	NEG
Iasi-12-138	SSTI	POS	POS	NEG	POS	POS	POS	NEG	NEG	NEG	POS	POS	AMB	AMB	NEG	NEG	NEG	POS	POS	NEG	AMB	POS	POS	NEG	POS		POS	POS	NEG	POS	NEG	NEG	POS	POS	POS	POS	NEG	NEG	POS	POS	POS	NEG	NEG	NEG
Iasi-13-140	SSTI	POS	POS	NEG	POS	POS	POS	NEG	NEG	NEG	POS	POS	AMB	NEG	NEG	NEG	NEG	POS	POS	NEG	NEG	POS	POS	NEG	POS		POS	POS	NEG	POS	NEG	NEG	POS	POS	POS	POS	NEG	NEG	POS	POS	POS	NEG	NEG	NEG
Iasi-15-152	SSTI	POS	POS	NEG	POS	POS	POS	NEG	NEG	NEG	POS	POS	AMB	AMB	NEG	NEG	NEG	POS	POS	NEG	NEG	POS	POS	NEG	POS		POS	POS	NEG	POS	NEG	NEG	POS	POS	POS	POS	NEG	NEG	POS	POS	POS	NEG	NEG	NEG
Iasi-17-168	SSTI	POS	POS	NEG	POS	POS	POS	NEG	NEG	NEG	POS	POS	AMB	AMB	NEG	NEG	NEG	POS	POS	NEG	NEG	POS	POS	NEG	POS		POS	POS	NEG	POS	NEG	NEG	POS	POS	POS	POS	NEG	NEG	POS	POS	POS	NEG	NEG	NEG
Iasi-20-193	SSTI	POS	POS	NEG	POS	POS	POS	NEG	NEG	NEG	POS	POS	AMB	AMB	NEG	NEG	NEG	POS	POS	NEG	NEG	POS	POS	NEG	POS		POS	POS	NEG	POS	NEG	NEG	POS	POS	POS	POS	NEG	NEG	POS	POS	POS	NEG	NEG	NEG
Iasi-26-218	SSTI	POS	POS	NEG	POS	POS	POS	NEG	NEG	NEG	POS	POS	AMB	AMB	NEG	NEG	NEG	POS	POS	NEG	NEG	POS	POS	NEG	POS		POS	POS	NEG	POS	NEG	NEG	POS	POS	POS	POS	NEG	NEG	POS	POS	POS	NEG	NEG	NEG
Iasi-28-335	SSTI	POS	POS	NEG	POS	POS	POS	NEG	NEG	NEG	POS	POS	AMB	NEG	NEG	NEG	NEG	POS	POS	NEG	NEG	POS	POS	NEG	POS		POS	POS	NEG	POS	NEG	NEG	POS	POS	POS	POS	NEG	NEG	POS	POS	POS	NEG	NEG	NEG
Iasi-29-299b	SSTI	POS	POS	AMB	POS	POS	POS	NEG	NEG	NEG	POS	POS	AMB	AMB	NEG	NEG	NEG	POS	POS	NEG	AMB	POS	POS	NEG	POS		POS	POS	AMB	POS	NEG	NEG	POS	POS	POS	POS	NEG	NEG	POS	POS	POS	NEG	NEG	NEG
Iasi-32-364	SSTI	POS	POS	NEG	POS	POS	POS	NEG	NEG	NEG	POS	POS	AMB	NEG	NEG	NEG	NEG	POS	POS	NEG	NEG	POS	POS	NEG	POS		POS	POS	NEG	POS	NEG	NEG	POS	POS	POS	POS	NEG	NEG	POS	POS	POS	NEG	NEG	NEG
Iasi-369	SSTI	POS	POS	NEG	POS	POS	POS	NEG	NEG	NEG	POS	POS	AMB	AMB	NEG	NEG	NEG	POS	POS	NEG	AMB	POS	POS	NEG	POS		POS	POS	NEG	POS	NEG	NEG	POS	POS	POS	POS	NEG	NEG	POS	POS	POS	NEG	NEG	NEG
Iasi-03-S11	Carrier	POS	POS	NEG	POS	POS	POS	NEG	NEG	NEG	POS	POS	AMB	AMB	NEG	NEG	NEG	POS	POS	NEG	POS	POS	POS	NEG	POS		POS	POS	NEG	POS	NEG	NEG	POS	POS	POS	POS	NEG	NEG	POS	POS	POS	NEG	NEG	NEG
Iasi-19-S35	Carrier	POS	POS	AMB	POS	POS	POS	NEG	NEG	NEG	POS	POS	AMB	AMB	NEG	NEG	NEG	POS	POS	NEG	POS	POS	POS	AMB	POS		POS	POS	AMB	POS	NEG	NEG	POS	POS	POS	POS	NEG	NEG	POS	POS	POS	NEG	NEG	NEG
Iasi-20-P1	Carrier	POS	POS	NEG	POS	POS	POS	NEG	NEG	NEG	POS	POS	AMB	AMB	NEG	NEG	NEG	POS	POS	NEG	AMB	POS	POS	NEG	POS		POS	POS	NEG	POS	NEG	NEG	POS	POS	POS	POS	NEG	NEG	POS	POS	POS	NEG	NEG	NEG
Iasi-35-P24	Carrier	POS	POS	NEG	POS	POS	POS	NEG	NEG	NEG	POS	POS	AMB	AMB	NEG	NEG	NEG	POS	POS	NEG	AMB	POS	POS	NEG	POS		POS	POS	NEG	POS	NEG	NEG	POS	POS	POS	POS	NEG	NEG	POS	POS	POS	NEG	NEG	NEG
Iasi-36-P25	Carrier	POS	POS	NEG	POS	POS	POS	NEG	NEG	NEG	POS	POS	AMB	AMB	NEG	NEG	NEG	POS	POS	NEG	AMB	POS	POS	NEG	POS		POS	POS	AMB	POS	NEG	NEG	POS	POS	POS	POS	NEG	NEG	POS	POS	POS	NEG	NEG	NEG
Iasi-14-150	SSTI	POS	POS	NEG	POS	POS	POS	NEG	NEG	NEG	POS	POS	AMB	AMB	NEG	NEG	NEG	POS	POS	NEG	NEG	POS	POS	NEG	POS		POS	POS	NEG	POS	NEG	NEG	POS	POS	POS	POS	NEG	NEG	POS	POS	POS	NEG	NEG	NEG
Iasi-07-54	BSI	POS	POS	NEG	POS	POS	POS	NEG	NEG	NEG	NEG	NEG	NEG	NEG	NEG	NEG	NEG	POS	POS	NEG	AMB	POS	POS	NEG	POS	NEG	POS	POS	NEG	POS	NEG	NEG	POS	POS	POS	POS	NEG	NEG	POS	POS	POS	NEG	NEG	NEG
Iasi-16-188	BSI	POS	POS	AMB	POS	POS	POS	NEG	NEG	NEG	NEG	NEG	NEG	NEG	NEG	NEG	NEG	POS	POS	NEG	AMB	POS	POS	NEG	POS	NEG	POS	POS	NEG	POS	NEG	NEG	POS	POS	POS	POS	NEG	NEG	POS	POS	POS	NEG	NEG	NEG
Iasi-32-318	BSI	POS	POS	NEG	POS	POS	POS	NEG	NEG	NEG	NEG	NEG	NEG	NEG	NEG	NEG	NEG	POS	POS	NEG	NEG	POS	POS	NEG	POS	NEG	POS	POS	NEG	POS	NEG	NEG	POS	POS	POS	POS	NEG	NEG	POS	POS	POS	NEG	NEG	NEG
Iasi-42-397	BSI	POS	POS	NEG	POS	POS	POS	NEG	NEG	NEG	NEG	NEG	NEG	NEG	NEG	NEG	NEG	POS	POS	NEG	NEG	POS	POS	NEG	POS	NEG	POS	POS	NEG	POS	NEG	NEG	POS	POS	POS	POS	NEG	NEG	POS	POS	POS	NEG	NEG	NEG
Iasi-48-408	BSI	POS	POS	NEG	POS	POS	POS	NEG	NEG	NEG	NEG	NEG	NEG	NEG	NEG	NEG	NEG	POS	POS	NEG	NEG	POS	POS	NEG	POS	NEG	POS	POS	NEG	POS	NEG	NEG	POS	POS	POS	POS	NEG	NEG	POS	POS	POS	NEG	NEG	NEG
Iasi-49-430	BSI	POS	POS	AMB	POS	POS	POS	NEG	NEG	NEG	NEG	NEG	NEG	NEG	NEG	NEG	NEG	POS	POS	NEG	AMB	POS	POS	AMB	POS	NEG	POS	POS	NEG	POS	NEG	NEG	POS	POS	POS	POS	NEG	NEG	POS	POS	POS	NEG	NEG	NEG
Iasi-52-52	BSI	POS	POS	NEG	POS	POS	POS	NEG	NEG	NEG	NEG	NEG	NEG	NEG	NEG	NEG	NEG	POS	POS	NEG	AMB	POS	POS	NEG	POS	NEG	POS	POS	NEG	POS	NEG	NEG	POS	POS	POS	POS	NEG	NEG	POS	POS	POS	NEG	NEG	NEG
Iasi-01-2	SSTI	POS	POS	NEG	POS	POS	POS	NEG	NEG	NEG	NEG	NEG	NEG	NEG	NEG	NEG	NEG	NEG	NEG	NEG	NEG	POS	POS	NEG	POS	NEG	POS	POS	NEG	POS	NEG	NEG	POS	POS	POS	POS	NEG	NEG	POS	POS	POS	NEG	NEG	NEG
Iasi-10-101	SSTI	POS	POS	NEG	POS	POS	POS	NEG	NEG	NEG	NEG	NEG	NEG	NEG	NEG	NEG	NEG	POS	POS	NEG	NEG	POS	POS	NEG	POS	NEG	POS	POS	NEG	POS	NEG	NEG	POS	POS	POS	POS	NEG	NEG	POS	POS	POS	NEG	NEG	NEG
Iasi-11-106	SSTI	POS	POS	NEG	POS	POS	POS	NEG	NEG	NEG	NEG	NEG	NEG	NEG	NEG	NEG	NEG	POS	POS	NEG	NEG	POS	POS	NEG	POS	NEG	POS	POS	NEG	POS	NEG	NEG	POS	POS	POS	POS	NEG	NEG	POS	POS	POS	NEG	NEG	NEG
Iasi-49-414	SSTI	POS	POS	AMB	POS	POS	POS	NEG	NEG	NEG	NEG	NEG	NEG	NEG	NEG	NEG	NEG	POS	POS	NEG	AMB	POS	POS	NEG	POS	NEG	POS	POS	NEG	POS	NEG	NEG	POS	POS	POS	POS	NEG	NEG	POS	POS	POS	NEG	NEG	NEG

Isolate	Group	eno	fib	fib (MRSA252)	fnbA (total)	fnbA (consensus)	fnbA (COL)	fnbA (MRSA252)	fnbA (Mu50+MW2)	fnbA (RF122)	fnbB (total)	fnbB (COL)	fnbB (COL+Mu50+MW2)	fnbB (Mu50)	fnbB (MW2)	fnbB (ST15)	fnbB (ST45-2)	map (total)	map (COL)	map (MRSA252)	map (Mu50+MW2)	sasG (total)	sasG (COL+Mu50)	sasG (MW2)	sasG (other than MRSA252+RF122)	sasX	sdrC (total)	sdrC (consensus)	sdrC (B1)	sdrC (COL)	sdrC (Mu50)	sdrC (MW2+MRSA252+RF122)	sdrC (other than MRSA252+RF122)	sdrD (total)	sdrD (consensus)	sdrD (COL+MW2)	sdrD (Mu50)	sdrD (other)	vwb (total)	vwb (consensus)	vwb (COL+MW2)	vwb (MRSA252)	vwb (Mu50)	vwb (RF122)
		ADHAESION FACTORS / GENES ENCODING MICROBIAL SURFACE COMPONENTS RECOGNIZING ADHESIVE MATRIX MOLECULES (MSCRAMM GENES)																																										
		enolase	fibrinogen binding protein (19 kDa)		fibronectin-binding protein A						fibronectin-binding protein B							Major histocompatibility complex class II analog protein (=Extracellular adherence protein, eap)				Staphylococcus aureus surface protein G					Ser-Asp rich fibrinogen-/bone sialoprotein-binding protein C							Ser-Asp rich fibrinogen-/bone sialoprotein-binding protein D					van Willebrand factor binding protein					
Iasi-05-46	misc. MRSA	POS	POS	NEG	POS	POS	POS	NEG	NEG	NEG	NEG	NEG	NEG	NEG	NEG	NEG	NEG	POS	POS	NEG	AMB	POS	POS	NEG	POS	NEG	POS	POS	NEG	POS	NEG	NEG	POS	POS	POS	POS	NEG	NEG	POS	POS	POS	NEG	NEG	NEG
Iasi-06-47	misc. MRSA	POS	POS	AMB	POS	POS	POS	NEG	NEG	NEG	NEG	NEG	NEG	NEG	NEG	NEG	NEG	POS	POS	NEG	AMB	POS	POS	NEG	POS	NEG	POS	POS	NEG	POS	NEG	NEG	POS	POS	POS	POS	NEG	NEG	POS	POS	POS	NEG	NEG	NEG
Iasi-08-73	misc. MRSA	POS	POS	NEG	POS	POS	POS	NEG	NEG	NEG	NEG	NEG	NEG	NEG	NEG	NEG	NEG	POS	POS	NEG	AMB	POS	POS	NEG	POS	NEG	POS	POS	NEG	POS	NEG	NEG	POS	POS	POS	POS	NEG	NEG	POS	POS	POS	NEG	NEG	NEG
Iasi-19-194	misc. MRSA	POS	POS	NEG	POS	POS	POS	NEG	NEG	NEG	NEG	NEG	NEG	NEG	NEG	NEG	NEG	POS	POS	NEG	AMB	POS	POS	NEG	POS	NEG	POS	POS	NEG	POS	NEG	NEG	POS	POS	POS	POS	NEG	NEG	POS	POS	POS	NEG	NEG	NEG
Iasi-21-219	misc. MRSA	POS	POS	NEG	POS	POS	POS	NEG	NEG	NEG	NEG	NEG	NEG	NEG	NEG	NEG	NEG	POS	POS	NEG	AMB	POS	POS	NEG	POS	NEG	POS	POS	NEG	POS	NEG	NEG	POS	POS	POS	POS	NEG	NEG	POS	POS	POS	NEG	NEG	NEG
Iasi-25-284	misc. MRSA	POS	POS	NEG	POS	POS	POS	NEG	NEG	NEG	NEG	NEG	NEG	NEG	NEG	NEG	NEG	POS	POS	NEG	AMB	POS	POS	NEG	POS	NEG	POS	POS	NEG	POS	NEG	NEG	POS	POS	POS	POS	NEG	NEG	POS	POS	POS	NEG	NEG	NEG
Iasi-284	misc. MRSA	POS	POS	NEG	POS	POS	POS	NEG	NEG	NEG	NEG	NEG	NEG	NEG	NEG	NEG	NEG	POS	POS	NEG	AMB	POS	POS	NEG	POS	NEG	POS	POS	NEG	POS	NEG	NEG	POS	POS	POS	POS	NEG	NEG	POS	POS	POS	NEG	NEG	NEG
Iasi-36-336	misc. MRSA	POS	POS	NEG	POS	POS	POS	NEG	NEG	NEG	NEG	NEG	NEG	NEG	NEG	NEG	NEG	POS	POS	NEG	NEG	POS	POS	NEG	POS	NEG	POS	POS	NEG	POS	NEG	NEG	POS	POS	POS	POS	NEG	NEG	POS	POS	POS	NEG	NEG	NEG
Iasi-43-402	misc. MRSA	POS	POS	NEG	POS	POS	POS	NEG	NEG	NEG	NEG	NEG	NEG	NEG	NEG	NEG	NEG	POS	POS	NEG	AMB	POS	POS	NEG	POS	NEG	POS	POS	NEG	POS	NEG	NEG	POS	POS	POS	POS	NEG	NEG	POS	POS	POS	NEG	NEG	NEG
Iasi-44-404	misc. MRSA	POS	POS	NEG	POS	POS	POS	NEG	NEG	NEG	NEG	NEG	NEG	NEG	NEG	NEG	NEG	POS	POS	NEG	NEG	POS	POS	NEG	POS	NEG	POS	POS	NEG	POS	NEG	NEG	POS	POS	POS	POS	NEG	NEG	POS	POS	POS	NEG	NEG	NEG
Iasi-54-430	misc. MRSA	POS	POS	NEG	POS	POS	POS	NEG	NEG	NEG	POS	NEG	NEG	NEG	POS	NEG	NEG	POS	POS	NEG	POS	POS	POS	AMB	POS	NEG	POS	POS	AMB	POS	NEG	AMB	POS	POS	POS	POS	NEG	NEG	POS	POS	POS	NEG	NEG	NEG
Iasi-24-216	SSTI	POS	NEG	AMB	POS	POS	NEG	NEG	POS	NEG	POS	NEG	POS	AMB	NEG	NEG	NEG	POS	POS	POS	POS	NEG	NEG	NEG	NEG		POS	POS	NEG	NEG	NEG	NEG	NEG	NEG	NEG	NEG	NEG	NEG	POS	POS	NEG	NEG	NEG	POS
Iasi-05-S13	Carrier	POS	NEG	AMB	POS	POS	NEG	NEG	POS	NEG	POS	NEG	AMB	POS	NEG	NEG	NEG	POS	POS	POS	POS	NEG	NEG	NEG	NEG		POS	POS	NEG	NEG	NEG	NEG	NEG	NEG	NEG	NEG	NEG	NEG	POS	POS	NEG	NEG	NEG	POS
Iasi-45-362	BSI	POS	POS	NEG	POS	POS	POS	NEG	NEG	NEG	POS	NEG	AMB	POS	NEG	AMB	NEG	POS	POS	NEG	POS	POS	NEG	POS	POS		POS	POS	NEG	POS	NEG	NEG	POS	POS	POS	NEG	POS	NEG	POS	POS	NEG	NEG	NEG	NEG
Iasi-22-199	SSTI	POS	POS	NEG	POS	POS	POS	NEG	NEG	NEG	POS	NEG	AMB	POS	NEG	AMB	NEG	POS	POS	NEG	POS	POS	NEG	POS	POS		POS	POS	NEG	POS	NEG	NEG	POS	POS	POS	NEG	POS	NEG	POS	POS	NEG	NEG	NEG	NEG
Iasi-12-S26	Carrier	POS	POS	NEG	POS	POS	POS	NEG	NEG	NEG	POS	NEG	AMB	AMB	NEG	POS	NEG	POS	POS	NEG	POS	POS	NEG	POS	POS		POS	POS	NEG	POS	NEG	NEG	POS	POS	POS	NEG	POS	NEG	POS	POS	NEG	NEG	NEG	NEG
Iasi-21-P2	Carrier	POS	POS	NEG	POS	POS	POS	NEG	NEG	NEG	POS	NEG	AMB	POS	NEG	AMB	NEG	POS	POS	NEG	POS	POS	NEG	POS	POS		POS	POS	NEG	POS	NEG	NEG	POS	POS	POS	NEG	POS	NEG	POS	POS	NEG	NEG	NEG	NEG
Iasi-06-60	BSI	POS	NEG	POS	POS	POS	NEG	NEG	POS	NEG	POS	NEG	AMB	NEG	POS	NEG	NEG	POS	NEG	NEG	POS	POS	NEG	POS	POS		POS	POS	NEG	NEG	POS	NEG	POS	POS	POS	NEG	NEG	POS	POS	POS	NEG	NEG	NEG	POS
Iasi-12-79	BSI	POS	NEG	POS	POS	POS	NEG	NEG	POS	NEG	POS	NEG	POS	NEG	AMB	NEG	NEG	POS	NEG	NEG	POS	POS	NEG	POS	POS		POS	POS	NEG	NEG	POS	NEG	POS	POS	POS	NEG	NEG	POS	POS	POS	NEG	NEG	NEG	POS
Iasi-17-90	BSI	POS	NEG	POS	POS	POS	NEG	NEG	POS	NEG	POS	NEG	POS	NEG	AMB	NEG	NEG	POS	NEG	NEG	POS	POS	NEG	POS	POS		POS	POS	NEG	NEG	POS	NEG	POS	POS	POS	NEG	NEG	POS	POS	POS	NEG	NEG	NEG	POS
Iasi-21-105	BSI	POS	NEG	POS	POS	POS	NEG	NEG	POS	NEG	NEG	NEG	NEG	NEG	NEG	NEG	NEG	POS	NEG	NEG	POS	POS	NEG	POS	POS		POS	POS	NEG	NEG	POS	NEG	POS	POS	POS	NEG	NEG	POS	POS	POS	NEG	NEG	NEG	POS
Iasi-22-109	BSI	POS	NEG	POS	POS	POS	NEG	NEG	POS	NEG	POS	AMB	AMB	AMB	POS	NEG	NEG	POS	AMB	NEG	POS	POS	NEG	POS	POS		POS	POS	AMB	NEG	POS	AMB	POS	POS	POS	NEG	NEG	POS	POS	POS	NEG	NEG	NEG	POS
Iasi-23-110	BSI	POS	NEG	POS	POS	POS	NEG	NEG	POS	NEG	POS	NEG	AMB	NEG	POS	NEG	NEG	POS	NEG	NEG	POS	POS	NEG	POS	POS		POS	POS	NEG	NEG	POS	NEG	POS	POS	POS	NEG	NEG	POS	POS	POS	NEG	NEG	NEG	POS
Iasi-24-136	BSI	POS	NEG	POS	POS	POS	NEG	NEG	POS	NEG	NEG	NEG	NEG	NEG	NEG	NEG	NEG	POS	NEG	NEG	POS	POS	NEG	POS	POS		POS	POS	NEG	NEG	POS	NEG	POS	POS	POS	NEG	NEG	POS	POS	POS	NEG	NEG	NEG	POS
Iasi-34-224	BSI	POS	NEG	POS	POS	POS	NEG	NEG	POS	NEG	NEG	NEG	NEG	NEG	NEG	NEG	NEG	POS	NEG	NEG	POS	POS	NEG	POS	POS		POS	POS	NEG	NEG	POS	NEG	POS	POS	POS	NEG	NEG	POS	POS	POS	NEG	NEG	NEG	POS
Iasi-39-297	BSI	POS	NEG	POS	POS	POS	NEG	NEG	POS	NEG	POS	NEG	AMB	AMB	POS	NEG	NEG	POS	NEG	NEG	POS	POS	NEG	POS	POS		POS	POS	NEG	NEG	POS	AMB	POS	POS	POS	NEG	NEG	POS	POS	POS	NEG	NEG	NEG	POS
Iasi-50-431	BSI	POS	NEG	POS	POS	POS	NEG	NEG	POS	NEG	POS	NEG	POS	NEG	AMB	NEG	NEG	POS	NEG	NEG	POS	POS	NEG	POS	POS		POS	POS	NEG	NEG	POS	NEG	POS	POS	POS	NEG	NEG	POS	POS	POS	NEG	NEG	NEG	POS
Iasi-51-432	BSI	POS	NEG	POS	POS	POS	NEG	NEG	POS	NEG	POS	NEG	NEG	POS	NEG	NEG	NEG	POS	NEG	NEG	POS	POS	NEG	POS	POS		POS	POS	NEG	NEG	POS	NEG	POS	POS	POS	NEG	NEG	POS	POS	POS	NEG	NEG	NEG	POS
Iasi-08-76	SSTI	POS	NEG	POS	POS	POS	NEG	NEG	POS	NEG	POS	NEG	POS	NEG	AMB	NEG	NEG	POS	NEG	NEG	POS	POS	NEG	POS	POS		POS	POS	NEG	NEG	POS	NEG	POS	POS	POS	NEG	NEG	POS	POS	POS	NEG	NEG	NEG	POS
Iasi-30-350	SSTI	POS	NEG	POS	POS	POS	NEG	NEG	POS	NEG	POS	NEG	POS	NEG	AMB	NEG	NEG	POS	NEG	NEG	POS	POS	NEG	POS	POS		POS	POS	NEG	NEG	POS	NEG	POS	POS	POS	NEG	NEG	POS	POS	POS	NEG	NEG	NEG	POS
Iasi-33-319	SSTI	POS	NEG	POS	POS	POS	NEG	NEG	POS	NEG	POS	NEG	AMB	NEG	POS	NEG	NEG	NEG	NEG	NEG	AMB	POS	NEG	POS	POS		POS	POS	NEG	NEG	POS	NEG	POS	POS	POS	NEG	NEG	POS	POS	POS	NEG	NEG	NEG	POS
Iasi-40-383	SSTI	POS	NEG	POS	POS	POS	NEG	NEG	POS	NEG	POS	NEG	AMB	NEG	POS	NEG	NEG	POS	NEG	NEG	POS	POS	NEG	POS	POS		POS	POS	NEG	NEG	POS	NEG	POS	POS	POS	NEG	NEG	POS	POS	POS	NEG	NEG	NEG	POS
Iasi-49-423	SSTI	POS	NEG	POS	POS	POS	NEG	NEG	POS	NEG	POS	NEG	POS	NEG	AMB	NEG	NEG	POS	NEG	NEG	POS	POS	NEG	POS	POS		POS	POS	NEG	NEG	POS	NEG	POS	POS	POS	NEG	NEG	POS	POS	POS	NEG	NEG	NEG	POS
Iasi-16-S31	Carrier	POS	NEG	POS	POS	POS	NEG	NEG	POS	NEG	POS	NEG	AMB	NEG	NEG	NEG	NEG	POS	NEG	NEG	POS	POS	NEG	POS	POS		POS	POS	NEG	NEG	POS	NEG	POS	POS	POS	NEG	NEG	POS	POS	POS	NEG	NEG	NEG	POS
Iasi-24-P9	Carrier	POS	NEG	POS	POS	POS	NEG	NEG	POS	NEG	POS	NEG	NEG	NEG	POS	NEG	NEG	POS	NEG	NEG	POS	POS	NEG	POS	POS		POS	POS	NEG	NEG	POS	NEG	POS	POS	POS	NEG	NEG	POS	POS	POS	NEG	NEG	NEG	POS
Iasi-27-P12	Carrier	POS	NEG	POS	POS	POS	NEG	NEG	POS	NEG	POS	NEG	AMB	NEG	POS	NEG	NEG	POS	NEG	NEG	POS	POS	NEG	POS	POS		POS	POS	NEG	NEG	POS	NEG	POS	POS	POS	NEG	NEG	POS	POS	POS	NEG	NEG	NEG	POS
Iasi-38-P27	Carrier	POS	NEG	POS	POS	POS	NEG	NEG	POS	NEG	POS	NEG	AMB	NEG	POS	NEG	NEG	POS	NEG	NEG	POS	POS	NEG	POS	POS		POS	POS	NEG	NEG	POS	NEG	POS	POS	POS	NEG	NEG	POS	POS	POS	NEG	NEG	NEG	POS
Iasi-42-G511	Carrier	POS	NEG	POS	POS	POS	NEG	NEG	POS	NEG	POS	NEG	AMB	NEG	POS	NEG	NEG	POS	NEG	NEG	POS	POS	NEG	POS	POS		POS	POS	NEG	NEG	POS	NEG	POS	POS	POS	NEG	NEG	POS	POS	POS	NEG	NEG	NEG	POS
Iasi-46-3N	Carrier	POS	NEG	POS	NEG	NEG	NEG	NEG	NEG	NEG	POS	NEG	AMB	NEG	POS	NEG	NEG	POS	NEG	NEG	POS	POS	NEG	POS	POS		POS	POS	NEG	NEG	POS	NEG	POS	POS	POS	NEG	NEG	POS	POS	POS	NEG	NEG	NEG	POS
Iasi-47-42O	Carrier	POS	NEG	POS	POS	POS	NEG	NEG	POS	NEG	POS	NEG	AMB	NEG	POS	NEG	NEG	POS	NEG	NEG	POS	POS	NEG	POS	POS		POS	POS	NEG	NEG	POS	NEG	POS	POS	POS	NEG	NEG	POS	POS	POS	NEG	NEG	NEG	POS
Iasi-48-36N	Carrier	POS	NEG	POS	POS	POS	NEG	NEG	POS	NEG	POS	NEG	AMB	NEG	POS	NEG	NEG	POS	NEG	NEG	POS	POS	NEG	POS	POS		POS	POS	NEG	NEG	POS	NEG	POS	POS	POS	NEG	NEG	POS	POS	POS	NEG	NEG	NEG	POS
Iasi-49-T3	Carrier	POS	NEG	POS	POS	POS	NEG	NEG	POS	NEG	POS	NEG	AMB	NEG	POS	NEG	NEG	POS	NEG	NEG	POS	POS	NEG	POS	POS		POS	POS	NEG	NEG	POS	NEG	POS	POS	POS	NEG	NEG	POS	POS	POS	NEG	NEG	NEG	POS
Iasi-50-T2	Carrier	POS	NEG	POS	POS	POS	NEG	NEG	POS	NEG	POS	NEG	AMB	NEG	POS	NEG	NEG	POS	NEG	NEG	POS	POS	NEG	POS	POS		POS	POS	NEG	NEG	POS	NEG	POS	POS	POS	NEG	NEG	POS	POS	POS	NEG	NEG	NEG	POS
Iasi-22-251	BSI	POS	NEG	POS	POS	POS	NEG	NEG	POS	NEG	NEG	NEG	NEG	NEG	NEG	NEG	NEG	POS	NEG	NEG	POS	POS	NEG	POS	POS		POS	POS	NEG	NEG	POS	NEG	POS	POS	POS	NEG	NEG	POS	POS	POS	NEG	NEG	NEG	POS
Iasi-27-158	BSI	POS	POS	NEG	POS	POS	NEG	NEG	POS	NEG	POS	NEG	POS	AMB	NEG	AMB	NEG	POS	POS	NEG	POS	NEG	NEG	NEG	NEG		POS	POS	AMB	NEG	NEG	POS	POS	POS	POS	POS	NEG	AMB	POS	POS	NEG	NEG	NEG	NEG
Iasi-08-70	BSI	POS	NEG	POS	POS	POS	NEG	POS	NEG	NEG	NEG	NEG	NEG	NEG	NEG	NEG	NEG	POS	NEG	POS	NEG	NEG	NEG	NEG	NEG		POS	POS	NEG	NEG	NEG	AMB	NEG	NEG	NEG	NEG	NEG	NEG	POS	POS	NEG	POS	NEG	NEG
Iasi-18-93	BSI	POS	NEG	POS	POS	POS	NEG	POS	NEG	NEG	NEG	NEG	NEG	NEG	NEG	NEG	NEG	POS	NEG	POS	NEG	NEG	NEG	NEG	NEG		POS	POS	NEG	NEG	NEG	AMB	NEG	POS	POS	NEG	NEG	POS	POS	POS	NEG	POS	NEG	NEG
Iasi-28-P14	Carrier	POS	NEG	POS	POS	POS	NEG	POS	NEG	NEG	NEG	NEG	NEG	NEG	NEG	NEG	NEG	POS	NEG	POS	NEG	NEG	NEG	NEG	NEG		POS	POS	NEG	NEG	NEG	AMB	NEG	POS	POS	NEG	NEG	POS	POS	POS	NEG	POS	NEG	NEG
Iasi-29-P15	Carrier	POS	NEG	POS	POS	POS	NEG	POS	NEG	NEG	NEG	NEG	NEG	NEG	NEG	NEG	NEG	POS	NEG	POS	NEG	NEG	NEG	NEG	NEG		POS	POS	NEG	NEG	NEG	POS	NEG	POS	POS	NEG	NEG	POS	POS	POS	NEG	POS	NEG	NEG
Iasi-03-19	BSI	POS	NEG	POS	POS	POS	NEG	NEG	POS	NEG	POS	NEG	POS	AMB	NEG	NEG	NEG	POS	NEG	POS	NEG	NEG	NEG	NEG	NEG		POS	POS	POS	NEG	NEG	NEG	NEG	POS	POS	POS	NEG	NEG	POS	NEG	NEG	POS	NEG	NEG
Iasi-11-77	BSI	POS	NEG	POS	POS	POS	NEG	NEG	POS	NEG	POS	NEG	POS	AMB	NEG	NEG	NEG	POS	NEG	POS	NEG	NEG	NEG	NEG	NEG		POS	POS	POS	NEG	NEG	NEG	NEG	NEG	NEG	NEG	NEG	NEG	POS	NEG	NEG	POS	NEG	NEG
Iasi-13-82	BSI	POS	NEG	POS	POS	POS	NEG	NEG	POS	NEG	POS	NEG	POS	AMB	NEG	NEG	NEG	POS	NEG	POS	NEG	NEG	NEG	NEG	NEG		POS	POS	POS	NEG	NEG	NEG	NEG	POS	POS	POS	NEG	NEG	POS	NEG	NEG	POS	NEG	NEG
Iasi-15-86	BSI	POS	NEG	POS	POS	POS	NEG	NEG	POS	NEG	POS	NEG	AMB	POS	NEG	AMB	NEG	POS	NEG	POS	NEG	NEG	NEG	NEG	NEG		POS	POS	POS	NEG	NEG	AMB	NEG	NEG	NEG	NEG	NEG	NEG	POS	AMB	NEG	POS	NEG	NEG
Iasi-20-103	BSI	POS	NEG	POS	POS	POS	NEG	NEG	POS	NEG	POS	NEG	AMB	POS	NEG	AMB	NEG	POS	NEG	POS	NEG	NEG	NEG	NEG	NEG		POS	POS	POS	NEG	NEG	NEG	NEG	POS	POS	POS	NEG	NEG	POS	NEG	NEG	POS	NEG	NEG
Iasi-33-222	BSI	POS	NEG	POS	POS	POS	NEG	NEG	POS	NEG	POS	NEG	AMB	POS	NEG	AMB	NEG	POS	NEG	POS	NEG	NEG	NEG	NEG	NEG		POS	POS	POS	NEG	NEG	AMB	NEG	POS	POS	POS	NEG	NEG	POS	AMB	NEG	POS	NEG	NEG
Iasi-44-360	BSI	POS	NEG	POS	POS	POS	NEG	NEG	POS	NEG	POS	NEG	POS	AMB	NEG	NEG	NEG	POS	NEG	POS	NEG	NEG	NEG	NEG	NEG		POS	POS	POS	NEG	NEG	NEG	NEG	POS	POS	POS	NEG	NEG	POS	AMB	NEG	POS	NEG	NEG
Iasi-47-399	BSI	POS	NEG	POS	POS	POS	NEG	NEG	POS	NEG	POS	NEG	AMB	POS	NEG	AMB	NEG	POS	NEG	POS	NEG	NEG	NEG	NEG	NEG		POS	POS	POS	NEG	NEG	AMB	NEG	NEG	NEG	NEG	NEG	NEG	POS	AMB	NEG	POS	NEG	NEG
Iasi-34-368	SSTI	POS	NEG	POS	POS	POS	NEG	NEG	POS	NEG	POS	NEG	POS	AMB	NEG	AMB	NEG	POS	NEG	POS	NEG	NEG	NEG	NEG	NEG		POS	POS	POS	NEG	NEG	AMB	NEG	POS	POS	POS	NEG	NEG	POS	AMB	NEG	POS	NEG	NEG
Iasi-36-371	SSTI	POS	NEG	POS	POS	POS	NEG	NEG	POS	NEG	POS	NEG	POS	AMB	NEG	AMB	NEG	POS	NEG	POS	NEG	NEG	NEG	NEG	NEG		POS	POS	POS	NEG	NEG	AMB	NEG	POS	POS	POS	NEG	NEG	POS	NEG	NEG	POS	NEG	NEG
Iasi-04-S12	Carrier	POS	NEG	POS	POS	POS	NEG	NEG	POS	NEG	POS	NEG	POS	AMB	NEG	AMB	NEG	POS	NEG	POS	NEG	NEG	NEG	NEG	NEG		POS	POS	POS	NEG	NEG	AMB	NEG	POS	POS	POS	NEG	NEG	POS	AMB	NEG	POS	NEG	NEG
Iasi-06-S15	Carrier	POS	NEG	POS	POS	POS	NEG	NEG	POS	NEG	POS	NEG	POS	AMB	NEG	AMB	NEG	POS	NEG	POS	NEG	NEG	NEG	NEG	NEG		POS	POS	POS	NEG	NEG	AMB	NEG	POS	POS	POS	NEG	NEG	POS	AMB	NEG	POS	NEG	NEG
Iasi-07-S16	Carrier	POS	NEG	POS	POS	POS	NEG	NEG	POS	NEG	POS	NEG	AMB	POS	NEG	AMB	NEG	POS	NEG	POS	NEG	NEG	NEG	NEG	NEG		POS	POS	POS	NEG	NEG	AMB	NEG	POS	POS	POS	NEG	NEG	POS	AMB	NEG	POS	NEG	NEG
Iasi-11-S24	Carrier	POS	NEG	POS	POS	POS	NEG	NEG	POS	NEG	POS	NEG	POS	AMB	NEG	AMB	NEG	POS	NEG	POS	NEG	NEG	NEG	NEG	NEG		POS	POS	POS	NEG	NEG	AMB	NEG	POS	POS	POS	NEG	NEG	POS	POS	NEG	POS	NEG	NEG
Iasi-22-P4	Carrier	POS	NEG	POS	POS	POS	NEG	NEG	POS	NEG	POS	NEG	AMB	POS	NEG	AMB	NEG	POS	NEG	POS	NEG	NEG	NEG	NEG	NEG		POS	POS	POS	NEG	NEG	AMB	NEG	NEG	NEG	NEG	NEG	NEG	POS	AMB	NEG	POS	NEG	NEG
Iasi-34-P23	Carrier	POS	NEG	POS	POS	POS	NEG	NEG	POS	NEG	POS	NEG	AMB	POS	NEG	AMB	NEG	POS	NEG	POS	NEG	NEG	NEG	NEG	NEG		POS	POS	POS	NEG	NEG	AMB	NEG	NEG	NEG	NEG	NEG	NEG	POS	AMB	NEG	POS	NEG	NEG
Iasi-37-P26	Carrier	POS	NEG	POS	POS	POS	NEG	NEG	POS	NEG	POS	NEG	POS	AMB	NEG	AMB	NEG	POS	NEG	POS	NEG	NEG	NEG	NEG	NEG		POS	POS	POS	NEG	NEG	POS	NEG	NEG	NEG	NEG	NEG	NEG	POS	POS	NEG	POS	NEG	NEG
Iasi-30-196	BSI	POS	NEG	POS	POS	POS	NEG	NEG	POS	NEG	POS	NEG	POS	AMB	NEG	AMB	NEG	POS	POS	POS	AMB	NEG	NEG	NEG	NEG		POS	POS	POS	AMB	NEG	POS	NEG	NEG	NEG	NEG	NEG	NEG	POS	POS	NEG	POS	NEG	NEG
Iasi-27-298	BSI	POS	POS	NEG	POS	POS	NEG	NEG	NEG	NEG	POS	NEG	AMB	NEG	POS	NEG	NEG	POS	NEG	NEG	POS	POS	POS	NEG	POS		POS	POS	NEG	POS	NEG	NEG	POS	POS	POS	NEG	NEG	POS	POS	POS	NEG	POS	NEG	NEG
Iasi-37-363	BSI	POS	POS	NEG	POS	POS	NEG	NEG	NEG	NEG	POS	NEG	AMB	NEG	POS	NEG	NEG	POS	NEG	NEG	POS	POS	POS	NEG	POS		POS	POS	NEG	POS	NEG	NEG	POS	POS	POS	NEG	NEG	POS	POS	POS	NEG	POS	NEG	NEG
Iasi-38-372	BSI	POS	POS	NEG	POS	POS	NEG	NEG	NEG	NEG	POS	NEG	AMB	NEG	POS	NEG	NEG	POS	NEG	NEG	POS	POS	POS	NEG	POS		POS	POS	NEG	POS	NEG	NEG	POS	POS	POS	NEG	NEG	POS	POS	POS	NEG	NEG	NEG	NEG
Iasi-39-374	BSI	POS	POS	NEG	POS	POS	NEG	NEG	NEG	NEG	POS	NEG	AMB	NEG	POS	NEG	NEG	POS	POS	NEG	POS	POS	POS	NEG	POS		POS	POS	NEG	POS	NEG	NEG	POS	POS	POS	NEG	NEG	POS	POS	POS	NEG	POS	NEG	NEG
Iasi-28-299	SSTI	POS	POS	NEG	POS	POS	NEG	NEG	NEG	NEG	POS	NEG	AMB	NEG	POS	NEG	NEG	POS	NEG	NEG	POS	POS	POS	NEG	POS		POS	POS	NEG	POS	NEG	NEG	POS	POS	POS	NEG	NEG	POS	POS	POS	NEG	POS	NEG	NEG
Iasi-07-65	BSI	POS	POS	NEG	POS	POS	NEG	NEG	NEG	NEG	POS	NEG	AMB	POS	NEG	NEG	NEG	POS	NEG	NEG	POS	POS	POS	NEG	POS		POS	POS	NEG	NEG	POS	POS	POS	POS	POS	NEG	NEG	POS	POS	POS	NEG	NEG	POS	NEG
Iasi-19-99	BSI	POS	POS	NEG	POS	POS	NEG	NEG	NEG	NEG	POS	NEG	AMB	POS	NEG	NEG	NEG	POS	POS	NEG	POS	POS	POS	POS	NEG		POS	POS	NEG	NEG	POS	POS	POS	POS	POS	NEG	POS	NEG	POS	POS	NEG	NEG	NEG	POS
Iasi-25-217	SSTI	POS	POS	NEG	POS	POS	NEG	NEG	NEG	NEG	POS	NEG	AMB	POS	NEG	NEG	NEG	POS	POS	AMB	POS	POS	POS	POS	NEG		POS	POS	NEG	NEG	POS	POS	POS	POS	POS	NEG	POS	NEG	POS	POS	NEG	NEG	NEG	POS
Iasi-23-P5	Carrier	POS	POS	NEG	POS	POS	NEG	NEG	NEG	NEG	POS	NEG	AMB	POS	NEG	NEG	NEG	POS	POS	NEG	POS	POS	POS	POS	NEG		POS	POS	NEG	NEG	POS	POS	POS	POS	POS	NEG	POS	NEG	POS	POS	NEG	NEG	NEG	POS
Iasi-43-G513	Carrier	POS	POS	NEG	POS	POS	NEG	NEG	NEG	NEG	POS	NEG	AMB	POS	NEG	NEG	NEG	POS	POS	NEG	POS	POS	POS	POS	NEG		POS	POS	NEG	NEG	POS	POS	POS	POS	POS	NEG	POS	NEG	POS	POS	NEG	NEG	NEG	POS
Iasi-44-G514	Carrier	POS	POS	NEG	POS	POS	NEG	NEG	NEG	NEG	POS	NEG	AMB	POS	NEG	NEG	NEG	POS	POS	NEG	POS	POS	POS	POS	NEG		POS	POS	AMB	NEG	POS	POS	POS	POS	POS	NEG	POS	NEG	POS	POS	NEG	NEG	NEG	POS
Iasi-28-172	BSI	POS	POS	AMB	POS	POS	NEG	NEG	NEG	NEG	POS	NEG	POS	AMB	NEG	NEG	NEG	POS	POS	NEG	POS	NEG	NEG	AMB	NEG		POS	POS	NEG	POS	NEG	AMB	NEG	POS	POS	NEG	POS	NEG	POS	POS	POS	NEG	NEG	NEG
Iasi-42-358	BSI	POS	POS	POS	POS	POS	NEG	NEG	NEG	NEG	POS	NEG	POS	AMB	NEG	NEG	NEG	POS	POS	NEG	POS	NEG	NEG	AMB	NEG		POS	POS	NEG	POS	NEG	AMB	NEG	POS	POS	NEG	POS	NEG	POS	POS	POS	NEG	NEG	NEG
Iasi-48-403	BSI	POS	POS	AMB	POS	POS	NEG	NEG	NEG	NEG	POS	NEG	POS	AMB	NEG	NEG	NEG	POS	POS	NEG	POS	NEG	NEG	AMB	NEG		POS	POS	NEG	POS	NEG	AMB	NEG	POS	POS	NEG	AMB	NEG	POS	POS	POS	NEG	NEG	NEG
Iasi-26-153	BSI	POS	POS	NEG	POS	POS	NEG	NEG	NEG	NEG	POS	NEG	POS	NEG	NEG	NEG	NEG	POS	POS	NEG	POS	NEG	NEG	NEG	NEG		POS	POS	NEG	POS	NEG	NEG	NEG	POS	POS	NEG	NEG	NEG	POS	POS	POS	NEG	NEG	NEG
Iasi-29-190	BSI	POS	POS	AMB	POS	POS	NEG	NEG	NEG	NEG	POS	NEG	POS	AMB	NEG	NEG	NEG	POS	POS	NEG	POS	NEG	NEG	AMB	NEG		POS	POS	NEG	POS	NEG	NEG	NEG	POS	POS	NEG	AMB	NEG	POS	POS	POS	NEG	NEG	NEG
Iasi-31-201	BSI	POS	POS	NEG	POS	POS	NEG	NEG	NEG	NEG	POS	NEG	POS	NEG	NEG	NEG	NEG	POS	POS	NEG	POS	NEG	NEG	NEG	NEG		POS	POS	NEG	POS	NEG	NEG	NEG	POS	POS	NEG	AMB	NEG	POS	POS	POS	NEG	NEG	NEG
Iasi-37-289	BSI	POS	POS	AMB	POS	POS	NEG	NEG	NEG	NEG	POS	NEG	POS	AMB	NEG	NEG	NEG	POS	POS	NEG	POS	NEG	NEG	AMB	NEG		POS	POS	NEG	POS	NEG	AMB	NEG	POS	POS	NEG	POS	NEG	POS	POS	POS	NEG	NEG	NEG
Iasi-41-334	BSI	POS	POS	POS	POS	POS	NEG	NEG	NEG	NEG	POS	NEG	POS	AMB	NEG	NEG	NEG	POS	POS	NEG	POS	NEG	NEG	AMB	NEG		POS	POS	NEG	POS	NEG	POS	NEG	POS	POS	NEG	POS	NEG	POS	POS	POS	NEG	NEG	NEG
Iasi-52-433	BSI	POS	POS	NEG	POS	POS	NEG	NEG	NEG	NEG	POS	NEG	POS	NEG	NEG	NEG	NEG	POS	POS	NEG	NEG	NEG	NEG	NEG	NEG		POS	POS	NEG	POS	NEG	NEG	NEG	POS	POS	NEG	NEG	NEG	POS	POS	POS	NEG	NEG	NEG
Iasi-07-66	SSTI	POS	POS	NEG	POS	POS	NEG	NEG	NEG	NEG	POS	NEG	POS	NEG	NEG	NEG	NEG	POS	POS	NEG	NEG	NEG	NEG	NEG	NEG		POS	POS	NEG	POS	NEG	NEG	NEG	POS	POS	NEG	NEG	NEG	POS	POS	POS	NEG	NEG	NEG
Iasi-23-211	SSTI	POS	POS	NEG	POS	POS	NEG	NEG	NEG	NEG	POS	NEG	POS	NEG	NEG	NEG	NEG	POS	POS	NEG	POS	NEG	NEG	NEG	NEG		POS	POS	NEG	POS	NEG	NEG	NEG	POS	POS	NEG	NEG	NEG	POS	POS	POS	NEG	NEG	NEG
Iasi-43-401	SSTI	POS	POS	NEG	POS	POS	NEG	NEG	NEG	NEG	POS	NEG	POS	NEG	NEG	NEG	NEG	POS	POS	NEG	AMB	NEG	NEG	NEG	NEG		POS	POS	NEG	POS	NEG	NEG	NEG	POS	POS	NEG	NEG	NEG	POS	POS	POS	NEG	NEG	NEG
Iasi-47-418	SSTI	POS	POS	NEG	POS	POS	NEG	NEG	NEG	NEG	POS	NEG	POS	NEG	NEG	NEG	NEG	POS	POS	NEG	AMB	NEG	NEG	NEG	NEG		POS	POS	NEG	POS	NEG	NEG	NEG	POS	POS	NEG	NEG	NEG	POS	POS	POS	NEG	NEG	NEG
Iasi-18-169	SSTI	POS	POS	NEG	POS	POS	NEG	NEG	NEG	NEG	POS	NEG	POS	NEG	NEG	NEG	NEG	POS	POS	NEG	POS	NEG	NEG	NEG	NEG		POS	POS	NEG	POS	NEG	NEG	NEG	POS	POS	NEG	POS	NEG	POS	POS	NEG	NEG	NEG	NEG
Iasi-18-S34	Carrier	POS	POS	NEG	POS	POS	NEG	NEG	NEG	NEG	POS	NEG	AMB	POS	NEG	AMB	NEG	POS	POS	NEG	POS	NEG	NEG	NEG	NEG		POS	POS	NEG	POS	NEG	NEG	NEG	POS	POS	NEG	POS	NEG	POS	POS	NEG	POS	NEG	NEG
Iasi-36-276	BSI	POS	NEG	POS	POS	POS	NEG	NEG	NEG	NEG	POS	NEG	POS	AMB	NEG	NEG	NEG	POS	POS	POS	AMB	NEG	NEG	NEG	NEG		POS	POS	NEG	NEG	POS	POS	NEG	POS	POS	POS	NEG	NEG	POS	POS	NEG	NEG	NEG	NEG
Iasi-151	BSI	POS	NEG	POS	POS	POS	NEG	NEG	NEG	NEG	POS	NEG	POS	AMB	NEG	NEG	NEG	POS	POS	POS	POS	NEG	NEG	AMB	NEG		POS	POS	NEG	NEG	POS	POS	NEG	POS	POS	NEG	AMB	NEG	POS	POS	NEG	NEG	NEG	NEG
Iasi-02-11	BSI	POS	POS	NEG	POS	POS	NEG	NEG	NEG	NEG	NEG	NEG	NEG	NEG	NEG	NEG	NEG	POS	POS	NEG	POS	POS	POS	NEG	POS		POS	POS	NEG	NEG	NEG	AMB	NEG	NEG	NEG	NEG	NEG	NEG	POS	POS	POS	NEG	NEG	NEG

Isolate	Group	IMMUNOD.AG.B		DEFENSIN RESIST.		TRANSFERRIN BINDING PROT			PUTATIVE TRANSPORTER				TYPE I RESTRICTION-MODIFICATION SYSTEM, SINGLE SEQUENCE SPECIFICITY PROTEIN					TYPE I RESTRICTION-MODIFICATION SYSTEM, SINGLE SEQUENCE SPECIFICITY PROTEIN								HYALURONATE LYASE								MISCELLANEOUS GENES		
		immunodominant antigen B		defensin resistance protein		transferrin-binding protein			hypothetical protein, similar to integral membrane protein LmrP				type I site-specific deoxyribo-nuclease subunit, 1st locus	type I site-specific deoxyribonuclease subunit, 2nd locus				type I site-specific deoxyribonuclease subunit, 3rd locus					type I site-specific deoxyribonuclease subunit, unknown locus			hyaluronate lyase, first / second locus			hyaluronate lyase, second locus					hypothetical protein, located next to serine protease operon	Unspecific efflux/trans-porter	hypothetical protein
		isaB	isaB (MRSA252)	mprF (COL+MW2)	mprF (Mu50+MRSA252)	isdA (consensus)	isdA (MRSA252)	isdA (other than MRSA252)	lmrP (other than RF122)_probe1	lmrP (other than RF122)_probe2	lmrP (RF122)_probe1	lmrP (RF122)_probe2	hsdS1 (RF122)	hsdS2 (Mu50+N315+COL+USA300+NCTC8325)	hsdS2 (MW2+MSSA476)	hsdS2 (RF122)	hsdS2 (MRSA252)	hsdS3 (all other than RF122+ MRSA252)	hsdS3 (COL+USA300+NCTC8325+MW2+MSSA476+RF122)	hsdS3 (Mu50+N315)	hsdS3 (CC51+ MRSA252)	hsdS3 (MRSA252)	hsdSx (CC25)	hsdSx (CC15)	hsdSx (etd)	hysA1 (MRSA252)	hysA1 (MRSA252+RF122) and/or hysA2 (consensus)	hysA1 (MRSA252+RF122) and/or hysA2 (COL+USA300)	hysA2 (all other than MRSA252)	hysA2 (COL+USA300+NCTC8325)	hysA2 (all other than COL+USA300+NCTC8325)	hysA2 (all other than COL+USA300+NCTC8325)	hysA2 (MRSA252)	Q2FXC0	Q2YUB3	Q7A4X2
Iasi-01-4	BSI	POS	AMB	POS	AMB	POS	NEG	POS	POS	POS	NEG	NEG	NEG	NEG	POS	NEG	NEG	POS	POS	NEG	NEG	NEG	POS	NEG	NEG	NEG	POS	NEG	POS	NEG	POS	POS	NEG	POS	NEG	NEG
Iasi-05-32	BSI	POS	AMB	POS	AMB	POS	NEG	POS	POS	POS	NEG	NEG	NEG	NEG	POS	NEG	NEG	POS	POS	NEG	NEG	NEG	POS	NEG	NEG	NEG	POS	NEG	POS	NEG	POS	POS	AMB	POS	NEG	NEG
Iasi-16-88	BSI	POS	AMB	POS	AMB	POS	NEG	POS	POS	POS	NEG	NEG	NEG	NEG	POS	NEG	NEG	POS	POS	NEG	AMB	NEG	POS	AMB	NEG	NEG	POS	NEG	POS	NEG	POS	POS	AMB	POS	AMB	NEG
Iasi-25-141	BSI	POS	AMB	POS	AMB	POS	AMB	POS	POS	POS	NEG	NEG	NEG	NEG	POS	NEG	NEG	POS	POS	NEG	AMB	NEG	POS	AMB	NEG	NEG	POS	NEG	POS	NEG	POS	POS	NEG	POS	AMB	NEG
Iasi-32-215	BSI	POS	AMB	AMB	POS	POS	AMB	POS	POS	POS	NEG	NEG	NEG	NEG	POS	NEG	NEG	POS	POS	NEG	AMB	NEG	POS	AMB	NEG	NEG	POS	NEG	POS	NEG	POS	POS	AMB	POS	AMB	NEG
Iasi-43-359	BSI	AMB	POS	POS	AMB	POS	NEG	POS	POS	POS	NEG	NEG	NEG	NEG	POS	NEG	NEG	POS	POS	NEG	NEG	NEG	POS	NEG	NEG	NEG	POS	NEG	POS	NEG	POS	POS	NEG	POS	NEG	NEG
Iasi-03-27	SSTI	POS	AMB	POS	AMB	POS	NEG	POS	POS	POS	NEG	NEG	NEG	NEG	POS	NEG	NEG	POS	POS	NEG	NEG	NEG	POS	NEG	NEG	NEG	POS	NEG	POS	NEG	POS	POS	NEG	POS	NEG	NEG
Iasi-13-149	SSTI	POS	AMB	POS	AMB	POS	NEG	POS	POS	POS	NEG	NEG	NEG	NEG	POS	NEG	NEG	POS	POS	NEG	NEG	NEG	POS	NEG	NEG	NEG	POS	NEG	POS	NEG	POS	POS	NEG	POS	NEG	NEG
Iasi-29-337	SSTI	POS	AMB	POS	AMB	POS	NEG	POS	POS	POS	NEG	NEG	NEG	NEG	AMB	NEG	NEG	POS	POS	NEG	NEG	NEG	POS	NEG	NEG	NEG	POS	NEG	POS	NEG	POS	POS	NEG	POS	NEG	NEG
Iasi-31-355	SSTI	POS	AMB	POS	AMB	POS	NEG	POS	POS	POS	NEG	NEG	NEG	NEG	POS	NEG	NEG	POS	POS	NEG	NEG	NEG	POS	NEG	NEG	NEG	POS	NEG	POS	NEG	POS	POS	NEG	POS	NEG	NEG
Iasi-38-377	SSTI	POS	AMB	POS	AMB	POS	NEG	POS	POS	POS	NEG	NEG	NEG	NEG	POS	NEG	NEG	POS	POS	NEG	NEG	NEG	POS	NEG	NEG	NEG	POS	NEG	POS	NEG	POS	POS	NEG	POS	NEG	NEG
Iasi-41-388	SSTI	AMB	POS	POS	AMB	POS	NEG	POS	POS	POS	NEG	NEG	NEG	NEG	POS	NEG	NEG	POS	POS	NEG	NEG	NEG	POS	NEG	NEG	NEG	POS	NEG	POS	NEG	POS	POS	NEG	POS	NEG	NEG
Iasi-46-417	SSTI	POS	AMB	POS	AMB	POS	NEG	POS	POS	POS	NEG	NEG	NEG	NEG	POS	NEG	NEG	POS	POS	NEG	NEG	NEG	POS	NEG	NEG	NEG	POS	NEG	POS	NEG	POS	POS	NEG	POS	NEG	NEG
Iasi-02-S9	Carrier	POS	AMB	POS	AMB	POS	AMB	POS	POS	POS	AMB	NEG	NEG	NEG	POS	NEG	NEG	POS	POS	NEG	AMB	NEG	POS	POS	NEG	NEG	POS	NEG	POS	NEG	POS	POS	AMB	POS	POS	NEG
Iasi-17-S33	Carrier	POS	AMB	POS	AMB	POS	NEG	POS	POS	POS	NEG	NEG	NEG	NEG	POS	NEG	NEG	POS	POS	NEG	NEG	NEG	POS	AMB	NEG	NEG	POS	NEG	POS	NEG	POS	POS	NEG	POS	POS	NEG
Iasi-30-P17	Carrier	POS	AMB	POS	AMB	POS	AMB	POS	POS	POS	NEG	NEG	NEG	NEG	POS	NEG	NEG	POS	POS	NEG	NEG	NEG	POS	AMB	NEG	NEG	POS	NEG	POS	NEG	POS	POS	NEG	POS	AMB	NEG
Iasi-39-P28	Carrier	POS	AMB	POS	AMB	POS	AMB	POS	POS	POS	NEG	NEG	NEG	NEG	POS	NEG	NEG	POS	POS	NEG	NEG	NEG	POS	AMB	NEG	NEG	POS	NEG	POS	NEG	POS	POS	AMB	POS	AMB	NEG
Iasi-04-26	BSI	POS	AMB	POS	AMB	POS	NEG	POS	POS	POS	NEG	NEG	NEG	NEG	POS	NEG	NEG	POS	POS	NEG	NEG	NEG	POS	AMB	NEG	NEG	POS	NEG	POS	NEG	POS	POS	AMB	POS	AMB	NEG
Iasi-09-72	BSI	POS	AMB	POS	AMB	POS	NEG	POS	POS	POS	NEG	NEG	NEG	NEG	POS	NEG	NEG	POS	POS	NEG	NEG	NEG	POS	NEG	NEG	NEG	POS	NEG	POS	NEG	POS	POS	NEG	POS	NEG	NEG
Iasi-11-131	BSI	POS	AMB	POS	AMB	POS	NEG	POS	POS	POS	NEG	NEG	NEG	NEG	POS	NEG	NEG	POS	POS	NEG	NEG	NEG	POS	NEG	NEG	NEG	POS	NEG	POS	NEG	POS	POS	NEG	POS	AMB	NEG
Iasi-14-143	BSI	POS	AMB	POS	AMB	POS	NEG	POS	POS	POS	NEG	NEG	NEG	NEG	POS	NEG	NEG	POS	POS	NEG	NEG	NEG	POS	NEG	NEG	NEG	POS	NEG	POS	NEG	POS	POS	NEG	POS	NEG	NEG
Iasi-18-192	BSI	POS	AMB	POS	AMB	POS	NEG	POS	POS	POS	NEG	NEG	NEG	NEG	POS	NEG	NEG	POS	POS	NEG	NEG	NEG	POS	NEG	NEG	NEG	POS	NEG	POS	NEG	POS	POS	NEG	POS	NEG	NEG
Iasi-23-253	BSI	POS	AMB	POS	AMB	POS	AMB	POS	POS	POS	NEG	NEG	NEG	NEG	POS	NEG	NEG	POS	POS	NEG	AMB	NEG	POS	AMB	NEG	NEG	POS	NEG	POS	NEG	POS	POS	NEG	POS	POS	NEG
Iasi-35-322	BSI	POS	AMB	POS	AMB	POS	NEG	POS	POS	POS	NEG	NEG	NEG	NEG	POS	NEG	NEG	POS	POS	NEG	NEG	NEG	POS	NEG	NEG	NEG	POS	NEG	POS	NEG	POS	POS	NEG	POS	NEG	NEG
Iasi-40-380	BSI	POS	AMB	POS	AMB	POS	NEG	POS	POS	POS	NEG	NEG	NEG	NEG	POS	NEG	NEG	POS	POS	NEG	NEG	NEG	POS	AMB	NEG	NEG	POS	NEG	POS	NEG	POS	POS	NEG	POS	NEG	NEG
Iasi-46-406	BSI	POS	AMB	POS	AMB	POS	NEG	POS	POS	POS	NEG	NEG	NEG	NEG	POS	NEG	NEG	POS	POS	NEG	NEG	NEG	POS	NEG	NEG	NEG	POS	NEG	POS	NEG	POS	POS	NEG	POS	NEG	NEG
Iasi-02-6	SSTI	POS	AMB	POS	AMB	POS	NEG	POS	POS	POS	NEG	NEG	NEG	NEG	POS	NEG	NEG	POS	POS	NEG	NEG	NEG	POS	NEG	NEG	NEG	POS	NEG	POS	NEG	POS	POS	NEG	POS	NEG	NEG
Iasi-09-97	SSTI	POS	AMB	POS	AMB	POS	NEG	POS	POS	POS	NEG	NEG	NEG	NEG	POS	NEG	NEG	POS	POS	NEG	NEG	NEG	POS	NEG	NEG	NEG	POS	NEG	POS	NEG	POS	POS	NEG	POS	NEG	NEG
Iasi-19-182	SSTI	POS	AMB	POS	AMB	POS	NEG	POS	POS	POS	NEG	NEG	NEG	NEG	POS	NEG	NEG	POS	POS	NEG	NEG	NEG	POS	NEG	NEG	NEG	POS	NEG	POS	NEG	POS	POS	NEG	POS	NEG	NEG
Iasi-31-314	SSTI	POS	AMB	POS	AMB	POS	NEG	POS	POS	POS	NEG	NEG	NEG	NEG	POS	NEG	NEG	POS	POS	NEG	NEG	NEG	POS	NEG	NEG	NEG	POS	NEG	POS	NEG	POS	POS	NEG	POS	NEG	NEG
Iasi-33-367	SSTI	POS	AMB	POS	AMB	POS	NEG	POS	POS	POS	NEG	NEG	NEG	NEG	POS	NEG	NEG	POS	POS	NEG	NEG	NEG	POS	NEG	NEG	NEG	POS	NEG	POS	NEG	POS	POS	NEG	POS	NEG	NEG
Iasi-39-378	SSTI	POS	AMB	POS	AMB	POS	NEG	POS	POS	POS	NEG	NEG	NEG	NEG	POS	NEG	NEG	POS	POS	NEG	NEG	NEG	POS	NEG	NEG	NEG	POS	NEG	POS	NEG	POS	POS	NEG	POS	NEG	NEG
Iasi-42-394	SSTI	POS	AMB	POS	AMB	POS	NEG	POS	POS	POS	NEG	NEG	NEG	NEG	POS	NEG	NEG	POS	POS	NEG	NEG	NEG	POS	NEG	NEG	NEG	POS	NEG	POS	NEG	POS	POS	NEG	POS	NEG	NEG
Iasi-44-409	SSTI	POS	AMB	POS	AMB	POS	NEG	POS	POS	POS	NEG	NEG	NEG	NEG	POS	NEG	NEG	POS	POS	NEG	NEG	NEG	POS	NEG	NEG	NEG	POS	NEG	POS	NEG	POS	POS	NEG	POS	NEG	NEG
Iasi-45-405	SSTI	POS	AMB	POS	AMB	POS	NEG	POS	POS	POS	NEG	NEG	NEG	NEG	POS	NEG	NEG	POS	POS	NEG	NEG	NEG	POS	NEG	NEG	NEG	POS	NEG	POS	NEG	POS	POS	NEG	POS	NEG	NEG
Iasi-50-425	SSTI	POS	AMB	POS	AMB	POS	NEG	POS	POS	POS	NEG	NEG	NEG	NEG	POS	NEG	NEG	POS	POS	NEG	NEG	NEG	POS	NEG	NEG	NEG	POS	NEG	POS	NEG	POS	POS	NEG	POS	NEG	NEG
Iasi-50-428	SSTI	POS	AMB	POS	AMB	POS	NEG	POS	POS	POS	NEG	NEG	NEG	NEG	POS	NEG	NEG	POS	POS	NEG	NEG	NEG	POS	NEG	NEG	NEG	POS	NEG	POS	NEG	POS	POS	NEG	POS	NEG	NEG
Iasi-51-37	SSTI	AMB	POS	POS	AMB	POS	NEG	POS	POS	POS	NEG	NEG	NEG	NEG	POS	NEG	NEG	POS	POS	NEG	NEG	NEG	POS	AMB	NEG	NEG	POS	NEG	POS	NEG	POS	POS	NEG	POS	AMB	NEG
Iasi-01-S8	Carrier	POS	AMB	POS	AMB	POS	AMB	POS	POS	POS	NEG	NEG	NEG	NEG	POS	NEG	NEG	POS	POS	NEG	AMB	NEG	POS	AMB	NEG	NEG	POS	NEG	POS	NEG	POS	POS	NEG	POS	POS	NEG
Iasi-08-S17	Carrier	POS	AMB	POS	AMB	POS	NEG	POS	POS	POS	NEG	NEG	NEG	NEG	POS	NEG	NEG	POS	POS	NEG	NEG	NEG	POS	AMB	NEG	NEG	POS	NEG	POS	NEG	POS	POS	NEG	POS	AMB	NEG
Iasi-10-S23	Carrier	POS	AMB	POS	AMB	POS	AMB	POS	POS	POS	NEG	NEG	NEG	NEG	POS	NEG	NEG	POS	POS	NEG	NEG	NEG	POS	POS	NEG	NEG	POS	NEG	POS	NEG	POS	POS	NEG	POS	AMB	NEG
Iasi-13-S27	Carrier	POS	AMB	POS	AMB	POS	NEG	POS	POS	POS	NEG	NEG	NEG	NEG	POS	NEG	NEG	POS	POS	NEG	AMB	NEG	POS	AMB	NEG	NEG	POS	NEG	POS	NEG	POS	POS	AMB	POS	AMB	NEG
Iasi-14-S28	Carrier	POS	AMB	POS	AMB	POS	AMB	POS	POS	POS	NEG	NEG	NEG	NEG	POS	NEG	NEG	POS	POS	NEG	NEG	NEG	POS	AMB	NEG	NEG	POS	NEG	POS	NEG	POS	POS	NEG	POS	AMB	NEG
Iasi-15-S29	Carrier	POS	AMB	POS	AMB	POS	AMB	POS	POS	POS	NEG	NEG	NEG	NEG	POS	NEG	NEG	POS	POS	NEG	AMB	NEG	POS	POS	NEG	NEG	POS	NEG	POS	NEG	POS	POS	NEG	POS	POS	NEG
Iasi-25-P10	Carrier	POS	AMB	POS	AMB	POS	AMB	POS	POS	POS	AMB	NEG	NEG	NEG	POS	NEG	NEG	POS	POS	NEG	AMB	NEG	POS	AMB	NEG	NEG	POS	NEG	POS	NEG	POS	POS	NEG	POS	POS	NEG
Iasi-26-P11	Carrier	AMB	POS	POS	AMB	POS	NEG	POS	POS	POS	NEG	NEG	NEG	NEG	POS	NEG	NEG	POS	POS	NEG	AMB	NEG	POS	AMB	NEG	NEG	POS	NEG	POS	NEG	POS	POS	NEG	POS	POS	NEG
Iasi-32-P19	Carrier	POS	AMB	POS	AMB	POS	NEG	POS	POS	POS	NEG	NEG	NEG	NEG	POS	NEG	NEG	POS	POS	NEG	NEG	NEG	POS	AMB	NEG	NEG	POS	NEG	POS	NEG	POS	POS	NEG	POS	AMB	NEG
Iasi-33-P22	Carrier	POS	AMB	POS	AMB	POS	AMB	POS	POS	POS	NEG	NEG	NEG	NEG	POS	NEG	NEG	POS	POS	NEG	NEG	NEG	POS	AMB	NEG	NEG	POS	NEG	POS	NEG	POS	POS	AMB	POS	POS	NEG
Iasi-41-P44	Carrier	POS	AMB	POS	AMB	POS	NEG	POS	POS	POS	NEG	NEG	NEG	NEG	POS	NEG	NEG	POS	POS	NEG	NEG	NEG	POS	AMB	NEG	NEG	POS	NEG	POS	NEG	POS	POS	AMB	POS	AMB	NEG
Iasi-45-G515	Carrier	AMB	POS	POS	AMB	POS	AMB	POS	POS	POS	NEG	NEG	NEG	NEG	POS	NEG	NEG	POS	POS	NEG	NEG	NEG	POS	AMB	NEG	NEG	POS	NEG	POS	NEG	POS	POS	NEG	POS	AMB	NEG
Iasi-02-39	misc. MRSA	POS	AMB	POS	AMB	POS	NEG	POS	POS	POS	NEG	NEG	NEG	NEG	POS	NEG	NEG	POS	POS	NEG	NEG	NEG	POS	NEG	NEG	NEG	POS	NEG	POS	NEG	POS	POS	NEG	POS	NEG	NEG
Iasi-24-263	misc. MRSA	POS	AMB	POS	AMB	POS	NEG	POS	POS	POS	NEG	NEG	NEG	NEG	POS	NEG	NEG	POS	POS	NEG	NEG	NEG	POS	NEG	NEG	NEG	POS	NEG	POS	NEG	POS	POS	NEG	POS	AMB	NEG
Iasi-26-285	misc. MRSA	POS	AMB	POS	AMB	POS	NEG	POS	POS	POS	NEG	NEG	NEG	NEG	POS	NEG	NEG	POS	POS	NEG	NEG	NEG	POS	NEG	NEG	NEG	POS	NEG	POS	NEG	POS	POS	NEG	POS	AMB	NEG
Iasi-30-306	misc. MRSA	POS	AMB	POS	AMB	POS	AMB	POS	POS	POS	NEG	NEG	NEG	NEG	POS	NEG	NEG	POS	POS	NEG	AMB	NEG	POS	AMB	NEG	NEG	POS	NEG	POS	NEG	POS	POS	NEG	POS	POS	NEG
Iasi-41-390	misc. MRSA	POS	AMB	POS	AMB	POS	NEG	POS	POS	POS	NEG	NEG	NEG	NEG	POS	NEG	NEG	POS	POS	NEG	NEG	NEG	POS	AMB	NEG	NEG	POS	NEG	POS	NEG	POS	POS	NEG	POS	AMB	NEG
Iasi-47-407	misc. MRSA	POS	AMB	POS	AMB	POS	NEG	POS	POS	POS	NEG	NEG	NEG	NEG	POS	NEG	NEG	POS	POS	NEG	NEG	NEG	POS	AMB	NEG	NEG	POS	NEG	POS	NEG	POS	POS	NEG	POS	POS	NEG
Iasi-55-408CP	misc. MRSA	AMB	POS	POS	AMB	POS	NEG	POS	POS	POS	NEG	NEG	NEG	NEG	POS	NEG	NEG	POS	POS	NEG	NEG	NEG	POS	AMB	NEG	NEG	POS	NEG	POS	NEG	POS	POS	NEG	POS	AMB	NEG
Iasi-15-151	BSI	POS	AMB	POS	AMB	POS	AMB	POS	POS	POS	NEG	NEG	NEG	POS	NEG	NEG	NEG	POS	NEG	POS	NEG	NEG	POS	AMB	NEG	NEG	POS	NEG	POS	NEG	POS	POS	NEG	NEG	POS	POS
Iasi-46-386	BSI	POS	AMB	POS	AMB	POS	AMB	POS	POS	POS	NEG	NEG	NEG	POS	POS	NEG	NEG	POS	NEG	POS	AMB	NEG	POS	AMB	NEG	NEG	POS	NEG	POS	NEG	POS	POS	NEG	NEG	POS	POS
Iasi-21-195	SSTI	POS	AMB	AMB	POS	POS	NEG	POS	POS	POS	NEG	NEG	NEG	POS	NEG	NEG	NEG	POS	NEG	POS	NEG	NEG	POS	NEG	NEG	NEG	POS	NEG	POS	NEG	POS	POS	NEG	NEG	NEG	POS
Iasi-37-375	SSTI	POS	AMB	AMB	POS	POS	NEG	POS	POS	POS	NEG	NEG	NEG	POS	AMB	NEG	NEG	POS	NEG	POS	NEG	NEG	POS	NEG	NEG	NEG	POS	NEG	POS	NEG	POS	POS	NEG	NEG	NEG	POS
Iasi-45-415	SSTI	POS	AMB	AMB	POS	POS	NEG	POS	POS	POS	NEG	NEG	NEG	POS	AMB	NEG	NEG	POS	NEG	POS	NEG	NEG	POS	NEG	NEG	NEG	POS	NEG	POS	NEG	POS	POS	NEG	NEG	NEG	POS
Iasi-09-S19	Carrier	POS	AMB	POS	AMB	POS	AMB	POS	POS	POS	AMB	NEG	NEG	POS	POS	NEG	NEG	POS	NEG	POS	NEG	NEG	POS	AMB	NEG	NEG	POS	NEG	POS	NEG	POS	POS	NEG	NEG	POS	POS
Iasi-53-434	BSI	POS	AMB	POS	AMB	POS	AMB	POS	POS	POS	NEG	NEG	NEG	POS	POS	NEG	NEG	POS	NEG	POS	AMB	NEG	POS	AMB	NEG	NEG	POS	NEG	POS	NEG	POS	POS	NEG	NEG	POS	POS
Iasi-03-40	misc. MRSA	POS	AMB	POS	AMB	POS	AMB	POS	POS	POS	NEG	NEG	NEG	POS	NEG	NEG	NEG	POS	NEG	POS	NEG	NEG	POS	AMB	NEG	NEG	POS	NEG	POS	NEG	POS	POS	NEG	NEG	POS	POS
Iasi-04-43	misc. MRSA	POS	AMB	AMB	POS	POS	NEG	POS	POS	POS	NEG	NEG	NEG	POS	NEG	NEG	NEG	POS	NEG	POS	NEG	NEG	POS	NEG	NEG	NEG	POS	NEG	POS	NEG	POS	POS	NEG	NEG	NEG	POS
Iasi-01-20	misc. MRSA	POS	AMB	POS	AMB	POS	AMB	POS	POS	POS	NEG	NEG	NEG	POS	#ZAHL!	NEG	NEG	POS	NEG	POS	NEG	NEG	POS	AMB	NEG	NEG	POS	NEG	POS	NEG	POS	POS	NEG	NEG	AMB	POS
Iasi-35-228	BSI	POS	AMB	POS	AMB	POS	AMB	POS	POS	POS	NEG	NEG	NEG	NEG	NEG	NEG	NEG	POS	POS	NEG	POS	NEG	POS	AMB	NEG	NEG	POS	NEG	NEG	NEG	POS	POS	NEG	POS	AMB	NEG
Iasi-38-293	BSI	POS	AMB	POS	AMB	POS	AMB	POS	POS	POS	AMB	NEG	NEG	NEG	POS	NEG	NEG	NEG	NEG	NEG	NEG	NEG	POS	POS	POS	NEG	POS	NEG	NEG	NEG	POS	POS	NEG	NEG	POS	POS
Iasi-31-P18	Carrier	AMB	POS	POS	AMB	POS	AMB	POS	POS	POS	NEG	NEG	NEG	NEG	POS	NEG	NEG	NEG	NEG	NEG	NEG	NEG	POS	POS	POS	NEG	POS	NEG	NEG	NEG	POS	POS	NEG	NEG	POS	POS
Iasi-40-P42	Carrier	POS	AMB	POS	AMB	POS	AMB	POS	POS	POS	AMB	NEG	NEG	NEG	POS	NEG	NEG	NEG	NEG	NEG	NEG	NEG	POS	POS	POS	NEG	POS	NEG	AMB	NEG	POS	POS	AMB	NEG	POS	POS
Iasi-40-303	BSI	POS	AMB	POS	AMB	POS	AMB	POS	POS	POS	NEG	NEG	NEG	POS	AMB	NEG	NEG	POS	POS	NEG	NEG	NEG	POS	NEG	NEG	NEG	POS	POS	POS	POS	NEG	NEG	NEG	POS	AMB	NEG
Iasi-48-422	SSTI	AMB	POS	POS	AMB	POS	NEG	POS	POS	POS	NEG	NEG	NEG	POS	AMB	NEG	NEG	POS	POS	NEG	NEG	NEG	POS	NEG	NEG	NEG	POS	POS	POS	POS	NEG	NEG	NEG	POS	NEG	NEG
Iasi-54-435	BSI	POS	AMB	AMB	POS	POS	AMB	POS	POS	POS	AMB	NEG	NEG	POS	POS	NEG	AMB	POS	POS	NEG	AMB	NEG	POS	AMB	NEG	NEG	POS	POS	POS	POS	NEG	NEG	NEG	POS	POS	NEG
Iasi-16-161	SSTI	POS	AMB	POS	AMB	POS	NEG	POS	POS	POS	NEG	NEG	NEG	POS	AMB	NEG	NEG	POS	POS	NEG	NEG	NEG	POS	NEG	NEG	NEG	POS	POS	POS	POS	NEG	NEG	NEG	POS	NEG	NEG
Iasi-27-221	SSTI	POS	AMB	POS	AMB	POS	NEG	POS	POS	POS	NEG	NEG	NEG	POS	AMB	NEG	NEG	POS	POS	NEG	NEG	NEG	POS	NEG	NEG	NEG	POS	POS	POS	POS	NEG	NEG	NEG	POS	NEG	NEG
Iasi-09-85	BSI	POS	AMB	POS	AMB	POS	NEG	POS	POS	POS	NEG	NEG	NEG	POS	NEG	NEG	NEG	POS	POS	NEG	NEG	NEG	POS	NEG	NEG	NEG	POS	POS	POS	POS	NEG	NEG	NEG	POS	NEG	NEG
Iasi-17-189	BSI	POS	AMB	POS	AMB	POS	NEG	POS	POS	POS	NEG	NEG	NEG	POS	NEG	NEG	NEG	POS	POS	NEG	NEG	NEG	POS	NEG	NEG	NEG	POS	POS	POS	POS	NEG	NEG	NEG	POS	NEG	NEG
Iasi-20-200	BSI	POS	AMB	POS	AMB	POS	NEG	POS	POS	POS	NEG	NEG	NEG	POS	NEG	NEG	NEG	POS	POS	NEG	NEG	NEG	POS	NEG	NEG	NEG	POS	POS	POS	POS	NEG	NEG	NEG	POS	NEG	NEG
Iasi-34-321	BSI	POS	AMB	POS	AMB	POS	NEG	POS	POS	POS	NEG	NEG	NEG	POS	NEG	NEG	NEG	POS	POS	NEG	NEG	NEG	POS	NEG	NEG	NEG	POS	POS	POS	POS	NEG	NEG	NEG	POS	NEG	NEG
Iasi-04-57	SSTI	POS	AMB	POS	AMB	POS	NEG	POS	POS	POS	NEG	NEG	NEG	POS	NEG	NEG	NEG	POS	POS	NEG	NEG	NEG	POS	NEG	NEG	NEG	POS	POS	POS	POS	NEG	NEG	NEG	POS	NEG	NEG
Iasi-05-59	SSTI	POS	AMB	POS	AMB	POS	NEG	POS	POS	POS	NEG	NEG	NEG	POS	AMB	NEG	NEG	POS	POS	NEG	NEG	NEG	POS	NEG	NEG	NEG	POS	POS	POS	POS	NEG	NEG	NEG	POS	NEG	NEG
Iasi-06-64	SSTI	POS	AMB	POS	AMB	POS	NEG	POS	POS	POS	NEG	NEG	NEG	POS	NEG	NEG	NEG	POS	POS	NEG	NEG	NEG	POS	NEG	NEG	NEG	POS	POS	POS	POS	NEG	NEG	NEG	POS	NEG	NEG
Iasi-10-108	SSTI	POS	AMB	POS	AMB	POS	NEG	POS	POS	POS	NEG	NEG	NEG	POS	NEG	NEG	NEG	POS	POS	NEG	NEG	NEG	POS	NEG	NEG	NEG	POS	POS	POS	POS	NEG	NEG	NEG	POS	NEG	NEG
Iasi-12-117	SSTI	POS	AMB	POS	AMB	POS	NEG	POS	POS	POS	NEG	NEG	NEG	POS	NEG	NEG	NEG	POS	POS	NEG	NEG	NEG	POS	NEG	NEG	NEG	POS	POS	POS	POS	NEG	NEG	NEG	POS	NEG	NEG
Iasi-12-138	SSTI	POS	AMB	POS	AMB	POS	NEG	POS	POS	POS	NEG	NEG	NEG	POS	NEG	NEG	NEG	POS	POS	NEG	NEG	NEG	POS	NEG	NEG	NEG	POS	POS	POS	POS	NEG	NEG	NEG	POS	NEG	NEG
Iasi-13-140	SSTI	POS	AMB	POS	AMB	POS	NEG	POS	POS	POS	NEG	NEG	NEG	POS	NEG	NEG	NEG	POS	POS	NEG	NEG	NEG	POS	NEG	NEG	NEG	POS	POS	POS	POS	NEG	NEG	NEG	POS	NEG	NEG
Iasi-15-152	SSTI	POS	AMB	POS	AMB	POS	NEG	POS	POS	POS	NEG	NEG	NEG	POS	NEG	NEG	NEG	POS	POS	NEG	NEG	NEG	POS	NEG	NEG	NEG	POS	POS	POS	POS	NEG	NEG	NEG	POS	NEG	NEG
Iasi-17-168	SSTI	POS	AMB	POS	AMB	POS	NEG	POS	POS	POS	NEG	NEG	NEG	POS	AMB	NEG	NEG	POS	POS	NEG	NEG	NEG	POS	NEG	NEG	NEG	POS	POS	POS	POS	NEG	NEG	NEG	POS	NEG	NEG
Iasi-20-193	SSTI	POS	AMB	POS	AMB	POS	NEG	POS	POS	POS	NEG	NEG	NEG	POS	NEG	NEG	NEG	POS	POS	NEG	NEG	NEG	POS	NEG	NEG	NEG	POS	POS	POS	POS	NEG	NEG	NEG	POS	NEG	NEG
Iasi-26-218	SSTI	POS	AMB	POS	AMB	POS	NEG	POS	POS	POS	NEG	NEG	NEG	POS	AMB	NEG	NEG	POS	POS	NEG	NEG	NEG	POS	NEG	NEG	NEG	POS	POS	POS	POS	NEG	NEG	NEG	POS	NEG	NEG
Iasi-28-335	SSTI	POS	AMB	POS	AMB	POS	NEG	POS	POS	POS	NEG	NEG	NEG	POS	AMB	NEG	NEG	POS	POS	NEG	NEG	NEG	POS	NEG	NEG	NEG	POS	POS	POS	POS	NEG	NEG	NEG	POS	NEG	NEG
Iasi-29-299b	SSTI	POS	AMB	POS	AMB	POS	AMB	POS	POS	POS	NEG	NEG	NEG	POS	NEG	NEG	NEG	POS	POS	NEG	NEG	NEG	POS	NEG	NEG	NEG	POS	POS	POS	POS	NEG	NEG	NEG	POS	AMB	NEG
Iasi-32-364	SSTI	POS	AMB	POS	AMB	POS	NEG	POS	POS	POS	NEG	NEG	NEG	POS	AMB	NEG	NEG	POS	POS	NEG	NEG	NEG	POS	NEG	NEG	NEG	POS	POS	POS	POS	NEG	NEG	NEG	POS	NEG	NEG
Iasi-369	SSTI	POS	AMB	POS	AMB	POS	AMB	POS	POS	POS	NEG	NEG	NEG	POS	AMB	NEG	NEG	POS	POS	NEG	NEG	NEG	POS	NEG	NEG	NEG	POS	POS	POS	POS	NEG	NEG	NEG	POS	NEG	NEG
Iasi-03-S11	Carrier	POS	AMB	POS	AMB	POS	AMB	POS	POS	POS	NEG	NEG	NEG	POS	POS	NEG	NEG	POS	POS	NEG	NEG	NEG	POS	AMB	NEG	NEG	POS	POS	POS	POS	NEG	NEG	NEG	POS	AMB	NEG
Iasi-19-S35	Carrier	AMB	POS	AMB	POS	POS	AMB	POS	POS	POS	NEG	NEG	NEG	POS	POS	NEG	NEG	POS	POS	NEG	NEG	NEG	POS	AMB	NEG	NEG	POS	POS	POS	POS	NEG	NEG	AMB	POS	POS	NEG
Iasi-20-P1	Carrier	POS	AMB	POS	AMB	POS	AMB	POS	POS	POS	NEG	NEG	NEG	POS	POS	NEG	NEG	POS	POS	NEG	NEG	NEG	POS	AMB	NEG	NEG	POS	POS	POS	POS	NEG	NEG	NEG	POS	AMB	NEG
Iasi-35-P24	Carrier	POS	AMB	POS	AMB	POS	AMB	POS	POS	POS	NEG	NEG	NEG	POS	AMB	NEG	NEG	POS	POS	NEG	NEG	NEG	POS	NEG	NEG	NEG	POS	POS	POS	POS	NEG	NEG	AMB	POS	NEG	NEG
Iasi-36-P25	Carrier	POS	AMB	POS	AMB	POS	AMB	POS	POS	POS	NEG	NEG	NEG	POS	POS	NEG	NEG	POS	POS	NEG	NEG	NEG	POS	AMB	NEG	NEG	POS	POS	POS	POS	NEG	NEG	AMB	POS	AMB	NEG
Iasi-14-150	SSTI	POS	AMB	POS	AMB	POS	NEG	POS	POS	POS	NEG	NEG	NEG	POS	NEG	NEG	NEG	POS	POS	NEG	NEG	NEG	POS	NEG	NEG	NEG	POS	POS	POS	POS	NEG	NEG	NEG	POS	NEG	NEG
Iasi-07-54	BSI	NEG	POS	POS	AMB	POS	AMB	POS	POS	POS	NEG	NEG	NEG	POS	#ZAHL!	NEG	NEG	POS	POS	NEG	NEG	NEG	POS	NEG	NEG	NEG	POS	POS	POS	POS	NEG	NEG	NEG	POS	AMB	NEG
Iasi-16-188	BSI	NEG	POS	POS	AMB	POS	NEG	POS	POS	POS	NEG	NEG	NEG	POS	NEG	NEG	NEG	POS	POS	NEG	NEG	NEG	POS	NEG	NEG	NEG	POS	POS	POS	POS	NEG	NEG	NEG	POS	AMB	NEG
Iasi-32-318	BSI	NEG	POS	POS	AMB	POS	NEG	POS	POS	POS	NEG	NEG	NEG	POS	NEG	NEG	NEG	POS	POS	NEG	NEG	NEG	POS	NEG	NEG	NEG	POS	POS	POS	POS	NEG	NEG	NEG	POS	NEG	NEG
Iasi-42-397	BSI	NEG	POS	POS	AMB	POS	NEG	POS	POS	POS	NEG	NEG	NEG	POS	NEG	NEG	NEG	POS	POS	NEG	NEG	NEG	POS	NEG	NEG	NEG	POS	POS	POS	POS	NEG	NEG	NEG	POS	NEG	NEG
Iasi-48-408	BSI	NEG	POS	POS	AMB	POS	NEG	POS	POS	POS	NEG	NEG	NEG	POS	NEG	NEG	NEG	POS	POS	NEG	NEG	NEG	POS	NEG	NEG	NEG	POS	POS	POS	POS	NEG	NEG	NEG	POS	NEG	NEG
Iasi-49-430	BSI	NEG	POS	POS	AMB	POS	AMB	POS	POS	POS	NEG	NEG	NEG	POS	AMB	NEG	NEG	POS	POS	NEG	NEG	NEG	POS	NEG	NEG	NEG	POS	POS	POS	POS	NEG	NEG	NEG	POS	AMB	NEG
Iasi-52-52	BSI	NEG	POS	POS	AMB	POS	AMB	POS	POS	POS	NEG	NEG	NEG	POS	AMB	NEG	NEG	POS	POS	NEG	NEG	NEG	POS	NEG	NEG	NEG	POS	POS	POS	POS	NEG	NEG	NEG	POS	NEG	NEG
Iasi-01-2	SSTI	NEG	POS	POS	AMB	POS	AMB	POS	POS	POS	NEG	NEG	NEG	POS	AMB	NEG	NEG	POS	POS	NEG	NEG	NEG	POS	NEG	NEG	NEG	POS	POS	POS	POS	NEG	NEG	NEG	POS	NEG	NEG
Iasi-10-101	SSTI	NEG	POS	POS	AMB	POS	NEG	POS	POS	POS	NEG	NEG	NEG	POS	NEG	NEG	NEG	POS	POS	NEG	NEG	NEG	POS	NEG	NEG	NEG	POS	POS	POS	POS	NEG	NEG	NEG	POS	NEG	NEG
Iasi-11-106	SSTI	NEG	POS	POS	AMB	POS	NEG	POS	POS	POS	NEG	NEG	NEG	POS	NEG	NEG	NEG	POS	POS	NEG	NEG	NEG	POS	NEG	NEG	NEG	POS	POS	POS	POS	NEG	NEG	NEG	POS	NEG	NEG
Iasi-49-414	SSTI	NEG	POS	POS	AMB	POS	AMB	POS	POS	POS	NEG	NEG	NEG	POS	POS	NEG	NEG	POS	POS	NEG	NEG	NEG	POS	NEG	NEG	NEG	POS	POS	POS	POS	NEG	NEG	NEG	POS	AMB	NEG

Isolate	Group	IMMUNOD.AG.B		DEFENSIN RESIST.		TRANSFERRIN BINDING PROT			PUTATIVE TRANSPORTER				TYPE I RESTRICTION-MODIFICATION SYSTEM, SINGLE SEQUENCE SPECIFICITY PROTEIN					TYPE I RESTRICTION-MODIFICATION SYSTEM, SINGLE SEQUENCE SPECIFICITY PROTEIN								HYALURONATE LYASE								MISCELLANEOUS GENES		
		immunodominant antigen B		defensin resistance protein		transferrin-binding protein			hypothetical protein, similar to integral membrane protein LmrP				type I site-specific deoxyribo-nuclease subunit, 1st locus	type I site-specific deoxyribonuclease subunit, 2nd locus				type I site-specific deoxyribonuclease subunit, 3rd locus					type I site-specific deoxyribonuclease subunit, unknown locus			hyaluronate lyase, first / second locus			hyaluronate lyase, second locus					hypothetical protein, located next to serine protease operon	Unspecific efflux/trans-porter	hypothetical protein
		isaB	isaB (MRSA252)	mprF (COL+MW2)	mprF (Mu50+MRSA252)	isdA (consensus)	isdA (MRSA252)	isdA (other than MRSA252)	lmrP (other than RF122)_probe1	lmrP (other than RF122)_probe2	lmrP (RF122)_probe1	lmrP (RF122)_probe2	hsdS1 (RF122)	hsdS2 (Mu50+N315+COL+USA300+NCTC8325)	hsdS2 (MW2+MSSA476)	hsdS2 (RF122)	hsdS2 (MRSA252)	hsdS3 (all other than RF122+MRSA252)	hsdS3 (COL+USA300+NCTC8325+MW2+MSSA476+RF122)	hsdS3 (Mu50+N315)	hsdS3 (CC51+MRSA252)	hsdS3 (MRSA252)	hsdSx (CC25)	hsdSx (CC15)	hsdSx (etd)	hysA1 (MRSA252)	hysA1 (MRSA252+RF122) and/or hysA2 (consensus)	hysA1 (MRSA252+RF122) and/or hysA2 (COL+USA300)	hysA2 (all other than MRSA252)	hysA2 (COL+USA300+NCTC8325)	hysA2 (all other than COL+USA300+NCTC8325)	hysA2 (all other than COL+USA300+NCTC8325)	hysA2 (MRSA252)	Q2FXC0	Q2YUB3	Q7A4X2
Iasi-05-46	misc. MRSA	NEG	POS	POS	AMB	POS	NEG	POS	POS	POS	NEG	NEG	NEG	POS	NEG	NEG	NEG	POS	POS	NEG	NEG	NEG	POS	NEG	NEG	NEG	POS	POS	POS	POS	NEG	NEG	NEG	POS	NEG	NEG
Iasi-06-47	misc. MRSA	NEG	POS	POS	AMB	POS	AMB	POS	POS	POS	NEG	NEG	NEG	POS	NEG	NEG	NEG	POS	POS	NEG	NEG	NEG	POS	NEG	NEG	NEG	POS	POS	POS	POS	NEG	NEG	NEG	POS	AMB	NEG
Iasi-08-73	misc. MRSA	NEG	POS	POS	AMB	POS	NEG	POS	POS	POS	NEG	NEG	NEG	POS	NEG	NEG	NEG	POS	POS	NEG	NEG	NEG	POS	NEG	NEG	NEG	POS	POS	POS	POS	NEG	NEG	NEG	POS	AMB	NEG
Iasi-19-194	misc. MRSA	NEG	POS	POS	AMB	POS	NEG	POS	POS	POS	NEG	NEG	NEG	POS	NEG	NEG	NEG	POS	POS	NEG	NEG	NEG	POS	NEG	NEG	NEG	POS	POS	POS	POS	NEG	NEG	NEG	POS	NEG	NEG
Iasi-21-219	misc. MRSA	NEG	POS	POS	AMB	POS	NEG	POS	POS	POS	NEG	NEG	NEG	POS	NEG	NEG	NEG	POS	POS	NEG	NEG	NEG	POS	NEG	NEG	NEG	POS	POS	POS	POS	NEG	NEG	NEG	POS	NEG	NEG
Iasi-25-284	misc. MRSA	NEG	POS	POS	AMB	POS	AMB	POS	POS	POS	NEG	NEG	NEG	POS	AMB	NEG	NEG	POS	POS	NEG	NEG	NEG	POS	NEG	NEG	NEG	POS	POS	POS	POS	NEG	NEG	NEG	POS	NEG	NEG
Iasi-284	misc. MRSA	NEG	POS	POS	AMB	POS	AMB	POS	POS	POS	NEG	NEG	NEG	POS	AMB	NEG	NEG	POS	POS	NEG	NEG	NEG	POS	NEG	NEG	NEG	POS	POS	POS	POS	NEG	NEG	NEG	POS	NEG	NEG
Iasi-36-336	misc. MRSA	NEG	POS	POS	AMB	POS	NEG	POS	POS	POS	NEG	NEG	NEG	POS	NEG	NEG	NEG	POS	POS	NEG	NEG	NEG	POS	NEG	NEG	NEG	POS	POS	POS	POS	NEG	NEG	NEG	POS	NEG	NEG
Iasi-43-402	misc. MRSA	NEG	POS	POS	AMB	POS	AMB	POS	POS	POS	NEG	NEG	NEG	POS	AMB	NEG	NEG	POS	POS	NEG	NEG	NEG	POS	NEG	NEG	NEG	POS	POS	POS	POS	NEG	NEG	NEG	POS	NEG	NEG
Iasi-44-404	misc. MRSA	NEG	POS	POS	AMB	POS	NEG	POS	POS	POS	NEG	NEG	NEG	POS	NEG	NEG	NEG	POS	POS	NEG	NEG	NEG	POS	NEG	NEG	NEG	POS	POS	POS	POS	NEG	NEG	NEG	POS	NEG	NEG
Iasi-54-430	misc. MRSA	AMB	POS	AMB	POS	POS	AMB	POS	POS	POS	NEG	NEG	NEG	POS	POS	NEG	NEG	POS	POS	NEG	NEG	NEG	POS	AMB	NEG	NEG	POS	POS	POS	POS	NEG	NEG	NEG	POS	POS	NEG
Iasi-24-216	SSTI	NEG	POS	POS	AMB	POS	POS	AMB	POS	POS	NEG	NEG	NEG	POS	AMB	NEG	NEG	NEG	NEG	NEG	NEG	NEG	POS	NEG	NEG	POS	POS	POS	NEG	POS	POS	POS	NEG	NEG	NEG	POS
Iasi-05-S13	Carrier	AMB	POS	POS	AMB	POS	POS	AMB	POS	POS	NEG	NEG	NEG	POS	#ZAHL!	NEG	NEG	NEG	NEG	NEG	NEG	NEG	POS	NEG	NEG	POS	POS	POS	AMB	POS	POS	POS	NEG	NEG	POS	POS
Iasi-45-362	BSI	AMB	POS	POS	AMB	POS	POS	NEG	POS	POS	NEG	NEG	NEG	NEG	NEG	NEG	NEG	NEG	NEG	NEG	NEG	NEG	AMB	POS	NEG	NEG	POS	POS	POS	POS	POS	POS	NEG	NEG	NEG	NEG
Iasi-22-199	SSTI	POS	AMB	POS	AMB	POS	POS	NEG	POS	POS	NEG	NEG	NEG	NEG	AMB	NEG	NEG	NEG	NEG	NEG	NEG	NEG	POS	POS	NEG	NEG	POS	POS	POS	POS	POS	POS	NEG	NEG	NEG	NEG
Iasi-12-S26	Carrier	POS	AMB	POS	AMB	POS	POS	AMB	POS	POS	NEG	NEG	NEG	NEG	#ZAHL!	NEG	NEG	POS	NEG	NEG	NEG	NEG	POS	POS	NEG	NEG	POS	POS	POS	POS	POS	POS	NEG	NEG	POS	NEG
Iasi-21-P2	Carrier	POS	AMB	POS	AMB	POS	POS	AMB	POS	POS	NEG	NEG	NEG	NEG	#ZAHL!	NEG	NEG	POS	NEG	NEG	NEG	NEG	POS	POS	NEG	NEG	POS	POS	POS	POS	POS	POS	NEG	NEG	POS	NEG
Iasi-06-60	BSI	NEG	POS	POS	AMB	POS	AMB	POS	POS	POS	NEG	NEG	NEG	POS	NEG	NEG	NEG	POS	NEG	#NEG	NEG	NEG	POS	AMB	NEG	NEG	POS	NEG	AMB	NEG	POS	POS	POS	NEG	#ZAHL!	POS
Iasi-12-79	BSI	NEG	POS	AMB	POS	POS	AMB	POS	POS	POS	NEG	NEG	NEG	POS	NEG	NEG	NEG	AMB	NEG	NEG	NEG	NEG	POS	NEG	NEG	NEG	POS	NEG	NEG	NEG	POS	POS	AMB	NEG	NEG	POS
Iasi-17-90	BSI	NEG	POS	AMB	POS	POS	AMB	POS	POS	POS	NEG	NEG	NEG	POS	NEG	NEG	NEG	POS	NEG	NEG	NEG	NEG	POS	NEG	NEG	NEG	POS	NEG	AMB	NEG	POS	POS	AMB	NEG	NEG	POS
Iasi-21-105	BSI	NEG	POS	POS	AMB	POS	AMB	POS	POS	POS	NEG	NEG	NEG	POS	NEG	NEG	NEG	AMB	NEG	NEG	NEG	NEG	POS	NEG	NEG	NEG	POS	NEG	NEG	NEG	POS	POS	AMB	NEG	NEG	POS
Iasi-22-109	BSI	AMB	POS	POS	AMB	POS	AMB	POS	POS	POS	NEG	NEG	NEG	POS	AMB	NEG	NEG	POS	NEG	NEG	NEG	NEG	POS	AMB	NEG	NEG	POS	NEG	AMB	NEG	POS	POS	POS	NEG	AMB	POS
Iasi-23-110	BSI	NEG	POS	POS	AMB	POS	AMB	POS	POS	POS	NEG	NEG	NEG	POS	NEG	NEG	NEG	AMB	NEG	NEG	NEG	NEG	POS	AMB	NEG	NEG	POS	NEG	AMB	NEG	POS	POS	POS	NEG	AMB	POS
Iasi-24-136	BSI	NEG	POS	POS	AMB	POS	AMB	POS	POS	POS	NEG	NEG	NEG	POS	NEG	NEG	NEG	AMB	NEG	NEG	NEG	NEG	POS	NEG	NEG	NEG	POS	NEG	NEG	NEG	POS	POS	AMB	NEG	AMB	POS
Iasi-34-224	BSI	NEG	POS	POS	AMB	POS	AMB	POS	POS	POS	NEG	NEG	NEG	POS	NEG	NEG	NEG	POS	NEG	NEG	NEG	NEG	POS	NEG	NEG	NEG	POS	NEG	NEG	NEG	POS	POS	NEG	NEG	AMB	POS
Iasi-39-297	BSI	AMB	POS	POS	AMB	POS	AMB	POS	POS	POS	NEG	NEG	NEG	POS	POS	NEG	NEG	POS	NEG	NEG	NEG	NEG	POS	AMB	NEG	NEG	POS	NEG	NEG	NEG	POS	POS	AMB	NEG	POS	POS
Iasi-50-431	BSI	NEG	POS	POS	AMB	POS	AMB	POS	POS	POS	NEG	NEG	NEG	POS	AMB	NEG	NEG	POS	NEG	NEG	NEG	NEG	POS	NEG	NEG	NEG	POS	NEG	NEG	NEG	POS	POS	AMB	NEG	AMB	POS
Iasi-51-432	BSI	AMB	POS	POS	AMB	POS	AMB	POS	POS	POS	NEG	NEG	NEG	POS	POS	NEG	NEG	POS	NEG	NEG	NEG	NEG	POS	AMB	NEG	NEG	POS	NEG	NEG	NEG	POS	POS	AMB	NEG	AMB	POS
Iasi-08-76	SSTI	NEG	POS	AMB	POS	POS	NEG	POS	POS	POS	NEG	NEG	NEG	POS	NEG	NEG	NEG	NEG	NEG	NEG	NEG	NEG	POS	NEG	NEG	NEG	POS	NEG	NEG	NEG	POS	POS	NEG	NEG	NEG	POS
Iasi-30-350	SSTI	NEG	POS	POS	AMB	POS	AMB	POS	POS	POS	NEG	NEG	NEG	POS	AMB	NEG	NEG	AMB	NEG	NEG	NEG	NEG	POS	AMB	NEG	NEG	POS	NEG	NEG	NEG	POS	POS	AMB	NEG	AMB	POS
Iasi-33-319	SSTI	NEG	POS	AMB	POS	POS	NEG	POS	POS	AMB	NEG	NEG	NEG	POS	NEG	NEG	NEG	NEG	NEG	NEG	NEG	NEG	AMB	NEG	NEG	NEG	POS	NEG	NEG	NEG	POS	POS	NEG	NEG	NEG	POS
Iasi-40-383	SSTI	NEG	POS	POS	AMB	POS	AMB	POS	POS	POS	NEG	NEG	NEG	POS	AMB	NEG	NEG	AMB	NEG	NEG	NEG	NEG	POS	NEG	NEG	NEG	POS	NEG	NEG	NEG	POS	POS	AMB	NEG	AMB	POS
Iasi-49-423	SSTI	NEG	POS	AMB	POS	POS	NEG	POS	POS	POS	NEG	NEG	NEG	POS	POS	NEG	NEG	AMB	NEG	NEG	NEG	NEG	POS	NEG	NEG	NEG	POS	NEG	NEG	NEG	POS	POS	NEG	NEG	NEG	POS
Iasi-16-S31	Carrier	AMB	POS	POS	AMB	POS	AMB	POS	POS	POS	NEG	NEG	NEG	POS	AMB	NEG	NEG	AMB	NEG	NEG	NEG	NEG	POS	POS	NEG	NEG	POS	NEG	NEG	NEG	POS	POS	POS	NEG	AMB	POS
Iasi-24-P9	Carrier	AMB	POS	POS	AMB	POS	AMB	POS	POS	POS	NEG	NEG	NEG	POS	POS	NEG	NEG	POS	NEG	NEG	NEG	NEG	POS	POS	NEG	NEG	POS	NEG	NEG	NEG	POS	POS	POS	NEG	POS	POS
Iasi-27-P12	Carrier	NEG	POS	POS	AMB	POS	AMB	POS	POS	POS	NEG	NEG	NEG	POS	POS	NEG	NEG	AMB	NEG	NEG	NEG	NEG	POS	AMB	NEG	NEG	POS	NEG	NEG	NEG	POS	POS	AMB	NEG	AMB	POS
Iasi-38-P27	Carrier	AMB	POS	POS	AMB	POS	AMB	POS	POS	POS	NEG	NEG	NEG	POS	#ZAHL!	NEG	NEG	POS	NEG	NEG	NEG	NEG	POS	POS	NEG	NEG	POS	NEG	NEG	NEG	POS	POS	POS	NEG	POS	POS
Iasi-42-G511	Carrier	NEG	POS	POS	AMB	POS	AMB	POS	POS	POS	NEG	NEG	NEG	POS	AMB	NEG	NEG	AMB	NEG	NEG	NEG	NEG	POS	AMB	NEG	NEG	POS	NEG	NEG	NEG	POS	POS	POS	NEG	AMB	POS
Iasi-46-3N	Carrier	AMB	POS	POS	AMB	POS	AMB	POS	POS	POS	NEG	NEG	NEG	POS	AMB	NEG	NEG	POS	NEG	NEG	NEG	NEG	POS	POS	NEG	NEG	POS	NEG	NEG	NEG	POS	POS	POS	NEG	POS	POS
Iasi-47-42O	Carrier	AMB	POS	POS	AMB	POS	AMB	POS	POS	POS	NEG	NEG	NEG	POS	AMB	NEG	NEG	AMB	NEG	NEG	NEG	NEG	POS	AMB	NEG	NEG	POS	NEG	NEG	NEG	POS	POS	AMB	NEG	AMB	POS
Iasi-48-36N	Carrier	NEG	POS	POS	AMB	POS	AMB	POS	POS	POS	NEG	NEG	NEG	POS	AMB	NEG	NEG	AMB	NEG	NEG	NEG	NEG	POS	AMB	NEG	NEG	POS	NEG	NEG	NEG	POS	POS	AMB	NEG	AMB	POS
Iasi-49-T3	Carrier	NEG	POS	POS	AMB	POS	AMB	POS	POS	POS	NEG	NEG	NEG	POS	AMB	NEG	NEG	AMB	NEG	NEG	NEG	NEG	POS	AMB	NEG	NEG	POS	NEG	NEG	NEG	POS	POS	AMB	NEG	AMB	NEG
Iasi-50-T2	Carrier	AMB	POS	POS	AMB	POS	AMB	POS	POS	POS	NEG	NEG	NEG	POS	AMB	NEG	NEG	POS	NEG	NEG	NEG	NEG	POS	POS	NEG	NEG	POS	NEG	NEG	NEG	POS	POS	AMB	NEG	AMB	NEG
Iasi-22-251	BSI	NEG	POS	POS	AMB	POS	AMB	POS	POS	POS	NEG	NEG	NEG	POS	NEG	NEG	NEG	AMB	NEG	NEG	NEG	NEG	POS	NEG	NEG	NEG	POS	NEG	NEG	NEG	POS	POS	AMB	NEG	AMB	POS
Iasi-27-158	BSI	POS	AMB	POS	AMB	POS	AMB	POS	POS	POS	NEG	NEG	NEG	NEG	AMB	NEG	NEG	POS	NEG	POS	AMB	NEG	POS	AMB	POS	NEG	POS	NEG	POS	NEG	POS	POS	NEG	POS	AMB	POS
Iasi-08-70	BSI	NEG	POS	POS	AMB	POS	POS	NEG	POS	POS	NEG	NEG	NEG	NEG	NEG	NEG	POS	NEG	NEG	NEG	POS	POS	POS	NEG	NEG	POS	POS	POS	NEG	POS	POS	POS	POS	NEG	AMB	POS
Iasi-18-93	BSI	NEG	POS	POS	AMB	POS	POS	AMB	POS	POS	NEG	NEG	NEG	NEG	NEG	NEG	POS	AMB	NEG	NEG	POS	POS	POS	NEG	NEG	POS	POS	POS	NEG	POS	POS	POS	POS	NEG	NEG	POS
Iasi-28-P14	Carrier	NEG	POS	AMB	POS	POS	POS	NEG	POS	POS	NEG	NEG	NEG	NEG	AMB	NEG	POS	NEG	NEG	NEG	POS	POS	POS	AMB	NEG	POS	POS	POS	NEG	POS	POS	POS	POS	NEG	NEG	POS
Iasi-29-P15	Carrier	AMB	POS	POS	AMB	POS	POS	NEG	POS	POS	NEG	NEG	NEG	NEG	AMB	NEG	POS	NEG	NEG	NEG	POS	POS	POS	POS	NEG	POS	POS	POS	NEG	POS	POS	POS	POS	NEG	AMB	POS
Iasi-03-19	BSI	NEG	POS	NEG	NEG	POS	POS	NEG	POS	POS	NEG	NEG	NEG	NEG	NEG	NEG	POS	NEG	NEG	NEG	NEG	NEG	NEG	NEG	NEG	NEG	POS	NEG	AMB	NEG	POS	NEG	NEG	NEG	NEG	POS
Iasi-11-77	BSI	NEG	POS	NEG	NEG	POS	POS	AMB	POS	POS	NEG	NEG	NEG	NEG	NEG	NEG	POS	NEG	NEG	NEG	NEG	NEG	NEG	NEG	NEG	NEG	POS	NEG	NEG	NEG	AMB	NEG	NEG	NEG	NEG	POS
Iasi-13-82	BSI	NEG	POS	NEG	NEG	POS	POS	AMB	POS	POS	NEG	NEG	NEG	NEG	NEG	NEG	POS	NEG	NEG	NEG	NEG	NEG	NEG	NEG	NEG	NEG	POS	NEG	AMB	NEG	POS	NEG	NEG	NEG	NEG	POS
Iasi-15-86	BSI	AMB	POS	AMB	POS	POS	POS	AMB	POS	POS	NEG	NEG	NEG	NEG	NEG	NEG	POS	NEG	NEG	NEG	NEG	NEG	POS	AMB	NEG	NEG	POS	NEG	AMB	NEG	POS	NEG	NEG	NEG	AMB	POS
Iasi-20-103	BSI	NEG	POS	NEG	POS	POS	POS	AMB	POS	POS	NEG	NEG	NEG	NEG	NEG	NEG	POS	NEG	NEG	NEG	NEG	NEG	AMB	NEG	NEG	NEG	POS	NEG	AMB	NEG	POS	NEG	NEG	NEG	NEG	POS
Iasi-33-222	BSI	AMB	POS	AMB	POS	POS	POS	AMB	POS	POS	NEG	NEG	NEG	NEG	NEG	NEG	POS	NEG	NEG	NEG	NEG	NEG	POS	AMB	NEG	NEG	POS	NEG	AMB	NEG	POS	NEG	NEG	NEG	NEG	POS
Iasi-44-360	BSI	NEG	POS	AMB	POS	POS	POS	NEG	POS	POS	NEG	NEG	NEG	NEG	NEG	NEG	POS	NEG	NEG	NEG	NEG	NEG	AMB	NEG	NEG	NEG	POS	NEG	AMB	NEG	POS	NEG	NEG	NEG	NEG	POS
Iasi-47-399	BSI	AMB	POS	AMB	POS	POS	POS	AMB	POS	POS	NEG	NEG	NEG	NEG	NEG	NEG	POS	NEG	NEG	NEG	NEG	NEG	POS	NEG	NEG	NEG	POS	NEG	AMB	NEG	POS	NEG	NEG	NEG	NEG	POS
Iasi-34-368	SSTI	NEG	POS	AMB	POS	POS	POS	AMB	POS	POS	NEG	NEG	NEG	NEG	AMB	NEG	POS	NEG	NEG	NEG	NEG	NEG	AMB	AMB	NEG	NEG	POS	NEG	POS	NEG	POS	NEG	NEG	NEG	NEG	POS
Iasi-36-371	SSTI	NEG	POS	AMB	POS	POS	POS	AMB	POS	POS	NEG	NEG	NEG	NEG	POS	NEG	POS	NEG	NEG	NEG	NEG	NEG	AMB	NEG	NEG	NEG	POS	NEG	AMB	NEG	POS	NEG	NEG	NEG	NEG	POS
Iasi-04-S12	Carrier	AMB	POS	AMB	POS	POS	POS	AMB	POS	POS	NEG	NEG	NEG	NEG	#ZAHL!	NEG	POS	NEG	NEG	NEG	NEG	NEG	POS	POS	NEG	NEG	POS	NEG	POS	NEG	POS	NEG	NEG	NEG	POS	POS
Iasi-06-S15	Carrier	AMB	POS	AMB	POS	POS	POS	AMB	POS	POS	NEG	NEG	NEG	NEG	#ZAHL!	NEG	POS	NEG	NEG	NEG	NEG	NEG	POS	AMB	NEG	NEG	POS	NEG	POS	NEG	POS	NEG	NEG	NEG	NEG	POS
Iasi-07-S16	Carrier	AMB	POS	AMB	POS	POS	POS	AMB	POS	POS	NEG	NEG	NEG	NEG	#ZAHL!	NEG	POS	NEG	NEG	NEG	NEG	NEG	POS	AMB	NEG	NEG	POS	NEG	POS	NEG	POS	NEG	NEG	NEG	AMB	POS
Iasi-11-S24	Carrier	AMB	POS	AMB	POS	POS	POS	AMB	POS	POS	NEG	NEG	NEG	NEG	AMB	NEG	POS	NEG	NEG	NEG	NEG	NEG	POS	POS	NEG	NEG	POS	NEG	POS	NEG	POS	NEG	NEG	NEG	AMB	POS
Iasi-22-P4	Carrier	AMB	POS	AMB	POS	POS	POS	AMB	POS	POS	NEG	NEG	NEG	NEG	AMB	NEG	POS	NEG	NEG	NEG	NEG	NEG	POS	AMB	NEG	NEG	POS	NEG	AMB	NEG	POS	NEG	NEG	NEG	NEG	POS
Iasi-34-P23	Carrier	AMB	POS	NEG	POS	POS	POS	AMB	POS	POS	NEG	NEG	NEG	NEG	AMB	NEG	POS	NEG	NEG	NEG	NEG	NEG	POS	AMB	NEG	NEG	POS	NEG	POS	NEG	POS	NEG	NEG	NEG	NEG	POS
Iasi-37-P26	Carrier	AMB	POS	AMB	POS	POS	POS	AMB	POS	POS	NEG	NEG	NEG	NEG	AMB	NEG	POS	NEG	NEG	NEG	NEG	NEG	POS	POS	NEG	NEG	POS	NEG	AMB	NEG	POS	NEG	POS	NEG	AMB	POS
Iasi-30-196	BSI	AMB	POS	AMB	POS	POS	AMB	POS	POS	POS	NEG	NEG	NEG	NEG	POS	NEG	POS	NEG	NEG	NEG	NEG	NEG	POS	POS	NEG	NEG	POS	NEG	AMB	NEG	POS	NEG	NEG	NEG	POS	POS
Iasi-27-298	BSI	POS	AMB	AMB	POS	POS	NEG	POS	POS	POS	NEG	NEG	NEG	NEG	NEG	NEG	NEG	NEG	NEG	NEG	NEG	NEG	NEG	NEG	POS	NEG	POS	NEG	AMB	NEG	POS	POS	NEG	NEG	NEG	NEG
Iasi-37-363	BSI	POS	AMB	POS	AMB	POS	NEG	POS	POS	POS	NEG	NEG	NEG	NEG	NEG	NEG	NEG	NEG	NEG	NEG	NEG	NEG	NEG	NEG	POS	NEG	POS	NEG	POS	NEG	POS	POS	AMB	NEG	NEG	NEG
Iasi-38-372	BSI	POS	AMB	AMB	POS	POS	NEG	POS	POS	POS	NEG	NEG	NEG	NEG	NEG	NEG	NEG	NEG	NEG	NEG	NEG	NEG	NEG	NEG	POS	NEG	POS	NEG	AMB	NEG	POS	POS	AMB	NEG	NEG	NEG
Iasi-39-374	BSI	POS	AMB	POS	AMB	POS	AMB	POS	POS	POS	NEG	NEG	NEG	NEG	POS	NEG	NEG	NEG	NEG	NEG	NEG	NEG	NEG	AMB	POS	NEG	POS	NEG	POS	NEG	POS	POS	AMB	NEG	AMB	NEG
Iasi-28-299	SSTI	POS	AMB	AMB	POS	POS	NEG	POS	POS	POS	NEG	NEG	NEG	NEG	NEG	NEG	NEG	NEG	NEG	NEG	NEG	NEG	NEG	AMB	POS	NEG	POS	NEG	POS	NEG	POS	POS	POS	NEG	NEG	NEG
Iasi-07-65	BSI	POS	AMB	POS	AMB	POS	NEG	POS	POS	POS	NEG	NEG	POS	NEG	NEG	NEG	NEG	POS	NEG	NEG	NEG	POS	POS	NEG	NEG	NEG	POS	NEG	POS	NEG	POS	POS	NEG	NEG	AMB	NEG
Iasi-19-99	BSI	POS	AMB	POS	AMB	POS	NEG	POS	POS	POS	NEG	NEG	NEG	NEG	NEG	NEG	NEG	AMB	NEG	NEG	NEG	NEG	POS	NEG	NEG	NEG	POS	NEG	NEG	NEG	POS	POS	NEG	NEG	NEG	POS
Iasi-25-217	SSTI	POS	AMB	POS	AMB	POS	AMB	POS	POS	POS	NEG	NEG	NEG	NEG	POS	NEG	NEG	POS	NEG	NEG	NEG	NEG	POS	AMB	NEG	NEG	POS	NEG	NEG	NEG	POS	POS	NEG	NEG	POS	POS
Iasi-23-P5	Carrier	POS	AMB	POS	AMB	POS	AMB	POS	POS	POS	NEG	NEG	NEG	NEG	POS	NEG	NEG	AMB	NEG	NEG	NEG	NEG	POS	AMB	NEG	NEG	POS	NEG	NEG	NEG	POS	POS	NEG	NEG	AMB	POS
Iasi-43-G513	Carrier	POS	AMB	POS	AMB	POS	AMB	POS	POS	POS	NEG	NEG	NEG	NEG	POS	NEG	NEG	AMB	NEG	NEG	NEG	NEG	POS	AMB	NEG	NEG	POS	NEG	NEG	NEG	POS	POS	NEG	NEG	AMB	POS
Iasi-44-G514	Carrier	POS	AMB	POS	AMB	POS	AMB	POS	POS	POS	NEG	NEG	NEG	NEG	AMB	NEG	NEG	AMB	NEG	NEG	NEG	NEG	POS	AMB	NEG	NEG	POS	NEG	NEG	NEG	POS	POS	NEG	NEG	POS	POS
Iasi-28-172	BSI	POS	AMB	POS	AMB	POS	AMB	POS	NEG	AMB	POS	POS	NEG	POS	AMB	NEG	NEG	AMB	NEG	NEG	POS	NEG	POS	AMB	NEG	NEG	POS	POS	POS	POS	NEG	NEG	NEG	NEG	POS	POS
Iasi-42-358	BSI	POS	AMB	POS	AMB	POS	AMB	POS	NEG	AMB	POS	POS	NEG	POS	POS	NEG	NEG	AMB	NEG	NEG	POS	NEG	POS	AMB	NEG	NEG	POS	POS	AMB	POS	NEG	NEG	NEG	NEG	POS	POS
Iasi-48-403	BSI	POS	AMB	POS	AMB	POS	AMB	POS	NEG	NEG	POS	POS	NEG	POS	AMB	NEG	NEG	NEG	NEG	NEG	POS	NEG	POS	NEG	NEG	NEG	POS	POS	AMB	POS	NEG	NEG	NEG	NEG	AMB	POS
Iasi-26-153	BSI	AMB	POS	POS	AMB	POS	AMB	POS	NEG	NEG	POS	POS	NEG	POS	AMB	NEG	NEG	NEG	NEG	NEG	POS	NEG	POS	NEG	NEG	NEG	POS	POS	AMB	POS	NEG	NEG	NEG	NEG	NEG	POS
Iasi-29-190	BSI	POS	AMB	POS	AMB	POS	AMB	POS	NEG	NEG	POS	POS	NEG	POS	NEG	NEG	NEG	NEG	NEG	NEG	POS	NEG	POS	NEG	NEG	NEG	POS	POS	AMB	POS	NEG	NEG	NEG	NEG	AMB	POS
Iasi-31-201	BSI	AMB	POS	POS	AMB	POS	NEG	POS	NEG	NEG	POS	POS	NEG	POS	POS	NEG	NEG	NEG	NEG	NEG	POS	NEG	POS	AMB	NEG	NEG	POS	POS	NEG	POS	NEG	NEG	NEG	NEG	NEG	POS
Iasi-37-289	BSI	POS	AMB	POS	AMB	POS	AMB	POS	NEG	AMB	POS	POS	NEG	POS	POS	NEG	NEG	NEG	NEG	NEG	POS	NEG	POS	AMB	NEG	NEG	POS	POS	AMB	POS	NEG	NEG	NEG	NEG	POS	POS
Iasi-41-334	BSI	POS	AMB	POS	AMB	POS	AMB	POS	NEG	AMB	POS	POS	NEG	POS	POS	NEG	NEG	POS	NEG	NEG	POS	NEG	POS	POS	NEG	NEG	POS	POS	POS	POS	NEG	NEG	NEG	NEG	POS	POS
Iasi-52-433	BSI	POS	AMB	POS	AMB	POS	NEG	POS	NEG	NEG	POS	POS	NEG	POS	NEG	NEG	NEG	NEG	NEG	NEG	POS	NEG	POS	NEG	NEG	NEG	POS	POS	NEG	POS	NEG	NEG	NEG	NEG	NEG	POS
Iasi-07-66	SSTI	POS	AMB	POS	AMB	POS	NEG	POS	NEG	NEG	POS	POS	NEG	POS	NEG	NEG	NEG	NEG	NEG	NEG	POS	NEG	POS	NEG	NEG	NEG	POS	POS	AMB	POS	NEG	NEG	NEG	NEG	NEG	POS
Iasi-23-211	SSTI	POS	AMB	POS	AMB	POS	AMB	POS	NEG	NEG	POS	POS	NEG	POS	AMB	NEG	NEG	NEG	NEG	NEG	POS	NEG	POS	NEG	NEG	NEG	POS	POS	AMB	POS	NEG	NEG	NEG	NEG	NEG	POS
Iasi-43-401	SSTI	POS	AMB	POS	AMB	POS	NEG	POS	NEG	NEG	POS	POS	NEG	POS	AMB	NEG	NEG	NEG	NEG	NEG	POS	NEG	POS	NEG	NEG	NEG	POS	POS	AMB	POS	NEG	NEG	NEG	NEG	NEG	POS
Iasi-47-418	SSTI	POS	AMB	POS	AMB	POS	NEG	POS	AMB	NEG	POS	POS	NEG	POS	AMB	NEG	NEG	NEG	NEG	NEG	POS	NEG	POS	NEG	NEG	NEG	POS	POS	AMB	POS	NEG	NEG	NEG	NEG	NEG	POS
Iasi-18-169	SSTI	POS	AMB	NEG	NEG	POS	AMB	POS	NEG	NEG	POS	POS	NEG	NEG	AMB	NEG	NEG	POS	NEG	NEG	POS	NEG	AMB	POS	NEG	NEG	POS	NEG	NEG	NEG	POS	POS	NEG	NEG	NEG	POS
Iasi-18-S34	Carrier	POS	AMB	POS	AMB	POS	AMB	POS	POS	POS	NEG	NEG	NEG	NEG	POS	NEG	NEG	POS	NEG	NEG	POS	NEG	POS	POS	NEG	NEG	POS	NEG	POS	NEG	POS	POS	NEG	NEG	POS	NEG
Iasi-36-276	BSI	NEG	POS	NEG	POS	POS	POS	AMB	POS	POS	NEG	NEG	NEG	POS	AMB	NEG	NEG	AMB	NEG	NEG	NEG	NEG	POS	NEG	POS	NEG	POS	NEG	NEG	NEG	POS	POS	NEG	NEG	NEG	NEG
Iasi-151	BSI	AMB	POS	AMB	POS	POS	POS	AMB	POS	POS	NEG	NEG	NEG	NEG	POS	NEG	NEG	NEG	NEG	NEG	NEG	NEG	POS	POS	NEG	NEG	POS	POS	NEG	POS	NEG	NEG	POS	NEG	POS	NEG
Iasi-02-11	BSI	AMB	POS	NEG	NEG	POS	AMB	POS	NEG	NEG	POS	POS	NEG	NEG	NEG	NEG	NEG	NEG	NEG	NEG	POS	NEG	POS	NEG	POS	POS	POS	POS	AMB	POS	NEG	NEG	NEG	NEG	AMB	NEG
